# The Baeyer–Villiger Oxidation of Cubyl Ketones: A Synthetic Route to Functionalized Cubanols

**DOI:** 10.1021/acs.orglett.5c02760

**Published:** 2025-08-08

**Authors:** Jasmine Hind, Ian. A. Fallis, James. A. Platts, Matthew Tredwell

**Affiliations:** † School of Chemistry, 2112 Cardiff University, Main Building, Park Place, Cardiff, CF10 3AT, U.K.; ¶ Wales Research and Diagnostic PET Imaging Centre, Cardiff University, University Hospital of Wales, Heath Park, Cardiff, CF14 4XN, U.K.

## Abstract

The synthesis of functionalized cubanols has been developed via a 2-step protocol comprising of a Baeyer–Villiger oxidation of cubyl ketones, followed by acid hydrolysis. As part of this study, we determine the relative migratory aptitude of cubyl groups in the Baeyer–Villiger oxidation and rationalize these experimental findings with computational studies.

## Graphical abstract

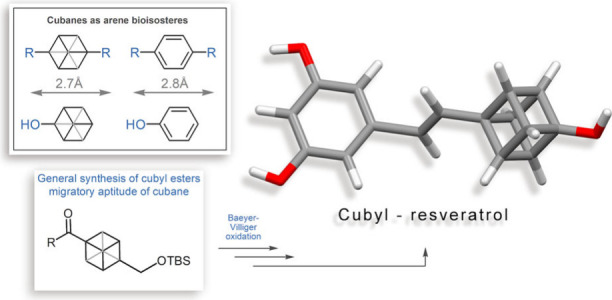

Strained hydrocarbon cages such as cubane (pentacyclo­[4.2.0.0^2,5^.0^3,8^.0^4,7^]­octane) and the related bicyclo[1.1.1]­pentane are used as bioisosteres of para-substituted phenyl rings in drug discovery.^1^ Interest in these hydrocarbon scaffolds has advanced novel chemical methods for their incorporation and functionalization in complex molecules.^2^ Since phenols and phenyl ethers are common functional groups in both pharmaceuticals and natural products, cubyl alcohols and ethers are of central interest in developing new medicines.^3^ Yet literature reports on the synthesis and stability of cubanols are inconclusive. Eaton reported that 4-methylcubanol ring-opens by homoketonization but can be isolated with care,^4^ while Zwanenburg reported that 4-bromocubanol could be isolated in 60% yield via hydrolysis of the corresponding diazonium salt, but it is unstable to treatment with KOH.^5,6^ Isolated enzymatic methods have been reported, but these are low-yielding and unselective.^7^ Methods for the synthesis of cubyl ethers are also rare; Linclau and Brown reported a method to access alkoxy cubanes under electrochemical flow conditions via a Hofer-Moest reaction,^8^ while isolated examples of cubyl ethers from the photolysis of cubyl iodides have been reported.^9^ We were interested in developing a general method toward cubanols that allowed us to investigate their suitability as bioisosteres for phenols. We hypothesized that a Baeyer–Villiger (BV) rearrangement of unsymmetric cubyl ketones may provide a straightforward transformation to cubyl esters, which upon hydrolysis yield cubanols.

The BV oxidation of cubyl methyl ketones has been reported to give exclusively the cubyl acetate resulting from the migration of the cubyl group.^10^ In contrast, the BV rearrangement of a cubyl phenyl ketone was reported to proceed with exclusive migration of the phenyl group (Figure 1).^11^ Considering the opposing migratory aptitudes of these two cubyl ketone systems, we performed a systematic examination of the BV reaction of a series of functionalized unsymmetric cubyl ketones to assess the relative impact of steric and electronic factors, both of which have been shown to influence migratory aptitude during BV rearrangements.^12^ We began our studies with the BV reaction of ketone **1a** (Table 1).^13^ Treatment of **1a** with 4 equiv of *m*-chloroperbenzoic acid (*m*-CPBA) at room temperature (rt) for 24 h resulted in 74% conversion of the starting ketone, with preferential formation of ester **2a** (46% yield) resulting from migration of cubane (Entry 1). A small quantity of the phenyl migration product **3a** was observed in 4% yield. Increasing the quantity of *m*-CPBA to 8 equiv led to increased conversion of starting material with predominant formation of **2a** in 72% yield compared to 5% of **3a** (Entry 2). To increase the extent of conversion without the need for a large excess of peracid we opted to use Lewis acid catalysts reported to promote BV reactions.^14^ The use of 10 mol % BF_3_·OEt_2_ as a catalyst at 50 °C resulted in 84% consumption of the starting material within 6 h but with overall decreased ester formation (Entry 3). The use of Sc­(OTf)_3_ (10 mol %) with *m*-CPBA (4 equiv) in CHCl_3_ at 50 °C for 6 h gave 87% conversion of **1a** and a 51% yield of **2a**, again with only trace quantities of **3a** (Entry 4).

**Figure 1:**
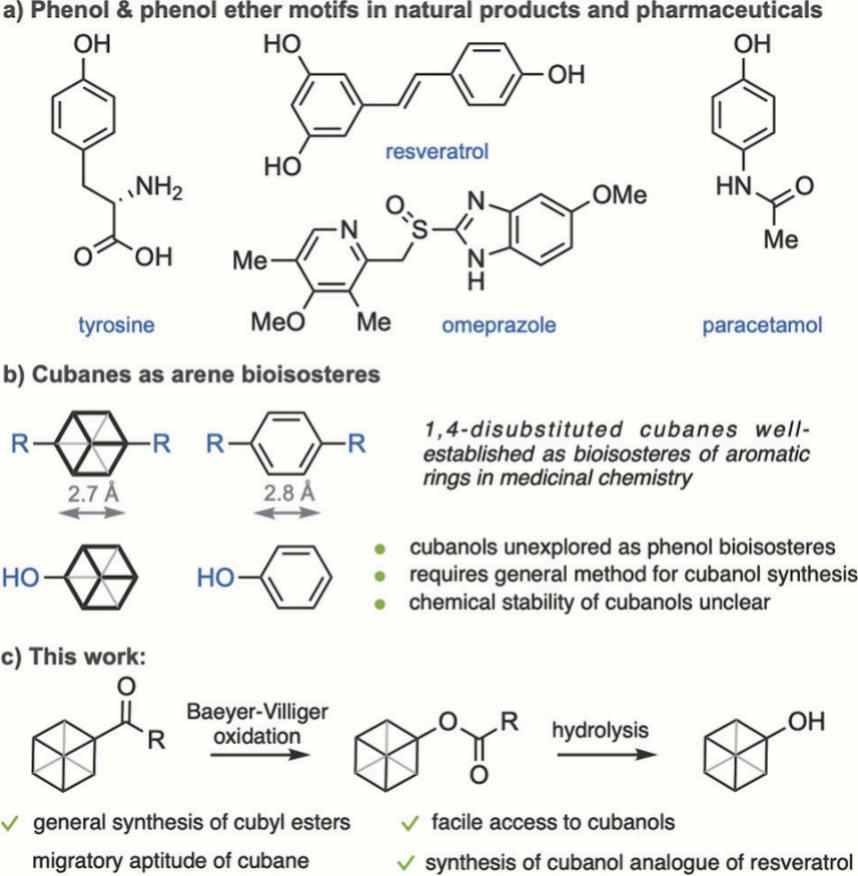
a) Phenols and phenol ether motifs. b) Cubane bioisosteres. c) This work.

**Table 1:**
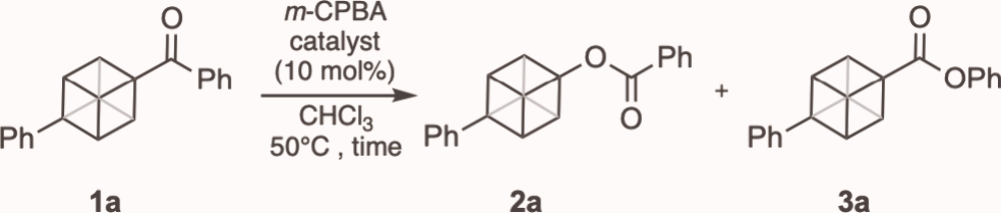
Optimization of Baeyer-Villiger Oxidation^a^

Entry	*m*-CPBA (equiv)	Catalyst	Time (hr)	Conversion **1a** (%)^b^	**2a** (%)^b^	**3a** (%)^b^
1^c^	4	none	24	74	46	4
2^c^	8	none	24	90	72	5
3	4	BF_3_·OEt_2_	6	84	51	3
4	4	Sc(OTf)_3_	6	87	51	3
5	4	Sc(OTf)_3_	3	65	46	3
6	1.7	Sc(OTf)_3_	3	29	22	2

a Reactions performed on 0.11 mmol of **1a** in 1 mL CHCl_3_.

b Yields determined by ^1^H NMR.

c Room temperature.

Decreasing the reaction time has a minor impact on conversion (Entry 5), while decreasing the quantity of *m*-CPBA to 1.7 equiv resulted in low conversions (Entry 6). In all cases, the major product observed was **2a**, a result of preferential migration of the cubyl group over the aryl ring. To verify that these yields were an accurate representation of the migratory aptitude rather than the stability of the products to the reaction conditions, we independently subjected **2a** and **3a** to the conditions described in Entry 4; for both substrates >70% of the material was reisolated, confirming that the yields represent a good indication of migratory aptitude. The migratory aptitude of cubyl > phenyl observed for **1a** is in contrast to the selectivity observed by Eaton using an amide-substituted cubyl ketone,^11^ although it should be noted that migratory aptitude in BV rearrangements can be dependent on the reaction conditions making direct comparisons between studies difficult.^12a^ Employing Eaton’s conditions (2 equiv of CF_3_CO_3_H, in 1 mL of DCM at room temperature for 1 h) resulted in predominant decomposition (66% **1a** consumed) and only 3% of **2a**, with **3a** and the corresponding carboxylic acid not observed.

Having established conditions for the BV reaction that allowed us to determine the selective distribution of the two ester products, we opted to use the conditions described in Entry 4 (Table 1) for further studies as this minimized the reaction time and quantity of oxidant needed. We next examined the relative migratory aptitude of cubane against a range of saturated alkyl groups (Table 2). The unsymmetrical cubyl ketone bearing a methyl group **1b** (Entry 1) gave only the ester **2b** in 79% yield, resulting from completely selective migration of the cubyl group; a similar result was found for ethyl ketone **1c** (Entry 2) yielding **2c** as the only product. The BV reaction of 1-phenyl cubyl 4-aldehyde (**1d**, Entry 3) gave a 2:1 mixture of the formate ester **2d** and carboxylic acid **3d**. Competition between an *i*-propyl group and a cubyl group still resulted in useful preferential cubyl migration, but only by a 2:1 ratio with significant quantities of **3e** formed (Entry 4). In contrast to all others (**1b**−**1e**) where complete consumption of the starting material was observed, the BV reaction with the highly hindered *t*-butyl ketone **1f** resulted in only 36% conversion, with a trace quantity of **3f** being formed (Entry 5). We were unable to unambiguously identify **2f** in the crude ^1^H NMR, allowing us to tentatively assign *t-*Bu as having a higher migratory aptitude. These migratory aptitudes for alkyl groups established in this study are consistent with previous reports, and under the conditions described they allow us to place cubane in this BV migratory aptitude sequence as follows; *t*-butyl > cubyl > *i*-propyl ≈ hydrogen > phenyl > ethyl > methyl.

**Table 2:**
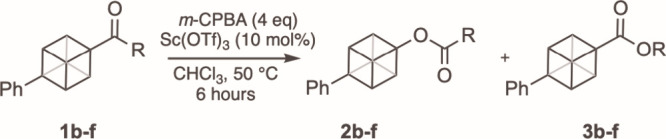
Determination of Migratory Aptitude

Entry	Substrate	R	Yield **2** (%)^b^	Yield **3** (%)^b^	Ratio (**2**:**3**)
1	**1b**	Me	79 (86)	0 (nd)	>99:1
2	**1c**	Et	87 (76)	0 (nd)	>99:1
3	**1d**	H	55 (50)	28 (nd)	2:1
4	**1e**	*i-*Pr	58 (62)	25 (27)	2:1
5	**1f**	*t*-Bu	0 (nd)	8 (nd)	nd

a Reactions performed with 0.2 mmol of substrate in 2 mL of CHCl_3_.

b Yields determined by ^1^H NMR, isolated yields are in parentheses; nd = not determined.

We were interested to see how substituents on the cubyl framework affected the reactivity and relative migratory aptitude of the cubane group as a prelude to using this BV methodology in preparing structurally complex cubane ether and alcohol bioisosteres. A series of isopropyl cubyl ketones (**1g** – **1l**) were prepared bearing a range of functional groups but maintaining the presence of the *i*-propyl group as the competitive migrating partner (Table 3). We continued to use the conditions detailed in Table 1, Entry 4; under these conditions, the trimethylsilyl-substituted cubane (**1g**, Table 3, Entry 1) resulted in a 65% NMR yield of **2g**, with a ratio of **2g**:**3g** of 7:1. This result shows a noticeably higher migratory aptitude of the cubyl component relative to the model substrate **1e** (cf. **2e**:**3e** = 2:1). The unsubstituted cubane **1h** (Table 3, Entry 2) yielded 44% and 15% of **2h** and **3h**, respectively; a similar result was observed for the ether-containing compound **1i** (Entry 3), with cubyl migration dominating in a 3:1 ratio of **2i**:**3i**. The presence of electron-withdrawing substituents on the cubane skeleton was found to decrease the relative migratory aptitude. Diisopropylamide-substituted compound **1j** (Entry 4) resulted in a **2j**:**3j** ratio of 1:1, while halogens had a more profound effect with both the bromo- and fluoro-substituted compounds producing the *i*-propyl esters as the predominant product (**2**:**3** = 1:4) (Entries 5 and 6). The experimental results observed are consistent with previous studies where the migrating group adopts an antiperiplanar arrangement with the O–O bond of the carboxylate leaving group in the Criegee intermediate, the so-called *primary stereoelectronic effect*.^12e,12f^

**Table 3:**
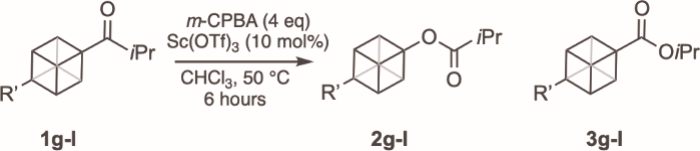
Effect of the Cubyl Substituent on Migratory Aptitude

Entry	Substrate	R’	Yield **2** (%)^b^	Yield **3** (%)^b^	Ratio (**2**:**3**)
1	**1g**	SiMe_3_	65 (61)	9 (10)	7:1
2	**1h**	H	44 (46)	15 (22)	3:1
3	**1i**	CH_2_OMe	44 (42)	15 (8)	3:1
4	**1j**	C(O)N(*i-*Pr)_2_	25 (22)	29 (9)	1:1
5	**1k**	Br	12 (9)	50 (46)	1:4
6	**1l**	F	11 (10)	49 (39)	1:4

a Reactions performed with 0.2 mmol of substrate in 2 mL CHCl_3_.

b Yields determined by ^1^H NMR, isolated yields are in parentheses.

The migratory aptitude of alkyl groups in BV reactions is then rationalized by considering the lowest-energy conformer that places the sterically largest group antiperiplanar to the O–O bond in the Criegee intermediate, thus minimizing steric interactions in the corresponding transition state.^12a,12b^ Our calculations found that for substrate **1e**, placing the cubyl group antiperiplanar to the O–O bond is 2.9 kJ/mol lower in free energy than the *i-*Pr antiperiplanar, leading to a 77/23 Boltzmann weighting that is in line with the observed 2:1 selectivity for the formation of **2e** over **3e**.^15^ The data in Table 3 demonstrates there is also an electronic component to the migratory aptitude, as was previously reported in the migration of substituted arenes.^12a,16^ The presence of a trimethylsilyl group at the 4-position of the cubane ring increases the migratory aptitude (**2g**/**3g** = 7:1) presumably by stabilization of the positive charge as a result of migration of this group in the transition state. Conversely the presence of electron-withdrawing substituents, such as fluorine (**2l**/**3l** = 1:4), decreases the migratory aptitude of the cubane ring. We performed calculations on the stability of the 4-substituted cubane cations that demonstrated that a trimethylsilyl group stabilized the cubane cation by 22.8 kJ/mol relative to the unsubstituted species, whereas fluorine destabilized the cation by 52.1 kJ/mol.^15^ Our experimental data and computation support the proposal by Schiesser and Della on the stabilization of cubyl cations by hyperconjugation of the α-β and β-γ C–C bonds.^17^

Given the scarcity of data on the synthesis and stability of cubanols, it was unclear how useful these compounds could be as phenol bioisosteres or as building blocks. We proposed that the hydrolysis of cubanyl esters may allow us to address these questions. The reduction of a cubyl acetate by diisobutylaluminum hydride (DIBAL) to a cubanol followed by *in situ* conversion to the corresponding triflate by trifluoromethanesulfonic anhydride has been reported by Eaton.^18^ In our hands, attempts to access cubanols directly with DIBAL either resulted in recovery of starting material or decomposition. To avoid decomposition of the cubanol under basic conditions we changed strategy, and pleasingly, hydrolysis of **2b** with 1.25 M HCl in ethanol resulted in complete hydrolysis of the acetate ester to the cubanol **4b** within 3 h at room temperature (Figure 2a).^15^ To test the usefulness of this method to access cubanols, we applied the Baeyer–Villiger/hydrolysis strategy to a cubyl derivative of phenol-containing natural product resveratrol (Figure 2b). The Baeyer–Villiger reaction of ketone **5** proceeded as expected with exclusive migration of the cubyl group to yield acetate **6** in good yield on a 2 mmol scale. Deprotection, followed by oxidation, provided the desired aldehyde functionality to install the alkene via a Wittig reaction in 34% yield (*E*/*Z* = 13:1). Employing our optimized hydrolysis conditions from above gave the target compound **8** in 38% yield. It is noteworthy that cubanol **4b** began to show decomposition products by ^1^H NMR in CDCl_3_ after an hour at room temperature but remained stable in acetic-d_3_ acid for over 24 h at room temperature. Cubanol **8** was also stable to storage in DMSO-*d*_6_. Further studies are underway to assess the stability of these compounds and their utility as intermediates in the synthesis of functionalized cubanes.

**Figure 2:**
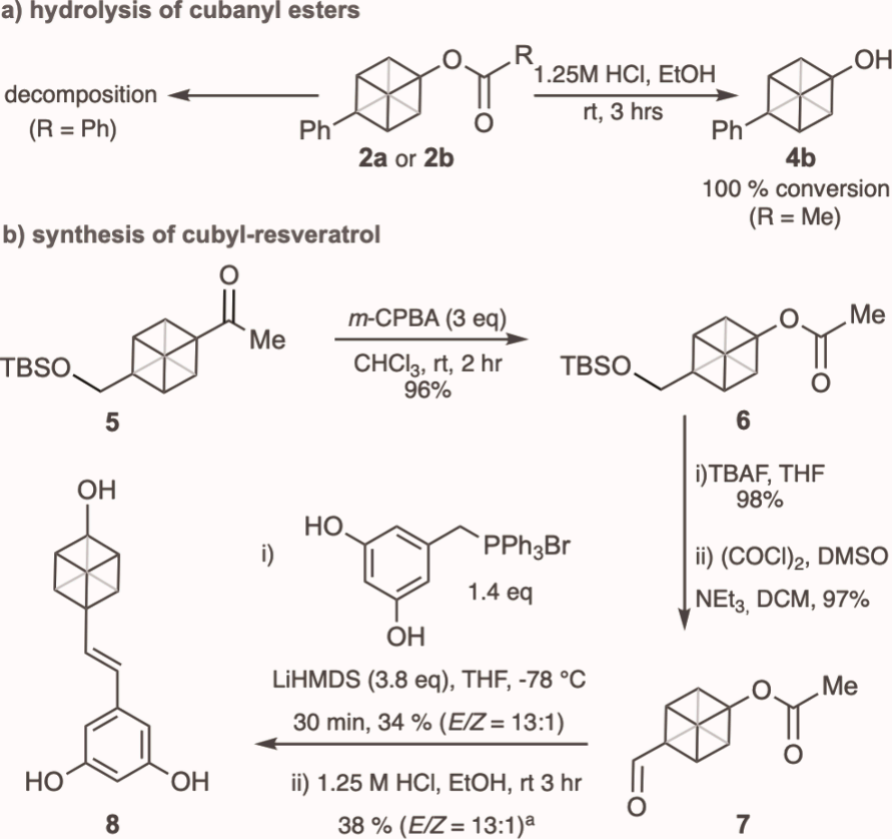
a) Hydrolysis of cubanyl esters; synthesis of cubyl resveratrol analogue. TBS = *t-*butyldimethylsilyl; TBAF = tetrabutylammonium fluoride; ^a^ yield determined by ^1^H NMR.

In summary, we have determined a relative migratory aptitude of cubane in the Baeyer–Villiger reaction of acyclic cubyl ketones and probed the influence of substituents on the migratory aptitude. We demonstrate how the resultant cubyl esters are also useful synthetic building blocks used to access complex cubanols.

## Supplementary Material



## Data Availability

The data underlying this study are available in the published article, in its Supporting Information, and openly available in the Cardiff University data catalogue at 10.17035/cardiff.28839242.

## Notes

Experimental protocols, optimization data, characterization of compounds, and NMR spectra (PDF)
