# Supplementary material for: The Baeyer–Villiger Oxidation of Cubyl Ketones: A Synthetic Route to Functionalized Cubanols
Source: Org Lett. 2025 Aug 8;27(33):9218–22. doi: 10.1021/acs.orglett.5c02760 (PMC12379164; doi:10.1021/acs.orglett.5c02760)

Supporting Information

The Baeyer-Villiger oxidation of cubyl ketones: a synthetic route to functionalised cubanols

Jasmine Hind[a], Ian A. Fallis[a], James. A. Platts[a] and Matthew Tredwell[a,b,*]

[a] School of Chemistry, Cardiff University, Main Building, Park Place, Cardiff, CF10 3AT, U.K.

[b] Wales Research and Diagnostic PET Imaging Centre, Cardiff University, University Hospital of Wales, Heath Park, Cardiff, CF14 4XN, U.K.

[*] *Correspondence should be addressed to tredwellm@cardiff.ac.uk*

Contents **Page No.**

General information	S2
Experimental	
Preparation of Baeyer-Villiger substrates	S3 - S25
Baeyer-Villiger optimisation	S25 - S26
Baeyer-Villiger general procedure	S26 – S27
Baeyer-Villiger substrate scope	S27 - S42
Cubanol screening	S43 - S44
Synthesis of cubyl-resveratrol	S44 – S51
Computational details	S52 - S54
References	S54
NMR spectra	S55 - S119

General Experimental

General information: ^{1}H, ^{13}C and ^{19}F NMR spectra were obtained on either a Bruker Avance 300 (300 MHz ^{1}H, 75 MHz ^{13}C), Bruker Avance 400 (400 MHz ^{1}H, 101 MHz ^{13}C, 376 MHz ^{19}F) or a Bruker Avance 500 (500 MHz ^{1}H, 126 MHz ^{13}C, 471 MHz ^{19}F) spectrometer at rt in the solvent stated. Chemical shifts (δ) for protons and carbons are reported in parts per million (ppm) relative to the residual deuterated solvent signal. Data has been reported as follows: chemical shift, multiplicity (s = singlet, brs = broad singlet, d = doublet, t = triplet, q = quartet, m = multiplet), coupling constants (Hz) and integration. High resolution mass spectrometry (HRMS, m/z) data was acquired at Cardiff University. ES/EI/CI HRMS data was collected on a Thermo Scientific Exactive GC machine with an orbitrap mass analyser or a Waters Xevo G2XS. TLC analysis was performed on commercially prepared 60 F_{254} silica gel plates and visualized by ultraviolet light (254 nm), followed by staining with 1% aqueous $KMnO_4$ solution. Flash chromatography used silica gel 60 (230-400 mesh) in the solvent system stated. IUPAC names were obtained using the ChemDraw service. Weighing was performed with a 4 or 5 decimal place balance. All reagents were used directly as obtained commercially unless otherwise noted. For reactions that required heating a heating mantle was used. Blue LED photoreactor (Aldrich® Micro Photochemical Reactor blue LED (ALDKIT001) were purchased from Merck. The irradiation vessel material was borosilicate glass and the distance of irradiation vessel from light source was 5 cm. Unless otherwise stated, all glassware was dried in a 125 °C oven before use and all reactions were performed under an atmosphere of nitrogen. All anhydrous solvents were purchased from Fisher Scientific, over molecular sieves in Acroseal bottles. All cubanol compounds were under an atmosphere of nitrogen and storred in the freezer (- 5 °C).

Experimental Information

Synthesis of Baeyer-Villiger oxidation substrates

Synthesis of 1a-f:

1-(1,3-Dioxoisoindolin-2-yl) 4-methyl-cubane-1,4-dicarboxylate (**S2**)

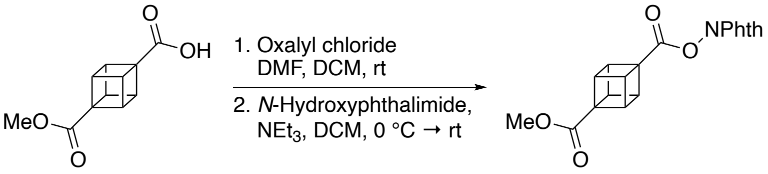

S1 **S2**

To a solution of the commercially available 4-(methoxycarbonyl)cubane-1-carboxylic acid (**S1**) (1.5 g, 1.0 equiv., 7.27 mmol) in anhydrous DCM (30 mL) was added oxalyl chloride (4.36 mL, 1.2 equiv., 2 M in DCM) at rt. Anhydrous DMF (55 µL, 10 mol%) was then added and the reaction mixture was allowed to stir at rt for 1.5 h. The mixture was concentrated *in vacuo* to afford the acid chloride as a light yellow solid.

The acid chloride was dissolved in anhydrous DCM (15 mL) and added dropwise to a solution of *N*-hydroxyphthalimide (1.4 g, 1.2 equiv., 8.73 mmol) and triethylamine (2.53 mL, 2.5 equiv., 18.2 mmol) in anhydrous DCM (15 mL) at 0 °C. After the addition, the reaction mixture was warmed to rt and stirred for a further 2 h. The reaction was quenched with sat. $NH_4Cl_{(aq)}$ (15 mL) and diluted with DCM (20 mL). The organic layer was washed with H_2O (4 x 20 mL), dried over $MgSO_4$, filtered, and concentrated *in vacuo* to give the crude. The beige solid was washed with hexane (3 x 5 mL) to afford the title compound **S2** (2.39 g, 94 %) as a white solid.

^{1}H NMR (400 MHz, $CDCl_3$) δ = 7.94 – 7.85 (m, 2H), 7.85 – 7.75 (m, 2H), 4.52 – 4.44 (m, 3H), 4.41 – 4.33 (m, 3H), 3.73 (s, 3H); **^{13}C NMR** (101 MHz, $CDCl_3$) δ = 171.6, 167.0, 162.1, 134.9, 129.1, 124.1, 55.9, 53.1, 51.9, 47.8, 47.7; **HRMS (ESI$^+$)** m/z: $[M+H]^+$ Calcd. for $C_{19}H_{14}NO_6$ 352.0816; Found 352.0823. All spectroscopic data were in accordance with the literature.[1]

Methyl-4-phenylcubane-1-carboxylate (S3)

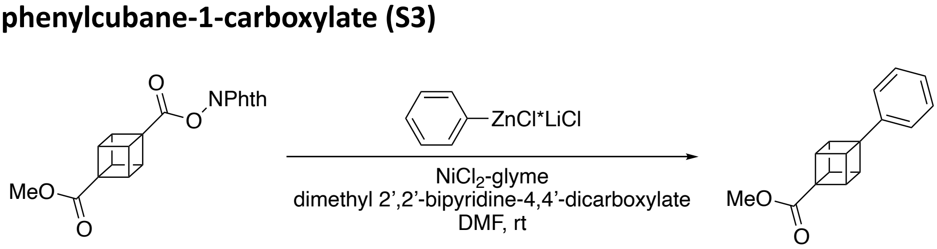

S2 **S3**

Following the procedure of Bernhard and co-workers.[2] To a solution of magnesium turnings (235 mg, 1.25 equiv., 10 mmol) and LiCl (425 mg, 1.25 equiv., 10 mmol) in anhydrous THF (2

mL), was added 1 pellet of iodine. To this mixture bromobenzene (0.84 mL, 1.0 equiv., 8 mmol) in anhydrous THF (6 mL) was added dropwise. After the addition was complete, the black mixture was left to stir at rt for 30 minutes. The newly formed Grignard was then added dropwise to a solution of $ZnCl_2$ (1.1 g, 1.0 equiv., 8 mmol) in anhydrous THF (8 mL) and was stirred for 15 minutes at rt.

The organozinc mixture (13.5 mL, 3.5 equiv., 6.76 mmol) was added to a solution of **S2** (678 mg, 1.0 equiv., 1.93 mmol), $NiCl_2$-glyme (425 mg, 1.0 equiv., 1.93 mmol), dimethyl 2,2'-bipyridine-4,4'-dicarboxylate (1.05 g, 2.0 equiv., 3.86 mmol) in anhydrous DMF (13.8 mL) in one quick addition. The purple mixture was stirred at rt for 2 hours and quenched with 1 M HCl (10 mL). To the mixture was added EtOAc (30 mL) and the organic layer was washed with H_2O (3 x 15 mL), sat. brine (3 x 15 mL), dried with anhydrous $MgSO_4$, filtered, and concentrated *in vacuo* to afford the crude. Purification by silica gel column chromatography (02:98 EtOAc/hexane) gave the title compound **S3** (244 mg, 53 %) as a yellow solid.

^{1}H NMR (500 MHz, $CDCl_3$) δ 7.40 – 7.32 (m, 2H), 7.25 – 7.17 (m, 3H), 4.28 – 4.21 (m, 3H), 4.20 – 4.12 (m, 3H), 3.74 (s, 3H); **HRMS (APCI$^+$)** m/z: $[M+H]^+$ Calcd. for $C_{16}H_{15}O_2$ 239.1067; Found 239.1067. All spectroscopic data were in accordance with the literature.[1]

4-Phenylcubane-1-carboxylic acid (S4)

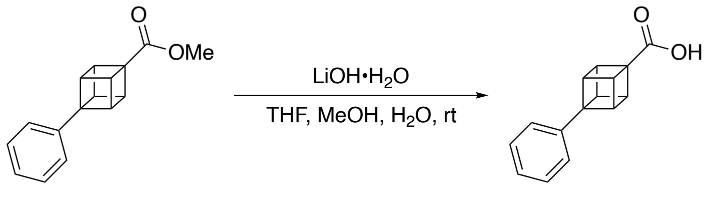

S3 **S4**

To a solution of methyl-4-phenylcubane-1-carboxylate (**S3**, 244 mg, 1.0 equiv., 1.0 mmol) in 3:3:1 ratio of THF:MeOH:H_2O (11.3 mL), was added lithium hydroxide monohydrate (230 mg, 9.6 mmol) in one-portion at 0 °C . The mixture was warmed to rt and stirred overnight. The mixture was acidified with 2 M HCl, and the aqueous layer was extracted with EtOAc (3 x 15 mL). The combined organic layers were washed sat. brine (15 mL), dried with anhydrous $MgSO_4$, filtered, and concentrated *in vacuo* to afford the crude. The crude solid was washed with hexane (3 x 5 mL) to afford the title compound **S4** (218 mg, 95 %) as a white solid.

^{1}H NMR (500 MHz, $CDCl_3$) δ 11.89 (br s, 1H), 7.42 – 7.34 (m, 2H), 7.25 – 7.19 (m, 3H), 4.36 – 4.27 (m, 3H), 4.24 – 4.16 (m, 3H); **^{13}C NMR** (126 MHz, $CDCl_3$) δ 178.8, 142.0, 128.6, 126.4, 124.9, 60.4, 56.3, 48.9, 46.2. All spectroscopic data were in accordance with the literature.[3]

_N_-Methoxy-_N_-methyl-4-phenylcubane-1-carboxamide (S5)

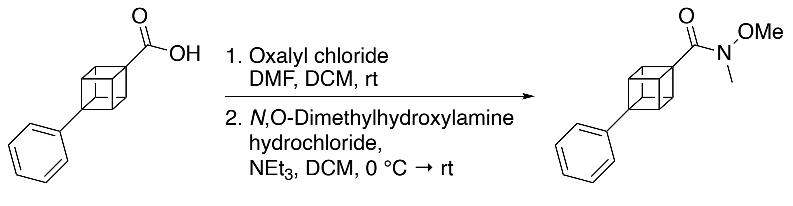

S4 **S5**

To a solution of 4-phenylcubane-1-carboxylic acid (**S4**) (218 mg, 1.0 equiv., 0.97 mmol) in anhydrous DCM (5 mL) was added oxalyl chloride (0.58 mL, 1.2 equiv., 2 M in DCM) at rt. Anhydrous DMF (7.5 μL, 10 mol%) was then added and the reaction mixture was allowed to stir at rt for 1.5 h. The mixture was concentrated *in vacuo* to afford the acid chloride as a yellow solid.

The acid chloride was dissolved in anhydrous DCM (2 mL) and added dropwise to a solution of *N*,O-dimethylhydroxylamine hydrochloride (130 mg, 1.4 equiv., 1.33 mmol) and triethylamine (0.34 mL, 2.5 equiv., 2.43 mmol) in anhydrous DCM (2 mL) at 0 °C. The mixture was warmed to rt and stirred for 3 h. The mixture was quenched with sat. $NH_4Cl_{(aq)}$ (5 mL) and diluted with DCM (10 mL). The organic layer was washed with 1 M HCl (10 mL), sat. brine (10 mL), dried with anhydrous $MgSO_4$, and concentrated *in vacuo* to afford the crude. Purification by silica gel column chromatography (1:3 EtOAc/hexane) gave the title compound **S5** (210 mg, 81 %) as a white solid.

^{1}H NMR (500 MHz, $CDCl_3$) δ 7.40 – 7.32 (m, 2H), 7.25 – 7.17 (m, 3H), 4.30 – 4.23 (m, 3H), 4.18 – 4.10 (m, 3H), 3.75 (s, 3H), 3.22 (s, 3H); **^{13}C NMR** (126 MHz, $CDCl_3$) δ 173.8, 142.5, 128.5, 126.1, 124.9, 61.8, 59.8, 58.4, 48.8, 46.3, 32.8; **HRMS (CI$^+$)** m/z: $[M+H]^+$ Calcd. for $C_{17}H_{18}O_2N$ 268.1332; Found 268.1333.

Phenyl(4-phenylcuban-1-yl)methanone (1a)

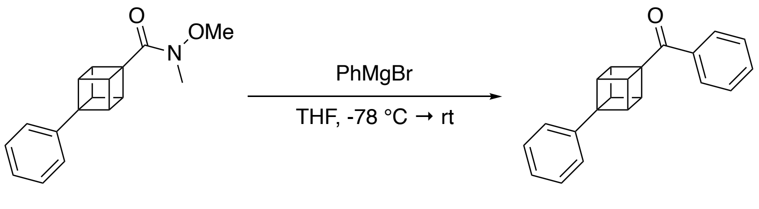

S5 **1a**

To a solution of *N*-methoxy-*N*-methyl-4-phenylcubane-1-carboxamide (**S5**) (264 mg, 1.0 equiv., 0.99 mmol) in anhydrous THF (5 mL) at -78 °C was added phenylmagnesium bromide (1.48 mL, 1.5 equiv., 1M in THF) dropwise. The mixture was stirred at -78 °C for 30 minutes and then allowed to warm to rt. After 30 minutes, the mixture was quenched with 1 M HCl (5 mL) at 0 °C. The aqueous was extracted with EtOAc (3 x 10 mL) and the combined organic layers were washed with sat. brine (10 mL), dried with anhydrous $MgSO_4$, filtered, and concentrated *in vacuo* to afford the crude. Purification by silica gel column chromatography (03:97 EtOAc:hexane) gave the title compound **1a** (206 mg, 74 %) as a white solid.

^{1}H NMR (400 MHz, $CDCl_3$) δ 7.93 – 7.85 (m, 2H), 7.63 – 7.55 (m, 1H), 7.54 – 7.47 (m, 2H), 7.45 – 7.36 (m, 2H), 7.31 – 7.20 (m, 3H), 4.52 – 4.42 (m, 3H), 4.38 – 4.26 (m, 3H); **^{13}C NMR** (101 MHz, $CDCl_3$) δ 198.4, 142.2, 135.0, 133.2, 128.9, 128.7, 128.1, 126.4, 125.0, 64.1, 59.5, 49.0, 47.7; **HRMS (CI$^+$)** m/z: $[M+H]^+$ Calcd. for $C_{21}H_{17}O$ 285.1274; Found 285.1274.

1-(4-Phenylcuban-1-yl)ethan-1-one (1b)

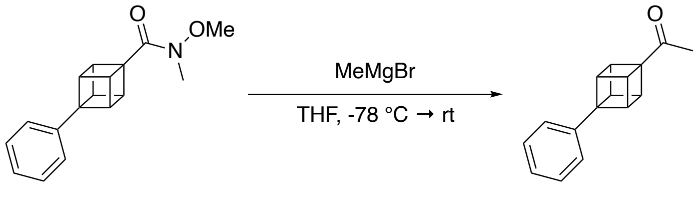

S5 **1b**

To a solution of *N*-methoxy-N-methyl-4-phenylcubane-1-carboxamide (**S5**) (75 mg, 1.0 equiv., 0.28 mmol) in anhydrous THF (3 mL) at -78 °C, was added methylmagnesium bromide (0.4 mL, 2.0 equiv., 1.4 M in toluene/THF) dropwise. The mixture was stirred at -78 °C for 30 minutes before being warmed to rt. After stirring for 30 minutes at rt, the mixture was quenched with 1 M HCl (5 mL) at 0 °C. The aqueous was extracted with EtOAc (3 x 10 mL) and the combined organic layers were washed with sat. brine (10 mL), dried with anhydrous $MgSO_4$, filtered, and concentrated *in vacuo* to afford the crude. Purification by silica gel column chromatography (05:95 EtOAc/petroleum ether) gave the title compound **1b** (60 mg, 96 %) as a white solid.

^{1}H NMR (500 MHz, $CDCl_3$) δ 7.41 – 7.33 (m, 2H), 7.26 – 7.18 (m, 3H), 4.31 – 4.24 (m, 3H), 4.18 – 4.11 (m, 3H), 2.19 (s, 3H); **^{13}C NMR** (126 MHz, $CDCl_3$) δ 206.7, 142.1, 128.6, 126.3, 124.9, 64.5, 60.7, 48.4, 46.3, 24.9; **HRMS (ESI$^+$)** m/z: $[M+H]^+$ Calcd. for $C_{16}H_{15}O$ 223.1117; Found 223.1126.

1-(4-Phenylcuban-1-yl)propan-1-one (1c)

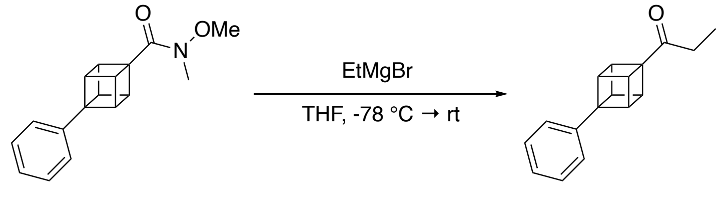

S5 **1c**

To a solution of *N*-methoxy-N-methyl-4-phenylcubane-1-carboxamide (**S5**) (81 mg, 1.0 equiv., 0.3 mmol) in anhydrous THF (3 mL) at -78 °C was added ethylmagnesium bromide (0.67 mL, 2.0 equiv., 0.9 M in THF) dropwise. The mixture was stirred at – 78 °C for 30 minutes and then allowed to warm to rt. After 90 minutes, the mixture was quenched with 1 M HCl (5 mL) at 0 °C. The aqueous was extracted with EtOAc (3 x 10 mL) and the combined organic layers were washed with sat. brine (10 mL), dried with anhydrous $MgSO_4$, filtered, and concentrated *in vacuo* to afford the crude orange oil. Purification by silica gel column

chromatography (05:95 EtOAc/petroleum ether) gave the title compound **1c** (53 mg, 74 %) as a white solid.

^{1}H NMR (500 MHz, $CDCl_3$) δ 7.41 – 7.34 (m, 2H), 7.26 – 7.19 (m, 3H), 4.31 – 4.23 (m, 3H), 4.18 – 4.11 (m, 3H), 2.53 (q, J = 7.4 Hz, 2H), 1.13 (t, J = 7.4 Hz, 3H); **^{13}C NMR** (126 MHz, $CDCl_3$) δ 209.5, 142.1, 128.6, 126.3, 124.8, 63.9, 60.5, 48.5, 46.4, 31.1, 7.7; **HRMS (EI$^+$)** m/z: $[M]^{+\bullet}$ Calcd. for $C_{17}H_{16}O$ 236.1196; Found 236.1194.

(4-Phenylcuban-1-yl)methanol (S4a)

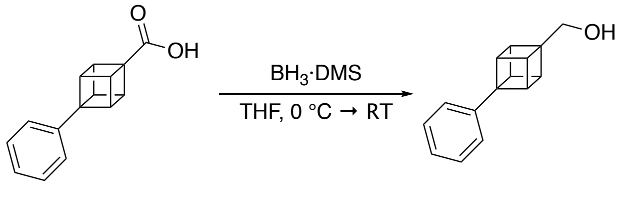

S4 **S4a**

Borane dimethyl sulfide (0.77 mL, 1.6 equiv., 1M in 2-methyltetrahydrofuran) was added dropwise to a solution of 4-phenylcubane-1-carboxylic acid (**S4**) (107 mg, 1.0 equiv., 0.48 mmol) in anhydrous THF (5 mL) at 0 °C. The mixture was stirred at 0 °C for 20 minutes and then allowed to warm to rt. After 5 hours the mixture was quenched with H_2O (5 mL) at 0 °C. The mixture was diluted with EtOAc (10 mL) and the organic layer was washed with sat. $NaHCO_3$ (3 x 10 mL), sat. brine (10 mL), dried with anhydrous $MgSO_4$, filtered, and concentrated *in vacuo* to afford the title compound **S4a** (97 mg, 96%) as a white solid.

^{1}H NMR (400 MHz, $CDCl_3$) δ 7.43 – 7.33 (m, 2H), 7.30 – 7.18 (m, 3H), 4.15 – 4.04 (m, 3H), 3.95 – 3.89 (m, 3H), 3.87 (s, 2H), 1.60 (br s, 1H); **^{13}C NMR** (101 MHz, $CDCl_3$) δ 143.1, 128.5, 125.9, 124.8, 64.0, 60.9, 59.5, 48.1, 43.4; **HRMS (CI$^+$)** m/z: $[M-H_2O]^+$ Calcd. for $C_{15}H_{12}$ 192.0933; Found 192.0941.

4-Phenylcubane-1-carbaldehyde (1d)

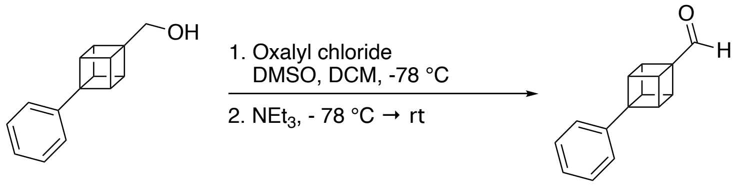

S4a **1d**

DMSO (87 µL, 2.7 equiv., 1.24 mmol) was added to a solution of oxalyl chloride (0.28 mL, 1.2 equiv., 2 M in DCM) in anhydrous DCM (3.5 mL) at -78 °C. After 20 minutes a solution of (4-phenylcuban-1-yl)methanol (**S4a**) (97 mg, 1.0 equiv., 0.46 mmol) in anhydrous DCM (1.5 mL) was added dropwise, and the mixture stirred at -78 °C for a further 1.5 hours. Triethylamine (0.34 mL, 5.4 equiv., 2.47 mmol) was then added and the mixture was allowed to warm to rt over 15 minutes. The mixture was quenched with H_2O (5 mL) and the aqueous layer was extracted with DCM (3 x 10 mL). The combined organic layer was washed sat. brine (10 mL),

dried with anhydrous $MgSO_4$, filtered, and concentrated *in vacuo* to afford the crude. Purification by silica gel column chromatography (05:95 EtOAc/hexane) gave the title compound **1d** (68 mg, 71 %) as a light-yellow oil.

^{1}H NMR (400 MHz, $CDCl_3$) δ 9.82 (s, 1H), 7.43 – 7.33 (m, 2H), 7.26 – 7.18 (m, 3H), 4.44 – 4.34 (m, 3H), 4.23 – 4.13 (m, 3H); **^{13}C NMR** (101 MHz, $CDCl_3$) δ 198.4, 141.9, 128.6, 126.4, 124.9, 63.5, 60.7, 48.9, 45.0; **HRMS (EI$^+$)** m/z: $[M]^{+\bullet}$ Calcd. for $C_{15}H_{12}O$ 208.0883; Found 208.0882.

2-Methyl-1-(4-phenylcuban-1-yl)propan-1-one (1e)

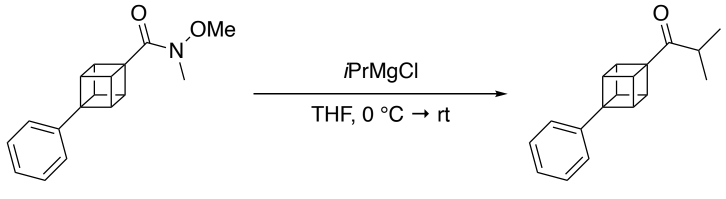

S5 **1e**

To a solution of *N*-methoxy-*N*-methyl-4-phenylcubane-1-carboxamide (**S5**) (250 mg, 1.0 equiv., 0.94 mmol) in anhydrous THF (5 mL) at 0 °C was added isopropylmagnesium chloride (1.4 mL, 3.0 equiv., 2 M in THF) dropwise. The mixture was stirred at 0 °C for 5 minutes and then allowed to warm to rt. After 2.5 hours, the mixture was quenched with 1 M HCl (5 mL) at 0 °C. The aqueous was extracted with EtOAc (3 x 10 mL) and the combined organic layers were washed with sat. brine (10 mL), dried with anhydrous $MgSO_4$, filtered, and concentrated *in vacuo* to afford the crude. Purification by silica gel column chromatography (03:97 EtOAc/petroleum ether) gave the title compound **1e** (179 mg, 77 %) as a white solid.

^{1}H NMR (400 MHz, $CDCl_3$) δ 7.41 – 7.32 (m, 2H), 7.25 – 7.19 (m, 3H), 4.31 – 4.25 (m, 3H), 4.19 – 4.11 (m, 3H), 2.85 (hept, *J* = 6.9 Hz, 1H), 1.15 (d, *J* = 6.9 Hz, 6H); **^{13}C NMR** (101 MHz, $CDCl_3$) δ 212.4, 142.2, 128.6, 126.3, 124.9, 63.9, 60.2, 48.5, 46.8, 37.7, 18.1; **HRMS (ESI$^+$)** m/z: $[M+H]^+$ Calcd. for $C_{18}H_{19}O$ 251.1431; Found 251.1443.

2,2-Dimethyl-1-(4-phenylcuban-1-yl)propan-1-one (1f)

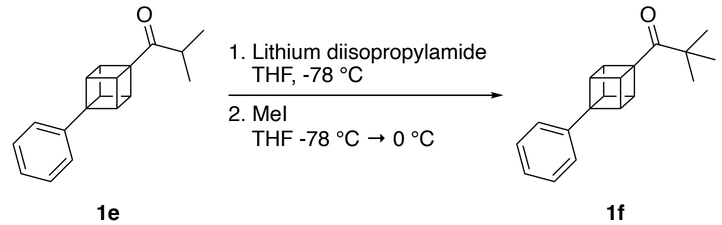

1e **1f**

To a solution of diisopropylamine (105 μL, 1.5 equiv., 0.75 mmol) in anhydrous THF (5 mL) was added *n*-BuLi (0.44 mL, 1.4 equiv., 1.6 M in hexanes) dropwise at -78 °C. After 10 minutes, a solution of 2-methyl-1-(4-phenylcuban-1-yl)propan-1-one (**1e**) (126 mg, 1 equiv., 0.5 mmol) in anhydrous THF (1 mL) was added dropwise to the freshly prepared lithium diisopropylamide solution. The mixture was stirred at -78 °C for a further 30 minutes, then methyl iodide (47 μL, 1.5 equiv., 0.75 mmol) was added dropwise. The reaction was allowed to stir for 10 minutes before being allowed to warm to 0 °C. After 30 minutes at 0 °C the

mixture was quenched with H_2O (5 mL) and diluted with EtOAc (15 mL). The organic layer was washed with 1 M NaOH (1 x 15 mL), sat. brine (10 mL), dried with anhydrous $MgSO_4$, filtered, and concentrated *in vacuo* to afford the crude. Purification by silica gel column chromatography (02:98 EtOAc/petroleum ether) gave the title compound **1f** (114 mg, 86 %) as a white solid.

^{1}H NMR (400 MHz, $CDCl_3$) δ 7.42 – 7.32 (m, 2H), 7.25 – 7.19 (m, 3H), 4.39 – 4.30 (m, 3H), 4.21 – 4.12 (m, 3H), 1.23 (s, 9H); **^{13}C NMR** (101 MHz, $CDCl_3$) δ 213.1, 142.2, 128.6, 126.2, 124.9, 64.4, 59.5, 48.4, 47.7, 44.5, 26.5; **HRMS (ESI$^+$)** m/z: $[M+H]^+$ Calcd. for $C_{19}H_{21}O$ 265.1587; Found 265.1591.

Synthesis of 1g:

Methyl-4-iodocubane-1-carboxylate (S6)

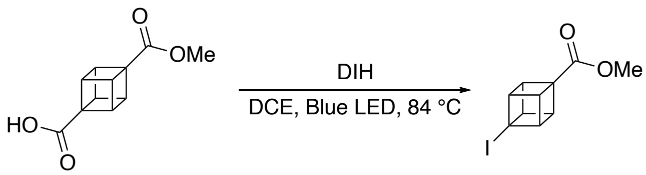

S1 **S6**

Following an adapted procedure of Kulbitski and co-workers.[4] A solution of the commercially available 4-(methoxycarbonyl)cubane-1-carboxylic acid (**S1**) (1.0 g, 1.0 equiv., 4.85 mmol) and 1,3-diiodo-5,5-dimethylhydantoin (DIH) (2.2 g, 1.2, equiv., 5.82 mmol) in anhydrous DCE (30 mL) was heated at 84 °C under blue light irradiation for 5 h (Aldrich® micro photochemical reactor blue LED (ALDKIT001)). The mixture was cooled to rt and quenched with 1 M sodium thiosulfate (15 mL) and diluted with DCM (15 mL). The organic layer was washed with 1 M sodium thiosulfate (2 x 15 mL), dried with anhydrous $MgSO_4$, filtered, and concentrated *in vacuo* to afford the crude. Purification by silica gel column chromatography (03:97 EtOAc: petroleum ether) gave the title compound **S6** (1.2 g, 85 %) as a white solid.

^{1}H NMR (400 MHz, $CDCl_3$) δ 4.42 – 4.34 (m, 3H), 4.33 – 4.22 (m, 3H), 3.70 (s, 3H). **^{13}C NMR** (101 MHz, $CDCl_3$) δ 172.0, 56.2, 55.0, 51.8, 50.4, 36.3; **HRMS (EI$^+$)** m/z: $[M-H]^+$ Calcd. for $C_{10}H_8O_2{}^{127}I$ 286.9564; Found 286.9561. All spectroscopic data were in accordance with the literature.[4]

4-Iodocubane-1-carboxylic acid (S7)

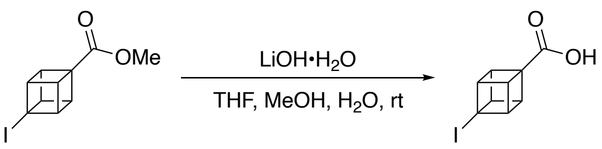

S6 **S7**

To a solution of methyl-4-iodocubane-1-carboxylate (**S6**) (1.2 g, 1.0 equiv., 4.17 mmol) in 3:3:1 ratio of THF:MeOH:H_2O (10 mL) was added lithium hydroxide monohydrate (437 mg,

2.5 equiv., 10.4 mmol) in one-portion at rt. The mixture was allowed to stir at rt overnight. The mixture was diluted with 1 M NaOH (2 mL) and the aqueous layer was washed with EtOAc (2 x 10 mL). The aqueous layer was acidified with 2 M HCl, and the aqueous layer was extracted with EtOAc (3 x 15 mL). The combined organic layers were washed sat. brine (15 mL), dried with anhydrous $MgSO_4$, filtered, and concentrated *in vacuo* to afford the title compound **S7** (1.1 g, 92 %) as a cream solid.

^{1}H-NMR (300 MHz, DMSO-d_6): δ 12.52 (br, 1H), 4.34-4.25 (m, 6H); **HRMS (ESI$^-$)** m/z: $[M-H]^-$ Calcd. for $C_9H_6{}^{127}IO_2$ 272.9418; Found 272.9407. All spectroscopic data were in accordance with the literature.[4]

4-Iodo-*N*,*N*-diisopropylcubane-1-carboxamide (S8)

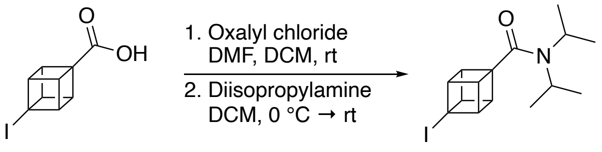

S7 **S8**

To a solution of 4-iodocubane-1-carboxylic acid (**S7**) (765 mg, 1.0 equiv., 2.79 mmol) in anhydrous DCM (15mL) was added oxalyl chloride (2.37 mL, 1.7 equiv., 2 M in DCM) at rt. Anhydrous DMF (22 μL, 10 mol%) was then added and the reaction mixture was allowed to stir at rt for 1.5 h. The mixture was concentrated *in vacuo* to afford the acid chloride as a yellow solid.

The acid chloride was dissolved in anhydrous DCM (7.5 mL) and added dropwise to a solution of diisopropylamine (0.98 mL, 2.5 equiv., 6.98 mmol) in anhydrous DCM (7.5 mL) at 0 °C. The mixture was warmed to rt and stirred for 3.5 h. The mixture was quenched with sat. NH_4Cl (5 mL) and diluted with DCM (15 mL). The organic layer was washed with 1 M HCl (10 mL), sat. brine (10 mL), dried with anhydrous $MgSO_4$, filtered and concentrated *in vacuo* to afford the crude. Purification by silica gel column chromatography (1:5 EtOAc: petroleum ether) gave the title compound **S8** (868 mg, 87 %) as a white solid.

^{1}H NMR (400 MHz, $CDCl_3$) δ 4.35 – 4.28 (m, 3H), 4.28 – 4.21 (m, 3H), 3.35 (hept, *J* = 6.7 Hz, 1H), 3.29 (hept, *J* = 6.8 Hz, 1H), 1.39 (d, *J* = 6.8 Hz, 6H), 1.18 (d, *J* = 6.7 Hz, 6H); **^{13}C NMR** (101 MHz, $CDCl_3$) δ 169.9, 59.9, 54.3, 50.3, 48.5, 46.1, 36.2, 21.1, 20.6; **HRMS (EI$^+$)** m/z: $[M-H]^+$ Calcd. for $C_{15}H_{19}ON^{127}I$ 356.0506; Found 356.0493. All spectroscopic data were in accordance with the literature.[5]

4-Iodo-*N*,*N*-diisopropylcubane-1-carboxamide (S9)

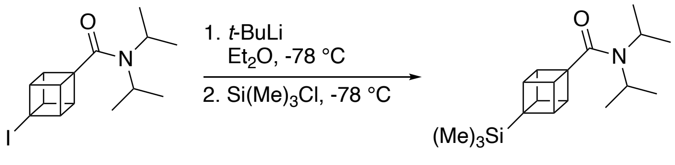

S8 **S9**

Following an adapted procedure of Lukin and Eaton.[6] To a solution of 4-iodo-*N*,*N*-diisopropylcubane-1-carboxamide (**S8**) (868 mg, 1.0 equiv., 2.43 mmol) in anhydrous diethyl ether (20 mL) was added *tert*-butyl lithium (2.74 mL, 2.15 equiv., 1.9 M in pentanes) dropwise over 5 minutes at -78 °C. After 20 minutes, chlorotrimethylsilane (1.17 mL, 3.8 equiv., 9.22 mmol) was added dropwise and the mixture was stirred for a further 1.5 hours at -78 °C. The mixture was quenched with methanol (1 mL) and then allowed to warm to 0 °C. After, H_2O (5 mL) was added dropwise to the mixture and allowed to warm to rt. The aqueous layer was extracted with Et_2O (3 x 15 mL) and the combined organic layers were washed with sat. brine (10 mL), dried with anhydrous $MgSO_4$, filtered and concentrated *in vacuo* to afford the crude. Purification by silica gel column chromatography (7:93 EtOAc: petroleum ether) gave the title compound **S9** (660 mg, 94 %) as a white solid.

^{1}H NMR (400 MHz, $CDCl_3$) δ 4.23 – 4.13 (m, 3H), 3.84 – 3.75 (m, 3H), 3.52 (hept, *J* = 6.6 Hz, 1H), 3.28 (hept, *J* = 6.8 Hz, 1H), 1.41 (d, *J* = 6.8 Hz, 6H), 1.17 (d, *J* = 6.6 Hz, 6H), -0.06 (s, 9H); **^{13}C NMR** (101 MHz, $CDCl_3$) δ 171.2, 59.6, 49.7, 48.3, 47.4, 45.9, 43.1, 21.1, 20.7, -4.7; **HRMS (ESI$^+$)** m/z: $[M+H]^+$ Calcd. for $C_{18}H_{30}NOSi$ 304.2091; Found 304.2089. All spectroscopic data were in accordance with the literature.[7]

***N*-Isopropyl-*N*-(4-(trimethylsilyl)cuban-1-yl)methyl)propan-2-amine (S10)**

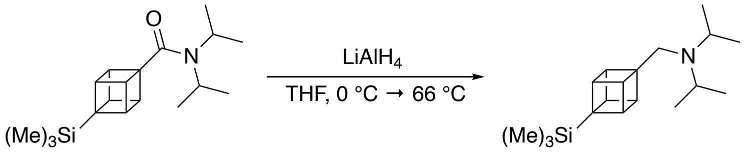

S9 **S10**

Following an adapted procedure of Lukin and Eaton.[6] To a solution of lithium aluminum hydride (124 mg, 1.5 equiv., 3.26 mmol) in anhydrous THF (15 mL) was added 4-iodo-*N*,*N*-diisopropylcubane-1-carboxamide (**S9**) (660 mg, 1.0 equiv., 2.17 mmol) in anhydrous THF (2 mL) dropwise at 0 °C. After the addition the mixture was heated to 66 °C for 1 h and then cooled to 0 °C. The mixture was quenched with H_2O (1 mL), 1 M NaOH (1 mL) and H_2O (2 mL) dropwise. The aqueous layer was extracted with Et_2O (15 mL x 3) and the combined organic layers were washed with sat. brine (10 mL), dried with anhydrous $MgSO_4$, filtered and concentrated *in vacuo* to afford the title compound **S10** (609 mg, 97 %) as a light-yellow oil. **S10** was used in the next step without further purification.

^{1}H NMR (400 MHz, $CDCl_3$) δ 3.83 – 3.76 (m, 3H), 3.75 – 3.66 (m, 3H), 2.93 (hept, *J* = 6.6 Hz, 2H), 2.63 (s, 2H), 0.97 (d, *J* = 6.6 Hz, 12H), -0.06 (s, 9H); **^{13}C NMR** (101 MHz, $CDCl_3$) δ 59.4, 48.8, 48.7, 47.9, 46.8, 43.2, 21.0, -4.7; **HRMS (ESI$^+$)** m/z: $[M+H]^+$ Calcd. for $C_{18}H_{32}NSi$ 290.2299; Found 290.2302.

4-(Trimethylsilyl)cubane-1-carboxylic acid (S11)

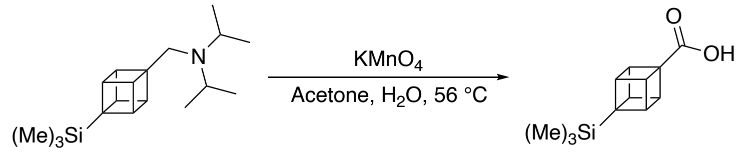

S10 **S11**

Following an adapted procedure of Lukin and Eaton.[6] To a solution of *N*-isopropyl-*N*-(4-(trimethylsilyl)cuban-1-yl)methyl)propan-2-amine (**S10**) (609 mg, 1.0 equiv., 2.1 mmol) in acetone (18 mL) at 56 °C was added 32 mL of a solution of $KMnO_4$ (1.5 g, 4.5 equiv., 9.26 mmol) dissolved in acetone (42 mL) and H_2O (8.4 mL), resulting in a deep purple colour. After 5 minutes, the mixture was cooled to rt and quenched with the addition of solid sodium bisulfite till a grey solid precipitate formed. The mixture was filtered through a pad of celite and the solid was washed with acetone (50 mL). The filtrate was concentrated *in vacuo* and the residue was diluted with 1 M NaOH (5 mL) and the aqueous layer was washed with EtOAc (2 x 10 mL). The aqueous layer was acidified with 2 M HCl, and the aqueous layer was extracted with EtOAc (3 x 15 mL). The combined organic layers were washed sat. brine (15 mL), dried with anhydrous $MgSO_4$, filtered, and concentrated *in vacuo* to afford the title compound **S11** (406 mg, 88 %) as a white solid

^{1}H NMR (500 MHz, $CDCl_3$) δ 4.34 – 4.23 (m, 3H), 3.92 – 3.79 (m, 3H), -0.04 (s, 9H); **^{13}C NMR** (126 MHz, $CDCl_3$) δ 178.3, 55.8, 49.9, 49.1, 44.2, -4.7; **HRMS (ESI$^+$)** m/z: $[M+H]^+$ Calcd. for $C_{12}H_{17}O_2Si$ 221.0992; Found 221.0992.

***N*-Methoxy-*N*-methyl-4-(trimethylsilyl)cubane-1-carboxamide (S12)**

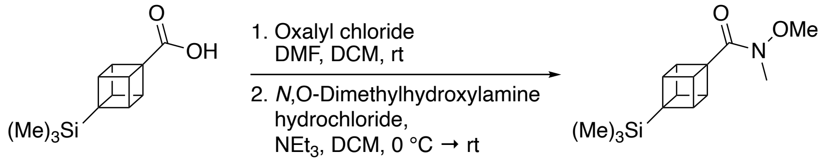

S11 **S12**

To a solution of 4-(trimethylsilyl)cubane-1-carboxylic acid (**S11**) (236 mg, 1.0 equiv., 1.07 mmol) in anhydrous DCM (5 mL) was added oxalyl chloride (0.91 mL, 1.7 equiv., 2 M in DCM) at rt. Anhydrous DMF (8 μL, 10 mol%) was then added and the reaction mixture was allowed to stir at rt for 1.5 h. The mixture was concentrated *in vacuo* to afford the acid chloride as a yellow solid.

The acid chloride was dissolved in anhydrous DCM (2.5 mL) and added dropwise to a solution of *N*,O-dimethylhydroxylamine hydrochloride (167 mg, 1.6 equiv., 1.71 mmol) and triethylamine (0.47 mL, 3.2 equiv., 3.39 mmol) in anhydrous DCM (2.5 mL) at 0 °C. The mixture was warmed to rt and stirred for 1 h. The mixture was quenched with sat. NH_4Cl (5 mL) and diluted with DCM (10 mL). The organic layer was washed with 1 M HCl (10 mL), sat. brine (10 mL), dried with anhydrous $MgSO_4$, and concentrated *in vacuo* to afford the crude. Purification by silica gel column chromatography (1:4 EtOAc/petroleum ether) gave the title compound **S12** (167 mg, 60 %) as a light yellow solid.

^{1}H NMR (500 MHz, $CDCl_3$) δ 4.31 – 4.23 (m, 3H), 3.88 – 3.79 (m, 3H), 3.69 (s, 3H), 3.17 (s, 3H), -0.05 (s, 9H); **^{13}C NMR** (126 MHz, $CDCl_3$) δ 173.8, 61.7, 57.9, 50.0, 48.2, 44.0, 32.8, -4.7; **HRMS (ESI$^+$)** m/z: $[M+H]^+$ Calcd. for $C_{14}H_{22}NO_2Si$ 264.1414; Found 264.1410.

2-Methyl-1-(4-(trimethylsilyl)cuban-1-yl)propan-1-one (1g)

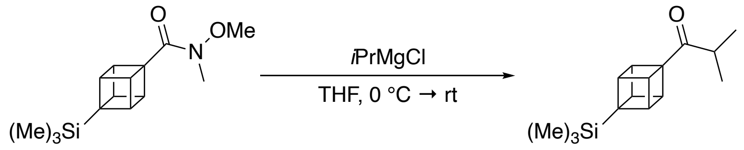

S12 **1g**

To a solution of *N*-methoxy-*N*-methyl-4-(trimethylsilyl)cubane-1-carboxamide (**S12**) (141 mg, 1.0 equiv., 0.54 mmol) in anhydrous THF (3 mL) at 0 °C was added isopropylmagnesium chloride (0.80 mL, 3.0 equiv., 2 M in THF) dropwise. The mixture was stirred at 0 °C for 5 minutes and allowed to warm to rt. After 3.5 hours, the mixture was quenched with 1 M HCl (5 mL) at 0 °C. The aqueous was extracted with EtOAc (3 x 10 mL) and the combined organic layers were washed with sat. brine (10 mL), dried with anhydrous $MgSO_4$, filtered, and concentrated *in vacuo* to afford the crude orange oil. Purification by silica gel column chromatography (02:98 EtOAc: petroleum ether) gave the title compound **1g** (99 mg, 75 %) as a colourless oil.

^{1}H NMR (400 MHz, $CDCl_3$) δ 4.34 – 4.24 (m, 3H), 3.88 – 3.78 (m, 3H), 2.78 (hept, *J* = 6.9 Hz, 1H), 1.09 (d, *J* = 6.9 Hz, 6H), -0.05 (s, 9H); **^{13}C NMR** (101 MHz, $CDCl_3$) δ 212.2, 63.4, 50.5, 48.9, 43.8, 37.6, 18.1, -4.7; **HRMS (ESI$^+$)** m/z: $[M+H]^+$ Calcd. for $C_{15}H_{23}OSi$ 247.1513; Found 247.1515.

Synthesis of 1h:

Methyl-cubane-1-carboxylate (S13)

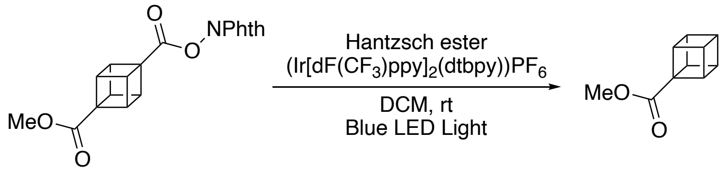

S2 **S13**

1-(1,3-Dioxoisoindolin-2-yl) 4-methyl-cubane-1,4-dicarboxylate (**S2**) (500 mg, 1.0 equiv., 1.42 mmol), hantzsch ester (540 mg, 1.5 equiv., 0.09 mmol) and $(Ir[dF(CF_3)ppy]_2(dtbpy))PF_6$ (2.2 mg, 0.13 mol %) were combined in anhydrous DCM (15 mL). The mixture was irradiated with blue LED light at rt for 5 h (Aldrich® micro photochemical reactor blue LED (ALDKIT001) was used as the blue LED light source). The reaction mixture was concentrated *in vacuo* and purification of the crude yellow solid by silica gel column chromatography (2:98 EtOAc/hexane) gave the title compound **S13** (162 mg, 70 %) as a white solid.

^{1}H NMR (400 MHz, $CDCl_3$) δ 4.30 – 4.20 (m, 3H), 4.06 – 3.94 (m, 4H), 3.70 (s, 3H); **^{13}C NMR** (101 MHz, $CDCl_3$) δ 173.0, 55.8, 51.6, 49.6, 48.0, 45.3; **HRMS (ESI$^+$)** m/z: $[M+H]^+$ Calcd. for

$C_{10}H_{11}O_2$ 163.0754; Found 163.0759. All spectroscopic data were in accordance with the literature.[8]

Cubane-1-carboxylic acid (S14)

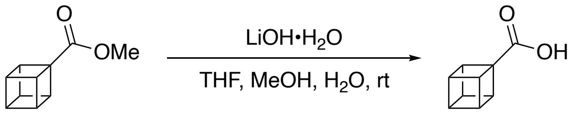

To a solution of methyl-cubane-1-carboxylate (**S13**) (411 mg, 1.0 equiv., 2.53 mmol) in 3:3:1 ratio of THF:MeOH:H_2O (7.9 mL) was added lithium hydroxide monohydrate (266 mg, 2.5 equiv., 6.34 mmol) in one-portion at rt. The mixture was allowed to stir at rt overnight. The mixture was acidified with 2 M HCl, and the aqueous layer was extracted with EtOAc (3 x 15 mL). The combined organic layers were washed sat. brine (15 mL), dried with anhydrous $MgSO_4$, filtered, and concentrated *in vacuo* to afford the crude. The solid was washed with hexane (3 x 5 mL) to afford the title compound **S14** (305 mg, 81%) as a white solid.

^{1}H NMR (400 MHz, $CDCl_3$) δ 11.69 (br s, 1H), 4.37 – 4.25 (m, 3H), 4.11 – 3.94 (m, 4H); **^{13}C NMR** (101 MHz, $CDCl_3$) δ 178.9, 55.6, 49.6, 48.0, 45.3; **HRMS (Cl^-)** m/z: $[M-H]^-$ Calcd. for $C_9H_7O_2$ 147.0451; Found 147.0441. All spectroscopic data were in accordance with the literature.[9]

***N*-Methoxy-*N*-methylcubane-1-carboxamide (S15)**

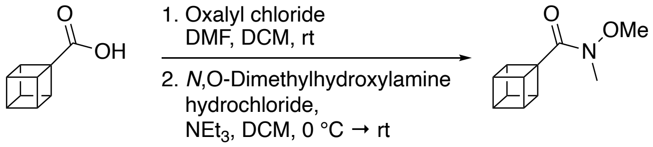

To a solution of cubane-1-carboxylic acid (**S14**) (237 mg, 1.0 equiv., 1.60 mmol) in anhydrous DCM (5 mL) was added oxalyl chloride (0.96 mL, 1.2 equiv., 2 M in DCM) dropwise at rt. Anhydrous DMF (12 μL, 10 mol%) was then added and the reaction mixture was allowed to stir at rt for 1.5 h. The mixture was concentrated *in vacuo* to afford the acid chloride as a yellow solid.

The acid chloride was dissolved in anhydrous DCM (2 mL) and added dropwise to a solution of *N*,O-dimethylhydroxylamine hydrochloride (250 mg, 1.6 equiv., 2.56 mmol) and triethylamine (0.71 mL, 3.2 equiv., 2.07 mmol) in anhydrous DCM (2 mL) at 0 °C. The mixture was warmed to rt and stirred for 3 h. The mixture was quenched with sat. NH_4Cl (5 mL) and diluted with DCM (10 mL). The organic layer was washed with 1 M HCl (10 mL), sat. brine (10 mL), dried with anhydrous $MgSO_4$, and concentrated *in vacuo* to afford the crude. Purification by silica gel column chromatography (1:3 EtOAc/petroleum ether) gave the title compound **S15** (167 mg, 55 %) as a white solid.

^{1}**H NMR** (500 MHz, $CDCl_3$) δ 4.28 – 4.20 (m, 3H), 4.00 – 3.91 (m, 4H), 3.68 (s, 3H), 3.15 (s, 3H); ^{13}**C NMR** (126 MHz, $CDCl_3$) δ 173.9, 61.6, 57.5, 49.7, 47.4, 45.1, 32.8; **HRMS (CI⁺)** m/z: $[M+H]^+$ Calcd. for $C_{11}H_{14}O_2N$ 192.1019; Found 192.1017. All spectroscopic data were in accordance with the literature.[10]

1-(Cuban-1-yl)-2-methylpropan-1-one (1h)

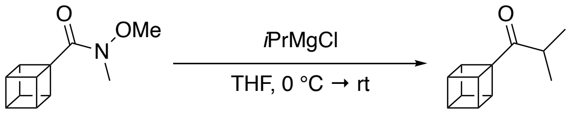

S15 **1h**

To a solution of *N*-methoxy-*N*-methylcubane-1-carboxamide (**S15**) (146 mg, 1.0 equiv., 0.76 mmol) in anhydrous THF (5 mL) at 0 ° was added isopropylmagnesium chloride (1.15 mL, 3.0 equiv., 2 M in THF) dropwise. The mixture was stirred at 0 °C for 5 minutes and allowed to warm to rt. After 3.5 hours, the mixture was quenched with 1 M HCl (5 mL) at 0 °C. The aqueous was extracted with EtOAc (3 x 10 mL) and the combined organic layers were washed with sat. brine (10 mL), dried with anhydrous $MgSO_4$, filtered, and concentrated *in vacuo* to afford the crude. Purification by silica gel column chromatography (02:98 EtOAc: petroleum ether) gave the title compound **1h** (89 mg, 67 %) as a white solid.

^{1}**H NMR** (400 MHz, $CDCl_3$) δ 4.33 – 4.22 (m, 3H), 4.05 – 3.92 (m, 4H), 2.77 (hept, *J* = 6.9 Hz, 1H), 1.08 (d, *J* = 6.9 Hz, 6H); ^{13}**C NMR** (101 MHz, $CDCl_3$) δ 212.5, 63.1, 50.2, 47.9, 44.9, 37.6, 18.1; **HRMS (EI⁺)** m/z: $[M]^{+\bullet}$ Calcd. for $C_{12}H_{14}O$ 174.1039; Found 174.1040.

Synthesis of 1i:

Methyl-4-(hydroxymethyl)cubane-1-carboxylate (S16)

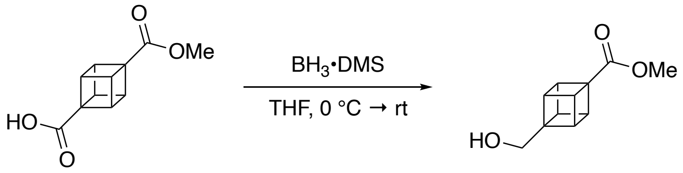

S1 **S16**

To a solution of the commercially available 4-(methoxycarbonyl)cubane-1-carboxylic acid (**S1**) (500 mg, 1.0 equiv., 2.42 mmol) in anhydrous THF (10 mL) was added borane dimethyl sulfide (4.12 mL, 1.7 equiv., 1 M in 2-methyltetrahydrofuran) dropwise at 0 °C. After the addition the mixture was stirred at rt for 4.5 h. The mixture was quenched with H_2O (2 mL) at 0 °C and diluted with EtOAc (20 mL). The organic layer was washed with sat. $NaHCO_3$ (2 x 10 mL), sat. brine (20 mL), dried with anhydrous $MgSO_4$, filtered, and concentrated *in vacuo* to afford the title compound **S16** (449 mg, 96 %) as a white solid.

^{1}**H NMR** (400 MHz, $CDCl_3$) δ 4.18 – 4.11 (m, 3H), 3.92 – 3.86 (m, 3H), 3.77 (s, 2H), 3.70 (s, 3H); **HRMS (EI⁺)** m/z: $[M-H_2O]^+$ Calcd. for $C_{11}H_{10}O_2$ 174.0675; Found 174.0674. All spectroscopic data were in accordance with the literature.[11]

Methyl-4-(methoxymethyl)cubane-1-carboxylate (S17)

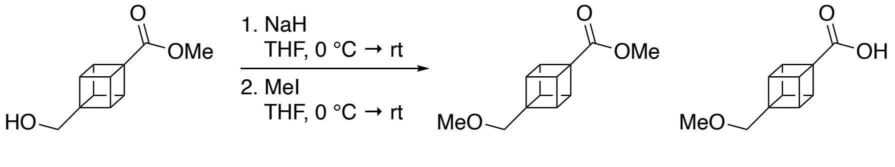

To a solution of sodium hydride (60% dispersion in mineral oil) (161 mg, 2.0 equiv., 3.98 mmol) in anhydrous THF (10 mL) was added methyl-4-(hydroxymethyl)cubane-1-carboxylate (**S16**) (383 mg, 1.0 equiv., 1.99 mmol) in anhydrous THF (5 mL) dropwise at 0 °C and then stirred at rt for 15 minutes. The mixture was re-cooled to 0 °C and iodomethane (0.37 mL, 3.0 equiv., 3.98 mmol) was added dropwise, after 10 minutes the mixture was warmed to rt. After 6 hours the mixture was cooled to 0 °C , quenched with H_2O (5 mL) and allowed to stir at rt for 15 minutes. The basic aqueous layer* was washed with EtOAc (2 x 15 mL) and the combined organic layers were washed with sat. brine (20 mL), dried with anhydrous $MgSO_4$, filtered, and concentrated *in vacuo* to afford the crude containing **S17**. Purification by silica gel column chromatography (1:9 EtOAc/petroleum ether) gave the title compound **S17** (66 mg, 16 %) as a white solid.

^{1}H NMR (400 MHz, $CDCl_3$) δ 4.19 – 4.09 (m, 3H), 3.92 – 3.81 (m, 3H), 3.69 (s, 3H), 3.52 (s, 2H), 3.36 (s, 3H); **^{13}C NMR** (101 MHz, $CDCl_3$) δ 172.9, 73.2, 59.4, 57.6, 56.3, 51.6, 46.7, 45.2; **HRMS (EI$^+$)** m/z: $[M-H]^+$ Calcd. for $C_{12}H_{13}O_3$ 205.0859; Found 205.0857.

*To isolate **S18**, the basic aqueous layer was acidified with 2 M HCl, and the aqueous layer was extracted with EtOAc (3 x 15 mL). The combined organic layers were washed sat. brine (15 mL), dried with anhydrous $MgSO_4$, filtered, and concentrated *in vacuo* to afford the title compound **S18** (101 mg, 26 %) as a white solid.

^{1}H NMR (400 MHz, $CDCl_3$) δ 4.23 – 4.16 (m, 3H), 3.93 – 3.86 (m, 3H), 3.54 (s, 2H), 3.38 (s, 3H); **^{13}C NMR** (101 MHz, $CDCl_3$) δ 178.4, 73.1, 59.4, 57.7, 56.1, 46.7, 45.3; **HRMS (ESI$^-$)** m/z: $[M-H]^-$ Calcd. for $C_{11}H_{11}O_3$ 191.0713; Found 191.0710.

4-(Methoxymethyl)cubane-1-carboxylic acid (S18)

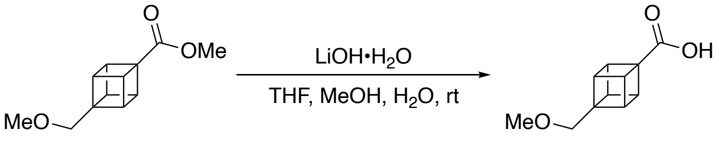

To a solution of Methyl-4-(methoxymethyl)cubane-1-carboxylate (**S17**) (66 mg, 1.0 equiv., 0.32 mmol) in 3:3:1 ratio of THF:MeOH:H_2O (1 mL) was added lithium hydroxide monohydrate (34 mg, 2.5 equiv., 0.8 mmol) in one-portion at rt. The mixture was allowed to stir at rt overnight. The mixture was diluted with 1 M NaOH (2 mL) and the aqueous layer was washed with EtOAc (2 x 10 mL). The aqueous layer was acidified with 2 M HCl, and the aqueous layer was extracted with EtOAc (3 x 10 mL). The combined organic layers were washed sat. brine (10 mL), dried with anhydrous $MgSO_4$, filtered, and concentrated *in vacuo*

to afford the title compound **S18** (36 mg, 59 %) as a cream solid. **S18** was used directly in the next step without further purification.

^{1}H NMR (400 MHz, $CDCl_3$) δ 4.23 – 4.16 (m, 3H), 3.93 – 3.86 (m, 3H), 3.54 (s, 2H), 3.38 (s, 3H); **^{13}C NMR** (101 MHz, $CDCl_3$) δ 178.4, 73.1, 59.4, 57.7, 56.1, 46.7, 45.3; **HRMS (ESI$^-$)** m/z: $[M-H]^-$ Calcd. for $C_{11}H_{11}O_3$ 191.0713; Found 191.0710.

***N*-Methoxy-4-(methoxymethyl)-*N*-methylcubane-1-carboxamide (S19)**

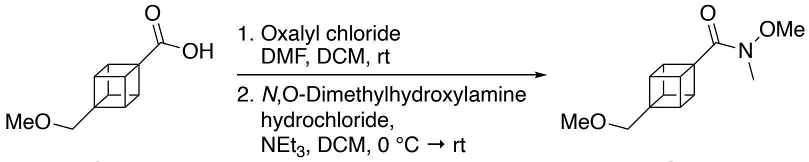

S18 **S19**

To a solution of 4-(methoxymethyl)cubane-1-carboxylic acid (**S18**) (137 mg, 1.0 equiv., 0.71 mmol) in anhydrous DCM (3 mL) was added oxalyl chloride (0.61 mL, 1.7 equiv., 2 M in DCM) at rt. Anhydrous DMF (6 µL, 10 mol%) was then added and the reaction mixture was allowed to stir at rt for 1.5 h. The mixture was concentrated *in vacuo* to afford the acid chloride as a yellow solid.

The acid chloride was dissolved in anhydrous DCM (1.5 mL) and added dropwise to a solution of *N*,O-dimethylhydroxylamine hydrochloride (117 mg, 1.7 equiv., 1.20 mmol) and triethylamine (0.33 mL, 3.4 equiv., 2.41 mmol) in anhydrous DCM (1.5 mL) at 0 °C. The mixture was warmed to rt and stirred for 2 h. The mixture was quenched with sat. NH_4Cl (5 mL) and diluted with DCM (10 mL). The organic layer was washed with 1 M HCl (10 mL), sat. brine (10 mL), dried with anhydrous $MgSO_4$, and concentrated *in vacuo* to afford the crude. Purification by silica gel column chromatography (1:1 EtOAc/petroleum ether) gave the title compound **S19** (93 mg, 55 %) as a white solid.

^{1}H NMR (400 MHz, $CDCl_3$) δ 4.19 – 4.08 (m, 3H), 3.87 – 3.77 (m, 3H), 3.66 (s, 3H), 3.50 (s, 2H), 3.34 (s, 3H), 3.14 (s, 3H); **^{13}C NMR** (101 MHz, $CDCl_3$) δ 173.8, 73.2, 61.6, 59.3, 58.1, 57.0, 46.7, 45.1, 32.7; **HRMS (EI$^+$)** m/z: $[M-OMe]^+$ Calcd. for $C_{12}H_{14}O_2N$ 204.1019; Found 204.1016.

1-(4-(Methoxymethyl)cuban-1-yl)-2-methylpropan-1-one (1i)

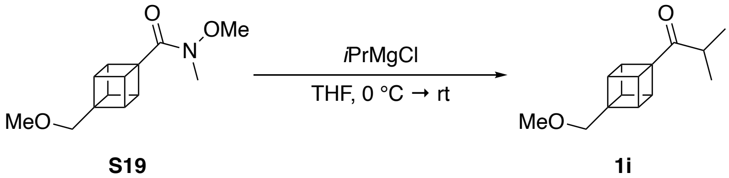

S19 **1i**

To a solution of *N*-methoxy-4-(methoxymethyl)-*N*-methylcubane-1-carboxamide (**S19**) (93 mg, 1.0 equiv., 0.40 mmol) in anhydrous THF (2.5 mL) at 0 °C was added isopropylmagnesium chloride (0.6 mL, 3.0 equiv., 2 M in THF) dropwise. The mixture was stirred at 0 °C for 5 minutes and allowed to warm to rt. After 2.5 hours, the mixture was quenched with 1 M HCl (5 mL) at 0 °C. The aqueous was extracted with EtOAc (3 x 10 mL) and the combined organic layers were washed with sat. brine (10 mL), dried with anhydrous

$MgSO_4$, filtered, and concentrated *in vacuo* to afford the crude. Purification by silica gel column chromatography (15:85 EtOAc: petroleum ether) gave the title compound **1i** (64 mg, 74 %) as a colourless oil.

^{1}H NMR (400 MHz, $CDCl_3$) δ 4.23 – 4.14 (m, 3H), 3.91 – 3.82 (m, 3H), 3.54 (s, 2H), 3.38 (s, 3H), 2.79 (hept, J = 6.9 Hz, 1H), 1.09 (d, J = 6.9 Hz, 6H); **^{13}C NMR** (101 MHz, $CDCl_3$) δ 212.5, 73.2, 63.7, 59.4, 57.6, 47.4, 44.9, 37.6, 18.1; **HRMS (EI$^+$)** m/z: $[M]^{+\bullet}$ Calcd. for $C_{14}H_{18}O_2$ 218.1301; Found 218.1296.

Synthesis of 1j:

Methyl-4-(diisopropylcarbamoyl)cubane-1-carboxylate (S20)

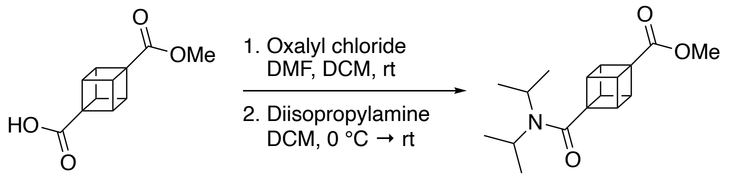

S1 **S20**

To a solution of the commercially available 4-(methoxycarbonyl)cubane-1-carboxylic acid (**S1**) (300 mg, 1.0 equiv., 1.45 mmol) in anhydrous DCM (5mL) was added oxalyl chloride (1.24 mL, 1.7 equiv., 2 M in DCM) at rt. Anhydrous DMF (12 μL, 10 mol%) was then added and the reaction mixture was allowed to stir at rt for 1.5 h. The mixture was concentrated *in vacuo* to afford the acid chloride as a yellow solid.

The acid chloride was dissolved in anhydrous DCM (2.5 mL) and added dropwise to a solution of diisopropylamine (0.51 mL, 2.5 equiv., 3.63 mmol) in anhydrous DCM (2.5 mL) at 0 °C. The mixture was warmed to rt and stirred for 4.5 h. The mixture was quenched with sat. NH_4Cl (5 mL) and diluted with DCM (10 mL). The organic layer was washed with 1 M HCl (10 mL), sat. brine (10 mL), dried with anhydrous $MgSO_4$, filtered and concentrated *in vacuo* to afford the title compound **S20** (401 mg, 95 %) as a yellow solid. **S20** was used directly in the next step without further purification.

^{1}H NMR (400 MHz, $CDCl_3$) δ 4.23 – 4.14 (m, 6H), 3.70 (s, 3H), 3.46 (hept, J = 6.6 Hz, 1H), 3.30 (hept, J = 6.8 Hz, 1H), 1.41 (d, J = 6.8 Hz, 6H), 1.20 (d, J = 6.6 Hz, 6H); **HRMS (EI$^+$)** m/z: $[M]^{+\bullet}$ Calcd. for $C_{17}H_{23}O_3N$ 289.1673; Found 289.1665. All spectroscopic data were in accordance with the literature.[12]

4-(Diisopropylcarbamoyl)cubane-1-carboxylic acid (S21)

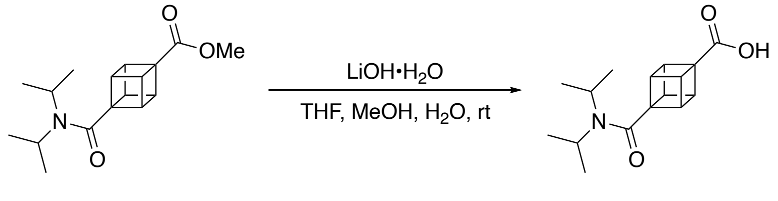

To a solution of methyl-4-(diisopropylcarbamoyl)cubane-1-carboxylate (**S20**) (401 mg, 1.0 equiv., 1.39 mmol) in 3:3:1 ratio of THF:MeOH:H_2O (4.1 mL) was added lithium hydroxide monohydrate (69 mg, 1.2 equiv., 1.64 mmol) in one-portion at rt. The mixture was allowed to stir at rt for 4 h. The mixture was diluted with 1 M NaOH (2 mL) and the aqueous layer was washed with EtOAc (2 x 10 mL). The aqueous layer was acidified with 2 M HCl, and the aqueous layer was extracted with EtOAc (3 x 15 mL). The combined organic layers were washed sat. brine (15 mL), dried with anhydrous $MgSO_4$, filtered, and concentrated *in vacuo* to afford the title compound **S21** (335 mg, 88 %) as a white solid. **S21** was used directly in the next step without further purification.

^{1}H NMR (400 MHz, $CDCl_3$) δ 10.83 (br s, 1H), 4.26 – 4.14 (m, 6H), 3.46 (hept, *J* = 6.7 Hz, 1H), 3.31 (hept, *J* = 6.9 Hz, 1H), 1.40 (d, *J* = 6.9 Hz, 6H), 1.20 (d, *J* = 6.7 Hz, 6H); **HRMS (ESI$^+$)** m/z: $[M+H]^+$ Calcd. for $C_{16}H_{22}NO_3$ 276.1594; Found 276.1600. All spectroscopic data were in accordance with the literature.[12]

***N*^{1},*N*^{1}-Diisopropyl-*N*^{4}-methoxy-*N*^{4}-methylcubane-1,4-dicarboxamide (S22)**

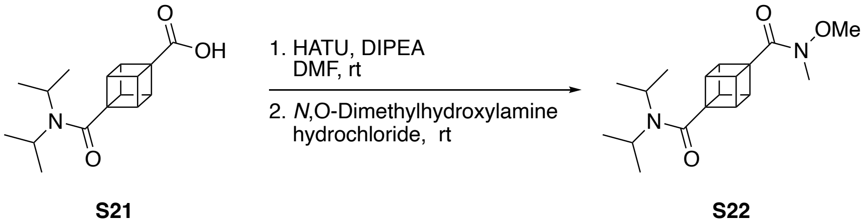

To a solution of 4-(diisopropylcarbamoyl)cubane-1-carboxylic acid (**S21**) (335 mg, 1.0 equiv., 1.22 mmol) and HATU (560 mg, 1.2 equiv., 1.47 mmol) in anhydrous DMF (10 mL) was added *N*,*N*-diisopropylethylamine (DIPEA) (0.72 mL, 3.4 equiv., 4.2 mmol) at rt. After 5 minutes, *N*,O-dimethylhydroxylamine hydrochloride (144 mg, 1.2 equiv., 1.48 mmol) was added in one portion and the reaction mixture was allowed to stir at rt for 2 h. The mixture was quenched with H_2O (3 mL) and diluted with EtOAc (20 mL). The organic layer was washed with H_2O (3 x 15 mL), sat. brine (15 mL), dried with anhydrous $MgSO_4$, filtered, and concentrated *in vacuo* to afford the crude. Purification by silica gel column chromatography (70:30 EtOAc: petroleum ether) gave the title compound **S22** (309 mg, 80 %) as a white solid.

^{1}H NMR (500 MHz, $CDCl_3$) δ 4.21 – 4.15 (m, 3H), 4.15 – 4.09 (m, 3H), 3.68 (s, 3H), 3.47 (hept, *J* = 6.6 Hz, 1H), 3.28 (hept, *J* = 6.8 Hz, 1H), 3.16 (s, 3H), 1.38 (d, *J* = 6.8 Hz, 6H), 1.18 (d, *J* = 6.6

Hz, 6H); **^{13}C NMR** (126 MHz, $CDCl_3$) δ 173.2, 170.5, 61.8, 58.9, 56.5, 48.5, 46.9, 46.4, 45.9, 32.6, 21.1, 20.6; **HRMS (ESI$^+$)** m/z: $[M+H]^+$ Calcd. for $C_{18}H_{27}N_2O_3$ 319.2016; Found 319.2017.

4-Isobutyryl-*N,N*-diisopropylcubane-1-carboxamide (1j)

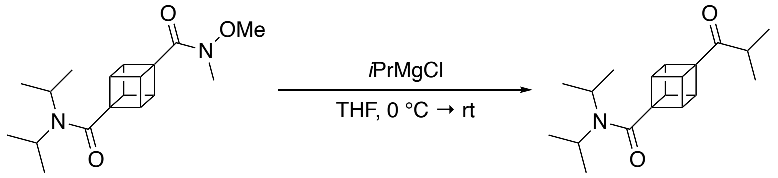

S22 **1j**

To a solution of N^1,N^1-diisopropyl-N^4-methoxy-N^4-methylcubane-1,4-dicarboxamide (**S22**) (280 mg, 1.0 equiv., 0.88 mmol) in anhydrous THF (5 mL) at 0 °C was added isopropylmagnesium chloride (1.32 mL, 3.0 equiv., 2 M in THF) dropwise. The mixture was stirred at 0 °C for 5 minutes and allowed to warm to rt. After 1.5 hours, the mixture was quenched with 1 M HCl (5 mL) at 0 °C. The aqueous was extracted with EtOAc (3 x 10 mL) and the combined organic layers were washed with sat. brine (10 mL), dried with anhydrous $MgSO_4$, filtered and concentrated *in vacuo* to afford the crude. Purification by silica gel column chromatography (1:3 EtOAc: petroleum ether) gave the title compound **1j** (142 mg, 47 %) as a white solid.

^{1}H NMR (400 MHz, $CDCl_3$) δ 4.25 – 4.18 (m, 3H), 4.18 – 4.12 (m, 3H), 3.47 (hept, *J* = 6.6 Hz, 1H), 3.30 (hept, *J* = 6.8 Hz, 1H), 2.78 (hept, *J* = 6.9 Hz, 1H), 1.40 (d, *J* = 6.8 Hz, 6H), 1.19 (d, *J* = 6.6 Hz, 6H), 1.09 (d, *J* = 6.9 Hz, 6H); **^{13}C NMR** (101 MHz, $CDCl_3$) δ 211.9, 170.2, 61.9, 59.4, 48.6, 46.9, 46.7, 46.0, 37.7, 21.1, 20.6, 18.0; **HRMS (ESI$^+$)** m/z: $[M+H]^+$ Calcd. for $C_{19}H_{28}NO_2$ 302.2115; Found 302.2122.

Synthesis of 1k:

Methyl-4-(bromocarbonyl)cubane-1-carboxylate (S23)

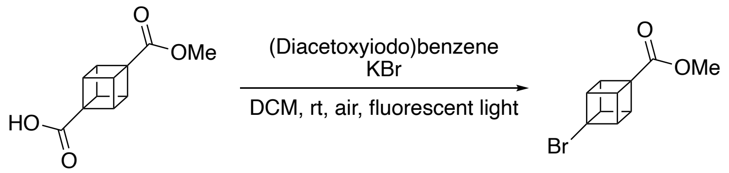

S1 **S23**

Following the procedure of Watanabe and co-workers.[13] To a solution of the commercially available 4-(methoxycarbonyl)cubane-1-carboxylic acid (**S1**) (300 mg, 1.0 equiv., 1.45 mmol) and (diacetoxyiodo)benzene (701 mg,1.5 equiv., 2.18 mmol) in anhydrous DCM (2.9 mL) was added potassium bromide (310 mg, 1.8 equiv., 2.61 mmol) at rt under air in the presence of the standard fluorescent light fitting in the fume hood. After 24 hours the reaction mixture was filtered through a pad of celite and the solid was washed with DCM (15 mL) and the filtrate was concentrated *in vacuo* to afford the crude. Purification by silica gel column chromatography (02:98 EtOAc: petroleum ether) gave the title compound **S23** (167 mg, 48 %) as a white solid.

^{1}**H NMR** (300 MHz, $CDCl_3$) δ 4.35 – 4.28 (m, 3H), 4.28 – 4.20 (m, 3H), 3.70 (s, 3H); **HRMS (EI$^+$)** m/z: [M-OMe]$^+$ Calcd. for $C_9H_6O^{79}Br$ 208.9597; Found 208.9593. All spectroscopic data were in accordance with the literature.[13]

4-Bromocubane-1-carboxylic acid (S24)

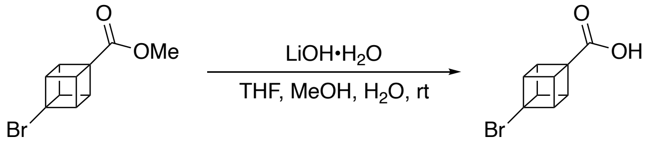

S23 **S24**

To a solution of methyl-4-(bromocarbonyl)cubane-1-carboxylate (**S23**) (150 mg, 1.0 equiv., 0.62 mmol) in 3:3:1 ratio of THF:MeOH:H_2O (1 mL) was added lithium hydroxide monohydrate (65 mg, 2.5 equiv., 1.55 mmol) in one-portion at rt. The mixture was allowed to stir at rt overnight. The mixture was diluted with H_2O (5 mL) and the aqueous layer was washed with EtOAc (10 mL). The aqueous layer was acidified with 2 M HCl, and the aqueous layer was extracted with EtOAc (3 x 10 mL). The combined organic layers were washed sat. brine (10 mL), dried with anhydrous $MgSO_4$, filtered, and concentrated *in vacuo* to afford the crude. The solid was washed with hexane (3 x 5 mL) to afford the title compound **S24** (131 mg, 93 %) as a white solid.

^{1}**H NMR** (400 MHz, $CDCl_3$) δ 4.40 – 4.32 (m, 3H), 4.32 – 4.24 (m, 3H); **HRMS (ESI$^+$)** m/z: [M+H]$^+$ Calcd. for $C_9H_8O_2{}^{79}Br$ 226.9702; Found 226.9710. All spectroscopic data were in accordance with the literature.[14]

4-Bromo-*N*-methoxy-*N*-methylcubane-1-carboxamide (S25)

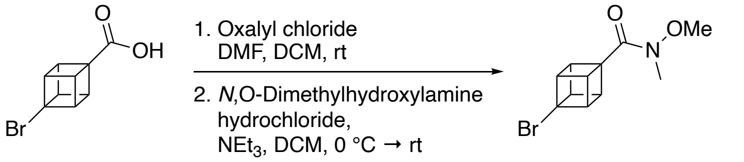

S24 **S25**

To a solution of 4-bromocubane-1-carboxylic acid (**S24**) (125 mg, 1.0 equiv., 0.55 mmol) in anhydrous DCM (3 mL) was added oxalyl chloride (0.47 mL, 1.7 equiv., 2 M in DCM) at rt. Anhydrous DMF (5 μL, 10 mol%) was then added and the reaction mixture was allowed to stir at rt for 1.5 h. The mixture was concentrated *in vacuo* to afford the acid chloride as a yellow solid.

The acid chloride was dissolved in anhydrous DCM (1.5 mL) and added dropwise to a solution of *N*,O-dimethylhydroxylamine hydrochloride (86 mg, 1.6 equiv., 0.88 mmol) and triethylamine (0.24 mL, 3.2 equiv., 1.76 mmol) in anhydrous DCM (1.5 mL) at 0 °C. The mixture was warmed to rt and stirred for 2.5 h. The mixture was quenched with sat. NH_4Cl (5 mL) and diluted with DCM (10 mL). The organic layer was washed with 1 M HCl (10 mL), sat. brine (10 mL), dried with anhydrous $MgSO_4$, filtered and concentrated *in vacuo* to afford the crude. Purification by silica gel column chromatography (1:3 EtOAc/petroleum ether) gave the title compound **S25** (93 mg, 62 %) as a white solid.

^{1}H NMR (400 MHz, $CDCl_3$) δ 4.37 – 4.28 (m, 3H), 4.26 – 4.19 (m, 3H), 3.69 (s, 3H), 3.17 (s, 3H); **^{13}C NMR** (101 MHz, $CDCl_3$) δ 172.6, 63.3, 61.7, 58.1, 54.7, 47.9, 32.6; **HRMS (ESI$^+$)** m/z: $[M+H]^+$ Calcd. for $C_{11}H_{13}NO_2{}^{79}Br$ 270.0124; Found 270.0133.

1-(4-Bromocuban-1-yl)-2-methylpropan-1-one (1k)

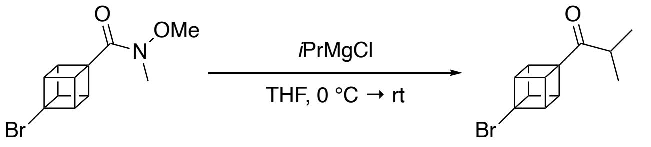

S25 **1k**

To a solution of 4-bromo-*N*-methoxy-*N*-methylcubane-1-carboxamide (**S25**) (93 mg, 1.0 equiv., 0.34 mmol) in anhydrous THF (3 mL) at 0 °C was added isopropylmagnesium chloride (0.52 mL, 3.0 equiv., 2 M in THF) dropwise. The mixture was stirred at 0 °C for 5 minutes and allowed to warm to rt. After 1.5 hours, the mixture was quenched with 1 M HCl (5 mL) at 0 °C. The aqueous was extracted with EtOAc (3 x 10 mL) and the combined organic layers were washed with sat. brine (10 mL), dried with anhydrous $MgSO_4$, filtered and concentrated *in vacuo* to afford the crude. Purification by silica gel column chromatography (03:97 EtOAc: petroleum ether) gave the title compound **1k** (59 mg, 68 %) as a colourless oil.

^{1}H NMR (500 MHz, $CDCl_3$) δ 4.35 – 4.30 (m, 3H), 4.27 – 4.18 (m, 3H), 2.74 (hept, *J* = 6.9 Hz, 1H), 1.07 (d, *J* = 6.9 Hz, 6H); **^{13}C NMR** (126 MHz, $CDCl_3$) δ 211.4, 63.3, 63.2, 54.3, 48.3, 37.8, 18.0; **HRMS (ESI$^+$)** m/z: $[M+H]^+$ Calcd. for $C_{12}H_{14}O^{79}Br$ 253.0223; Found 253.0231.

Synthesis of 1l:

Methyl-4-fluorocubane-1-carboxylate (S26)

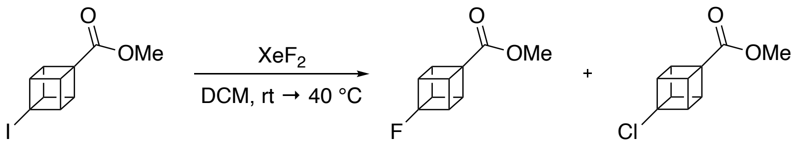

S6 **S26** **S27**

Following the procedure of Della and Head.[3] To a solution of methyl-4-iodocubane-1-carboxylate (**S6**) (150 mg, 1.0 equiv., 0.52 mmol) in anhydrous DCM (3.5 mL) was added xenon difluoride (130 mg, 1.5 equiv., 0.78 mmol) in two portions at rt. The mixture was heated to 40 °C for 6 hours behind a blast shield. After cooling to rt, the mixture was carefully quenched by dropwise addition of 1 M sodium thiosulfate (5 mL), followed by the addition of sat. $NaHCO_3$ (1 mL). The aqueous layer was extracted with DCM (1 x 15 mL), followed by the organic layer being washed with $NaHCO_3$ (2 x 5 mL), dried with anhydrous $MgSO_4$, filtered and concentrated *in vacuo* to afford the crude.

The reaction was repeated a further three times on the same scale and the crude for each was combined for the purification step. ***Note:*** *^{1}H NMR of the combined crude material had a ratio of* ***S26:S27:S6*** *of 4:1:1 before purification.*

Purification by silica gel column chromatography (02:98 EtOAc: petroleum ether) gave a mixture of **S26**:**S27**:**S6** in a ratio of 13:1:1 (142 mg, 38 %) as a white solid.

S26 ^{1}**H NMR** (500 MHz, $CDCl_3$) δ 4.34 – 4.28 (m, 3H), 4.11 – 4.00 (m, 3H), 3.70 (s, 3H); ^{19}**F{**^{1}**H}NMR** (471 MHz, $CDCl_3$) δ -140.36; ^{13}**C NMR** (126 MHz, $CDCl_3$) δ 172.5 (d, *J* = 7.2 Hz), 103.0 (d, *J* = 328.1 Hz), 56.7 (d, *J* = 13.8 Hz), 54.2 (d, *J* = 25.5 Hz), 51.7, 42.4 (d, *J* = 5.5 Hz); **HRMS (EI$^+$)** m/z: $[M-OMe]^+$ Calcd. for C_9H_6OF 149.0397; Found 149.0396.

Towards the synthesis of **1g** compound **S6** was fully characterised. Separately **S27** was synthesised following an adapted procedure of Candish and co-workers:[15]

Methyl-4-chlorocubane-1-carboxylate (S27)

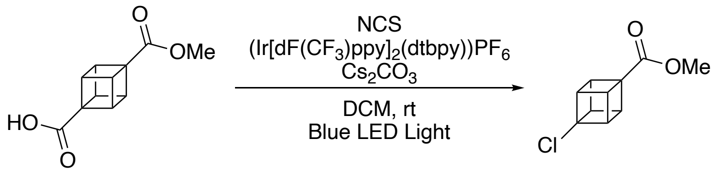

N_2 gas was bubbled through a solution of the commercially available 4-(methoxycarbonyl)cubane-1-carboxylic acid (**S1**) (50 mg, 1.0 equiv., 0.24 mmol), $(Ir[dF(CF_3)ppy]_2(dtbpy))PF_6$ (4.9 mg, 2 mol %), *N*-chlorosuccinimide (64 mg, 2.0 equiv., 0.48 mmol), cesium carbonate (78 mg, 1.0 equiv., 0.24 mmol) in anhydrous DCM (4.8 mL) for 5 minutes. The reaction mixture was then stirred at rt for 16 h whilst being irradiated with blue LED light (Aldrich® micro photochemical reactor blue LED (ALDKIT001) was used as the blue LED light source). The reaction mixture was filtered through a pad of celite and the solid was washed with DCM (15 mL) and the filtrate was concentrated *in vacuo* to afford the crude. Purification by silica gel column chromatography (05:95 EtOAc: petroleum ether) gave the title compound **S27** (13 mg, 28 %) as a white solid.

^{1}**H NMR** (500 MHz, $CDCl_3$) δ 4.25 – 4.20 (m, 3H), 4.19 – 4.12 (m, 3H), 3.71 (s, 3H); ^{13}**C NMR** (126 MHz, $CDCl_3$) δ 172.1, 72.2, 56.5, 54.2, 51.8, 46.0; **HRMS (EI$^+$)** m/z: $[M-OMe]^+$ Calcd. for C_9H_6OCl 165.0102; Found 165.0100.All spectroscopic data were in accordance with the literature.[14, 16]

4-Fluorocubane-1-carboxylic acid (S28)

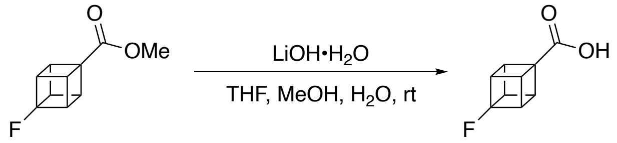

To a mixture of **S26**:**S27**:**6** in a ratio of 13:1:1 (142 mg, 1.0 equiv., 0.79 mmol) in 3:3:1 ratio of THF:MeOH:H_2O (1 mL) was added lithium hydroxide monohydrate (83 mg, 2.5 equiv., 1.97 mmol) in one-portion at rt. The mixture was allowed to stir at rt overnight. The aqueous layer was acidified with 2 M HCl, and the aqueous layer was extracted with EtOAc (3 x 10 mL). The combined organic layers were washed sat. brine (10 mL), dried with anhydrous

$MgSO_4$, filtered and concentrated *in vacuo* to afford the title compound **S28** (123 mg, 94 %) as a white solid. **S28** was used in the next step without further purification.

^{1}H NMR (400 MHz, $CDCl_3$) δ 11.39 (s, 1H), 4.39 – 4.29 (m, 3H), 4.15 – 4.06 (m, 3H); **^{19}F{^{1}H}NMR** (376 MHz, $CDCl_3$) δ -140.41; **^{13}C NMR** (101 MHz, $CDCl_3$) δ 178.6 (d, *J* = 7.2 Hz), 102.8 (d, *J* = 328.1 Hz), 56.4 (d, *J* = 13.7 Hz), 54.1 (d, *J* = 25.7 Hz), 42.4 (d, *J* = 5.5 Hz); **HRMS (EI$^+$)** m/z: $[M]^{+\bullet}$ Calcd. for $C_9H_7O_2F$ 166.0425; Found 166.0421.

***Note:** ^{1}H NMR shows that the product contains 9% of 4-chlorocubane-1-carboxylic acid and 6% of 4-iodocubane-1-carboxylic acid.*

4-Fluoro-*N*-methoxy-*N*-methylcubane-1-carboxamide (S29)

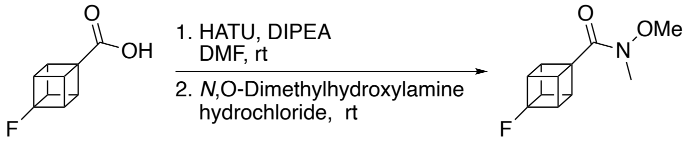

S28 **S29**

To a solution of 4-fluorocubane-1-carboxylic acid (**S28**) (119 mg, 1.0 equiv., 0.72 mmol) and HATU (326 mg, 1.2 equiv., 0.86 mmol) in anhydrous DMF (5 mL) was added *N*,*N*-diisopropylethylamine (DIPEA) (0.43 mL, 3.5 equiv., 2.52 mmol) at rt. After 5 minutes, *N*,O-dimethylhydroxylamine hydrochloride (83 mg, 1.2 equiv., 0.86 mmol) was added in one portion and the reaction mixture was allowed to stir at rt for 2 h. The mixture was quenched with H_2O (3 mL) and diluted with EtOAc (20 mL). The organic layer was washed with H_2O (3 x 15 mL), sat. brine (15 mL), dried with anhydrous $MgSO_4$, filtered and concentrated *in vacuo* to afford the crude. Purification by silica gel column chromatography (30:70 EtOAc: petroleum ether) gave the title compound **S29** (93 mg, 62 %) as a cream solid.

^{1}H NMR (500 MHz, $CDCl_3$) δ 4.30fh – 4.22 (m, 3H), 4.08 – 4.02 (m, 3H), 3.68 (s, 3H), 3.15z (s, 3H); **^{19}F{^{1}H}NMR** (471 MHz, $CDCl_3$) δ -141.11; **^{13}C NMR** (126 MHz, $CDCl_3$) δ 173.3, 102.8 (d, *J* = 328.3 Hz), 61.8, 58.5 (d, *J* = 14.6 Hz), 54.0 (d, *J* = 25.2 Hz), 42.5 (d, *J* = 5.5 Hz), 32.7; **HRMS (EI$^+$)** m/z: $[M\text{-}OMe]^+$ Calcd. for $C_{10}H_9FNO$ 178.0663; Found 178.0661.

***Note:** ^{1}H NMR shows that the product contains 6% of 4-chloro-N-methoxy-N-methylcubane-1-carboxamide and 2% of 4-iodo-N-methoxy-N-methylcubane-1-carboxamide.*

1-(4-Fluorocuban-1-yl)-2-methylpropan-1-one (1l)

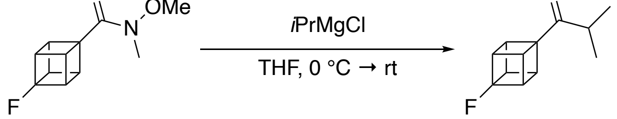

S29 **1l**

To a solution of 4-fluoro-*N*-methoxy-*N*-methylcubane-1-carboxamide (**S29**) (88 mg, 1.0 equiv., 0.42 mmol) in anhydrous THF (2.5 mL) at 0 °C was added isopropylmagnesium chloride (0.63 mL, 3.0 equiv., 2 M in THF) dropwise. The mixture was stirred at 0 °C for 5 minutes and allowed to warm to rt. After 2 hours, the mixture was quenched with 1 M HCl

(5 mL) at 0 °C. The aqueous was extracted with EtOAc (3 x 10 mL) and the combined organic layers were washed with sat. brine (10 mL), dried with anhydrous $MgSO_4$, filtered and concentrated *in vacuo* to afford the crude orange solid. Purification by silica gel column chromatography (02:98 EtOAc: petroleum ether) gave the title compound **1l** (61 mg, 75 %) as a white solid.

^{1}H NMR (400 MHz, $CDCl_3$) δ 4.35 – 4.27 (m, 3H), 4.12 – 4.04 (m, 3H), 2.76 (hept, *J* = 6.9 Hz, 1H), 1.09 (d, *J* = 6.9 Hz, 6H); **^{19}F{^{1}H}NMR** (376 MHz, $CDCl_3$) δ -140.25; **^{13}C NMR** (101 MHz, $CDCl_3$) δ 212.2 (d, *J* = 6.3 Hz), 102.8 (d, *J* = 329.0 Hz), 63.9 (d, *J* = 13.3 Hz), 53.8 (d, *J* = 25.4 Hz), 43.0 (d, *J* = 5.5 Hz), 37.8, 18.0; **HRMS (EI$^+$)** m/z: $[M]^{+\bullet}$ Calcd. for $C_{12}H_{13}OF$ 192.0945; Found 192.0942.

***Note:** ^{1}H NMR shows that the product contains 6% of 4-chloro-N-methoxy-N-methylcubane-1-carboxamide*

Baeyer-Villiger

Baeyer-Villiger oxidation optimisation:

Table S1: Screening of peracid loading in dichloromethane and chloroform.

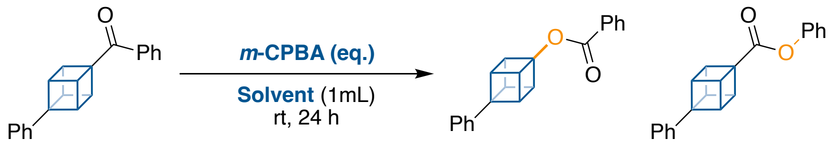

1a
(0.1 mmol)
2a
3a

Entry	*m*-CPBA / equiv[b]	Solvent	NMR yield[a] / %		
			**1a (% consumption)**	**2a**	**3a**
**1**	2	DCM	35	20	0
**2**	2	$CHCl_3$	31	30	0
**3**	4	DCM	66	42	3
**4**	4	$CHCl_3$	74	46	4
**5**	8	DCM	86	63	3
**6**	8	$CHCl_3$	90	72	5

[a] NMR yields were determined by ^{1}H NMR analysis using the internal standard durene.
[b] The equivalents of *m*-CPBA have not taken into account its purity (≤77 %)

***Note:** Whilst no issues were encountered during the course of this work, caution must be employed when heating solutions of m-CPBA.*

Table S2: Screening of catalyst, temperature and time.

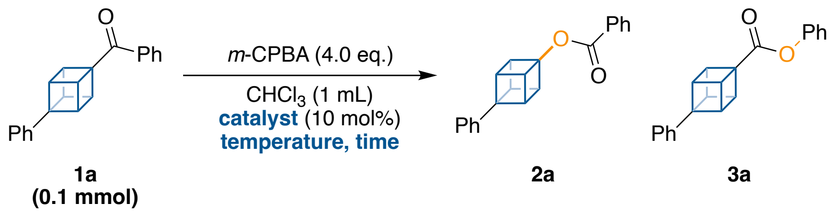

Entry	Catalyst	Temperature / °C	Time / h	NMR yield[a] / %		
				1a (% consumption)	2a	3a
**1**	-	rt	24	74	46	4
**2**	$Sc(OTf)_3$	rt	24	65	51	3
**3**	$Sc(OTf)_3$	rt	6	35	19	0
**4**[b]	$Sc(OTf)_3$	50	6	87	51	3
**5**	$BF_3{\cdot}OEt_2$	50	6	84	51	3
**6**	$Sc(OTf)_3$	50	3	65	46	3

[a] NMR yields were determined by ^{1}H NMR analysis using the internal standard durene.
[b] Optimised reaction conditions.

Baeyer-Villiger general procedure:

A 3 mL reaction vial was charged with cubane carbonyl (1.0 eq., 0.2 mmol), scandium (III) triflate (10 mg, 0.1 eq., 0.02 mmol) and 3-chloroperbenzoic acid (172 mg, 4.0 equiv., 0.77 mmol) and then flushed with N_2. The mixture was dissolved in anhydrous chloroform (2 mL) and stirred at 50 °C for 6 hours. After cooling to rt, the reaction mixture was quenched with sodium bisulfite (0.5 mL).

Workup 1: The reaction mixture was diluted with DCM (5 mL) and the organic layer was washed further with sodium bisulfite (10 mL). The aqueous layer was then re-extracted with DCM (3 x 5 mL) and the combined organic layers were dried with anhydrous $MgSO_4$, filtered and concentrated *in vacuo* to afford the crude. By ^{1}H NMR ($CDCl_3$) the crude material contain 3-chlorobenzoic acid as a by-product (highlighted in blue).

Workup 2: The crude from workup 1 was dissolved in DCM (15 mL) and washed with sat. $NaHCO_3$ (3 x 5 mL), dried with anhydrous $MgSO_4$, filtered, and concentrated *in vacuo* to afford the crude. The entire sample of the crude was dissolved in $CDCl_3$ (0.7 mL), added to a known mass of 1,2,4,5-tetramethylbenzene (internal standard) and transferred to an NMR tube for ^{1}H NMR ($CDCl_3$) analysis to determine % yield of products and unreacted starting material. By ^{1}H NMR the crude material will contain the internal standard (highlighted in red).

$$\% \, Yield = \frac{nIS * (rP/rIS)}{nSM}$$

Where:

P = Product

*n*IS = mmol of internal standard (1,2,4,5-tetramethylbenzene)

*n*SM = mmol of cubane starting material

$$rP/rIS = \frac{Integral\ of\ P\ /\ no.of\ protons}{Integral\ of\ IS\ /\ no.of\ protons}$$

Baeyer-Villiger substrate scope:

Baeyer-Villiger of phenyl(4-phenylcuban-1-yl)methanone (1a)

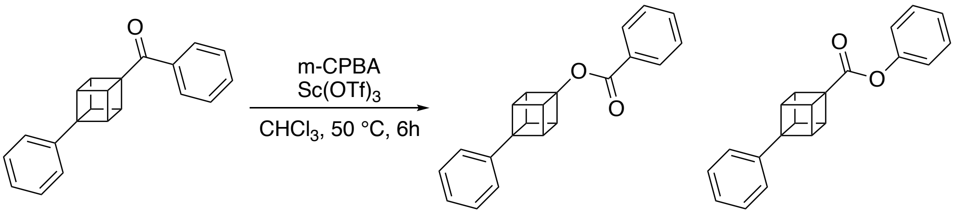

1a **2a** **3a**

The reaction was performed using the **general procedure** with phenyl(4-phenylcuban-1-yl)methanone (**1a**) (57 mg, 1.0 eq., 0.20 mmol), scandium (III) triflate (10.1 mg, 0.1 eq., 0.02 mmol), 3-chloroperbenzoic acid (170 mg, 4.0 equiv., 0.73 mmol) and $CHCl_3$ (2.0 mL). Purification by silica column chromatography (30:70 DCM/petroleum ether → 50:50 DCM/petroleum ether) gave the title compound **2a** (35 mg, 58 %) as a white solid.

^{1}H NMR (500 MHz, $CDCl_3$) δ 8.13 – 8.08 (m, 2H), 7.64 – 7.57 (m, 1H), 7.52 – 7.45 (m, 2H), 7.43 – 7.36 (m, 2H), 7.29 – 7.20 (m, 3H), 4.47 – 4.38 (m, 3H), 4.20 – 4.11 (m, 3H); **^{13}C NMR** (126 MHz, $CDCl_3$) δ 165.2, 142.6, 133.3, 129.9, 129.8, 128.6, 128.6, 126.2, 124.9, 89.2, 60.1, 51.6, 45.9; **HRMS (EI$^+$)** m/z: $[M-C_7H_5O]^+$ Calcd. for $C_{14}H_{11}O$ 195.0804; Found 195.0804.

Note: *The isolated yield of **3a** was not determined as it was not found during the purification process. However, **3a** was synthesised separately and fully characterised:*

NMR yield of **1a** / %	NMR yield of **2a** / %	NMR yield of **3a** / %	Isolated yield of **1a** / %	Isolated yield of **2a** / %	Isolated yield of **3a** / %
13	51	3	10	58	n.d

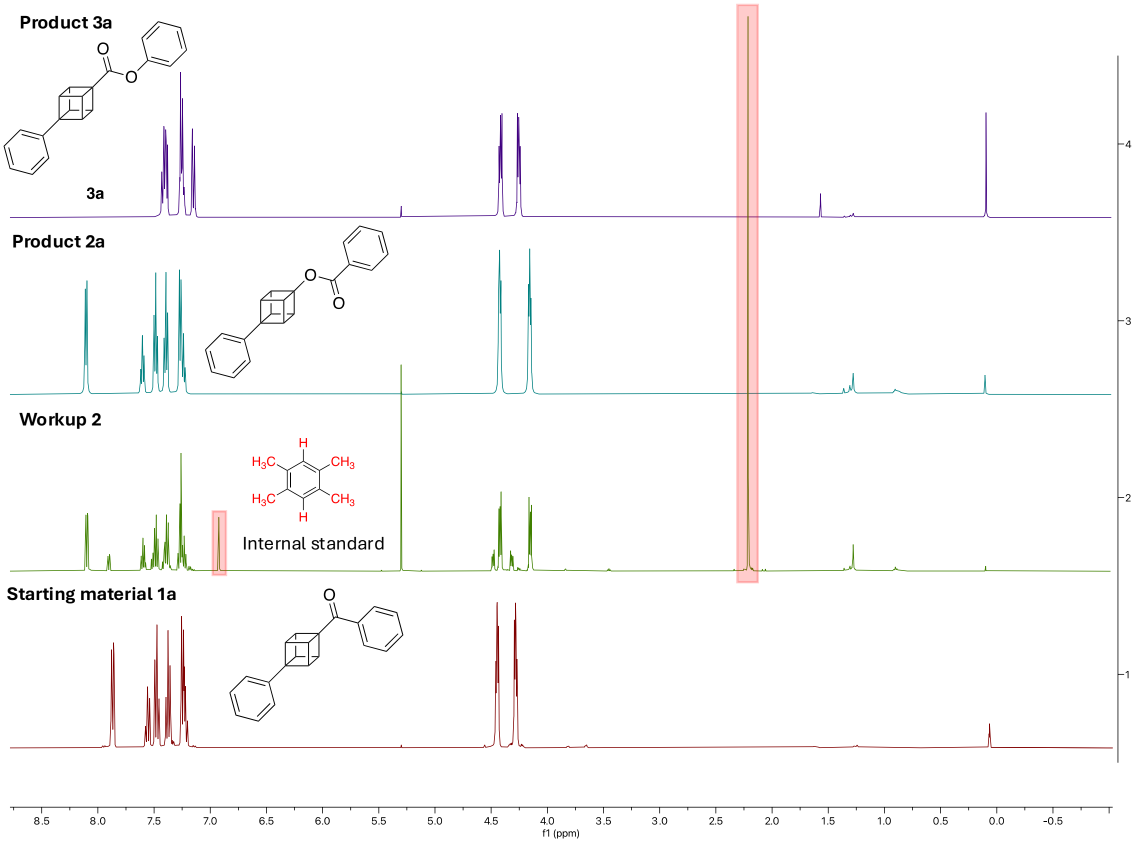

Phenyl-4-phenylcubane-1-carboxylate (3a)

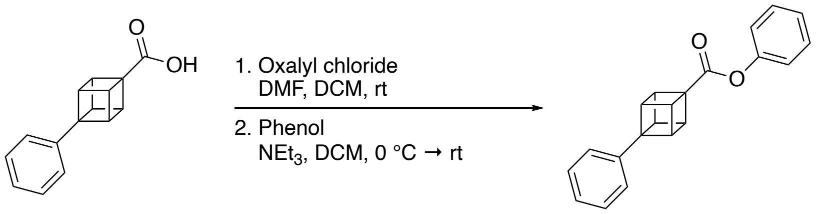

S4 **3a**

To a solution of 4-phenylcubane-1-carboxylic acid (**S4**) (100 mg, 1.0 equiv., 0.45 mmol) in anhydrous DCM (3 mL) was added oxalyl chloride (0.38 mL, 1.7 equiv., 2 M in DCM) at rt. Anhydrous DMF (4 µL, 10 mol%) was then added and the reaction mixture was allowed to stir at rt for 1.5 h. The mixture was concentrated *in vacuo* to afford the acid chloride as a yellow solid.

The acid chloride was dissolved in anhydrous DCM (1.5 mL) and added dropwise to a solution of phenol (68 mg, 1.6 equiv., 0.72 mmol) and triethylamine (0.2 mL, 3.2 equiv., 1.44 mmol) in anhydrous DCM (1.5 mL) at 0 °C. The mixture was warmed to rt and stirred for 2.5 h. The mixture was quenched with sat. NH_4Cl (5 mL) and diluted with DCM (10 mL). The organic layer was washed with 1 M HCl (10 mL), sat. brine (10 mL), dried with anhydrous $MgSO_4$, filtered and concentrated *in vacuo* to afford the crude. Purification by silica gel

column chromatography (1:9 EtOAc/petroleum ether) gave the title compound **3a** (128 mg, 95 %) as a white solid.

^{1}H NMR (400 MHz, $CDCl_3$) δ 7.46 – 7.35 (m, 4H), 7.29 – 7.20 (m, 4H), 7.19 – 7.11 (m, 2H), 4.46 – 4.36 (m, 3H), 4.30 – 4.21 (m, 3H); **^{13}C NMR** (101 MHz, $CDCl_3$) δ 170.7, 150.8, 142.0, 129.5, 128.6, 126.4, 125.9, 124.9, 121.7, 60.4, 56.6, 49.0, 46.4; **HRMS (ESI$^+$)** m/z: $[M+H]^+$ Calcd. for $C_{21}H_{17}O_2$ 301.1229; Found 301.1241.

Baeyer-Villiger of 1-(4-phenylcuban-1-yl)ethan-1-one (1b)

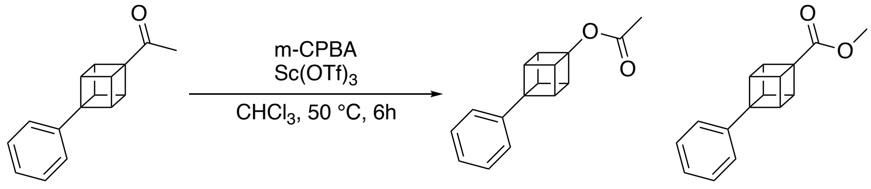

The reaction was performed using the **general procedure** with 1-(4-phenylcuban-1-yl)ethan-1-one (**1b**) (45 mg, 1.0 eq., 0.2 mmol), scandium (III) triflate (10 mg, 0.1 eq., 0.02 mmol), 3-chloroperbenzoic acid (172 mg, 4.0 equiv., 0.77 mmol) and $CHCl_3$ (2 mL). Purification by silica gel column chromatography (30:70 DCM/petroleum ether) gave the title compound **2b** (42 mg, 86 %) as a colourless oil.

^{1}**H NMR** (400 MHz, $CDCl_3$) δ 7.41 – 7.31 (m, 2H), 7.24 – 7.17 (m, 3H), 4.33 – 4.22 (m, 3H), 4.12 – 4.00 (m, 3H), 2.12 (s, 3H); **^{13}C NMR** (101 MHz, $CDCl_3$) δ 169.6, 142.7, 128.6, 126.2, 124.9, 88.7, 60.1, 51.4, 45.8, 21.2; **HRMS** (EI$^+$) m/z: $[M]^{+\bullet}$ Calcd. for $C_{16}H_{14}O_2$ 238.0988; Found 238.0988.

NMR yield of **1b** / %	NMR yield of **2b** / %	NMR yield of **3b** / %	Isolated yield of **1b** / %	Isolated yield of **2b** / %	Isolated yield of **3b** / %
0	79	0	0	86	0

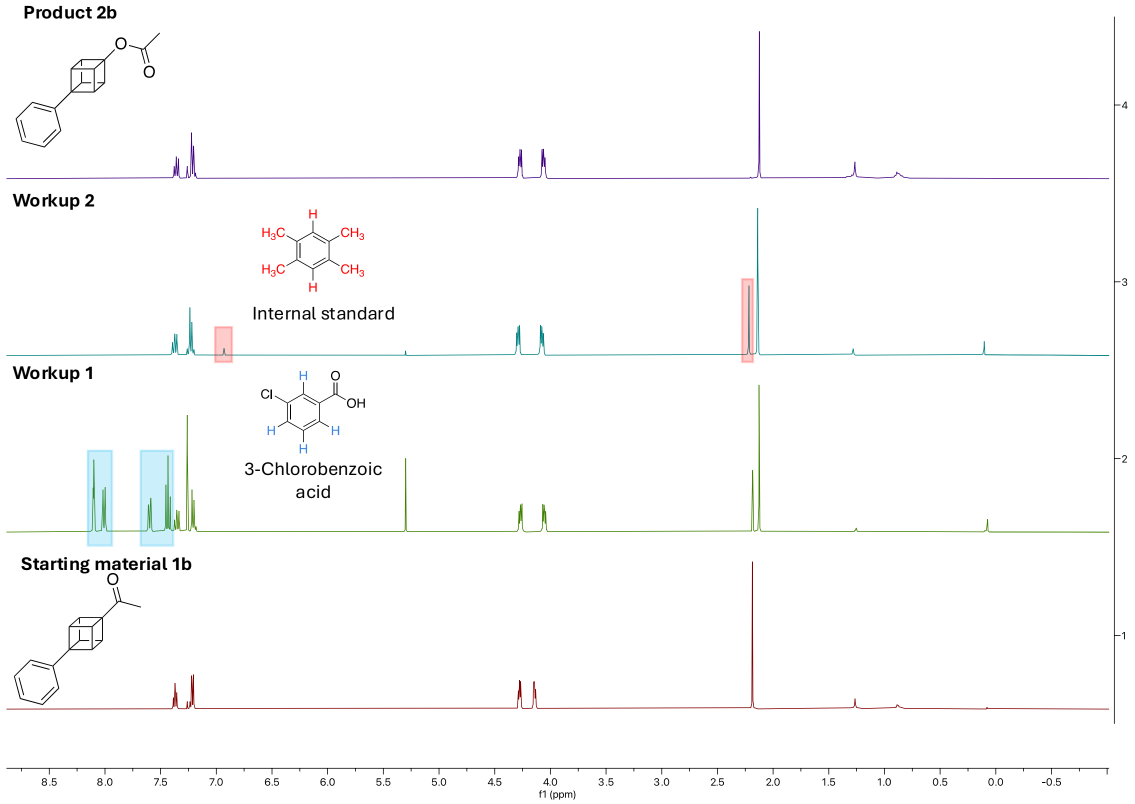

Baeyer-Villiger of 1-(4-phenylcuban-1-yl)propan-1-one (1c)

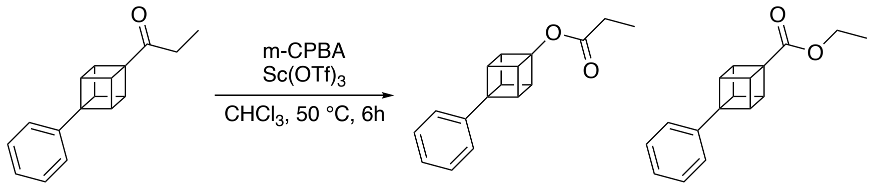

The reaction was performed using the **general procedure** with 1-(4-phenylcuban-1-yl)propan-1-one (**1c**) (45 mg, 1.0 eq., 0.19 mmol), scandium (III) triflate (10 mg, 0.1 eq., 0.02 mmol), 3-chloroperbenzoic acid (163 mg, 4.0 equiv., 0.73 mmol) and $CHCl_3$ (1.9 mL). Purification by silica gel column chromatography (30:70 DCM/petroleum ether) gave the title compound **2c** (36 mg, 76 %) as a white solid.

^{1}H NMR (400 MHz, $CDCl_3$) δ 7.40 – 7.33 (m, 2H), 7.25 – 7.18 (m, 3H), 4.34 – 4.23 (m, 3H), 4.13 – 4.02 (m, 3H), 2.42 (q, *J* = 7.6 Hz, 2H), 1.19 (t, *J* = 7.6 Hz, 3H); **^{13}C NMR** (101 MHz, $CDCl_3$) δ 173.1, 142.6, 128.5, 126.1, 124.9, 88.7, 60.1, 51.4, 45.7, 27.6, 9.0; **HRMS** (CI^+) m/z: $[M-H]^+$ Calcd. for $C_{17}H_{15}O_2$ 251.1067; Found 251.1065.

NMR yield of **1c** / %	NMR yield of **2c** / %	NMR yield of **3c** / %	Isolated yield of **1c** / %	Isolated yield of **2c** / %	Isolated yield of **3c** / %
0	87	0	0	76	0

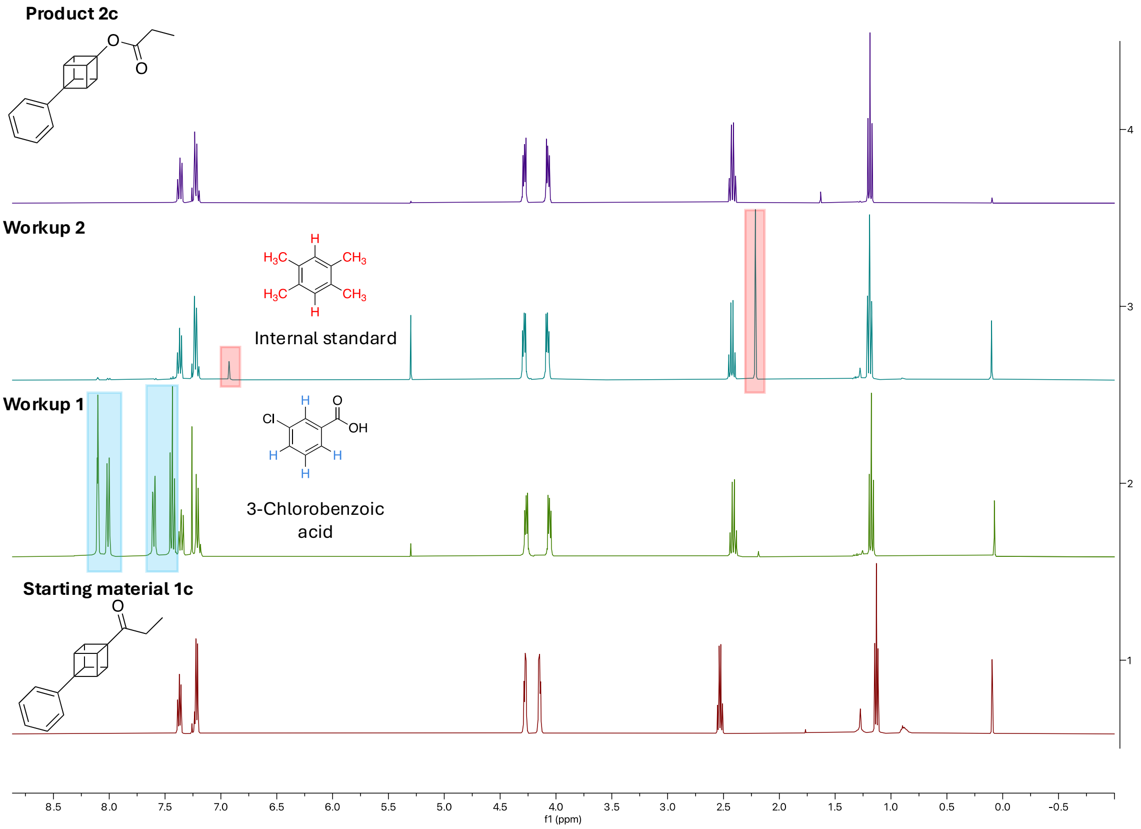

Baeyer-Villiger of 4-phenylcubane-1-carbaldehyde (1d)

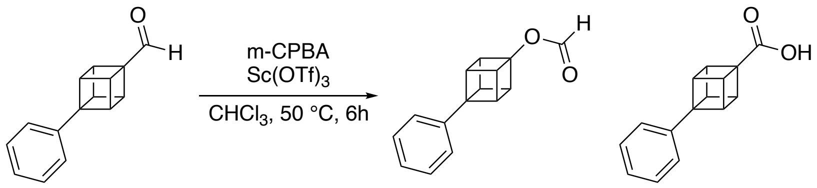

1d **2d** **3d**

The reaction was performed using the **general procedure** (workup 2 not used for this substrate) with 4-phenylcubane-1-carbaldehyde (**1d**) (41.4 mg, 1.0 eq., 0.20 mmol), scandium (III) triflate (9.9 mg, 0.1 eq., 0.02 mmol), 3-chloroperbenzoic acid (174 mg, 4.0 equiv., 0.73 mmol) and $CHCl_3$ (2.0 mL). Purification by silica column chromatography (3:97 EtOAc/petroleum ether → 1:1 EtOAc/petroleum ether) gave the title compound **2d** (29 mg, 65 %) as a white solid.

^{1}H NMR (400 MHz, $CDCl_3$) δ 8.07 (s, 1H), 7.42 – 7.32 (m, 2H), 7.25 – 7.18 (m, 3H), 4.38 – 4.26 (m, 3H), 4.15 – 4.04 (m, 3H); **^{13}C NMR** (101 MHz, $CDCl_3$) δ 159.3, 142.4, 128.6, 126.3, 124.9,

89.0, 60.1, 51.5, 45.9; **HRMS (EI⁺)** m/z: [M-COH]⁺ Calcd. for $C_{14}H_{11}O$ 195.0804; Found 195.0803.

Note: *The isolated yield of* ***3d*** *was not determined, as it was not possible to separate product* ***3d*** *with the by-product 3-chlorobenzoic acid during the purification.*

NMR yield of **1d** / %	NMR yield of **2d** / %	NMR yield of **3d** / %	Isolated yield of **1d** / %	Isolated yield of **2d** / %	Isolated yield of **3d** / %
0	55	28	0	65	n.d

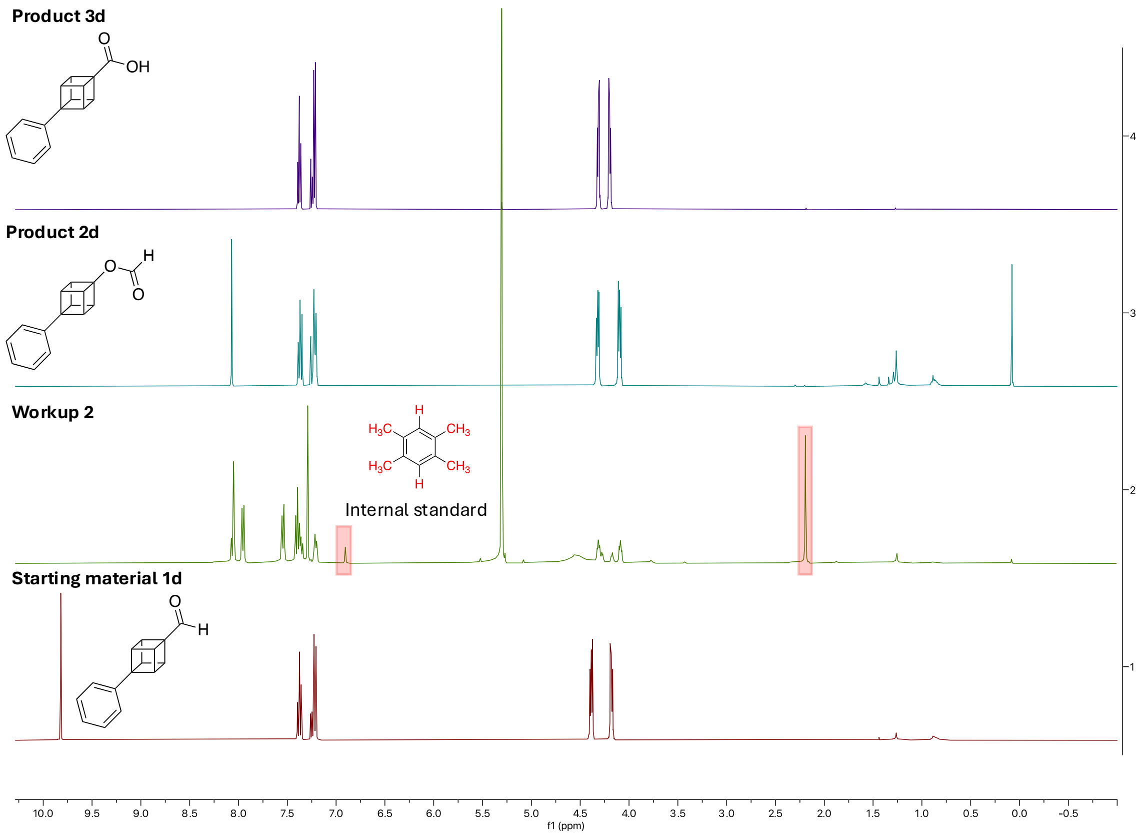

Baeyer-Villiger of 2-methyl-1-(4-phenylcuban-1-yl)propan-1-one (1e)

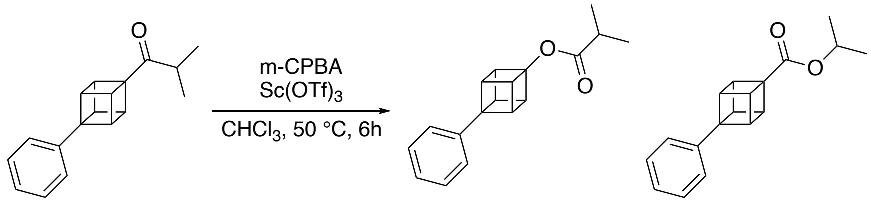

The reaction was performed using the **general procedure** with 2-methyl-1-(4-phenylcuban-1-yl)propan-1-one (**1e**) (50 mg, 1.0 eq., 0.20 mmol), scandium (III) triflate (10.1 mg, 0.1 eq., 0.02 mmol), 3-chloroperbenzoic acid (174 mg, 4.0 equiv., 0.73 mmol) and $CHCl_3$ (2.0 mL). Purification by silica gel column chromatography (30:70 DCM/petroleum ether → 50:50

DCM/petroleum ether) gave the title compound **2e** (33 mg, 62 %) as a white solid and **3e** (14 mg, 27%) as a white solid.

2e: ^{1}**H NMR** (400 MHz, $CDCl_3$) δ 7.40 – 7.33 (m, 2H), 7.25 – 7.18 (m, 3H), 4.33 – 4.21 (m, 3H), 4.13 – 4.02 (m, 3H), 2.62 (hept, *J* = 7.0 Hz, 1H), 1.23 (d, *J* = 7.0 Hz, 6H); ^{13}**C NMR** (101 MHz, $CDCl_3$) δ 175.9, 142.7, 128.5, 126.1, 124.9, 88.7, 60.0, 51.4, 45.7, 34.0, 19.0; **HRMS (EI$^+$)** m/z: $[M]^{+\bullet}$ Calcd. for $C_{18}H_{18}O_2$ 266.1301; Found 266.1301.

3e: ^{1}**H NMR** (400 MHz, $CDCl_3$) δ 7.40 – 7.33 (m, 2H), 7.24 – 7.19 (m, 3H), 5.06 (hept, *J* = 6.3 Hz, 1H), 4.24 – 4.18 (m, 3H), 4.18 – 4.10 (m, 3H), 1.27 (d, *J* = 6.3 Hz, 6H); ^{13}**C NMR** (101 MHz, $CDCl_3$) δ 172.2, 142.4, 128.6, 126.2, 124.9, 67.5, 60.3, 56.9, 48.8, 46.1, 22.0; **HRMS (EI$^+$)** m/z: $[M]^{+\bullet}$ Calcd. for $C_{18}H_{18}O_2$ 266.1301; Found 266.1303.

NMR yield of **1e** / %	NMR yield of **2e** / %	NMR yield of **3e** / %	Isolated yield of **1e** / %	Isolated yield of **2e** / %	Isolated yield of **3e** / %
0	58	25	0	62	27

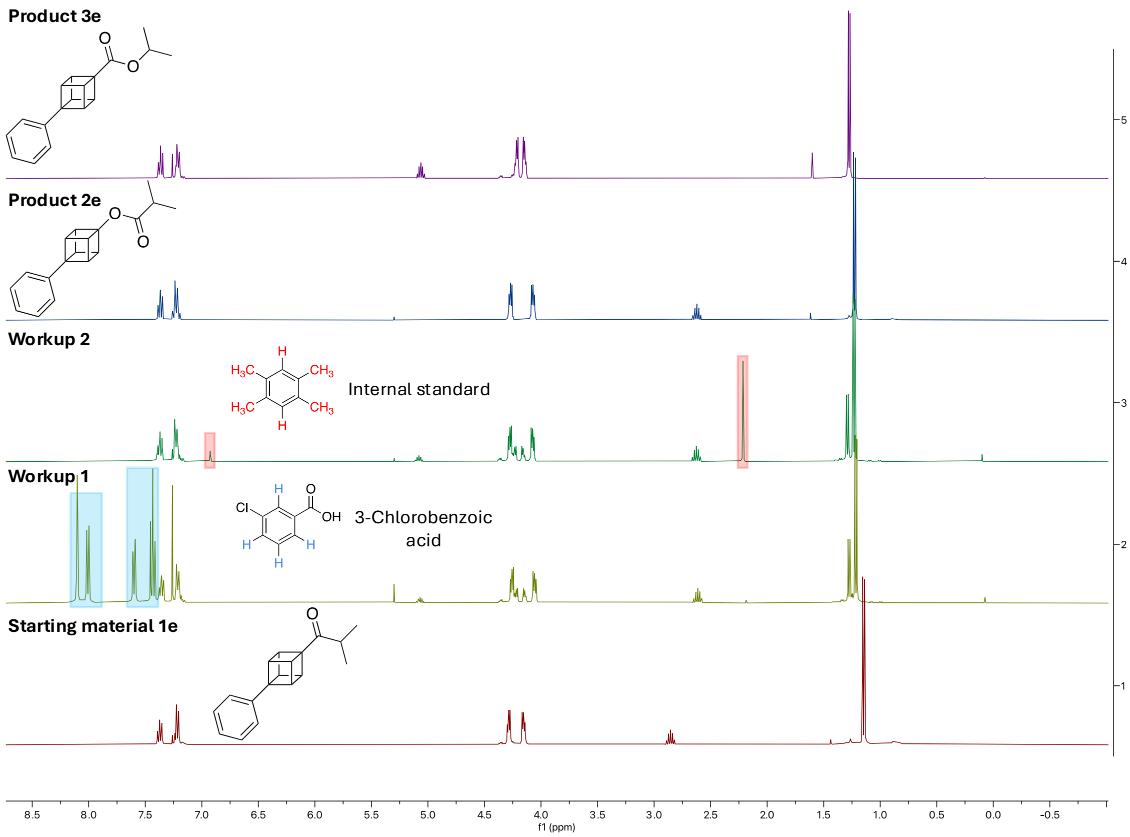

Baeyer-Villiger of 2,2-dimethyl-1-(4-phenylcuban-1-yl)propan-1-one (1f)

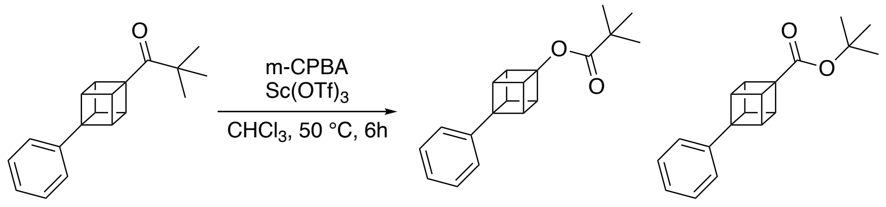

1f **2f** **3f**

The reaction was performed using the **general procedure** with 2,2-dimethyl-1-(4-phenylcuban-1-yl)propan-1-one (**1f**) (54 mg, 1.0 eq., 0.20 mmol), scandium (III) triflate (10.3 mg, 0.1 eq., 0.02 mmol), 3-chloroperbenzoic acid (173 mg, 4.0 equiv., 0.73 mmol) and $CHCl_3$ (2.0 mL). Purification by column chromatography (30:70 DCM/petroleum ether → 50:50 DCM/petroleum ether) gave a mixture of **1f**:**3f** in a ratio of 9:1 as a white solid (42 mg).

3f: ^{1}**H NMR** (500 MHz, $CDCl_3$) δ 7.40 – 7.35 (m, 2H), 7.25 – 7.19 (m, 3H), 4.21 – 4.10 (m, 6H), 1.50 (s, 9H); ^{13}**C NMR** (126 MHz, $CDCl_3$) δ 172.2, 142.4, 128.5, 126.1, 124.9, 80.2, 60.2, 57.6, 48.6, 46.1, 28.3; HRMS (EI$^+$) m/z: $[M-OC_4H_9]^+$ Calcd. for $C_{15}H_{11}O$ 207.0805; Found 207.0802.

NMR yield of **1f** / %	NMR yield of **2f** / %	NMR yield of **3f** / %	Isolated yield of **1f** / %	Isolated yield of **2f** / %	Isolated yield of **3f** / %
64	0	8	69	0	9

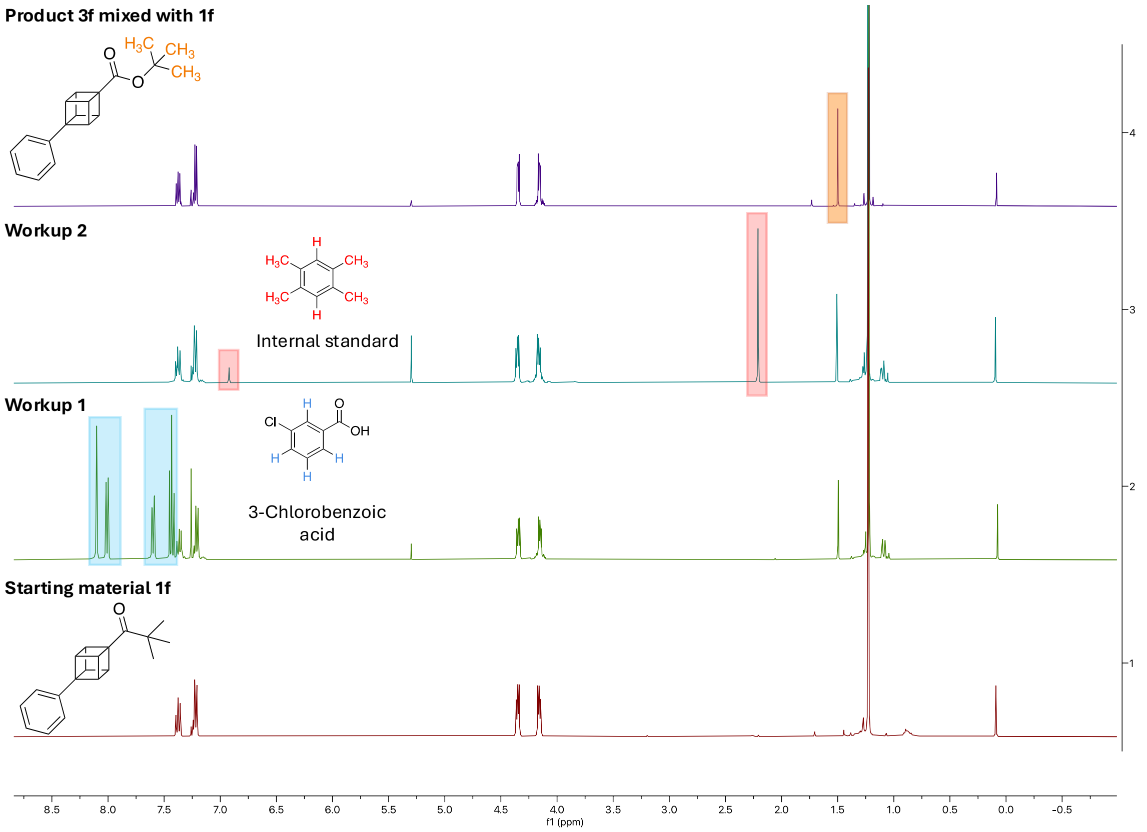

Baeyer-Villiger of 2-methyl-1-(4-(trimethylsilyl)cuban-1-yl)propan-1-one (1g)

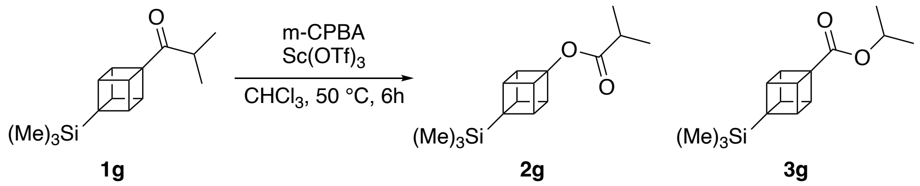

The reaction was performed using the **general procedure** with 2-methyl-1-(4-(trimethylsilyl)cuban-1-yl)propan-1-one (**1g**) (50 mg, 1.0 eq., 0.20 mmol), scandium (III) triflate (9.6 mg, 0.1 eq., 0.02 mmol), 3-chloroperbenzoic acid (177 mg, 4.0 equiv., 0.73 mmol) and $CHCl_3$ (2.0 mL). Purification by column chromatography (30:70 DCM/petroleum ether → 50:50 DCM/petroleum ether) gave the title compound **2g** (32 mg, 61 %) as a colourless oil and impure **3g**. Further purification of **3g** by silica gel column chromatography (02:98 EtOAc/petroleum ether) gave the title compound **3g** (5 mg, 10 %) as a colourless oil.

2g: **^{1}H NMR** (400 MHz, $CDCl_3$) δ 4.30 – 4.21 (m, 3H), 3.79 – 3.69 (m, 3H), 2.57 (hept, *J* = 7.0 Hz, 1H), 1.18 (d, *J* = 7.0 Hz, 6H), -0.05 (s, 9H); **^{13}C NMR** (101 MHz, $CDCl_3$) δ 175.8, 88.4, 55.2, 48.4, 40.9, 34.0, 19.0, -4.7; **HRMS (EI$^+$)** m/z: $[M\text{-}CH_3]^+$ Calcd. for $C_{14}H_{19}O_2{}^{28}Si$ 247.1149; Found 247.1145.

3g: **^{1}H NMR** (400 MHz, $CDCl_3$) δ 5.02 (hept, *J* = 6.2 Hz, 1H), 4.25 – 4.19 (m, 3H), 3.85 – 3.79 (m, 3H), 1.24 (d, *J* = 6.2 Hz, 6H), -0.05 (s, 9H); **^{13}C NMR** (126 MHz, $CDCl_3$) δ 172.1, 67.3, 56.5, 49.8, 48.9, 44.1, 22.0, -4.7; **HRMS (EI$^+$)** m/z: $[M\text{-}C_3H_7]^+$ Calcd. for $C_{12}H_{15}O_2{}^{28}Si$ 219.0836; Found 219.0839.

NMR yield of **1g** / %	NMR yield of **2g** / %	NMR yield of **3g** / %	Isolated yield of **1g** / %	Isolated yield of **2g** / %	Isolated yield of **3g** / %
0	65	9	0	61	10

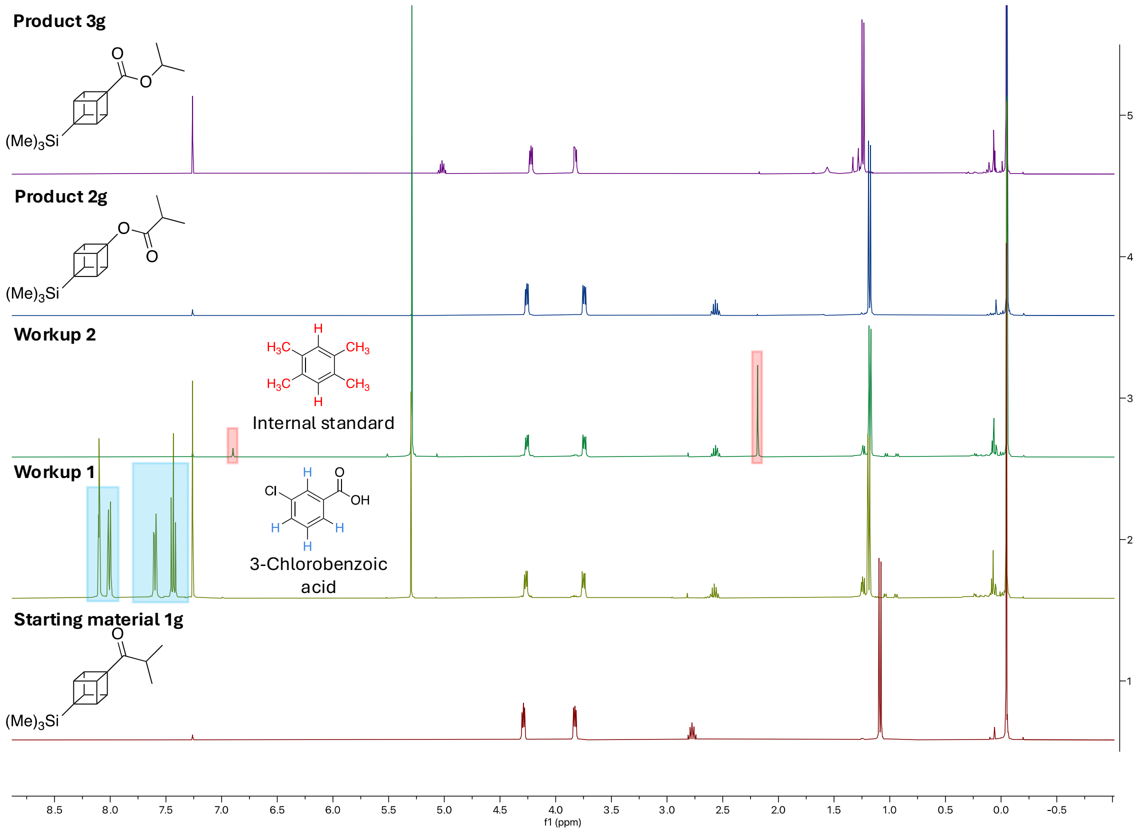

Baeyer-Villiger of 1-(cuban-1-yl)-2-methylpropan-1-one (1h)

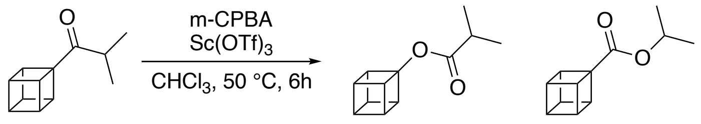

1h **2h** **3h**

The reaction was performed using the **general procedure** with 1-(cuban-1-yl)-2-methylpropan-1-one (**1h**) (34.5 mg, 1.0 eq., 0.20 mmol), scandium (III) triflate (10.1 mg, 0.1 eq., 0.02 mmol), 3-chloroperbenzoic acid (172 mg, 4.0 equiv., 0.73 mmol) and $CHCl_3$ (2.0 mL). Purification by silica gel column chromatography (30:70 DCM/petroleum ether → 50:50 DCM/petroleum ether) gave the title compound **2h** (18 mg, 46 %) as a white solid and impure **3h**. Further purification of **3h** by silica gel column chromatography (3:97 EtOAc/petroleum ether) gave the title compound **3h** (8 mg, 22 %) as a colourless oil.

2h: **^{1}H NMR** (400 MHz, $CDCl_3$) δ 4.30 – 4.21 (m, 3H), 3.96 – 3.87 (m, 4H), 2.57 (hept, *J* = 7.0 Hz, 1H), 1.18 (d, *J* = 7.0 Hz, 6H); **^{13}C NMR** (101 MHz, $CDCl_3$) δ 176.0, 88.2, 54.8, 47.7, 42.0, 34.0, 19.0; **HRMS (EI$^+$)** m/z: $[M-H]^+$ Calcd. for $C_{12}H_{13}O_2$ 189.0910; Found 189.0909.

3h: **^{1}H NMR** (500 MHz, $CDCl_3$) δ 5.02 (hept, *J* = 6.3 Hz, 1H), 4.25 – 4.18 (m, 3H), 4.03 – 3.95 (m, 4H), 1.24 (d, *J* = 6.3 Hz, 6H); **^{13}C NMR** (126 MHz, $CDCl_3$) δ 172.3, 67.3, 56.1, 49.5, 47.9, 45.2, 22.0; **HRMS (EI$^+$)** m/z: $[M]^{+\bullet}$ Calcd. for $C_{12}H_{14}O_2$ 190.0988; Found 190.0988.

NMR yield of **1h** / %	NMR yield of **2h** / %	NMR yield of **3h** / %	Isolated yield of **1h** / %	Isolated yield of **2h** / %	Isolated yield of **3h** / %
0	44	15	0	46	22

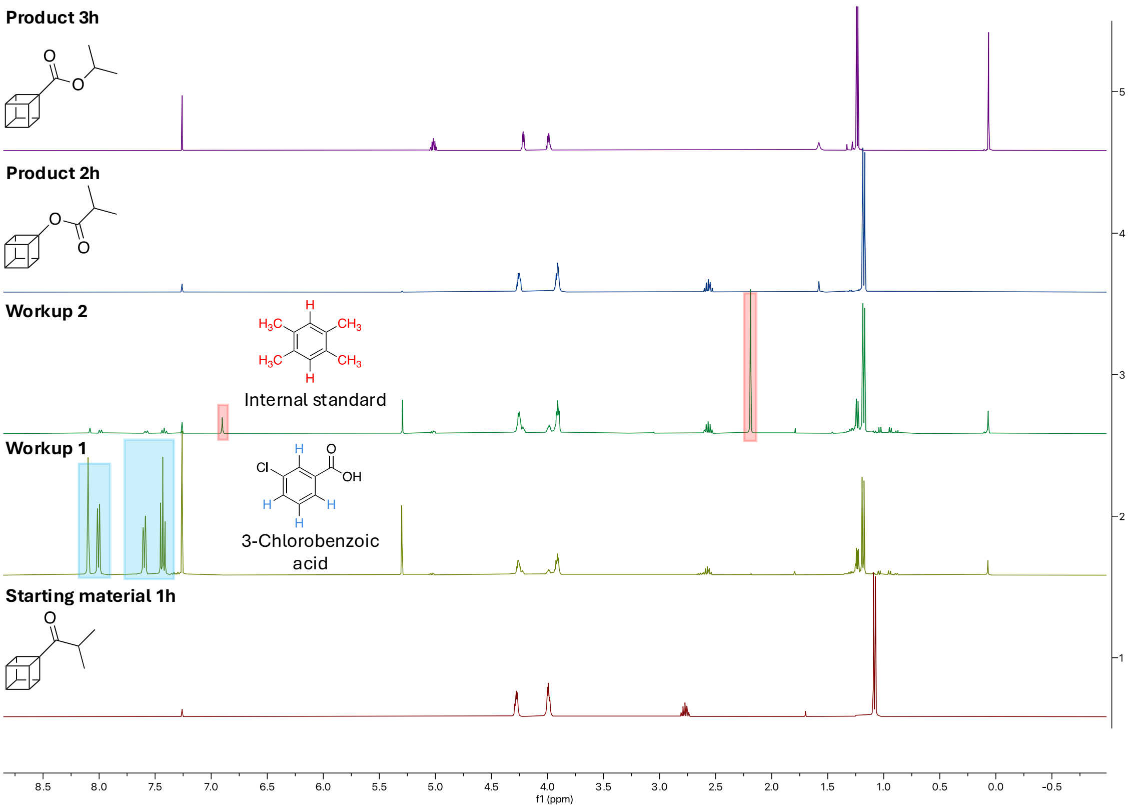

Baeyer-Villiger of 1-(4-(methoxymethyl)cuban-1-yl)-2-methylpropan-1-one (1i)

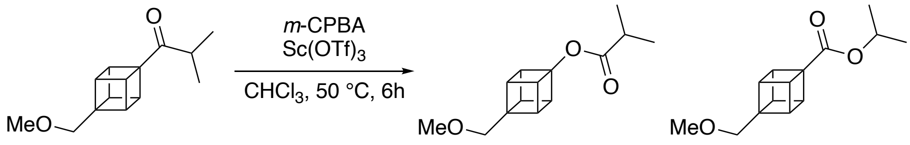

The reaction was performed using the **general procedure** with 1-(4-(methoxymethyl)cuban-1-yl)-2-methylpropan-1-one (**1i**) (44 mg, 1.0 eq., 0.20 mmol), scandium (III) triflate (9.9 mg, 0.1 eq., 0.02 mmol), 3-chloroperbenzoic acid (173 mg, 4.0 equiv., 0.73 mmol) and $CHCl_3$ (2.0 mL). Purification by silica gel column chromatography (07:93 EtOAc/petroleum ether) gave the title compound **2i** (20 mg, 42 %) as a white solid and **3i** (4 mg, 8 %) as a white solid.

2i: **^{1}H NMR** (500 MHz, $CDCl_3$) δ 4.19 – 4.13 (m, 3H), 3.81 – 3.74 (m, 3H), 3.54 (s, 2H), 3.37 (s, 3H), 2.56 (hept, J = 6.9 Hz, 1H), 1.18 (d, J = 6.9 Hz, 6H); **^{13}C NMR** (126 MHz, $CDCl_3$) δ 175.8, 88.5, 73.6, 59.4, 57.4, 52.0, 42.1, 33.9, 19.0; **HRMS (ESI$^+$)** m/z: $[M+H]^+$ Calcd. for $C_{14}H_{19}O_3$ 235.1329; Found 235.1326.

3i: **^{1}H NMR** (500 MHz, $CDCl_3$) δ 5.02 (hept, *J* = 6.3 Hz, 1H), 4.15 – 4.09 (m, 3H), 3.89 – 3.84 (m, 3H), 3.53 (s, 2H), 3.38 (s, 3H), 1.24 (d, *J* = 6.3 Hz, 6H); **^{13}C NMR** (126 MHz, $CDCl_3$) δ 172.2, 73.3, 67.5, 59.4, 57.6, 56.7, 46.7, 45.2, 22.0; **HRMS (EI^{+})** m/z: $[M-C_3H_7]^+$ Calcd. for $C_{11}H_{11}O_3$ 191.0703; Found 191.0701.

NMR yield of **1i** / %	NMR yield of **2i** / %	NMR yield of **3i** / %	Isolated yield of **1i** / %	Isolated yield of **2i** / %	Isolated yield of **3i** / %
0	44	15	0	42	8

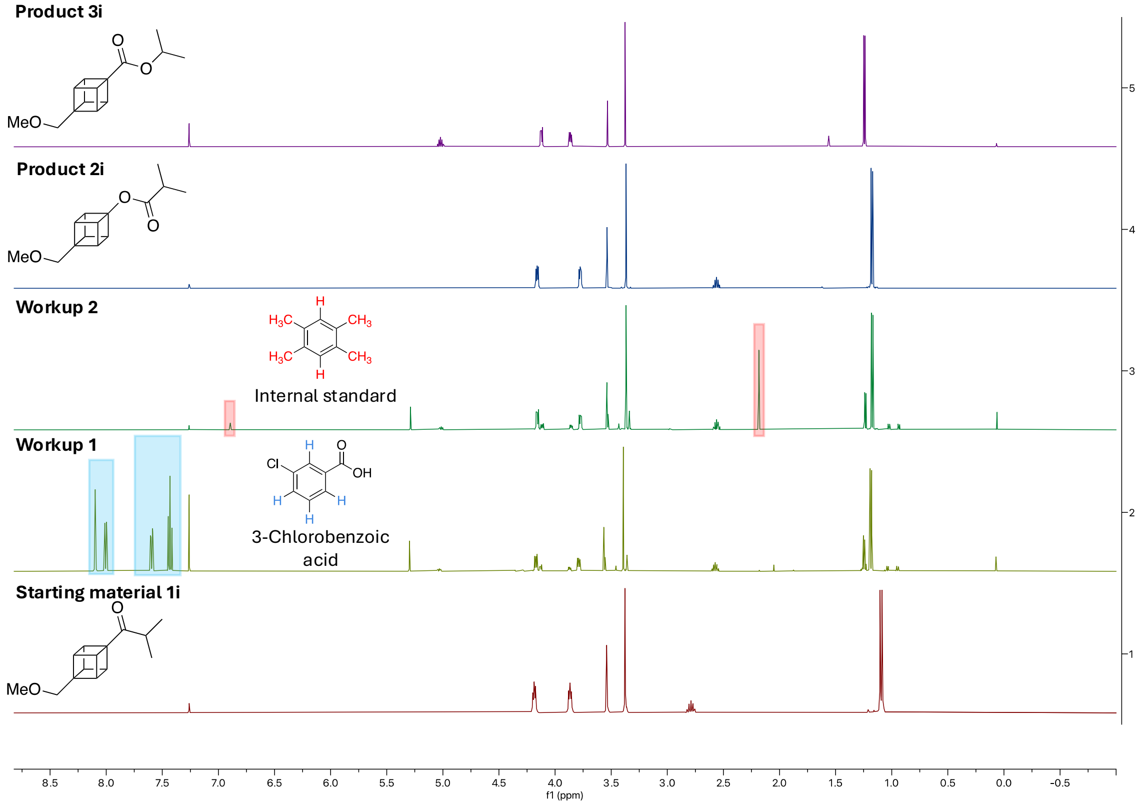

Baeyer-Villiger of 4-isobutyryl-*N*,*N*-diisopropylcubane-1-carboxamide (1j)

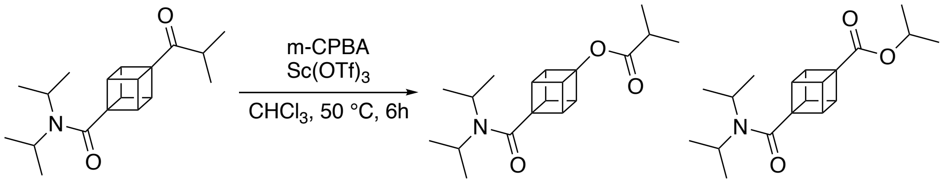

1j **2j** **3j**

The reaction was performed using the **general procedure** with 4-isobutyryl-N,N-diisopropylcubane-1-carboxamide (**1j**) (61 mg, 1.0 eq., 0.20 mmol), scandium (III) triflate (9.7 mg, 0.1 eq., 0.02 mmol), 3-chloroperbenzoic acid (175 mg, 4.0 equiv., 0.73 mmol) and $CHCl_3$

(2.0 mL). Purification by silica gel column chromatography (15:75 EtOAc/petroleum ether) separated **2j** from **3j**, but both compounds were still impure**.** Further purification of **2j** by silica gel column chromatography (15:75 EtOAc/toluene) gave the title compound **2j** (14 mg, 22 %) as a white solid. Further purification of **3j** by silica gel column chromatography (15:75 EtOAc/toluene) gave the title compound **3j** (11 mg, 22 %) as a white solid.

2j: **^{1}H NMR** (500 MHz, $CDCl_3$) δ 4.23 – 4.18 (m, 3H), 4.11 – 4.05 (m, 3H), 3.47 (hept, J = 6.6 Hz, 1H), 3.29 (hept, J = 6.7 Hz, 1H), 2.57 (hept, J = 7.0 Hz, 1H), 1.41 (d, J = 6.7 Hz, 6H), 1.19 (d, J = 6.6 Hz, 6H), 1.18 (d, J = 7.0 Hz, 6H); **^{13}C NMR** (126 MHz, $CDCl_3$) δ 176.0, 170.9, 86.8, 59.3, 51.6, 48.4, 46.0, 44.1, 33.9, 21.1, 20.6, 19.0; **HRMS (ESI$^+$)** m/z: $[M+H]^+$ Calcd. for $C_{19}H_{28}NO_3$ 318.2064; Found 318.2066.

3j: **^{1}H NMR** (500 MHz, $CDCl_3$) δ 5.02 (hept, J = 6.3 Hz, 1H), 4.17 (s, 6H), 3.47 (hept, J = 6.6 Hz, 1H), 3.30 (hept, J = 6.7 Hz, 1H), 1.41 (d, J = 6.7 Hz, 6H), 1.24 (d, J = 6.3 Hz, 6H), 1.20 (d, J = 6.6 Hz, 6H); **^{13}C NMR** (126 MHz, $CDCl_3$) δ 171.7, 170.4, 67.7, 59.4, 55.1, 48.6, 47.0, 46.3, 46.0, 22.0, 21.1, 20.6; **HRMS (ESI$^+$)** m/z: $[M+H]^+$ Calcd. for $C_{19}H_{28}NO_3$ 318.2069; Found 318.2065.

NMR yield of **1j** / %	NMR yield of **2j** / %	NMR yield of **3j** / %	Isolated yield of **1j** / %	Isolated yield of **2j** / %	Isolated yield of **3j** / %
0	25	29	0	22	9

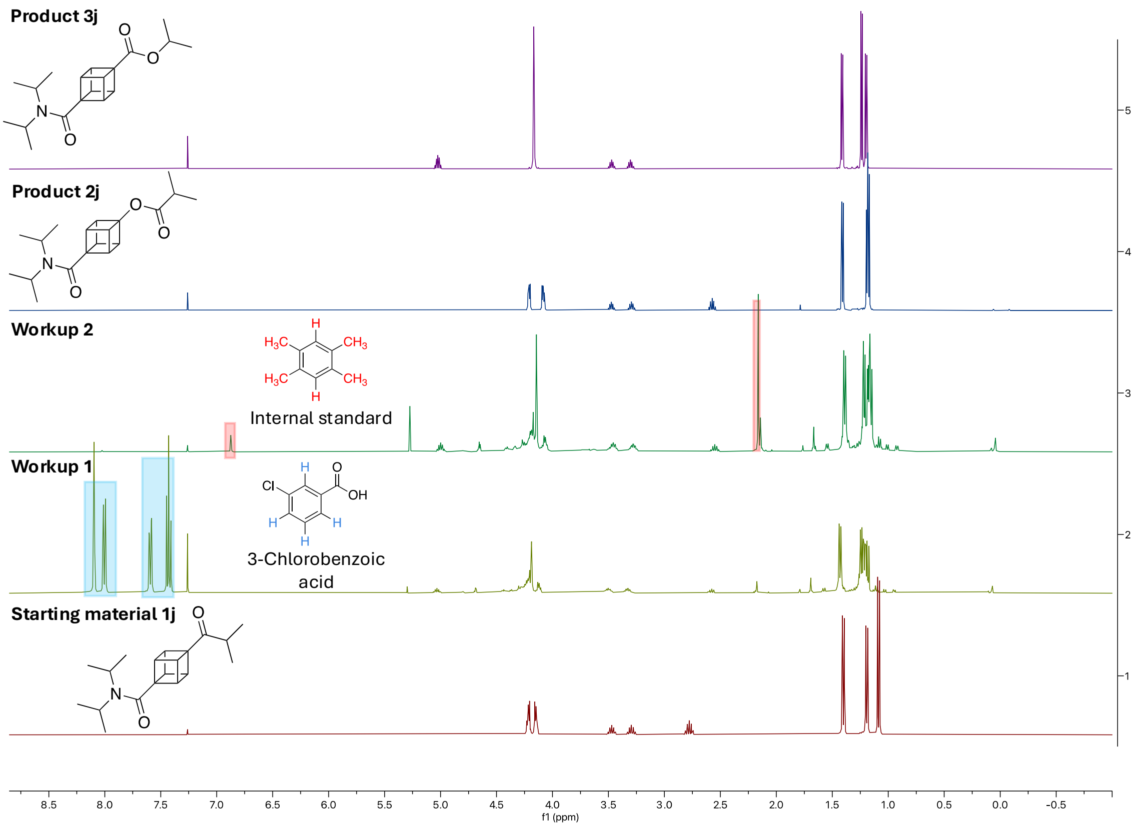

Baeyer-Villiger of 1-(4-bromocuban-1-yl)-2-methylpropan-1-one (1k)

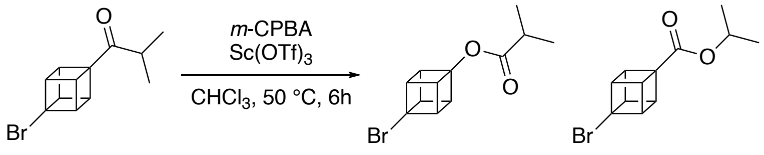

The reaction was performed using the **general procedure** with 1-(4-bromocuban-1-yl)-2-methylpropan-1-one (**1k**) (51 mg, 1.0 eq., 0.20 mmol), scandium (III) triflate (9.8 mg, 0.1 eq., 0.02 mmol), 3-chloroperbenzoic acid (173 mg, 4.0 equiv., 0.73 mmol) and $CHCl_3$ (2.0 mL). Purification by silica gel column chromatography (2:98 EtOAc/petroleum ether) gave an impure **2k** and the title compound **3k** (25 mg, 46 %) as a white solid. Further purification of **2k** by silica gel column chromatography (30:70 DCM/petroleum ether) gave the title compound **2k** (5 mg, 9 %) as a white solid.

2k: **^{1}H NMR** (500 MHz, $CDCl_3$) δ 4.35 – 4.28 (m, 3H), 4.18 – 4.11 (m, 3H), 2.57 (hept, *J* = 7.0 Hz, 1H), 1.18 (d, *J* = 7.0 Hz, 6H); **^{13}C NMR** (126 MHz, $CDCl_3$) δ 175.8, 88.4, 64.6, 52.7, 51.4, 33.9, 18.9; **HRMS (EI^{+})** m/z: $[M\text{-}CH_3]^+$ Calcd. for $C_{11}H_{10}O_2{}^{79}Br$ 252.9859; Found 252.9856.

3k: **^{1}H NMR** (400 MHz, $CDCl_3$) δ 5.02 (hept, *J* = 6.3 Hz, 1H), 4.31 – 4.20 (m, 6H), 1.23 (d, *J* = 6.3 Hz, 6H); **^{13}C NMR** (101 MHz, $CDCl_3$) δ 171.2, 67.9, 63.4, 56.7, 54.7, 47.7, 22.0; **HRMS (EI^{+})** m/z: $[M\text{-}C_3H_7]^+$ Calcd. for $C_9H_6O_2{}^{79}Br$ 224.9545; Found 224.9539.

NMR yield of **1k** / %	NMR yield of **2k** / %	NMR yield of **3k** / %	Isolated yield of **1k** / %	Isolated yield of **2k** / %	Isolated yield of **3k** / %
0	12	50	0	9	46

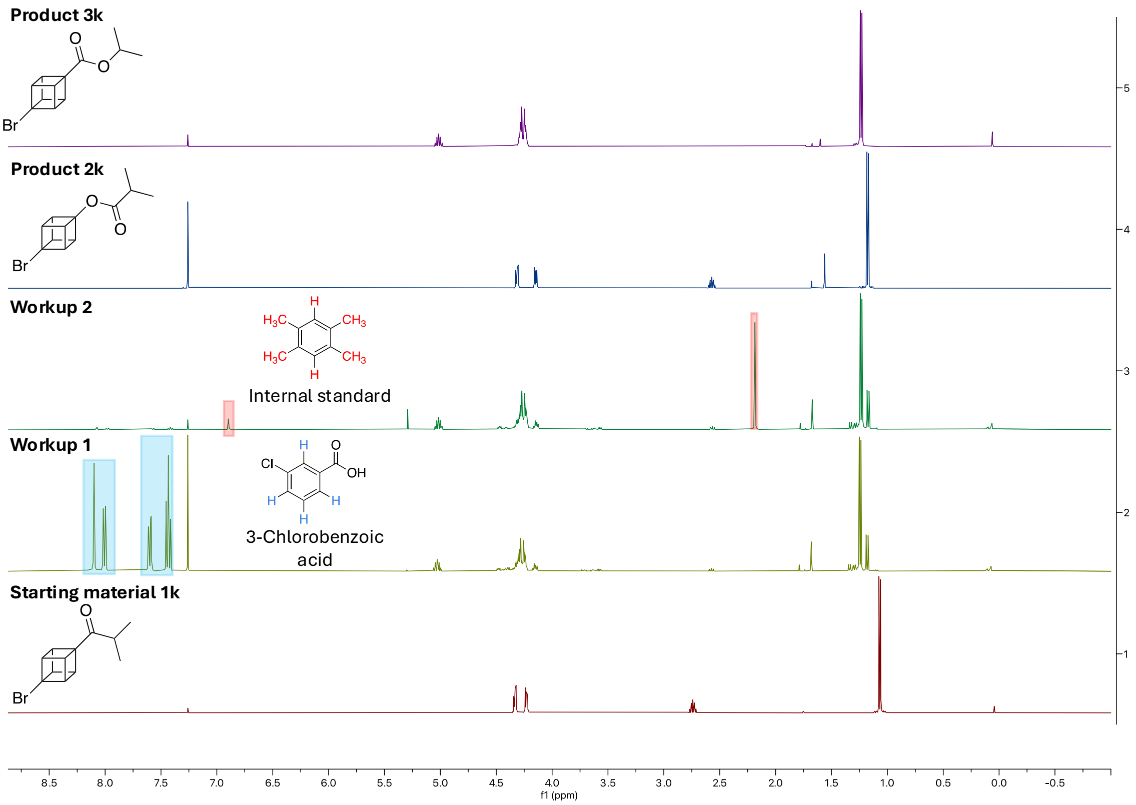

Baeyer-Villiger of 1-(4-fluorocuban-1-yl)-2-methylpropan-1-one (1l)

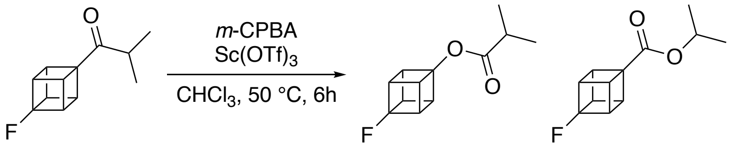

1l **2l** **3l**

The reaction was performed using the **general procedure** with 1-(4-fluorocuban-1-yl)-2-methylpropan-1-one (**1l**) (39 mg, 1.0 eq., 0.20 mmol), scandium (III) triflate (10.3 mg, 0.1 eq., 0.02 mmol), 3-chloroperbenzoic acid (171 mg, 4.0 equiv., 0.73 mmol) and $CHCl_3$ (2.0 mL). Purification by silica gel column chromatography (02:98 EtOAc/petroleum ether) gave the title compound **2l** (4 mg, 10 %) as a white solid and **3l** (16 mg, 39 %) as a white solid.

2l: **^{1}H NMR** (500 MHz, $CDCl_3$) δ 4.26 – 4.17 (m, 3H), 4.10 – 4.03 (m, 3H), 2.57 (hept, *J* = 6.9 Hz, 1H), 1.18 (d, *J* = 6.9 Hz, 6H). **^{19}F{^{1}H}NMR** (471 MHz, $CDCl_3$) δ -142.94. **^{13}C NMR** (126 MHz, $CDCl_3$) δ 175.7, 104.1 (d, *J* = 322.5 Hz), 89.0 (d, *J* = 21.7 Hz), 50.8 (d, *J* = 25.8 Hz), 47.4 (d, *J* = 6.5 Hz), 33.9, 18.9; **HRMS (EI$^+$)** m/z: $[M\text{-}CH_3]^+$ Calcd. for $C_{11}H_{10}O_2F$ 193.0659; Found 193.0657.

3l: **^{1}H NMR** (400 MHz, $CDCl_3$) δ 5.02 (hept, *J* = 6.3 Hz, 1H), 4.36 – 4.25 (m, 3H), 4.08 – 3.97 (m, 3H), 1.24 (d, *J* = 6.3 Hz, 6H); **^{19}F{^{1}H}NMR** (376 MHz, $CDCl_3$) δ -140.42; **^{13}C NMR** (101 MHz,

CDCl₃) δ 171.8 (d, J = 7.2 Hz), 103.1 (d, J = 328.0 Hz), 67.8, 57.1 (d, J = 13.8 Hz), 54.1 (d, J = 25.3 Hz), 42.3 (d, J = 5.6 Hz), 22.0; **HRMS (EI⁺)** m/z: $[M-C_3H_7]^+$ Calcd. for $C_9H_6O_2F$ 165.0346; Found 165.0344.

NMR yield of **1l** / %	NMR yield of **2l** / %	NMR yield of **3l** / %	Isolated yield of **1l** / %	Isolated yield of **2l** / %	Isolated yield of **3l** / %
0	11	49	0	10	39

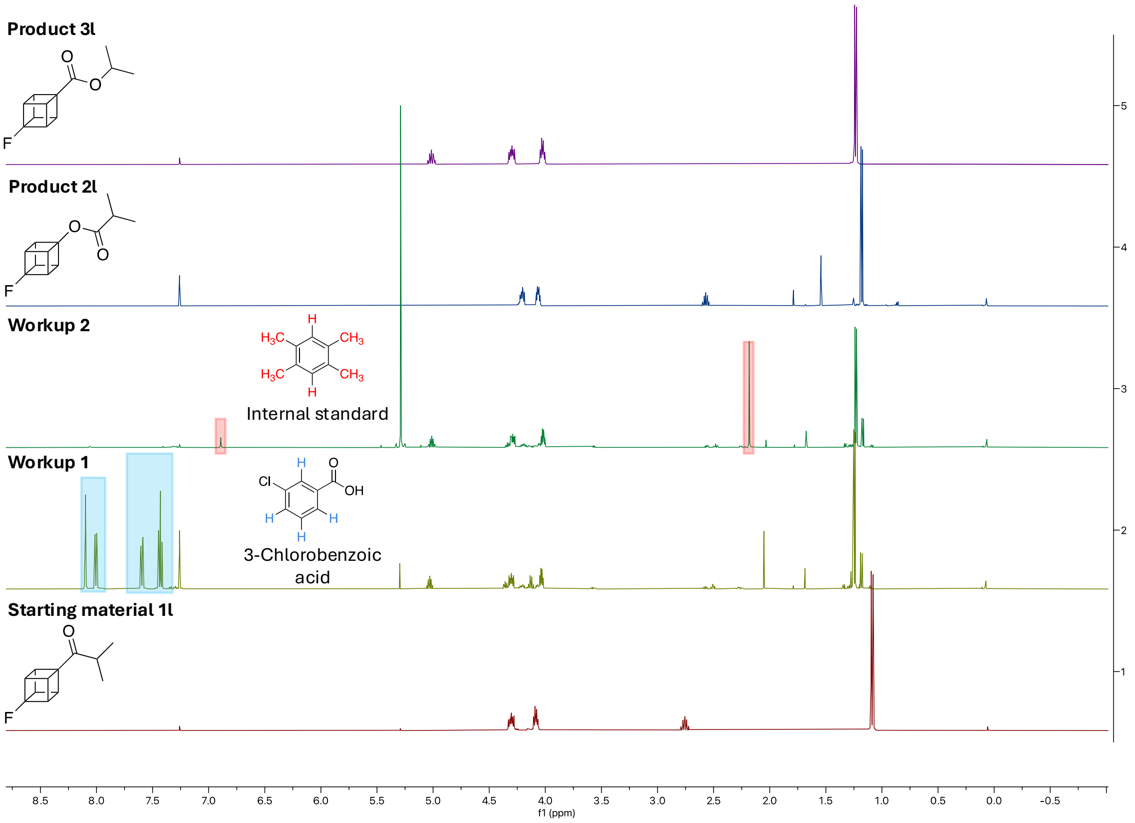

Cubanol screening

Table S3: Synthesis of cubanol **4b** under basic conditions

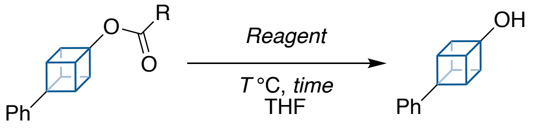

Entry	R	Reagent / equiv	Temp / °C	Time / h	Conversion of 4a to 4b[a] / %	Major products in crude ^{1}H NMR
**1**	Ph	DIBAL / 1.1	-78	2	0	**4a**
**2**	Ph	DIBAL / 1.1	rt	2	0	**4a** + Decomposition
**3**	H	DIBAL / 1.5	-78	2	0	Decomposition
**4**	Ph	$LiAlH_4$ / 2.0	0	0.25	0	Decomposition
**5**[b]	Ph	LiOH / 2.5	rt	24	0	**4a** + Decomposition

[a] Conversion calculated by integration of relevant peaks in ^{1}H NMR of the crude material. [b] Solvent 3:3:1 ratio of THF/MeOH/H_2O

Table S4: Synthesis cubanol **4b** under acidic conditions.

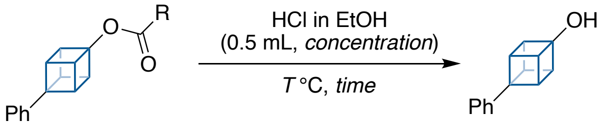

Entry	R	*Conc. of HCl in EtOH / M*	Temp / °C	Time / h	Conversion of 4a to 4b[a] / %	Major products in crude ^{1}H NMR
**1**	Me	0.001	78	3	0	**4a** + Decomposition
**2**	Me	0.001	78	6	0	Decomposition
**3**[b]	Me	1.25	rt	3	100	**4b**
**4**	Me	1.25	rt	18	100	**4b**
**5**	Ph	1.25	rt	18	6	**4a + 4b**

[a] Conversion calculated by integration of relevant peaks in ^{1}H NMR of the crude material. [b] Optimised conditions.

Synthesis of phenylcuban-1-ol (4b)

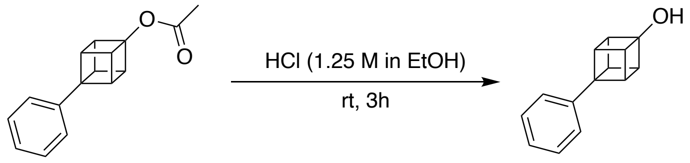

2b **4b**

4-Phenylcuban-1-yl acetate (**2b**) (10 mg, 1.0 equiv., 0.04 mmol) in a solution of anhydrous HCl in ethanol (0.5 mL, 1.25 M) was stirred at rt for 3 h. The reaction mixture was concentrated *in vacuo* to remove bulk of the ethanol to afford a white solid. ^{1}H NMR and HRMS analysis was performed to confirm formation of **4b**.

^{1}H NMR (300 MHz, $CDCl_3$) δ 7.39 – 7.31 (m, 2H), 7.24 – 7.16 (m, 3H), 4.08 – 3.98 (m, 3H), 3.96 – 3.87 (m, 3H); **^{1}H NMR** (400 MHz, acetic acid-d_3) δ 7.35 – 7.28 (m, 2H), 7.22 – 7.11 (m, 3H), 4.08 – 3.98 (m, 3H), 3.95 – 3.84 (m, 3H).). **HRMS (EI^{+})** m/z: $[M]^{+\bullet}$ Calcd. for $C_{14}H_{12}O$ 196.0882; Found 196.0881.

Synthesis of cubyl-resveratrol

The synthesis of cubyl-resveratrol followed the general synthetic methodology reported by Yi Ling Goh and co-workers for the synthesis of BCP-resveratrol.[17]

Synthetic of intermediate 7:

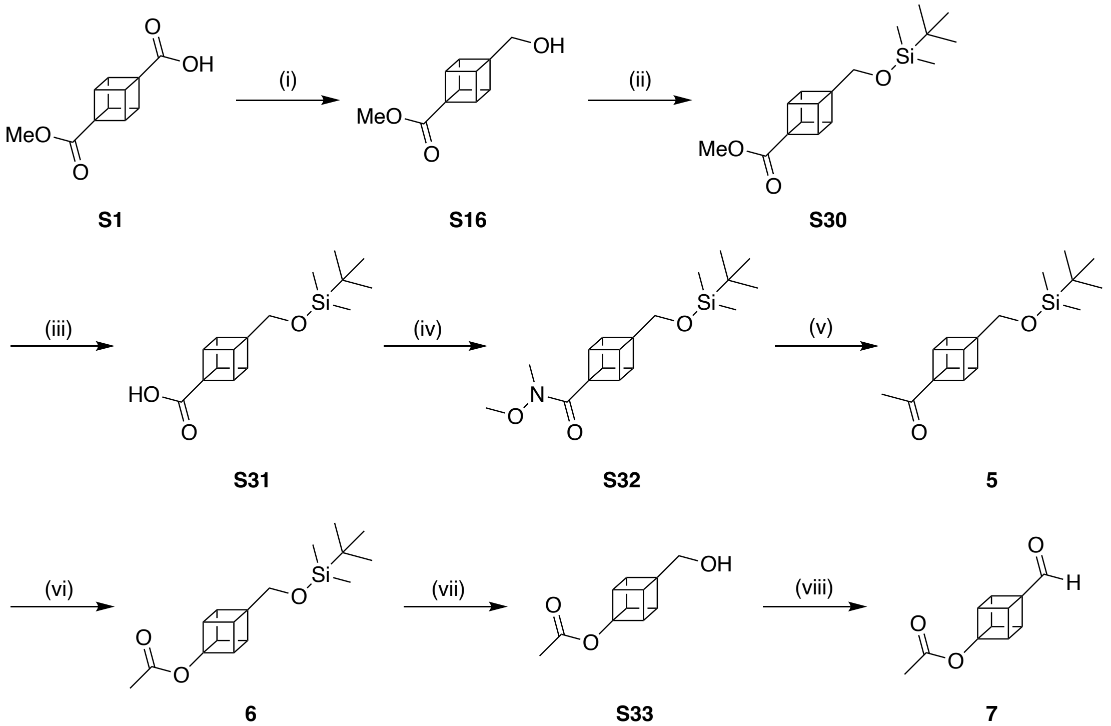

Conditions: (i) $BH_3{\cdot}DMS$ (1 M in 2-MeTHF), THF, 0 °C → rt, 6 h, 98 % (ii) Imidazole, TBSCl, DCM, rt, 3 h, 92 % (iii) LiOH, THF/MeOH/H_2O (3:1:1), rt, 3 h, 93 % (iv) HATU, DIPEA, $CH_3ONHCH_3{\cdot}HCl$, DMF, rt, 1.5 h, 89 % (v) MeMgBr (1 M in THF), THF, 0 °C →rt, 15 min, 88 % (vi) *m*-CPBA, $CHCl_3$, rt, 2 h, 96 % (vii)

TBAF (1 M in THF), THF, rt, 1 h, 98 % (viii) Oxalyl chloride (2 M in DCM), DMSO, DCM, -78 °C, 1.5 h; NEt_3, -78 °C → rt, 15 min, 97 %.

Synthesis of cubyl-resveratrol (8)

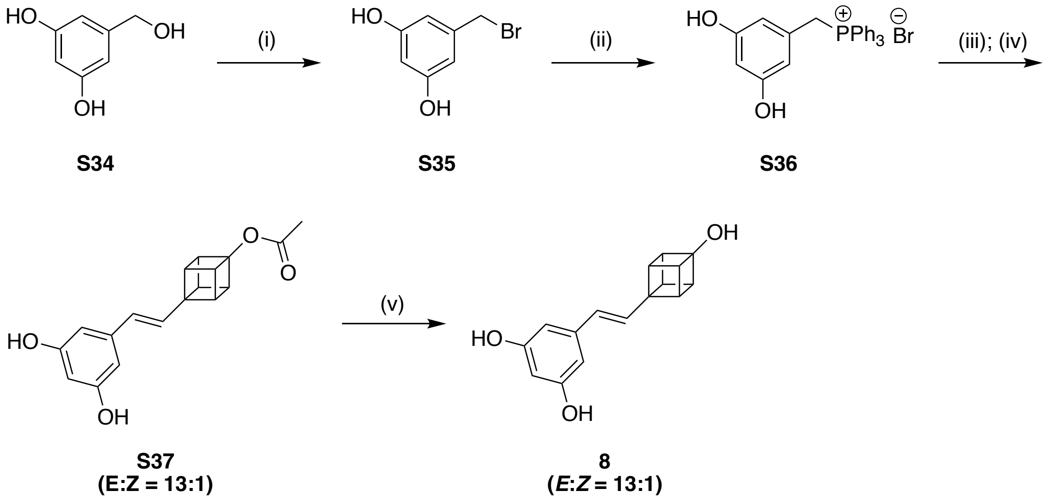

Conditions: (i) CBr_4, PPh_3, THF, rt, 16 h (ii) PPh_3, MeCN, 82 °C, 5 h, 24 % (over two steps) (iii) LiHMDS (1 M in THF), THF – 78 °C → 0 °C, 30 min; (iv) **7**, THF, – 78 °C, 20 min, 34 % (v) HCl (1.25 M in EtOH), rt, 3 h, 38 % (NMR yield).

Experimental:

Methyl-4-(hydroxymethyl)cubane-1-carboxylate (S16)

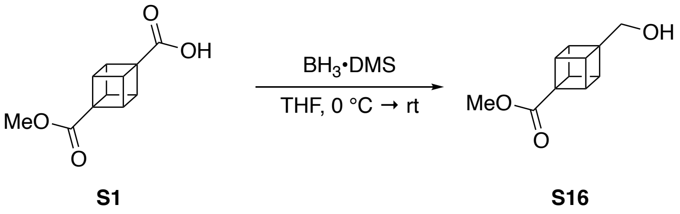

To a solution of the commercially available 4-(methoxycarbonyl)cubane-1-carboxylic acid (**S1**) (1.5 g, 1.0 equiv., 7.27 mmol) in anhydrous THF (30 mL) was added borane dimethyl sulfide (12 mL, 1.7 equiv., 1 M in 2-methyltetrahydrofuran) dropwise at 0 °C. After the addition the mixture was stirred at rt for 6 h. The mixture was quenched with H_2O (10 mL) at 0 °C and diluted with EtOAc (30 mL). The organic layer was washed with sat. $NaHCO_3$ (2 x 20 mL), sat. brine (20 mL), dried with anhydrous $MgSO_4$, filtered, and concentrated *in vacuo* to afford the title compound **S16** (1.37 g, 98 %) as a white solid. **S16** was used directly in the next step without further purification.

^{1}H NMR (400 MHz, $CDCl_3$) δ 4.18 – 4.11 (m, 3H), 3.92 – 3.86 (m, 3H), 3.77 (s, 2H), 3.70 (s, 3H); **HRMS (EI^+)** m/z: $[M-H_2O]^+$ Calcd. for $C_{11}H_{19}O_2$ 174.0675; Found 174.0674. All spectroscopic data were in accordance with the literature.[11]

Methyl-4-(((*tert*-butyldimethylsilyl)oxy)methyl)cubane-1-carboxylate (S30)

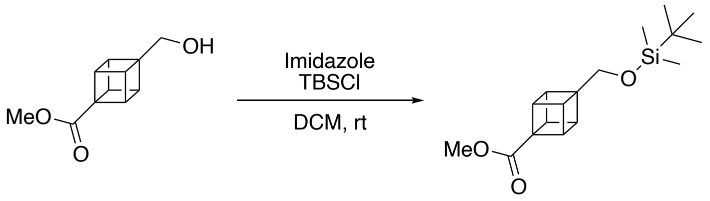

S16 **S30**

To a solution of methyl-4-(hydroxymethyl)cubane-1-carboxylate (**S16**) (1.37 g, 1.0 equiv., 7.13 mmol) in anhydrous DCM (32 mL) was added imidazole (1.2 g, 2.5 equiv., 17.8 mmol) in one portion at rt. After 10 minutes *tert*-butyldimethylsilyl chloride (TBSCl) (1.3 g, 1.2 equiv., 8.56 mmol) was added in one portion and the cloudy white mixture was stirred at rt for 3 hours. The mixture was quenched with H_2O (5 mL) and the organic layer was washed with H_2O (3 x 15 mL), dried with anhydrous $MgSO_4$, filtered and concentrated *in vacuo* to afford the crude. Purification by silica gel column chromatography (05:95 EtOAc: petroleum ether) gave the title compound **S30** (2.0 g, 92 %) as a colourless oil.

^{1}H NMR (400 MHz, $CDCl_3$) δ 4.14 – 4.07 (m, 3H), 3.85 – 3.79 (m, 3H), 3.73 (s, 2H), 3.70 (s, 3H), 0.88 (s, 9H), 0.04 (s, 6H); **^{13}C NMR** (101 MHz, $CDCl_3$) δ 173.0, 63.6, 59.1, 56.5, 51.6, 46.5, 44.8, 26.0, 18.6, -5.0; **HRMS (ESI$^+$)** m/z: $[M+H]^+$ Calcd. for $C_{17}H_{27}O_3Si$ 307.1724; Found 307.1730.

4-(((*tert*-Butyldimethylsilyl)oxy)methyl)cubane-1-carboxylic acid (S31)

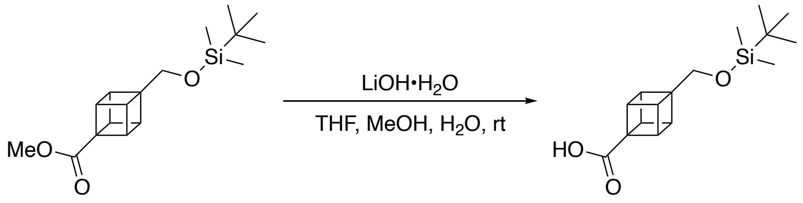

S30 **S31**

To a solution of methyl-4-(((*tert*-butyldimethylsilyl)oxy)methyl)cubane-1-carboxylate (**S30**) (2.0 g, 1.0 equiv., 6.56 mmol) in 3:3:1 ratio of THF:MeOH:H_2O (13 mL) was added lithium hydroxide monohydrate (302 mg, 1.1 equiv., 7.22 mmol) in one-portion at rt. The mixture was allowed to stir at rt for 3 hours. The mixture was diluted with 1 M NaOH (2 mL) and the aqueous layer was washed with EtOAc (1 x 20 mL). The aqueous layer was acidified with 2 M HCl and the aqueous layer was extracted with EtOAc (3 x 20 mL). The combined organic layers were washed sat. brine (10 mL), dried with anhydrous $MgSO_4$, filtered and concentrated *in vacuo* to afford the title compound **S31** (1.77 g, 93%) as a white solid. **S31** was used directly in the next step without further purification.

^{1}H NMR (400 MHz, $CDCl_3$) δ 11.36 (Br s, 1H), 4.18 – 4.12 (m, 3H), 3.89 – 3.81 (m, 3H), 3.74 (s, 2H), 0.88 (s, 9H), 0.05 (s, 6H); **^{13}C NMR** (101 MHz, $CDCl_3$) δ 178.6, 63.5, 59.2, 56.2, 46.5, 44.9, 26.0, 18.6, -5.0; **HRMS (ESI$^-$)** m/z: $[M-H]^-$ Calcd. for $C_{16}H_{23}O_3Si$ 291.1421; Found 291.1409.

4-(((*tert*-Butyldimethylsilyl)oxy)methyl)-*N*-methoxy-*N*-methylcubane-1-carboxamide (S32)

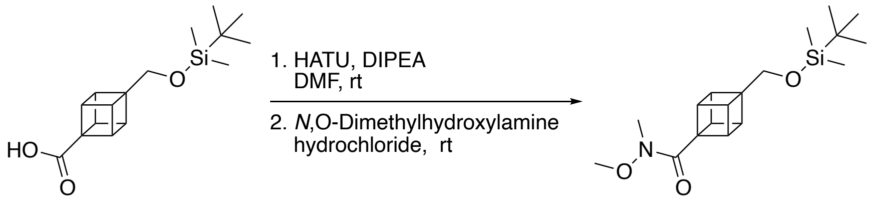

S31 **S32**

To a solution of 4-(((*tert*-butyldimethylsilyl)oxy)methyl)cubane-1-carboxylic acid (**S31**) (1.77 g, 1.0 equiv., 6.07 mmol) and HATU (2.77 g, 1.2 equiv., 7.29 mmol) in anhydrous DMF (30 mL) was added *N*,*N*-diisopropylethylamine (DIPEA) (3.6 mL, 3.5 equiv., 21.2 mmol) at rt. After 5 minutes, *N*,O-dimethylhydroxylamine hydrochloride (770 mg, 1.3 equiv., 7.89 mmol) was added in one portion and the reaction mixture was allowed to stir at rt for 1.5 h. The mixture was quenched with H_2O (10 mL) and diluted with EtOAc (20 mL). The organic layer was washed with H_2O (3 x 20 mL), sat. brine (15 mL), dried with anhydrous $MgSO_4$, filtered and concentrated *in vacuo* to afford the crude. Purification by silica gel column chromatography (1:3 EtOAc: petroleum ether) gave the title compound **S32** (1.81 g, 89 %) as a colourless oil.

^{1}H NMR (500 MHz, $CDCl_3$) δ 4.15 – 4.09 (m, 3H), 3.82 – 3.77 (m, 3H), 3.73 (s, 2H), 3.70 (s, 3H), 3.17 (s, 3H), 0.88 (s, 9H), 0.04 (s, 6H); **^{13}C NMR** (126 MHz, $CDCl_3$) δ 174.0, 63.7, 61.7, 58.5, 58.3, 46.6, 44.7, 32.8, 26.0, 18.5, -5.1; **HRMS (ESI$^+$)** m/z: $[M+H]^+$ Calcd. for $C_{18}H_{30}O_3NSi$ 336.1989; Found 336.2002.

1-(4-(((*tert*-Butyldimethylsilyl)oxy)methyl)cuban-1-yl)ethan-1-one (5)

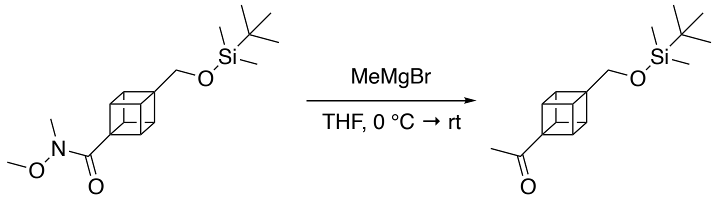

S32 **5**

To a solution of 4-(((*tert*-butyldimethylsilyl)oxy)methyl)-*N*-methoxy-*N*-methylcubane-1-carboxamide (**S32**) (1.81 g, 1.0 equiv., 5.40 mmol) in anhydrous THF (20 mL) at 0 °C was added methylmagnesium bromide (10.8 mL, 2.0 equiv., 1 M in THF) dropwise. The mixture was stirred at 0 °C for 5 minutes and allowed to warm to rt. After 15 minutes, the mixture was quenched with 1 M HCl (5 mL) at 0 °C. The aqueous was extracted with EtOAc (3 x 10 mL) and the combined organic layers were washed with sat. brine (10 mL), dried with anhydrous $MgSO_4$, filtered and concentrated *in vacuo* to afford the crude. Purification by silica gel column chromatography (07:93 EtOAc: petroleum ether) gave the title compound **5** (1.34 g, 88 %) as a colourless oil.

^{1}**H NMR** (400 MHz, $CDCl_3$) δ 4.17 – 4.07 (m, 3H), 3.83 – 3.76 (m, 3H), 3.73 (s, 2H), 2.12 (s, 3H), 0.88 (s, 9H), 0.05 (s, 6H); ^{13}**C NMR** (101 MHz, $CDCl_3$) δ 206.9, 64.4, 63.5, 59.4, 46.6, 44.3, 26.0, 24.8, 18.5, -5.1; **HRMS (ESI$^+$)** m/z: $[M+H]^+$ Calcd. for $C_{17}H_{27}O_2Si$ 291.1775; Found 291.1788.

4-(((*tert*-Butyldimethylsilyl)oxy)methyl)cuban-1-yl acetate (6)

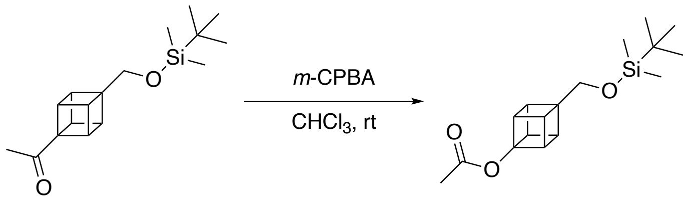

5 **6**

To a solution of 1-(4-(((*tert*-butyldimethylsilyl)oxy)methyl)cuban-1-yl)ethan-1-one (**5**) (580 mg, 1.0 equiv., 2.0 mmol) in anhydrous chloroform (20 mL) was added 3-chloroperbenzoic acid (1.73 g, 4.0 equiv., 9 mmol) in one portion at 0 °C. After 5 minutes, the reaction mixture was stirred at rt for 2 hours and quenched with sodium bisulfite (10 mL). The organic layer was washed further with sodium bisulfite (10 mL), sat. $NaHCO_3$ (2 x 10 mL), dried with anhydrous $MgSO_4$, filtered and concentrated *in vacuo* to afford the title compound **6** (585 mg, 96 %) as a colourless oil. **6** was used in the next step without further purification.

^{1}**H NMR** (500 MHz, $CDCl_3$) δ 4.16 – 4.10 (m, 3H), 3.75 – 3.70 (m, 3H), 3.73 (s, 2H), 2.08 (s, 3H), 0.88 (s, 9H), 0.04 (s, 6H); ^{13}**C NMR** (126 MHz, $CDCl_3$) δ 169.5, 88.7, 63.9, 58.9, 51.7, 41.7, 26.0, 21.2, 18.6, -5.1; **HRMS (ESI$^+$)** m/z: $[M+H]^+$ Calcd. for $C_{17}H_{27}O_3Si$ 307.1724; Found 307.1736.

4-(Hydroxymethyl)cuban-1-yl acetate (S33)

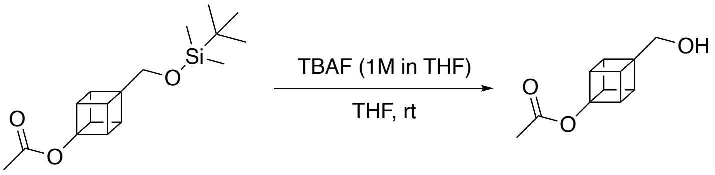

6 **S33**

To a solution of 4-(((*tert*-butyldimethylsilyl)oxy)methyl)cuban-1-yl acetate (**6**) (585 mg, 1.0 equiv., 1.91 mmol) in anhydrous THF (9.5 mL) was added TBAF (2.5 mL, 1.3 equiv., 1 M in THF) dropwise at rt. After 1 hour, the reaction mixture was quenched with H_2O (3 mL) and diluted with EtOAc (30 mL). The organic layer was washed with sat. $NaHCO_3$ (2 x 10 mL), sat. brine (20 mL), dried with anhydrous $MgSO_4$, filtered and concentrated *in vacuo* to afford the crude. Purification by silica gel column chromatography (4:6 EtOAc: petroleum ether) gave the title compound **S33** (357 mg, 98 %) as a colourless oil.

^{1}**H NMR** (400 MHz, $CDCl_3$) δ 4.19 – 4.11 (m, 3H), 3.80 – 3.73 (m, 3H), 3.76 (s, 2H) 2.07 (s, 3H), 1.94 (br s, 1H); ^{13}**C NMR** (101 MHz, $CDCl_3$) δ 169.6, 88.5, 63.7, 58.7, 51.7, 41.5, 21.1; **HRMS (EI$^+$)** m/z: $[(M - {}^\bullet C(O)OCH_3]^+$ Calcd. for C_9H_9O 133.0648; Found 133.0647.

4-Formylcuban-1-yl acetate (7)

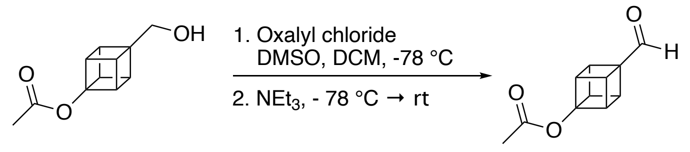

S33 **7**

DMSO (0.37 mL, 2.8 equiv., 5.27 mmol) was added to a solution of oxalyl chloride (1.5 mL, 1.6 equiv., 2 M in DCM) in anhydrous DCM (10 mL) at -78 °C. After 20 minutes a solution of 4-(hydroxymethyl)cuban-1-yl acetate (**S33**) (357 mg, 1.0 equiv., 1.86 mmol) in anhydrous DCM (5 mL) was added dropwise, and the mixture stirred at -78 °C for a further 1.5 hours. Triethylamine (1.45 mL, 5.6 equiv., 10.5 mmol) was then added and the mixture was allowed to warm to rt over 15 minutes. The mixture was quenched with H_2O (10 mL) and the aqueous layer was extracted with DCM (3 x 15 mL). The combined organic layer was washed sat. brine (20 mL), dried with anhydrous $MgSO_4$, filtered and concentrated *in vacuo* to afford the title compound **7** (342 mg, 97%) as a light yellow solid. **7** was used directly in the next step without further purification.

^{1}H NMR (400 MHz, $CDCl_3$) δ 9.75 (s, 1H), 4.25 (s, 6H), 2.07 (s, 3H); **^{13}C NMR** (101 MHz, $CDCl_3$) δ 198.4, 169.4, 87.2, 62.6, 52.5, 43.0, 21.0; **HRMS (EI$^+$)** m/z: $[M-CHO]^+$ Calcd. for $C_{10}H_9O_2$ 161.0597; Found 161.0594.

5-(Bromomethyl)benzene-1,3-diol (S35)

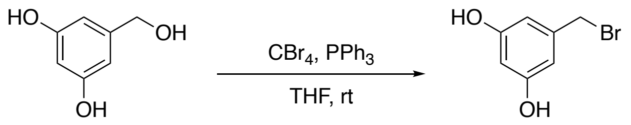

S34 **S35**

To a solution of tetrabromomethane (4.26 g, 1.2 equiv., 12.8 mmol) and triphenylphosphine (3.38 g, 1.2 equiv., 12.8 mmol) in anhydrous THF (20 mL), 3,5-dihydroxybenzyl alcohol (**S34**) (1.50 g, 1.0 equiv., 10.7 mmol) in anhydrous THF (10 mL) was added dropwise at rt. The reaction mixture was stirred at rt for 16 h before being quenched with H_2O (20 mL). The aqueous was extracted with EtOAc (3 x 20 mL) and the combined organic layers were washed with sat. brine (10 mL), dried with anhydrous $MgSO_4$, filtered, and concentrated *in vacuo* to afford the crude. Purification by column chromatography (40:60 EtOAc:petroleum ether) gave a mixture of the title compound **S35** and triphenylphosphine oxide in a 2:1 ratio (2.77 g) as a light-yellow sticky solid. The mixture was used in the next step without further purification. Note: 100 mg of the mixture was purified by column chromatography (40:60 EtOAc:petroleum ether) to afford pure **S35** (56 mg) as a light-yellow solid for full data analysis.

^{1}H NMR (400 MHz, acetone-d_6) δ 8.34 (s, 2H), 6.43 (d, *J* = 2.2 Hz, 2H), 6.30 (t, *J* = 2.2 Hz, 1H), 4.46 (s, 2H); **^{13}C NMR** (101 MHz, acetone-d_6) δ 159.5, 141.0, 108.5, 103.5, 34.6; **HRMS (EI$^+$)**

m/z: $[M]^{+\bullet}$ Calcd. for $C_7H_7O_2{}^{79}Br$ 201.9624; Found 201.9624. All spectroscopic data were in accordance with the literature.[18]

(3,5-Dihydroxybenzyl)triphenylphosphonium bromide (S36)

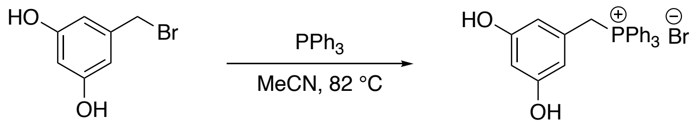

S35 **S36**

To a solution of a 2:1 mixture of **S35** and triphenylphosphine oxide (2.67 g) in anhydrous MeCN (30 mL) was added triphenylphosphine (3.93 g, 15 mmol) in one portion at rt. The reaction mixture was heated to 82 °C for 5 h then concentrated *in vacuo* to remove the MeCN. Et_2O (10 mL) was added to promote the product and triphenylphosphine oxide to precipitate out. The Et_2O was decanted off and the solid residue was washed further with Et_2O (2 x 10 mL). The solid was then washed with DCM (3 x 2 mL) to remove triphenylphosphine oxide impurity. The remaining solid was dried under vacuum to afford the title compound **S36** (1.21 g, 24 % yield over two steps) as a white solid.

^{1}H NMR (500 MHz, DMSO-d_6) δ 9.31 (s, 2H), 7.94 – 7.86 (m, 3H), 7.79 – 7.69 (m, 6H), 7.67 – 7.61 (m, 6H), 6.14 (q, *J* = 2.2 Hz, 1H), 5.85 (t, *J* = 2.2 Hz, 2H), 4.92 (d, *J* = 15.6 Hz, 2H); **^{13}C NMR** (126 MHz, DMSO-d_6) δ 158.5 (d, *J* = 3.1 Hz), 135.0 (d, *J* = 3.0 Hz), 134.0 (d, *J* = 9.8 Hz), 130.0 (d, *J* = 12.4 Hz), 129.4 (d, *J* = 8.4 Hz), 118.1 (d, *J* = 85.6 Hz), 109.0 (d, *J* = 5.7 Hz), 102.4 (d, *J* = 3.7 Hz), 28.2 (d, *J* = 46.7 Hz); **^{31}P{^{1}H}NMR** (162 MHz, DMSO-d_6) δ 22.67; **HRMS (ESI$^+$)** m/z: $[M-Br]^+$ Calcd. for $C_{25}H_{22}O_2P$ 385.1352; Found 385.1359.

4-((*E*)-3,5-dihydroxystyryl)cuban-1-yl acetate (S37)

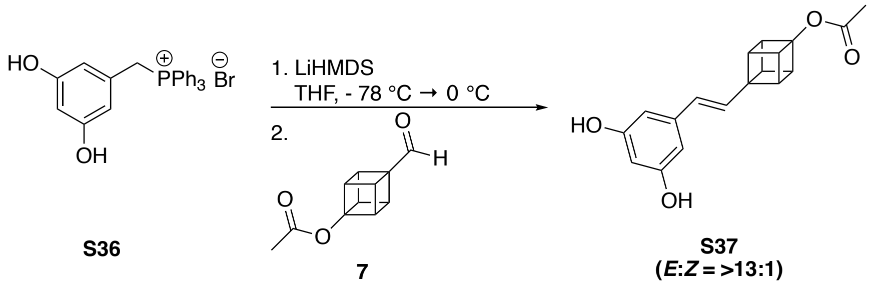

S36 **7** **S37** **(*E*:*Z* = >13:1)**

To a suspension of (3,5-dihydroxybenzyl)triphenylphosphonium bromide (**S36**) (517 mg, 1.4 equiv., 1.11 mmol) in anhydrous THF (5 mL) was added lithium bis(trimethylsilyl)amide (3 mL, 3.8 equiv., 1 M in THF) dropwise at -78 °C. After the addition, the mixture was stirred at 0 °C for 30 minutes in which time the suspension turned red. The reaction mixture was re-cooled to -78 °C and a solution of 4-formylcuban-1-yl acetate (**7**) (150 mg, 1.0 equiv., 0.79 mmol) in anhydrous THF (1 mL) was added dropwise. After stirring at -78 °C for 20 minutes the mixture was quenched with 1 M HCl (2 mL) at -78 °C and allowed to warm to rt. The aqueous was extracted with EtOAc (3 x 10 mL) and the combined organic layers were washed with sat. brine (10 mL), dried with anhydrous $MgSO_4$, filtered and concentrated *in*

vacuo to afford the crude (*E/Z* = >13:1). Purification by silica gel column chromatography (20:80 EtOAc:DCM) gave the title compound **S37** (80 mg, 34 %, *E/Z* = >13:1) as a light-yellow solid.

S37 (*E*-isomer): ^{1}**H NMR** (500 MHz, DMSO-d_6) δ 9.16 (s, 2H), 6.36 (d, *J* = 15.8 Hz, 1H), 6.26 (d, *J* = 2.2 Hz, 2H), 6.13 (d, *J* = 15.8 Hz, 1H), 6.09 (t, *J* = 2.2 Hz, 1H), 4.16 – 4.10 (m, 3H), 3.92 – 3.86 (m, 3H), 2.07 (s, 3H); ^{13}**C NMR** (126 MHz, DMSO-d_6) δ 168.9, 158.4, 138.6, 128.8, 128.3, 104.3, 101.8, 87.4, 58.8, 50.5, 43.9, 20.7; **HRMS (ESI$^+$)** m/z: $[M+H]^+$ Calcd. for $C_{18}H_{17}O_4$ 297.1121; Found 297.1121.

5-((*E*)-2-(4-Hydroxycuban-1-yl)vinyl)benzene-1,3-diol (8)

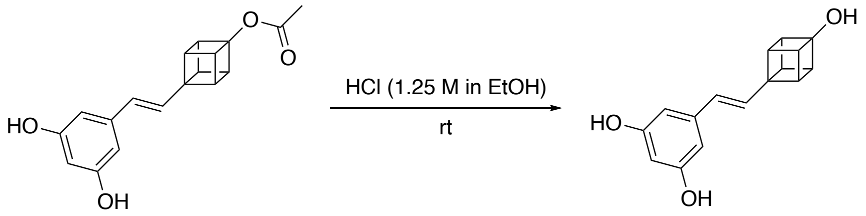

S37
(*E*:*Z* = >13:1)

8
(*E*:*Z* = >13:1)

4-((*E*)-3,5-Dihydroxystyryl)cuban-1-yl acetate (**S37**) (10 mg, 1.0 equiv., 0.04 mmol, *E/Z* = 13:1) in a solution of HCl in ethanol (0.5 mL, 1.25 M) was stirred at rt for 3 h. The reaction mixture was concentrated *in vacuo* to remove bulk of the ethanol to afford a brown sticky solid. The sample was triturated with Et_2O (1 mL) to afford a light brown solid which still contained ethanol. Removal of ethanol was achieved by dissolving the sample in EtOAc (10 mL) and washing the organic layer quickly with sat. NH_4Cl (4 x 5 mL), dried with anhydrous $MgSO_4$, filtered and concentrated *in vacuo* to afford the title compound **8** (3 mg, 32 %, *E/Z* = >13:1) as a light brown solid. NMR yield = 38 % when using durene as internal standard.

^{1}**H NMR** (500 MHz, DMSO-d_6) δ 9.15 (s, 2H), 6.36 (d, *J* = 15.8 Hz, 1H), 6.36 (s, 1H), 6.25 (d, *J* = 2.2 Hz, 2H), 6.08 (t, *J* = 2.2 Hz, 1H), 6.08 (d, *J* = 15.8 Hz, 1H), 3.84 – 3.78 (m, 3H), 3.73 – 3.67 (m, 3H); ^{13}**C NMR** (126 MHz, DMSO-d_6) δ 158.4, 138.7, 129.6, 127.7, 104.2, 101.7, 86.7, 60.0, 51.8, 42.1; **HRMS (ESI$^-$)** m/z: $[M-H]^-$ Calcd. for $C_{16}H_{13}O_3$ 253.0870; Found 253.0863.

Computational Details

DFT calculations were carried out at PBE0/def2-TZVP-D3BJ level[19,20,21] in CPCM model of $CHCl_3$,[22] using Orca v6.0.[5] All species were fully optimised without any symmetry constraints, and confirmed as minima through harmonic frequency calculation, from which enthalpy and entropy corrections were extracted.

Optimised Cartesian Coordinate

[cubane]$^{+}$

```
  C     -0.644726    1.043429   -1.176080
  H     -0.339143    1.724583   -1.960549
  C     -1.446034    1.433046    0.112495
  H     -1.731704    2.454686    0.340678
  C     -0.524536    0.580324    1.050464
  H     -0.129630    0.899951    2.006798
  C      0.102230    0.165969   -0.234175
  C     -0.611742   -1.119951   -0.463994
  H     -0.279928   -2.125599   -0.690479
  C     -1.532329   -0.233651   -1.370859
  H     -1.890740   -0.558059   -2.342334
  C     -2.333793    0.173353   -0.101834
  C     -1.414463   -0.687693    0.810533
  H     -1.674096   -1.379416    1.605248
  H     -3.415587    0.176579   -0.042316
```

[4F-cubane]+

```
  C     -0.629255    1.047692   -1.181835
  H     -0.304056    1.724319   -1.962056
  C     -1.428486    1.443511    0.113649
  H     -1.735689    2.458860    0.341982
  C     -0.508463    0.581814    1.055523
  H     -0.093813    0.899938    2.003803
  C      0.093104    0.166176   -0.233692
  C     -0.596036   -1.125933   -0.465591
  H     -0.243822   -2.124360   -0.692506
  C     -1.515263   -0.237009   -1.382004
  H     -1.895180   -0.559621   -2.345899
  C     -2.302277    0.172996   -0.103386
  C     -1.396676   -0.695416    0.818023
  H     -1.681610   -1.381998    1.608509
  F     -3.628701    0.176582   -0.030925
```

[3F-cubane]+

```
  C     -0.630525    1.035171   -1.159042
  H     -0.351430    1.672458   -1.990584
```

C	-1.434397	1.431271	0.111860
F	-1.825544	2.665028	0.388971
C	-0.512180	0.579946	1.030529
H	-0.135808	0.838272	2.013951
C	0.115771	0.147102	-0.238756
C	-0.605556	-1.155834	-0.471446
H	-0.258408	-2.154974	-0.697158
C	-1.527832	-0.258036	-1.351511
H	-1.869465	-0.529503	-2.345004
C	-2.340939	0.155148	-0.105150
C	-1.412465	-0.701819	0.782707
H	-1.655572	-1.352848	1.616078
H	-3.421870	0.176169	-0.041849

[2F-cubane]+

C	-0.667148	1.048841	-1.181044
F	-0.287612	1.873679	-2.129482
C	-1.448313	1.413585	0.100015
H	-1.702218	2.427734	0.395250
C	-0.523777	0.536193	1.051812
H	-0.158156	0.945392	1.986454
C	0.135404	0.145850	-0.212893
C	-0.608567	-1.114757	-0.420282
H	-0.315078	-2.110365	-0.732215
C	-1.532886	-0.219291	-1.354226
H	-1.860646	-0.612438	-2.312396
C	-2.335067	0.162349	-0.089651
C	-1.422572	-0.717171	0.845749
H	-1.722296	-1.401580	1.630490
H	-3.417289	0.169528	-0.033984

[4-$SiMe_3$-cubane]+

C	-0.706479	1.047828	-1.157517
H	-0.415786	1.740659	-1.937603
C	-1.512513	1.428377	0.121947
H	-1.787696	2.452134	0.355726
C	-0.606708	0.568209	1.056060
H	-0.238443	0.881607	2.025186
C	0.059141	0.165904	-0.222280
C	-0.681353	-1.111307	-0.466298
H	-0.371263	-2.122873	-0.697823
C	-1.586935	-0.222162	-1.373155
H	-1.922376	-0.539706	-2.355522
C	-2.417794	0.174178	-0.109167
C	-1.488557	-0.692886	0.801330
H	-1.743449	-1.394913	1.589177
Si	-4.307957	0.176591	-0.021691
C	-4.766479	0.746880	1.693440
H	-5.854301	0.765860	1.802590
H	-4.363971	0.074768	2.455477
H	-4.391346	1.754659	1.888453

C	-4.907657	1.357882	-1.334032
H	-4.585498	1.042158	-2.329504
H	-6.000456	1.395851	-1.330857
H	-4.535946	2.370001	-1.155412
C	-4.862055	-1.573803	-0.348004
H	-4.459931	-2.261940	0.399835
H	-5.953113	-1.634004	-0.307525
H	-4.543588	-1.913715	-1.336717

References

[1] F. Toriyama, J. Cornella, L. Wimmer, T.-G. Chen, D. D. Dixon, G. Creech, P. S. Baran, *J. Am. Chem. Soc.* **2016**, *138*, 11132-11135.

[2] S. S. Bernhard, G. M. Locke, S. Plunkett, A. Meindl, K. J. Flanagan, M. O. Senge, *Chem. Eur. J.* **2018**, *24*, 1026-1030.

[3] E. W. Della, N. J. Head, *J. Org. Chem.* **1992**, *57*, 2850-2855.

[4] K. Kulbitski, G. Nisnevich, M. Gandelman, *Adv. Synth. Catal.* **2011**, *353*, 1438-1442.

[5] M. Oi, R. Takita, J. Kanazawa, A. Muranaka, C. Wang, M. Uchiyama, *Chem. Sci.* **2019**, *10*, 6107-6112.

[6] K. Lukin, P. E. Eaton, *J. Am. Chem. Soc.* **1995**, *117*, 7652-7656.

[7] Y. Kato, C. M. Williams, M. Uchiyama, S. Matsubara, *Org. lett.* **2019**, *21*, 473-475.

[8] E. J. Ko, G. P. Savage, C. M. Williams, J. Tsanaktsidis, *Org. Lett.* **2011**, *13*, 1944-1947.

[9] P. E. Eaton, N. Nordari, J. Tsanaktsidis, S. P. Upadhyaya, *Synthesis* **1995**, *1995*, 501-502.

[10] J. Wlochal, R. D. Davies, J. Burton, *Org. lett.* **2014**, *16*, 4094-4097.

[11] K. C. Nicolaou, D. Vourloumis, S. Totokotsopoulos, A. Papakyriakou, H. Karsunky, H. Fernando, J. Gavrilyuk, D. Webb, A. F. Stepan, *ChemMedChem.* **2016**, *11*, 31-37.

[12] D. E. Collin, A. A. Folgueiras-Amador, D. Pletcher, M. E. Light, B. Linclau, R. C. Brown, *Chem. Eur. J.* **2020**, *26*, 374-378.

[13] A. Watanabe, K. Koyamada, K. Miyamoto, J. Kanazawa, M. Uchiyama, *Org. Process Res. Dev.* **2020**, *24*, 1328-1334.

[14] S. S. Kuduva, D. C. Craig, A. Nangia, G. R. Desiraju, *J. Am. Chem. Soc.* **1999**, *121*, 1936-1944.

[15] L. Candish, E. A. Standley, A. Gómez-Suárez, S. Mukherjee, F. Glorius, *Chem. Eur. J.* **2016**, *22*, 9971-9974.

[16] B. A. Chalmers, H. Xing, S. Houston, C. Clark, S. Ghassabian, A. Kuo, B. Cao, A. Reitsma, C. E. P. Murray, J. E. Stok, *Angew. Chem. Int. Ed.* **2016**, *55*, 3580-3585.

[17] Y. L. Goh, Y. T. Cui, V. Pendharkar, V. A. Adsool, *ACS Med. Chem. Lett.* **2017**, *8*, 516-520.

[18] D. S. Harmalkar, Q. Lu, K. Lee, *J. Nat. Prod.* **2018**, *81*, 798-805.

[19] C. Adamo and V. Barone, *J. Chem. Phys.*, **1999**, 110, 6158-69.

[20] F. Weigend and R. Ahlrichs, *Phys. Chem. Chem. Phys.*, **2005**, 7, 3297-305.

[21] A. D. Becke and E. R. Johnson, *J. Chem. Phys.* **2005**, 123, 154101.

[22] V. Barone and M. Cossi, *J. Phys. Chem. A*, **1998**, 102,1995-2001.

[23] Neese, F. Wennmohs, U Becker, C. Riplinger, *J. Chem. Phys.*, **2020**, 152, L224108

NMR Spectra: ^{1}H NMR (500 MHz, CDCl$_3$) of S5

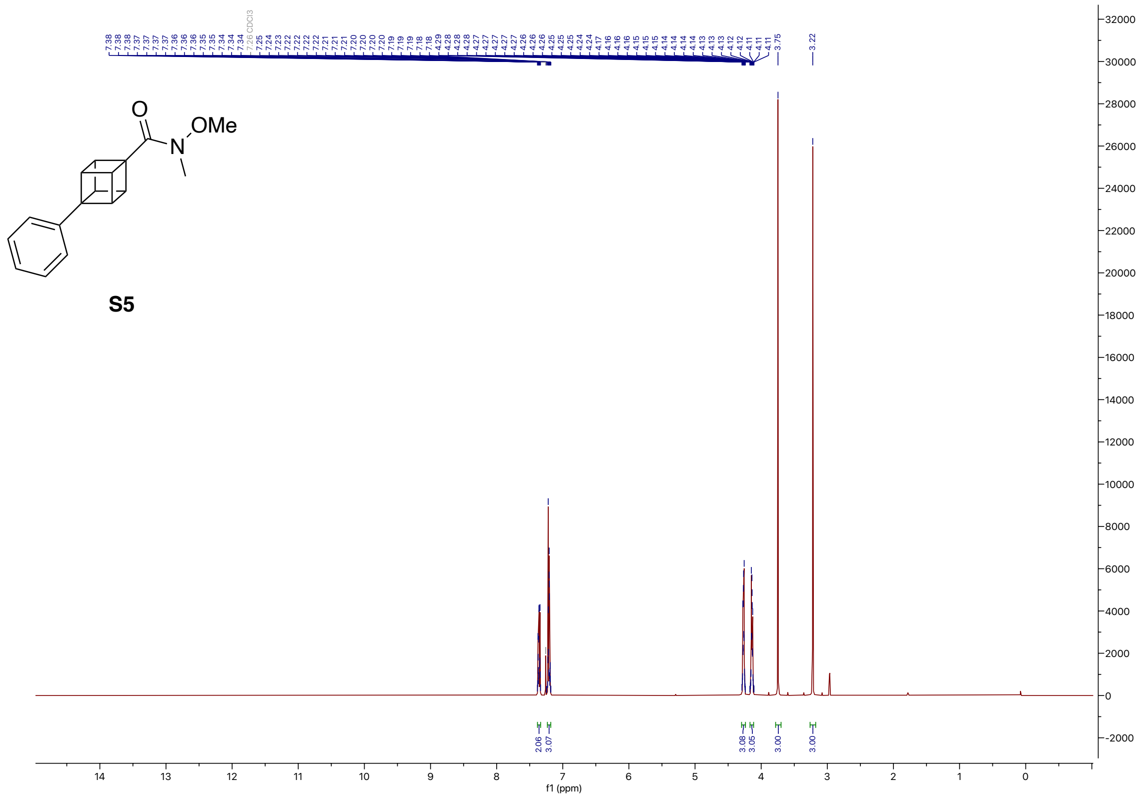

^{13}C NMR (126 MHz, CDCl$_3$) of S5

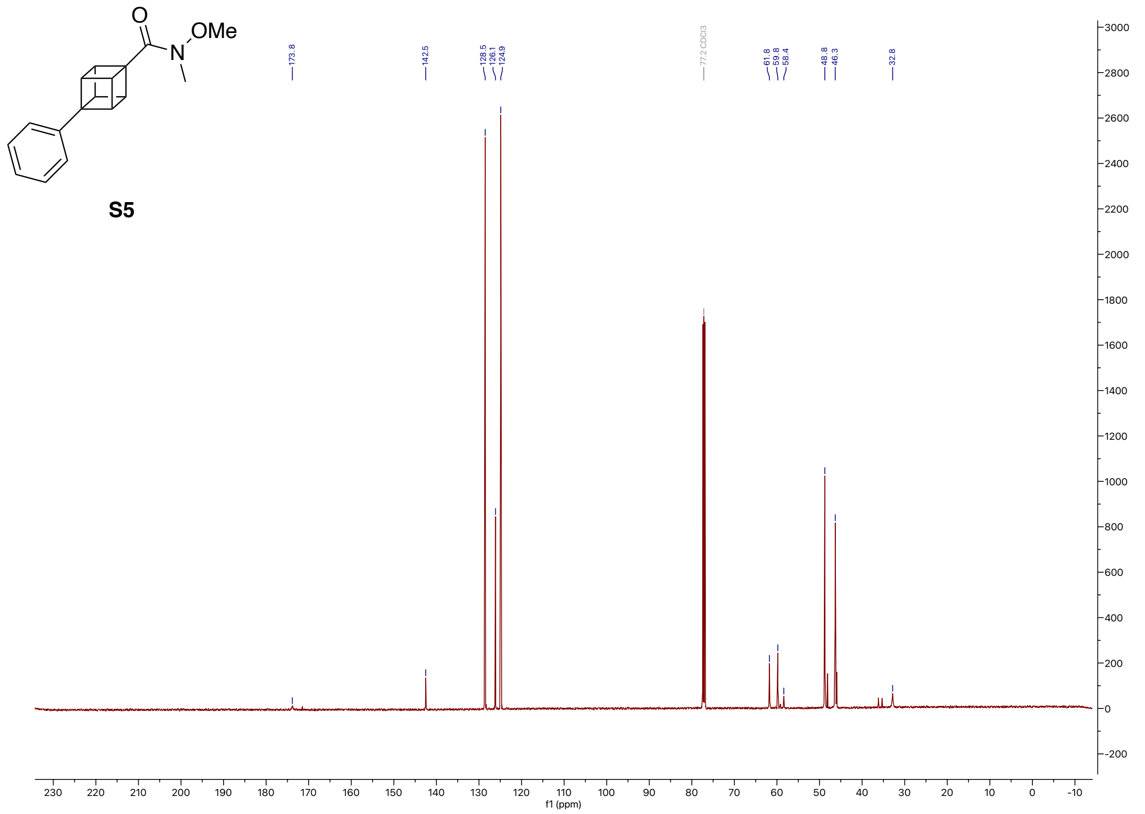

^{1}H NMR (500 MHz, $CDCl_3$) of 1a

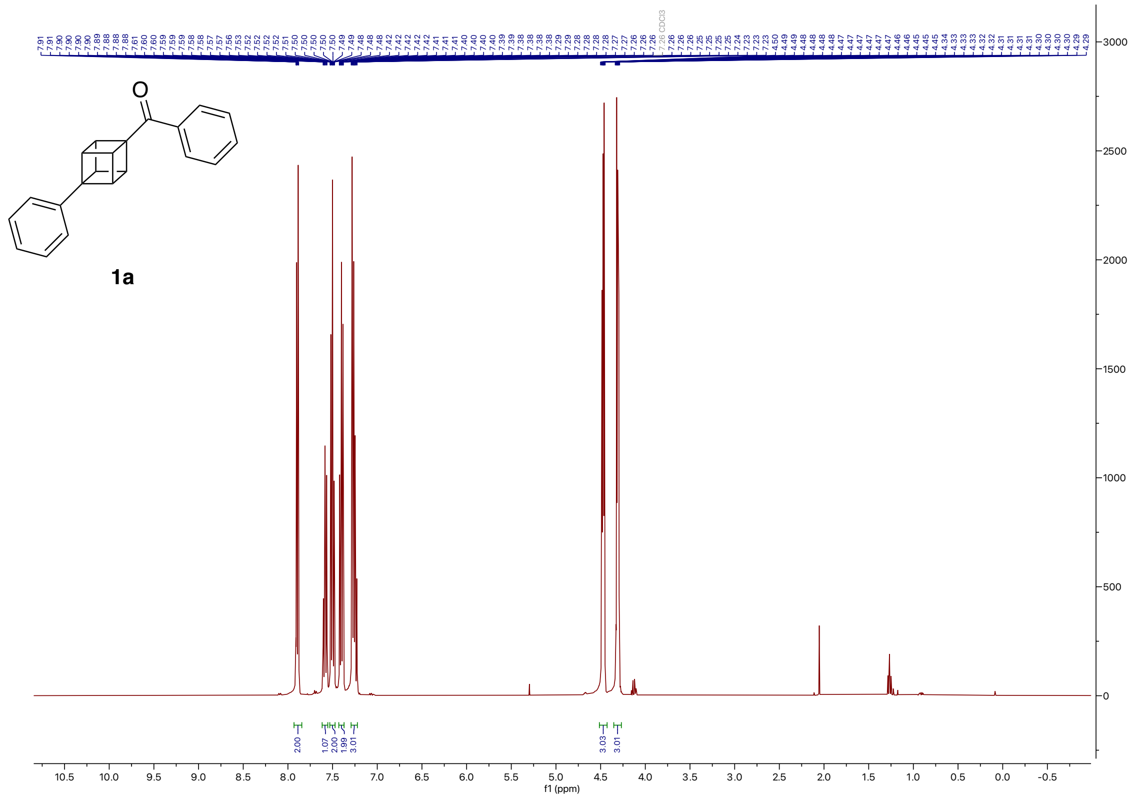

^{13}C NMR (126 MHz, $CDCl_3$) of 1a

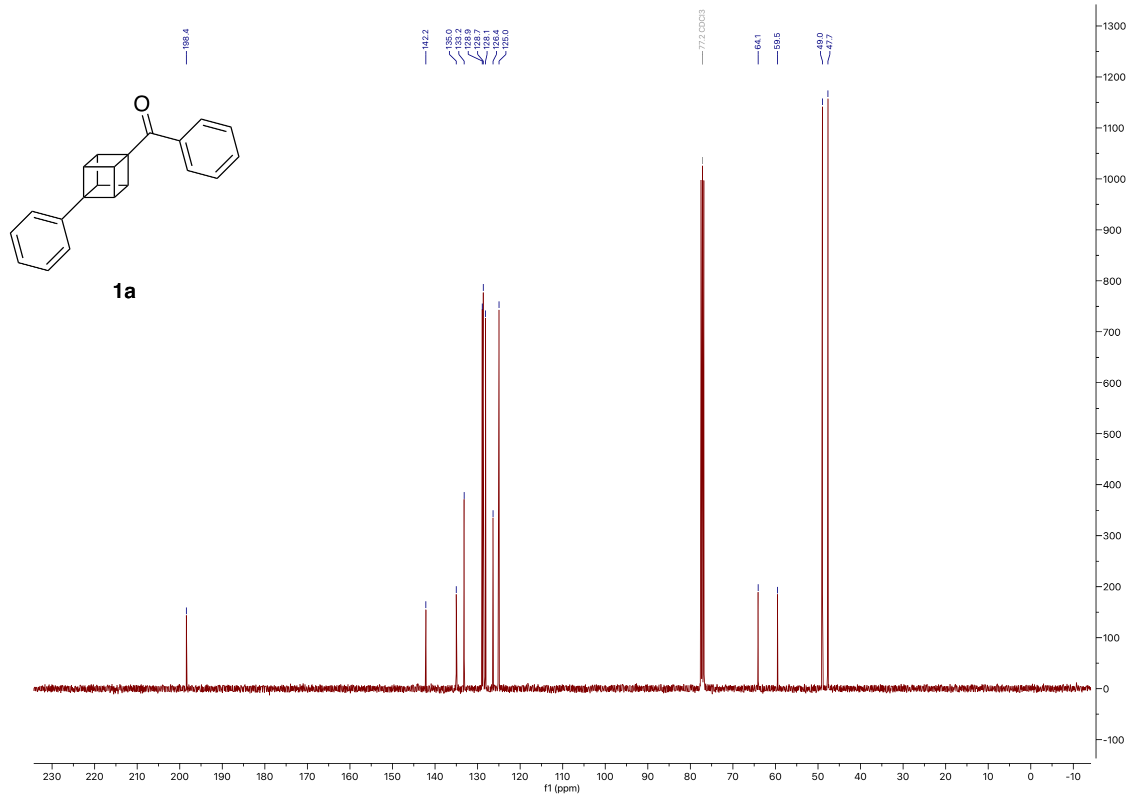

^{1}H NMR (500 MHz, $CDCl_3$) of 1b

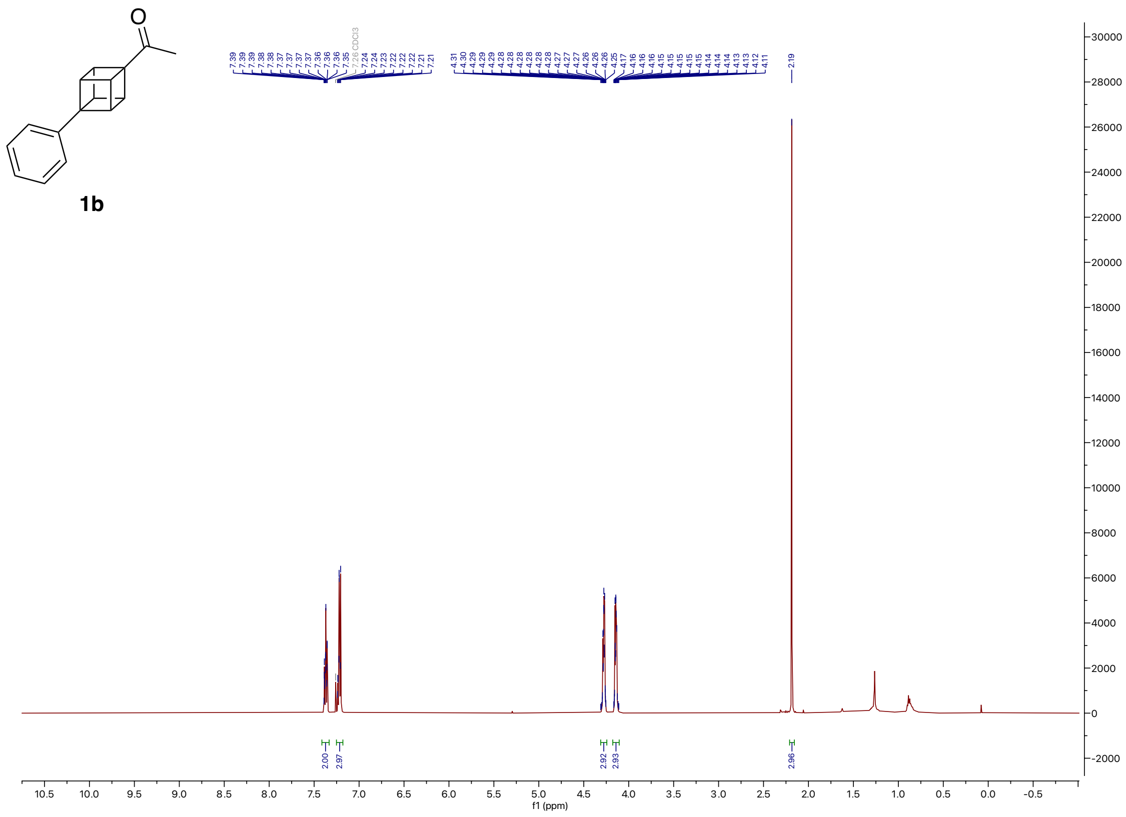

^{13}C NMR (126 MHz, $CDCl_3$) of 1b

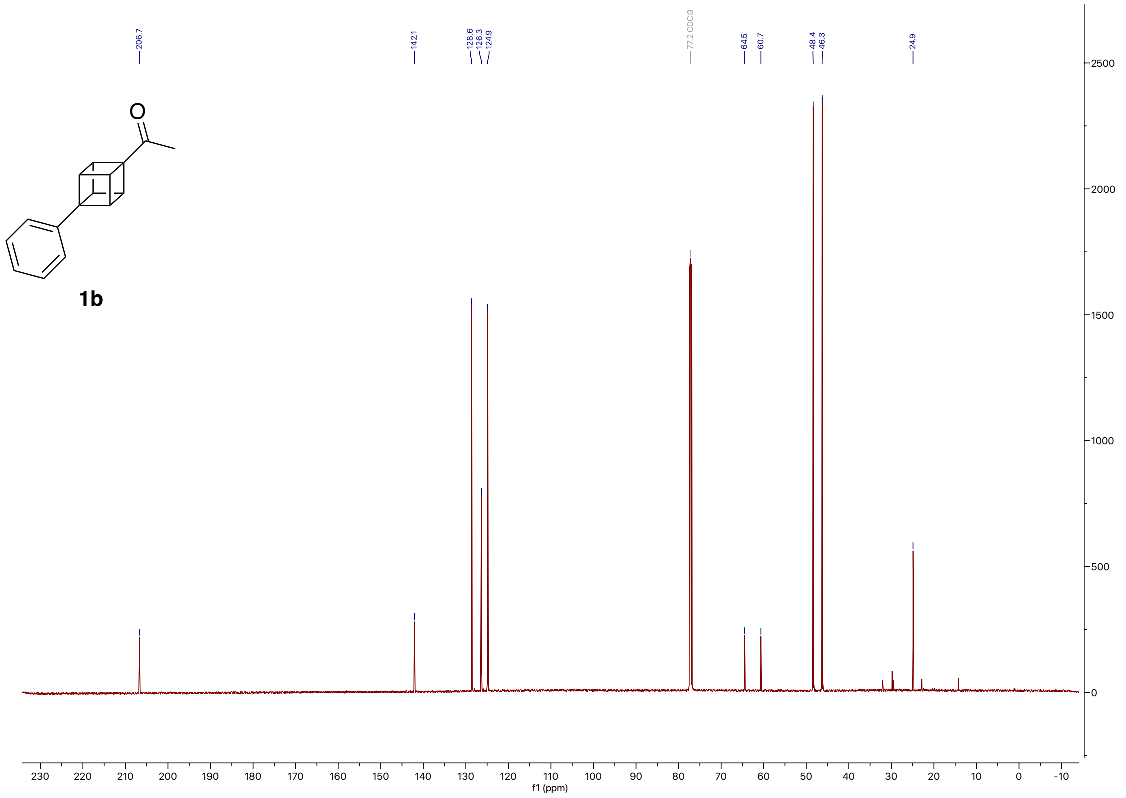

^{1}H NMR (500 MHz, $CDCl_3$) of 1c

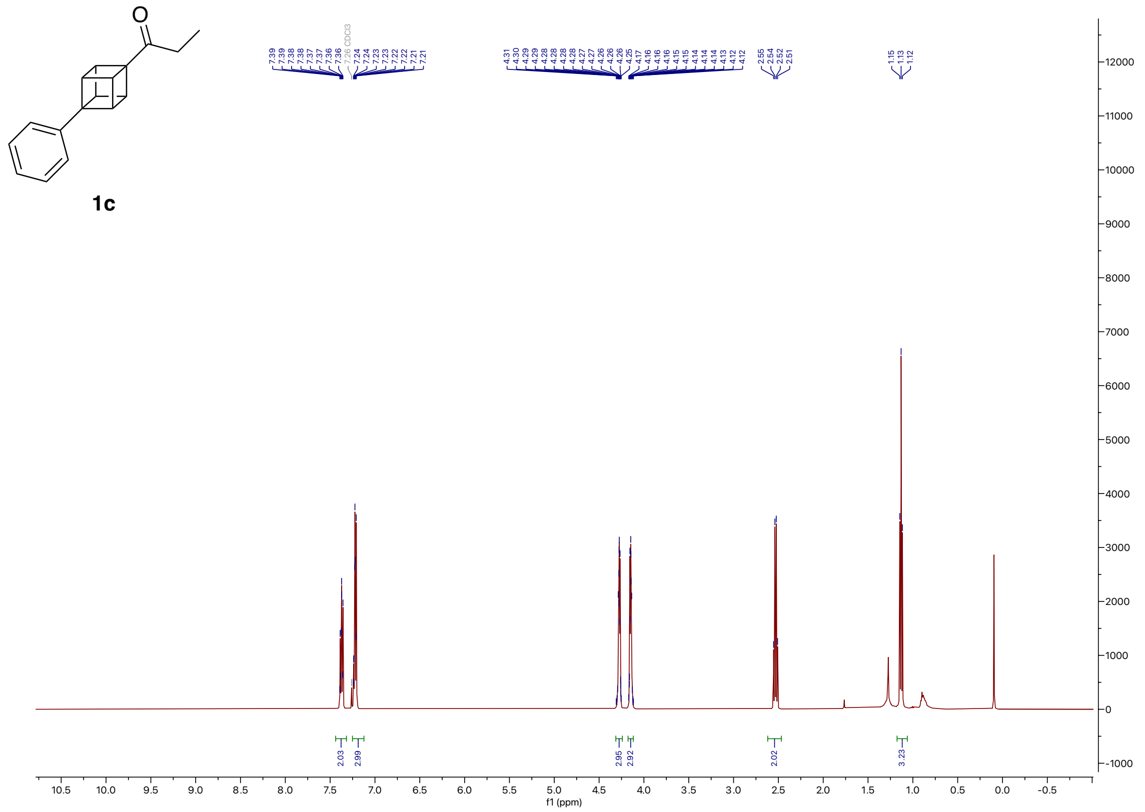

^{13}C NMR (126 MHz, $CDCl_3$) of 1c

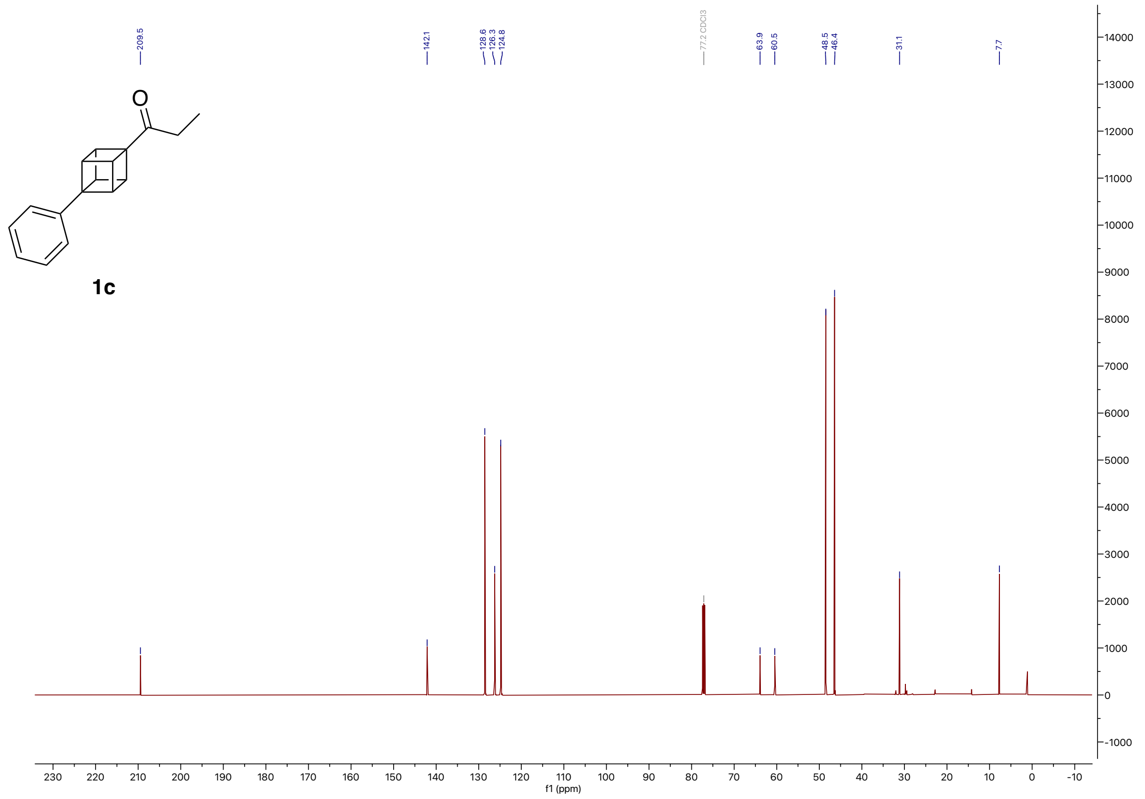

^{1}H NMR (400 MHz, $CDCl_3$) of S4a

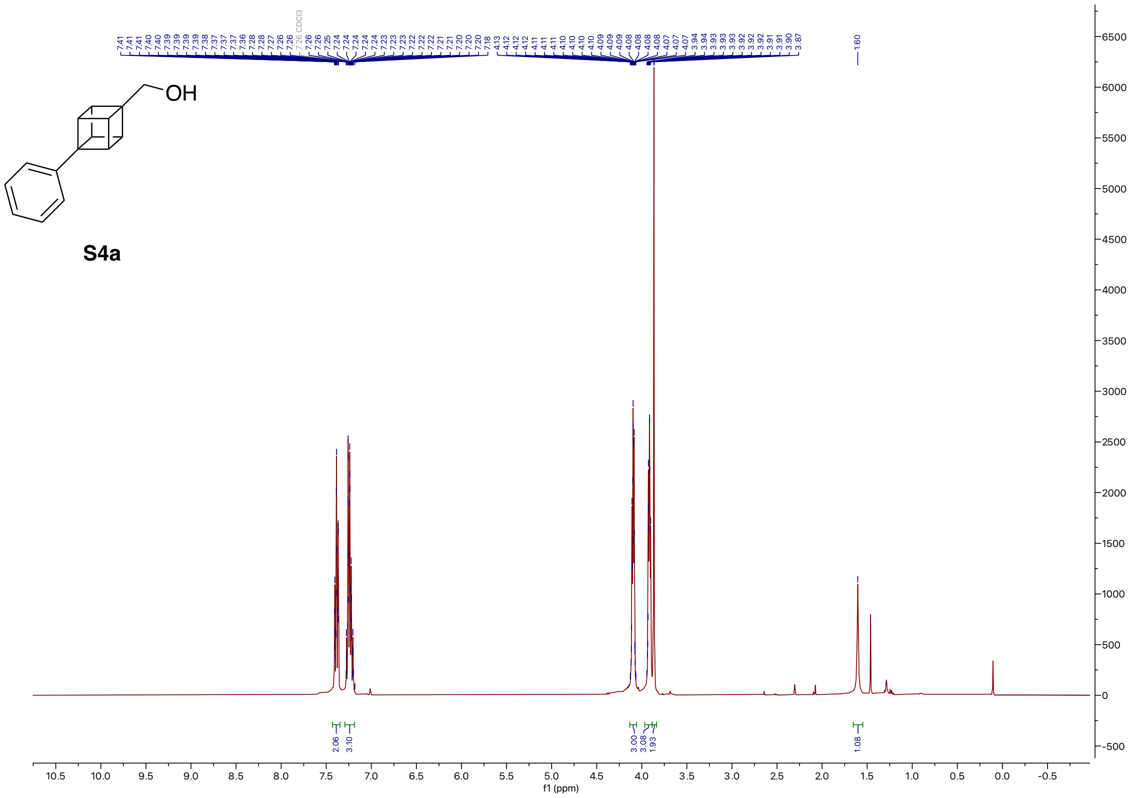

^{13}C NMR (101 MHz, $CDCl_3$) of S4a

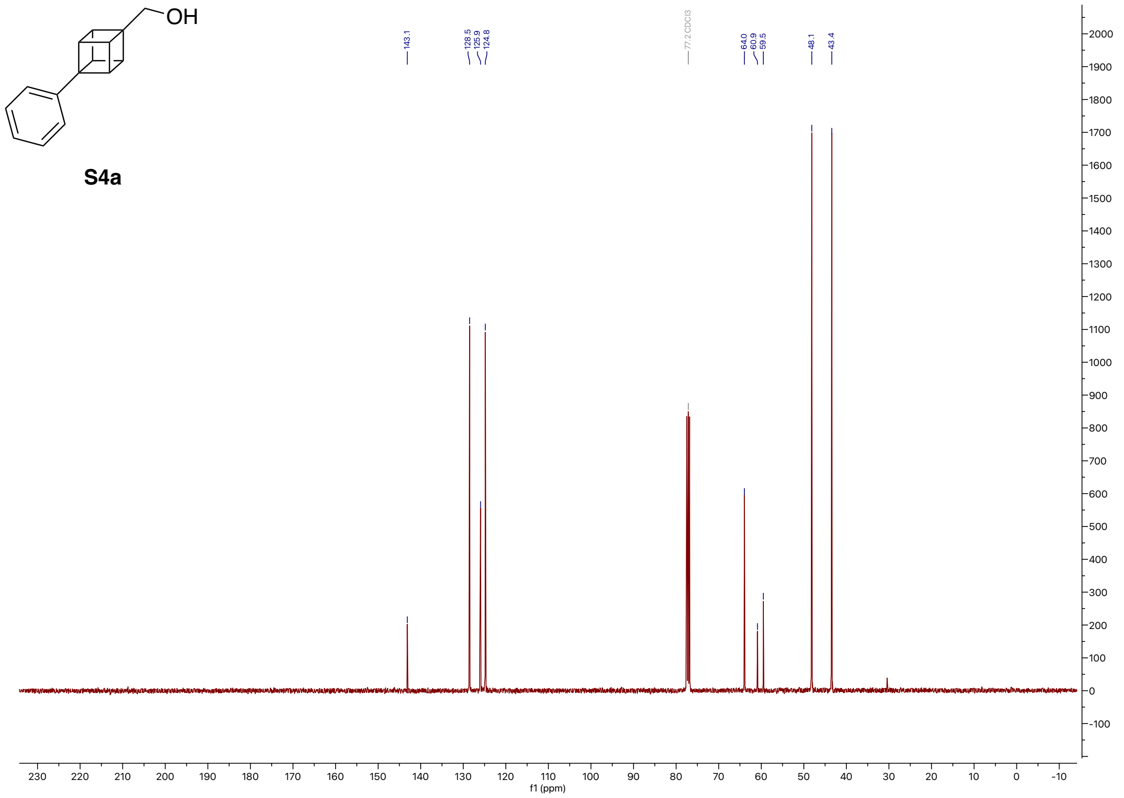

^{1}H NMR (400 MHz, $CDCl_3$) of 1d

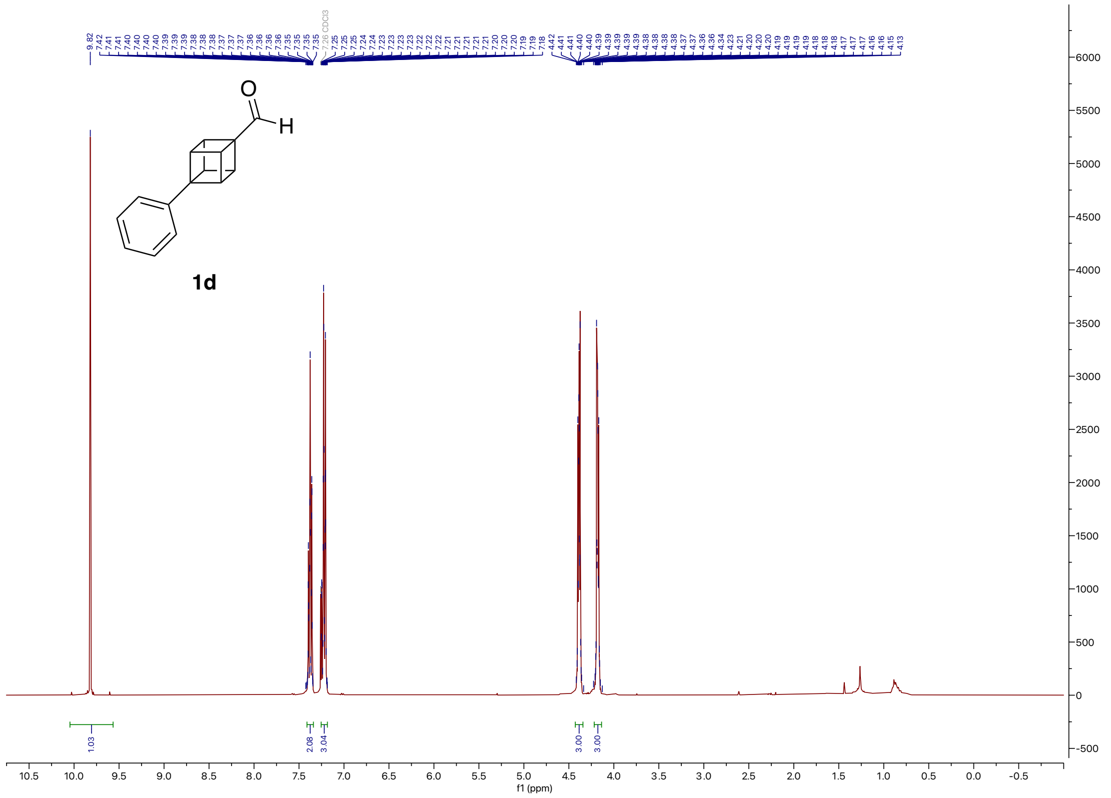

^{13}C NMR (101 MHz, $CDCl_3$) of 1d

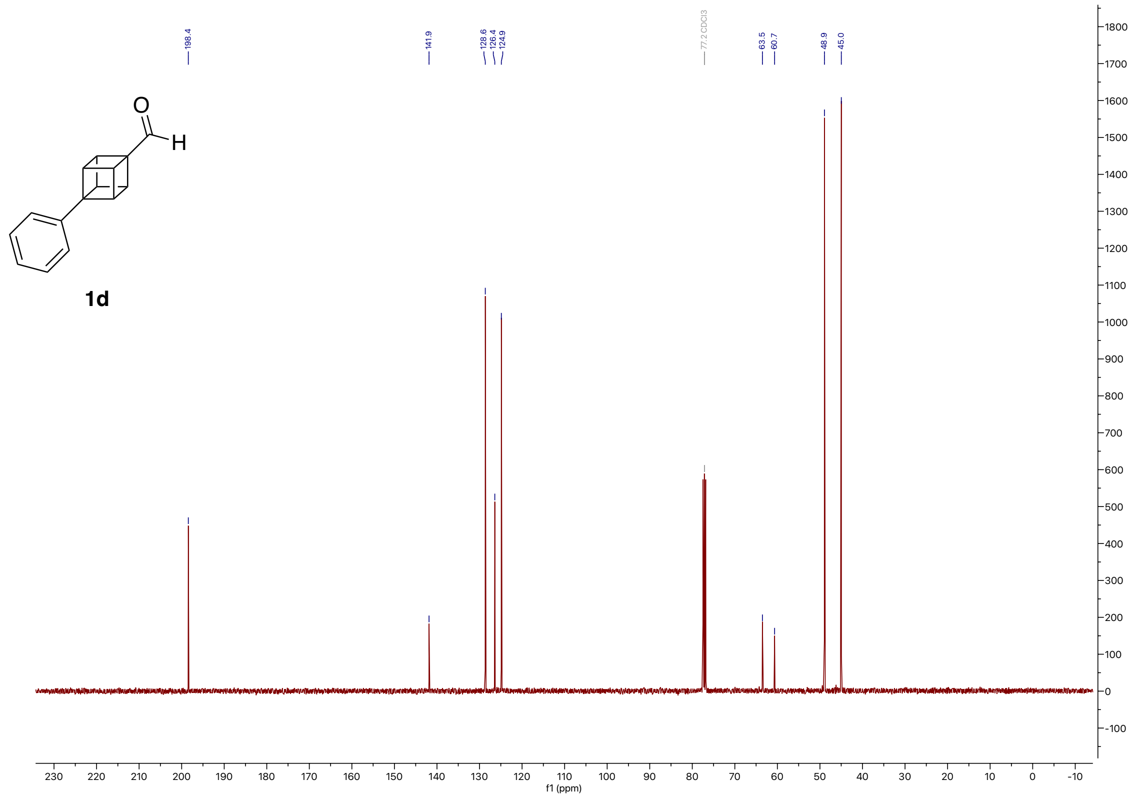

^{1}H NMR (400 MHz, $CDCl_3$) of 1e

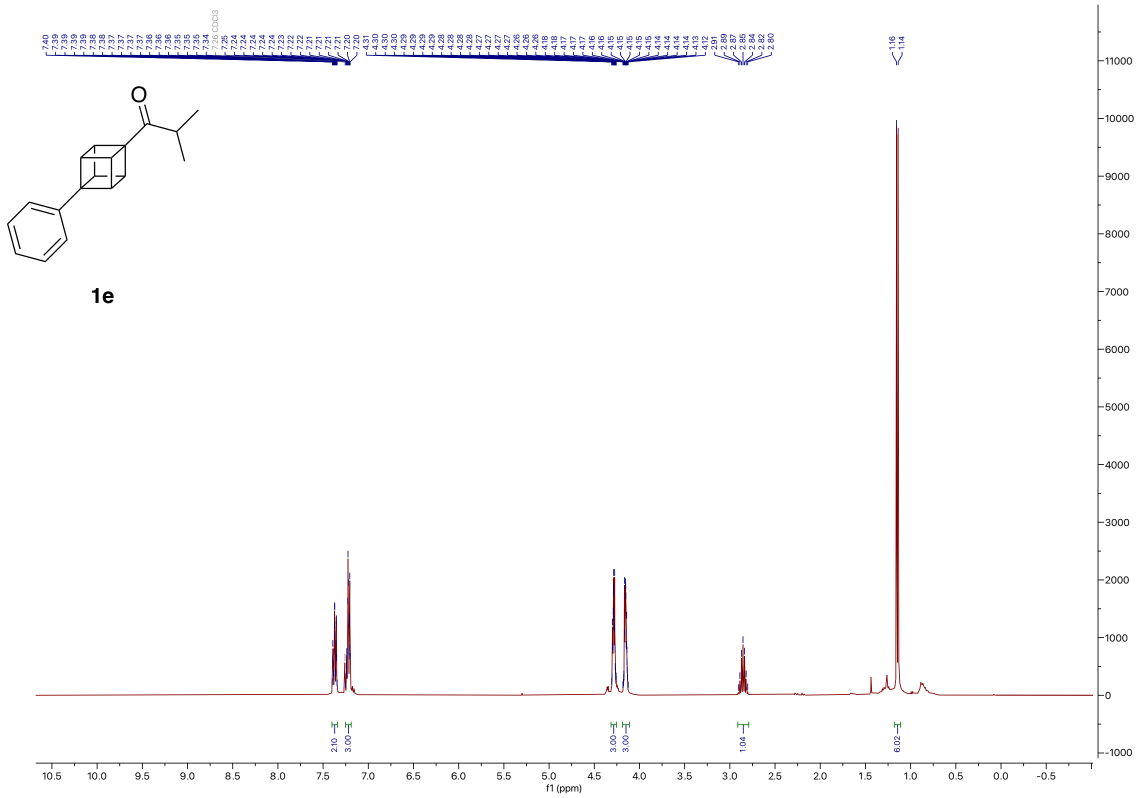

^{13}C NMR (101 MHz, $CDCl_3$) of 1e

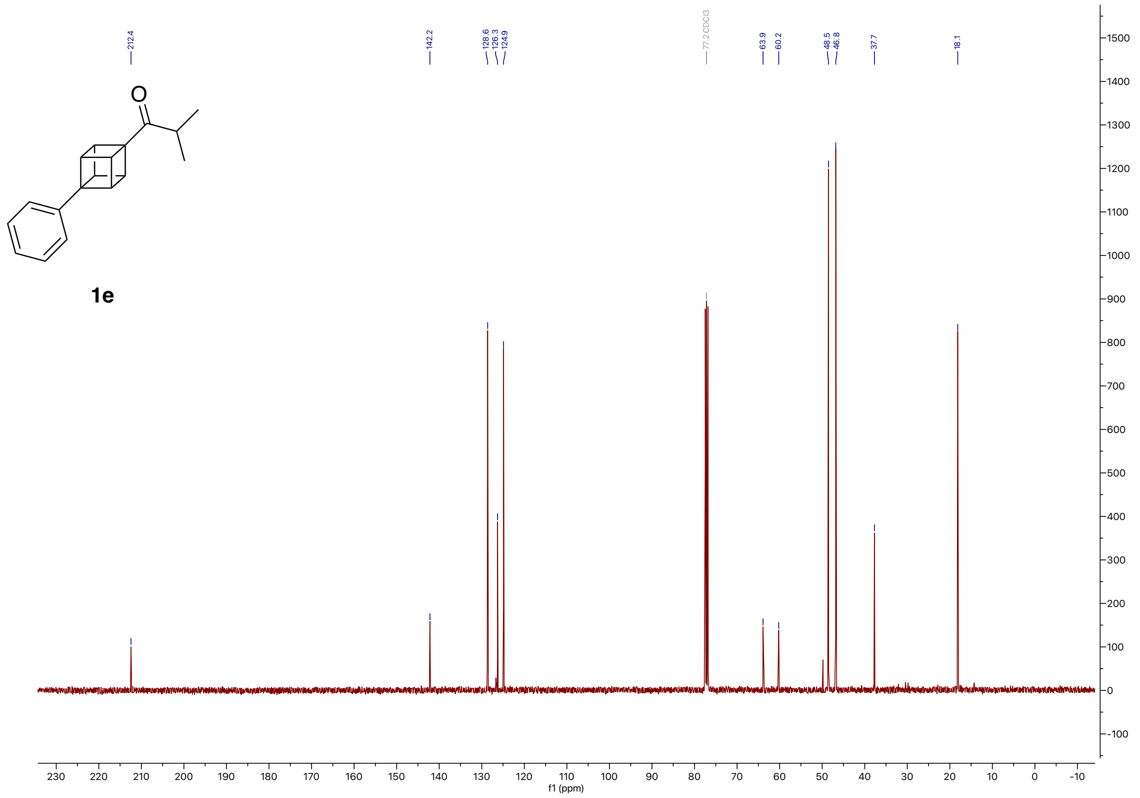

^{1}H NMR (400 MHz, $CDCl_3$) of 1f

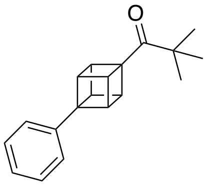

1f

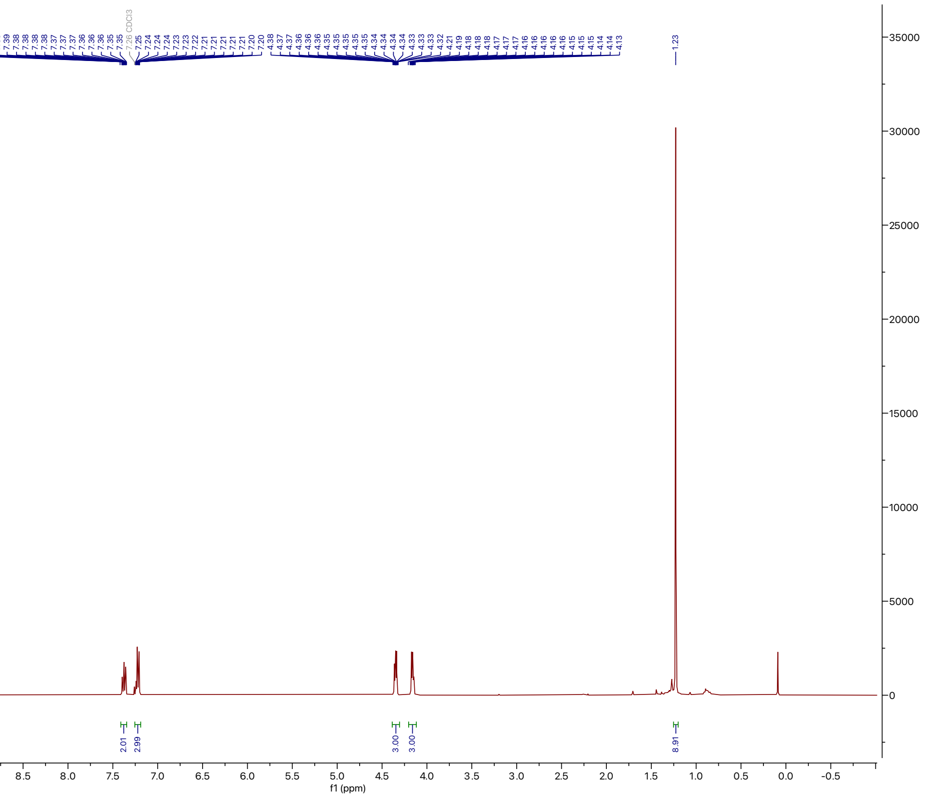

^{13}C NMR (101 MHz, $CDCl_3$) of 1f

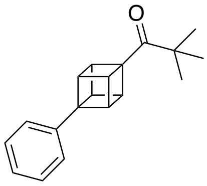

1f

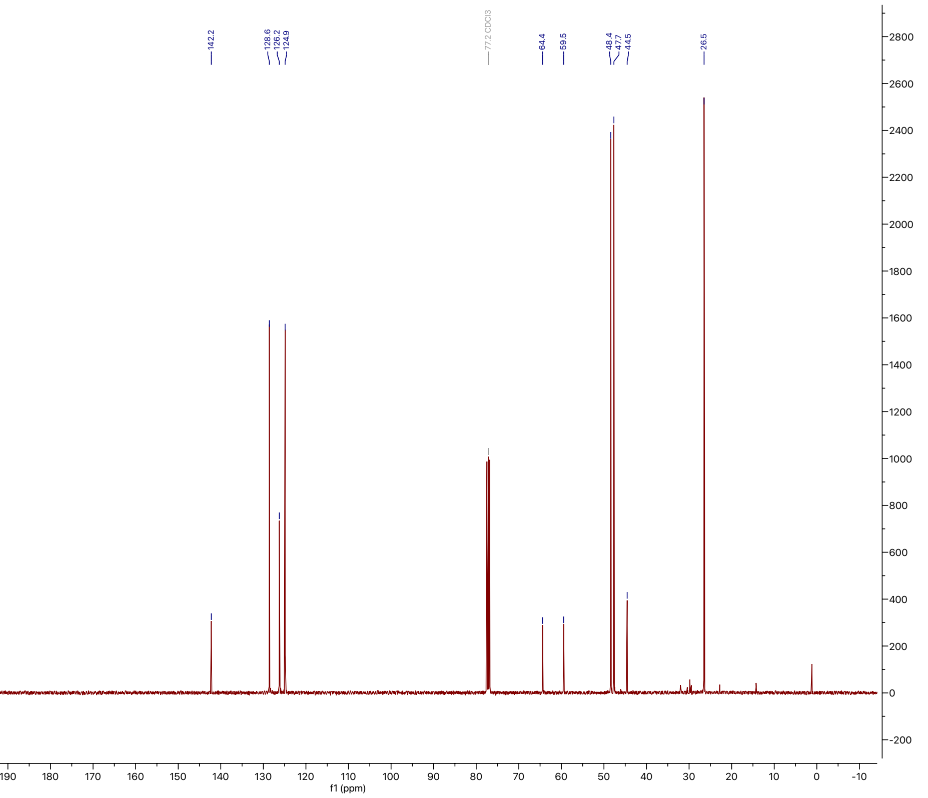

^{1}H NMR (400 MHz, $CDCl_3$) of S6

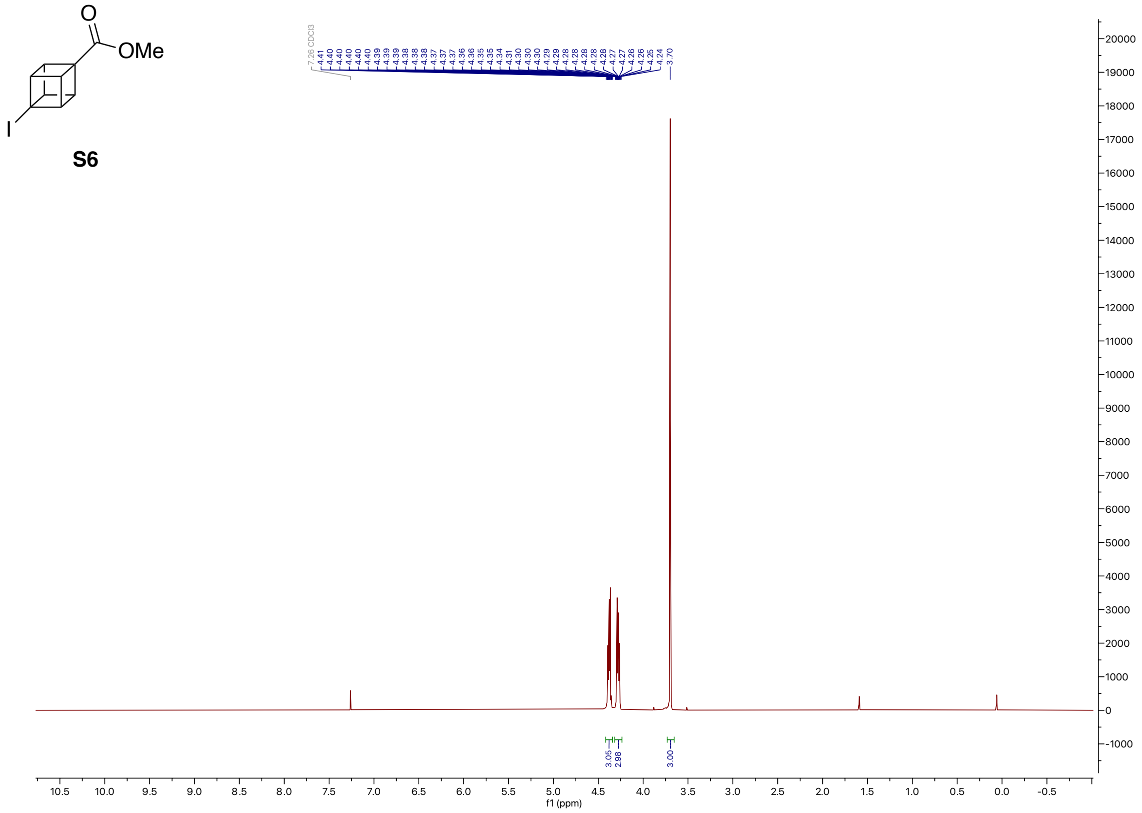

^{13}C NMR (101 MHz, $CDCl_3$) of S6

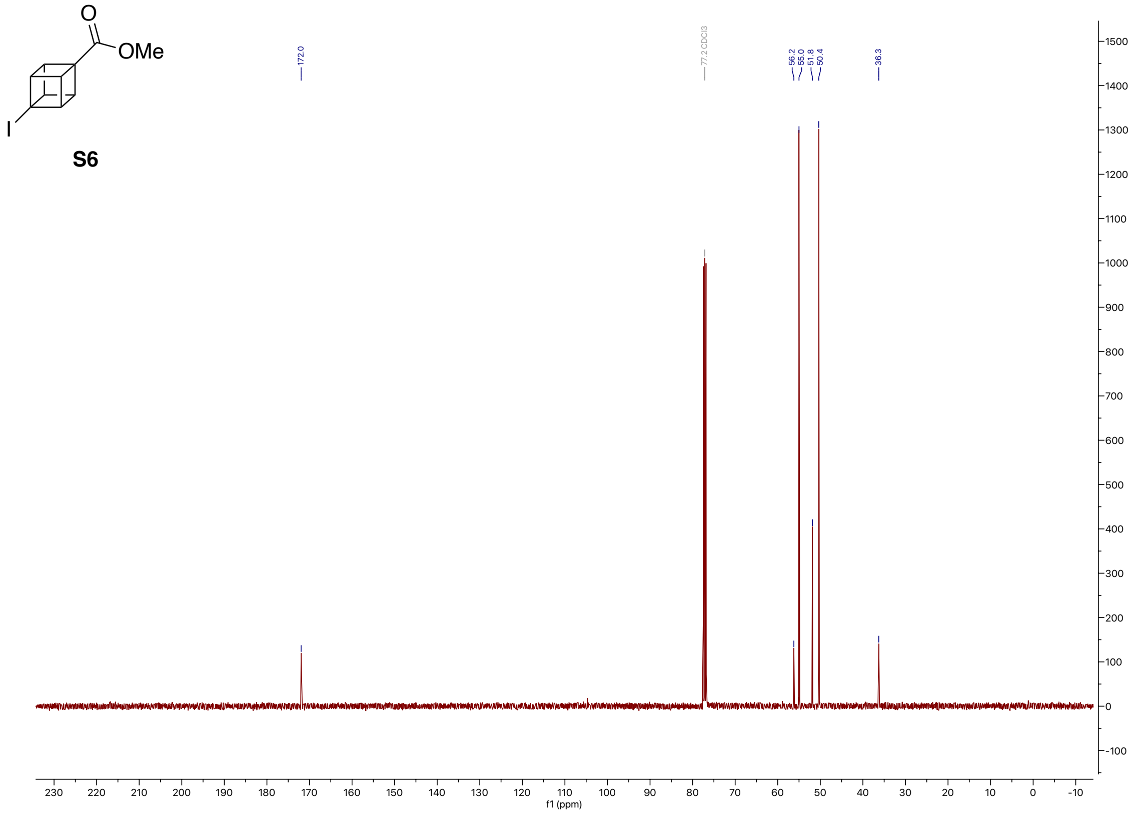

^{1}H NMR (400 MHz, $CDCl_3$) of S10

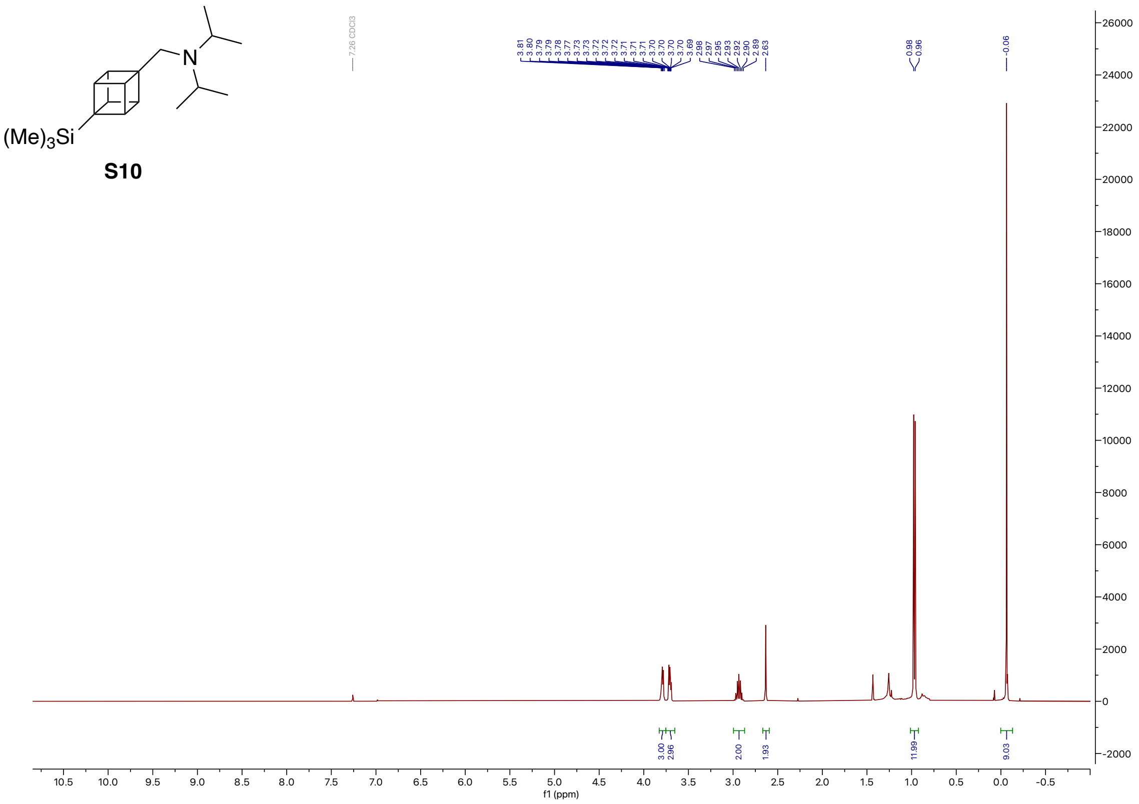

^{13}C NMR (101 MHz, $CDCl_3$) of S10

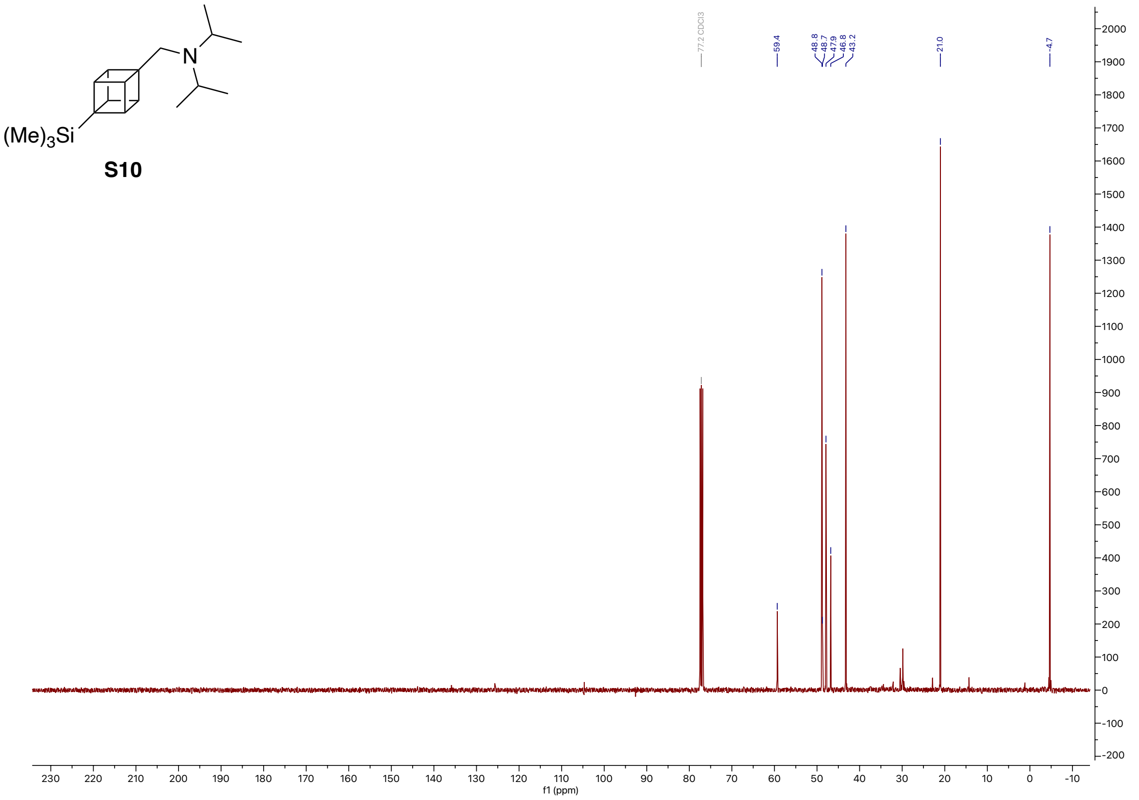

^{1}H NMR (500 MHz, $CDCl_3$) of S11

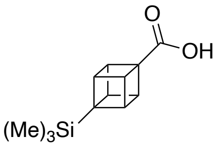

S11

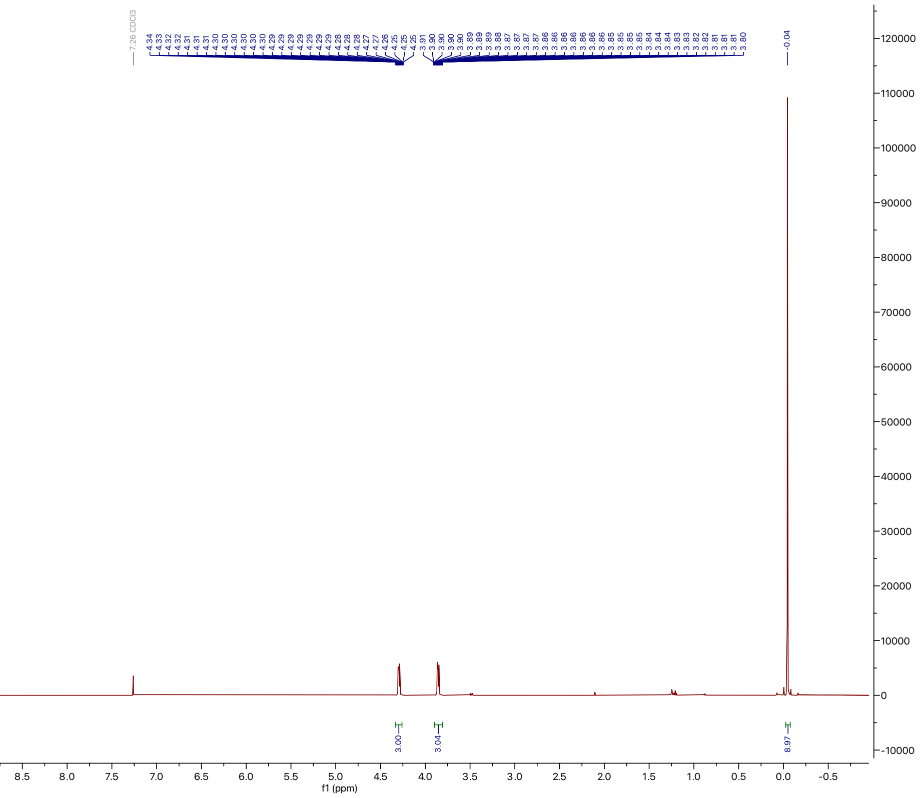

^{13}C NMR (126 MHz, $CDCl_3$) of S11

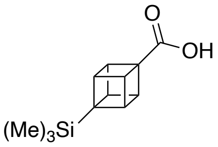

S11

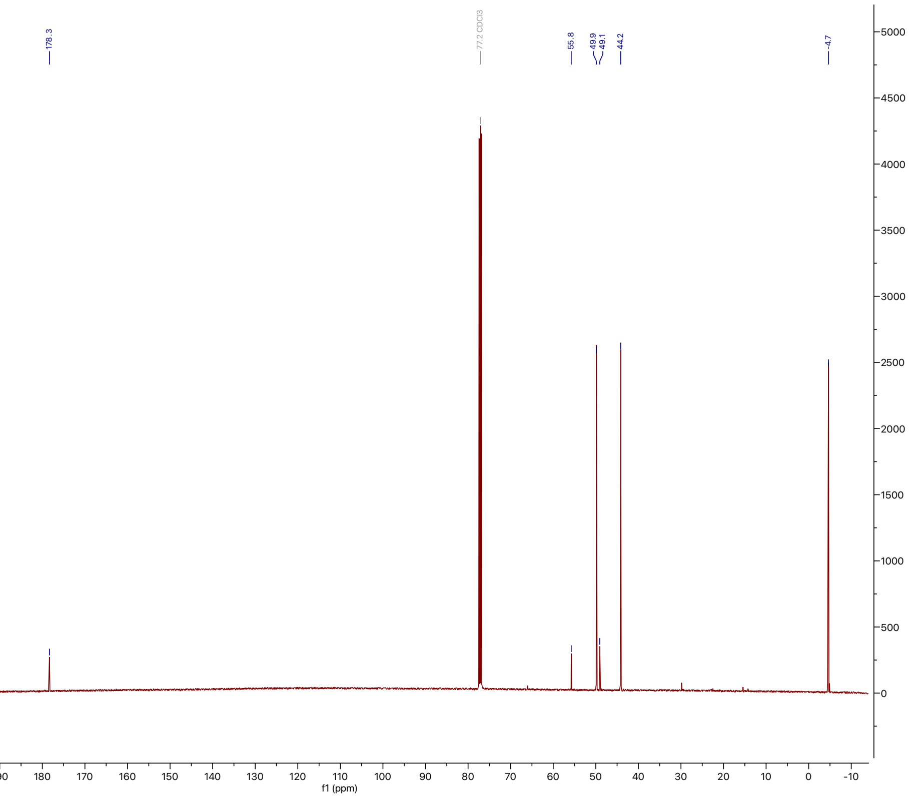

^{1}H NMR (500 MHz, $CDCl_3$) of S12

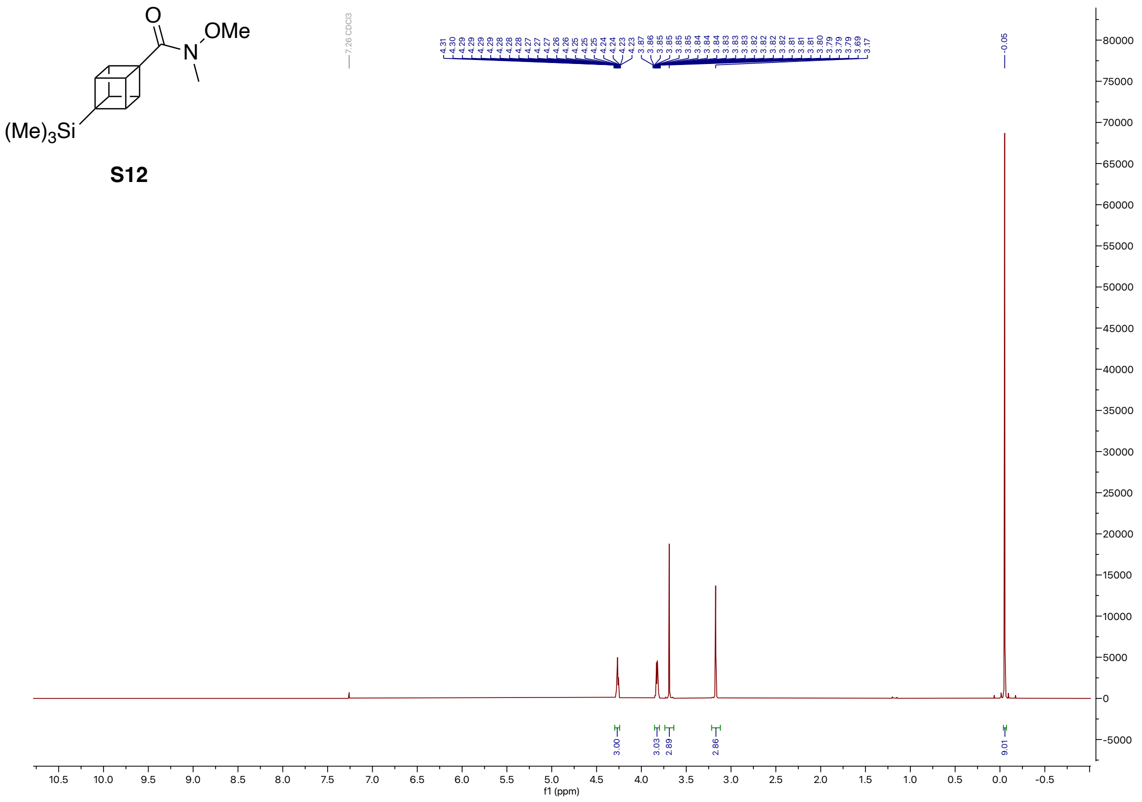

^{13}C NMR (126 MHz, $CDCl_3$) of S12

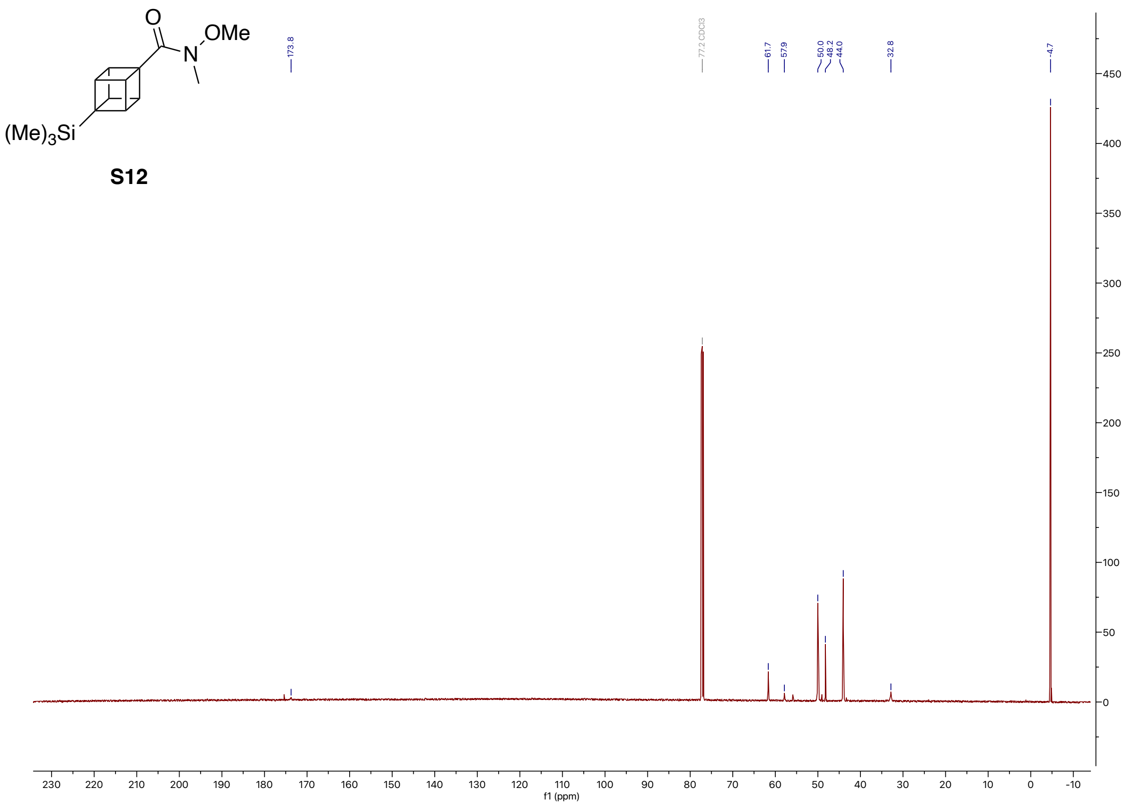

^{1}H NMR (400 MHz, $CDCl_3$) of 1g

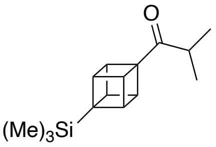

1g

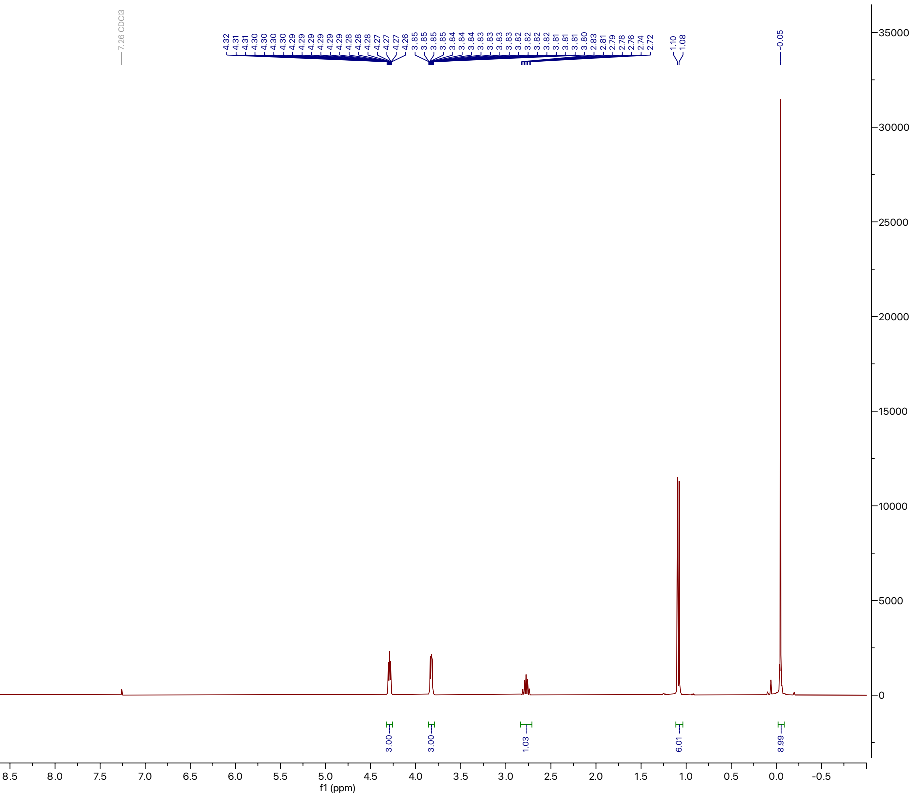

^{13}C NMR (101 MHz, $CDCl_3$) of 1g

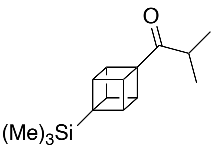

1g

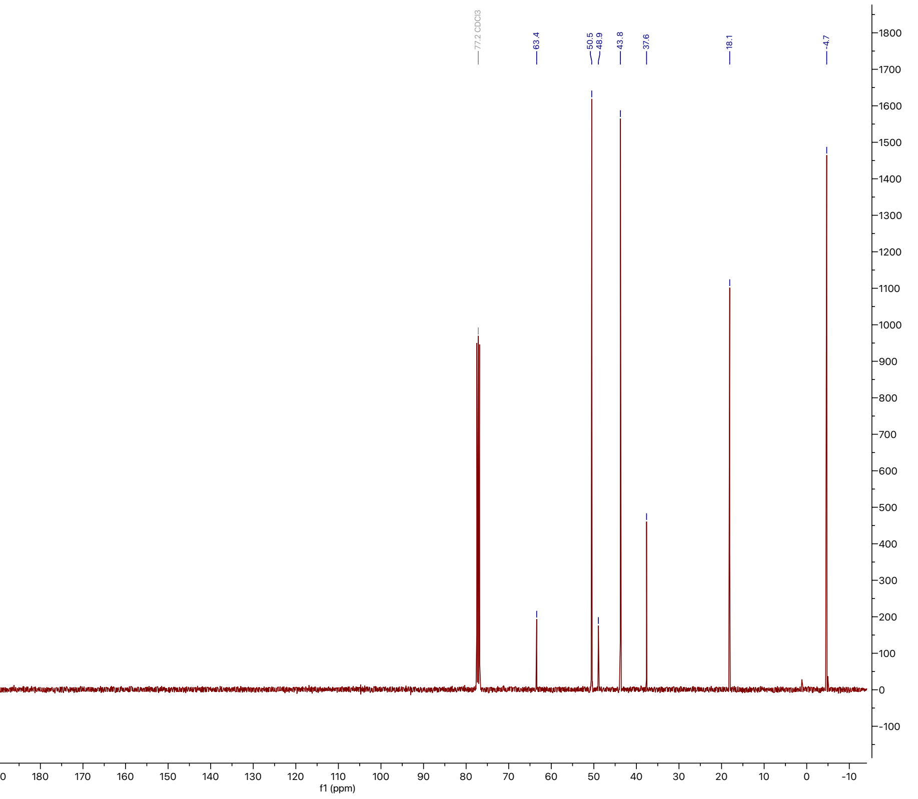

^{1}H NMR (400 MHz, $CDCl_3$) of 1h

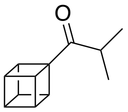

1h

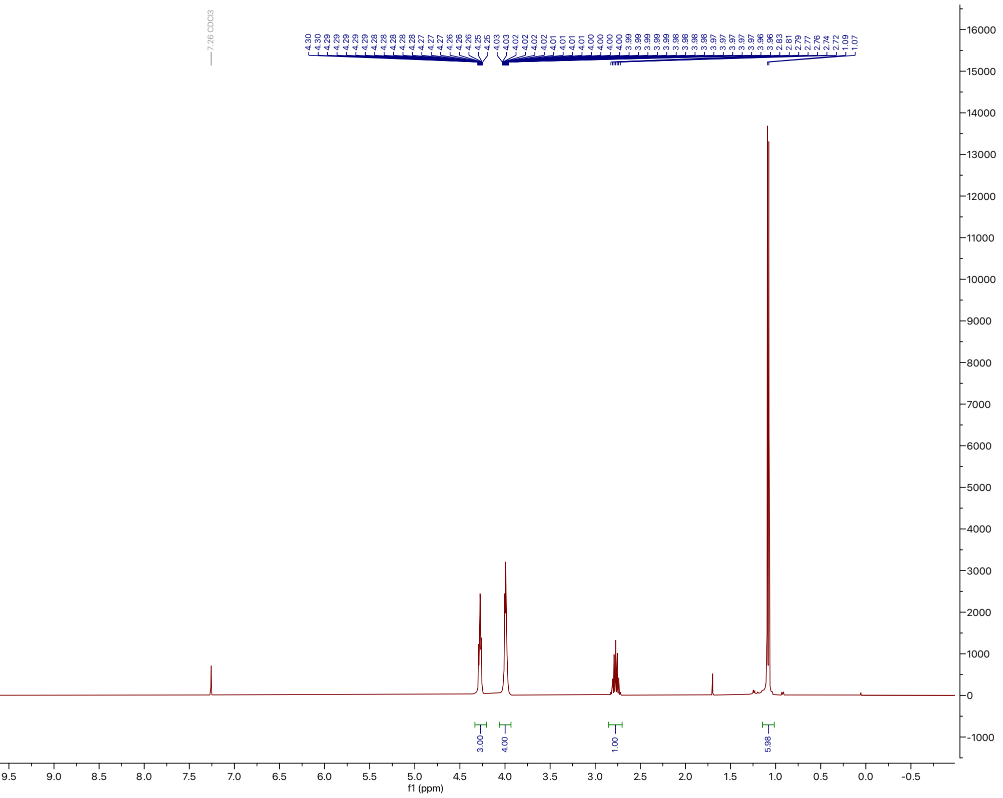

^{13}C NMR (101 MHz, $CDCl_3$) of 1h

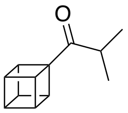

1h

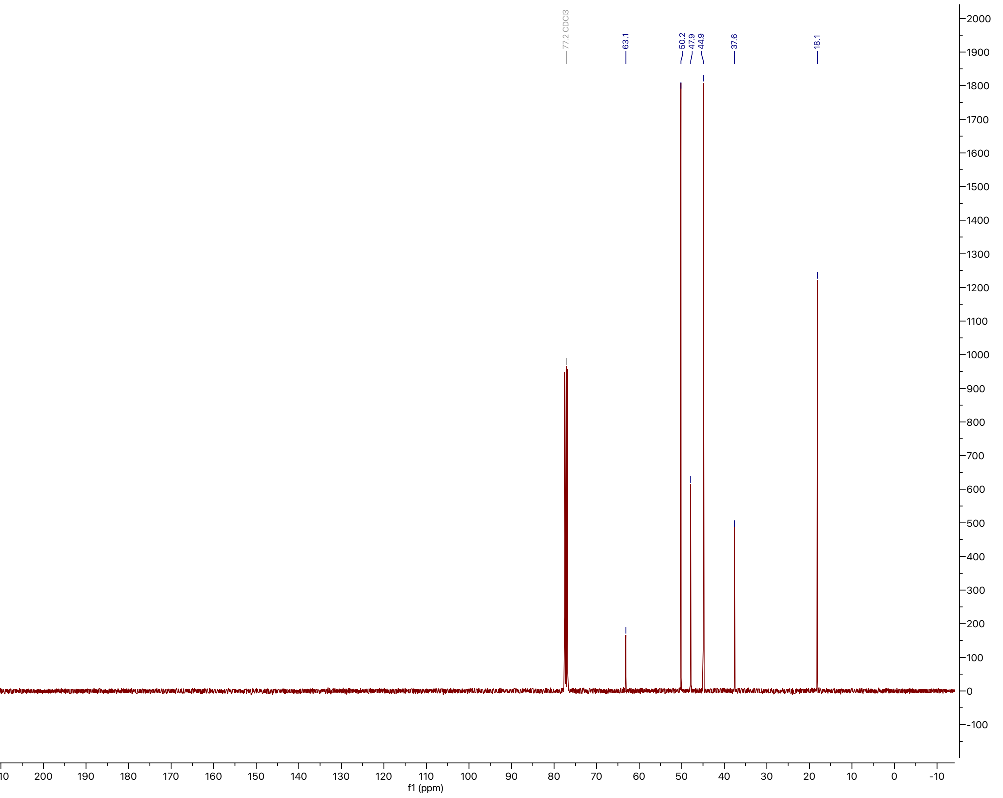

^{1}H NMR (400 MHz, $CDCl_3$) of S17

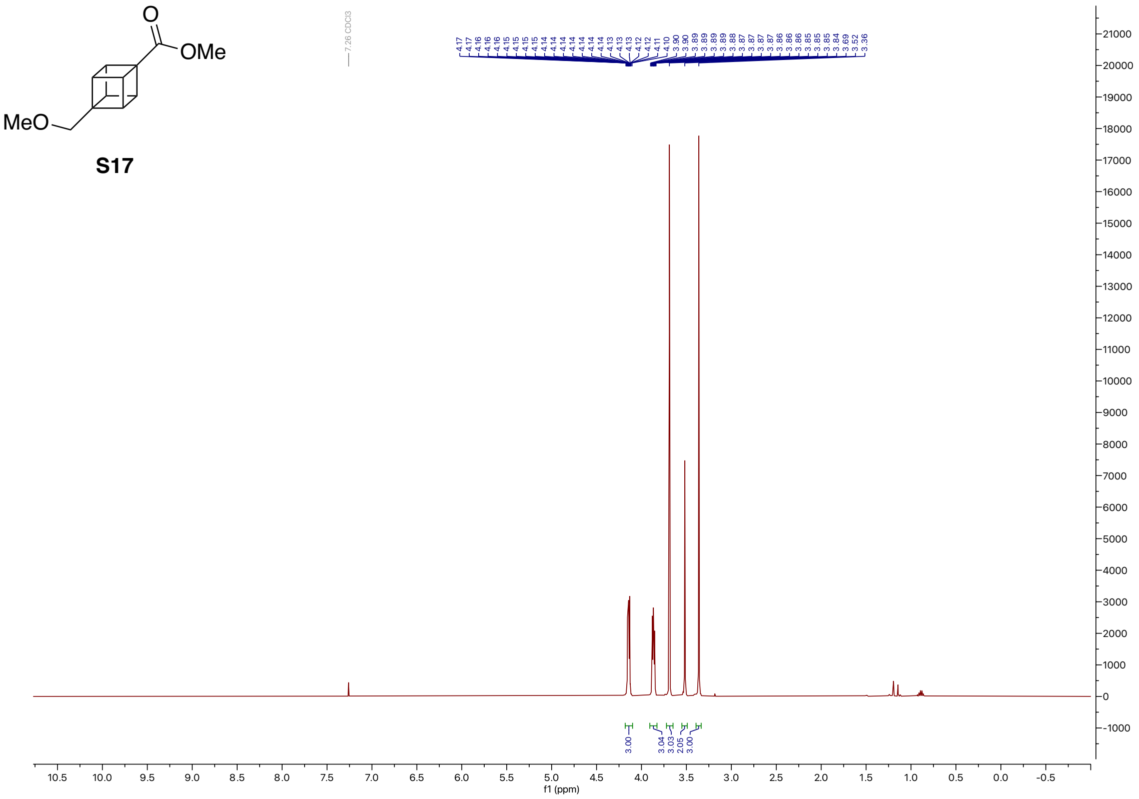

^{13}C NMR (101 MHz, $CDCl_3$) of S17

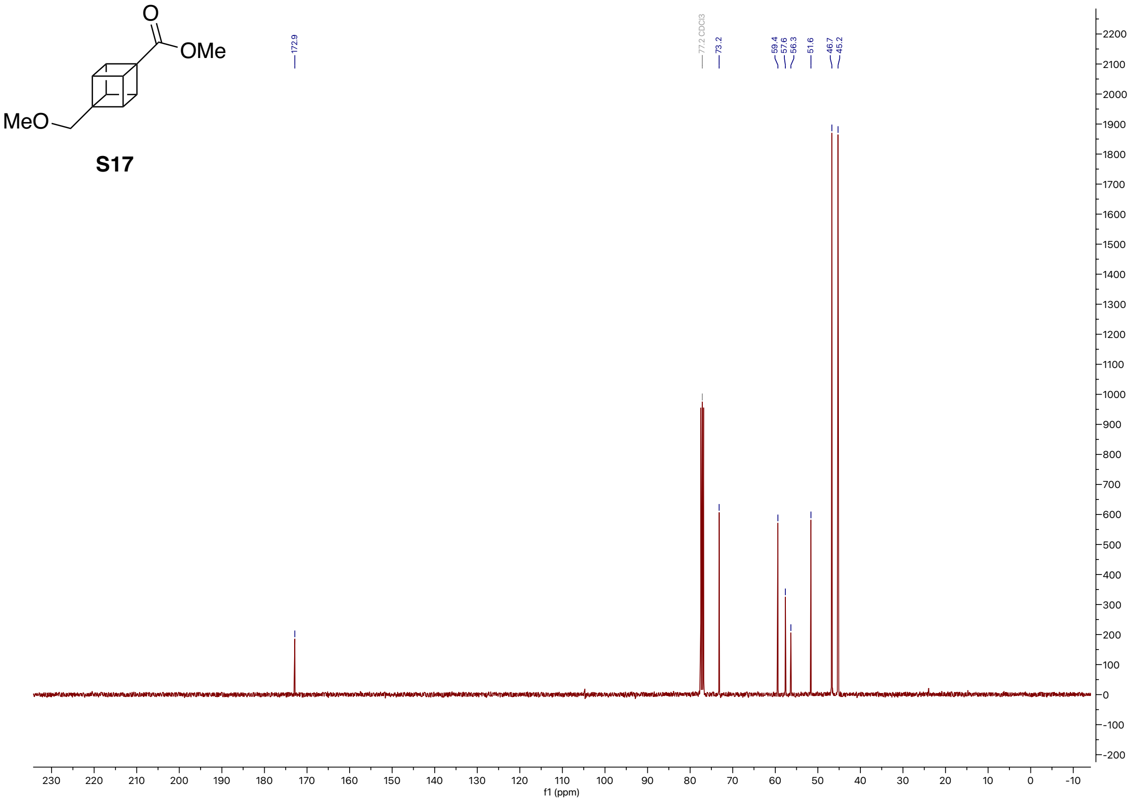

^{1}H NMR (400 MHz, $CDCl_3$) of S18

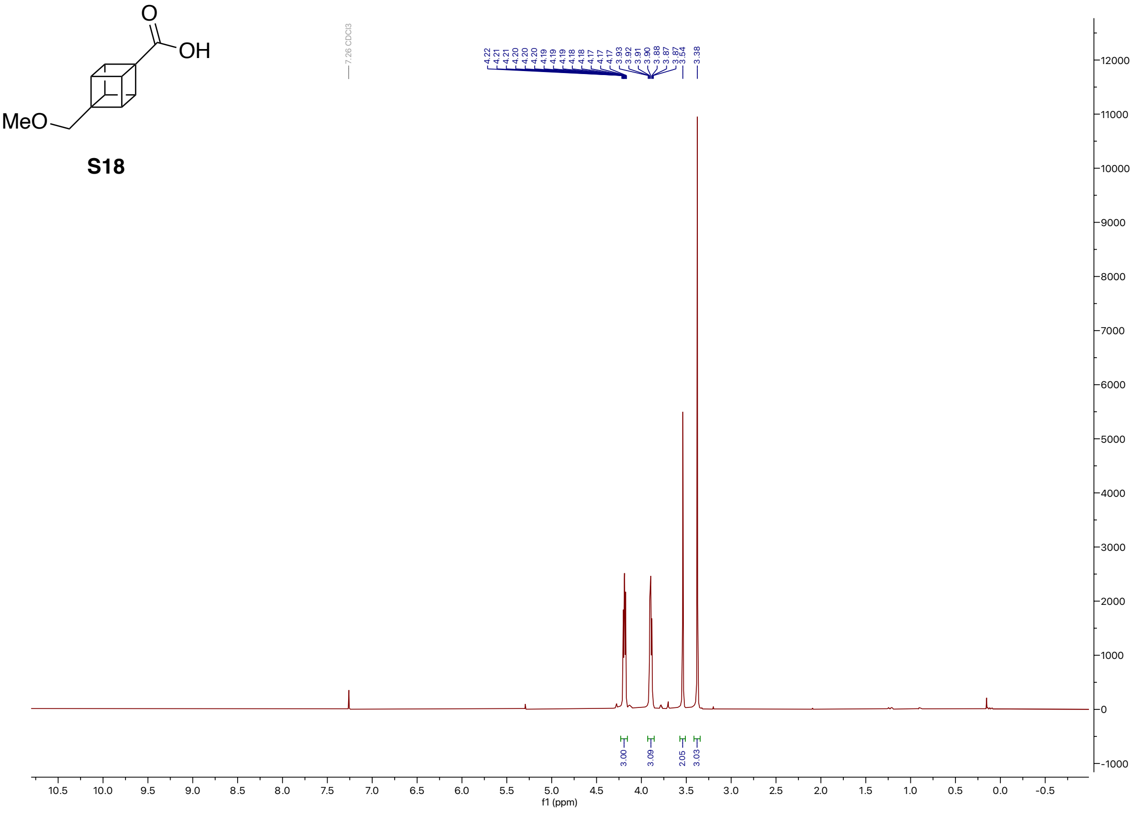

^{13}C NMR (101 MHz, $CDCl_3$) of S18

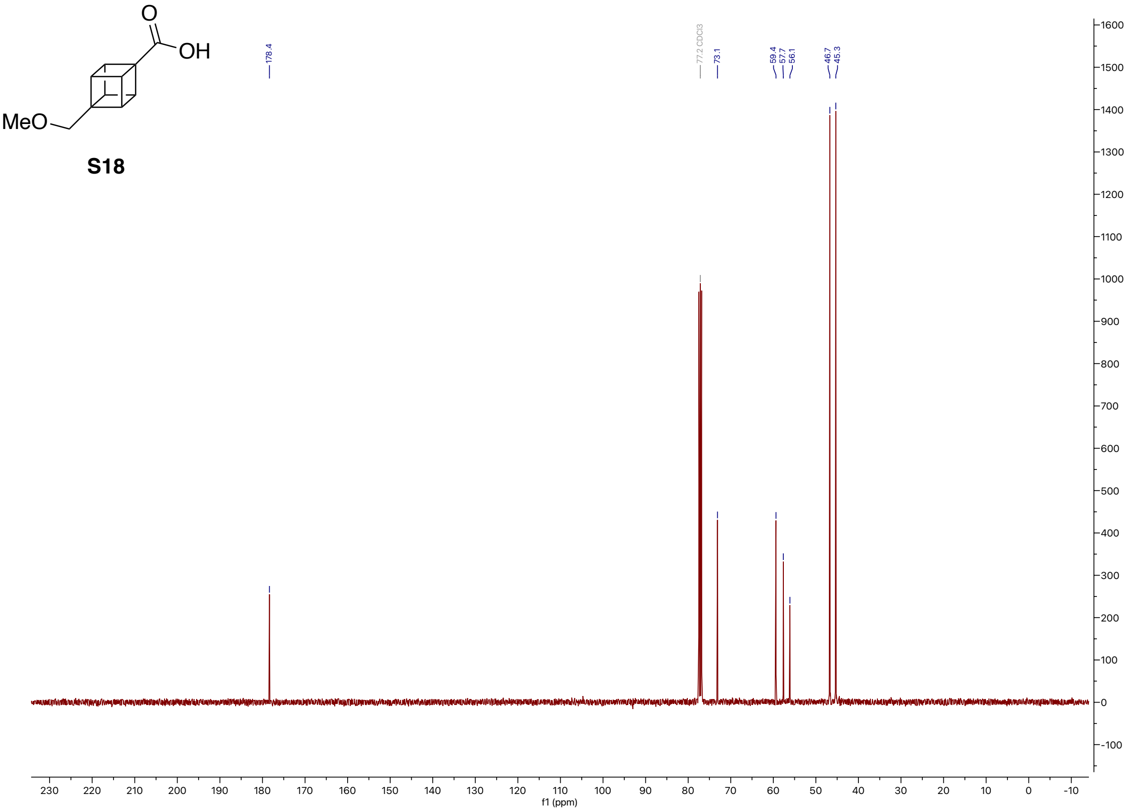

^{1}H NMR (400 MHz, CDCl$_3$) of S19

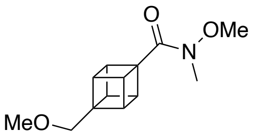

S19

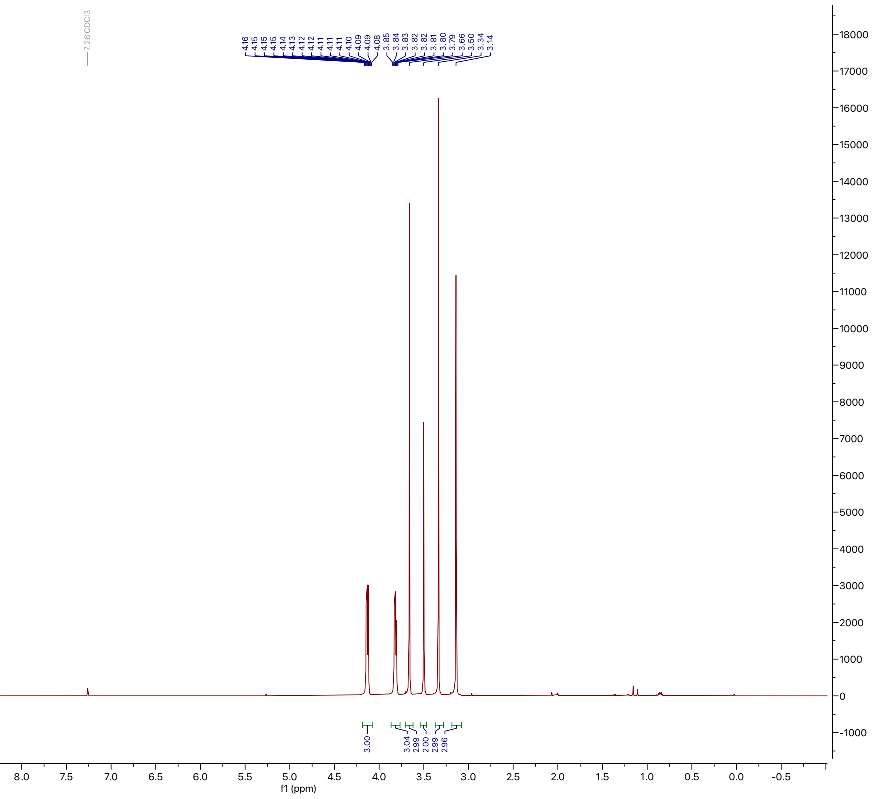

^{13}C NMR (101 MHz, CDCl$_3$) of S19

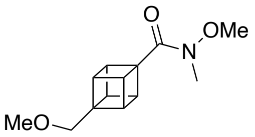

S19

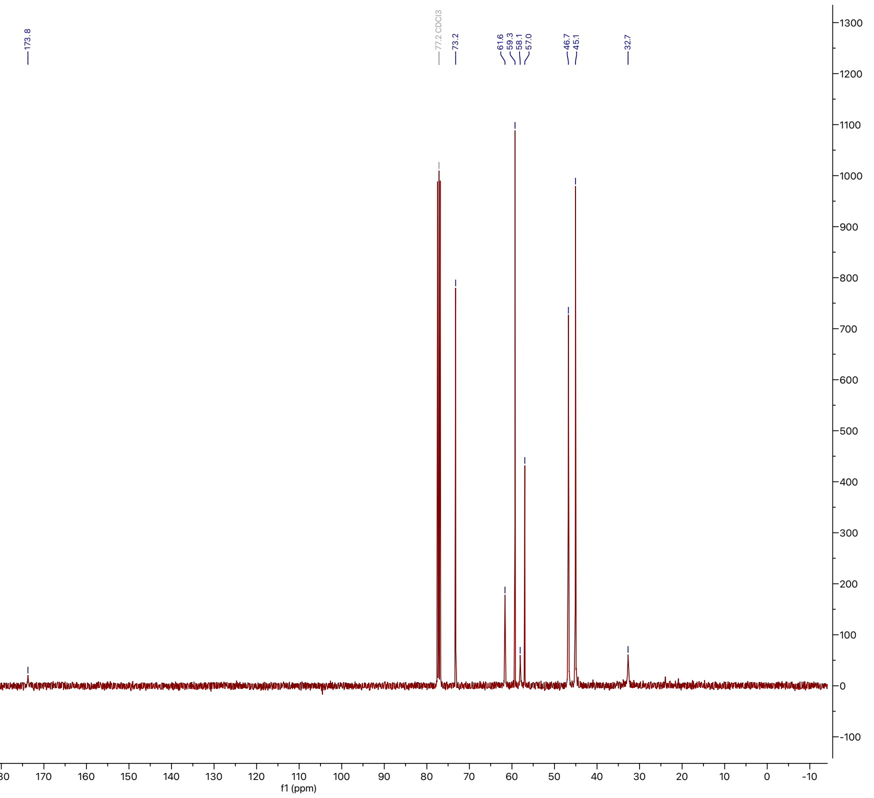

^{1}H NMR (400 MHz, $CDCl_3$) of 1i

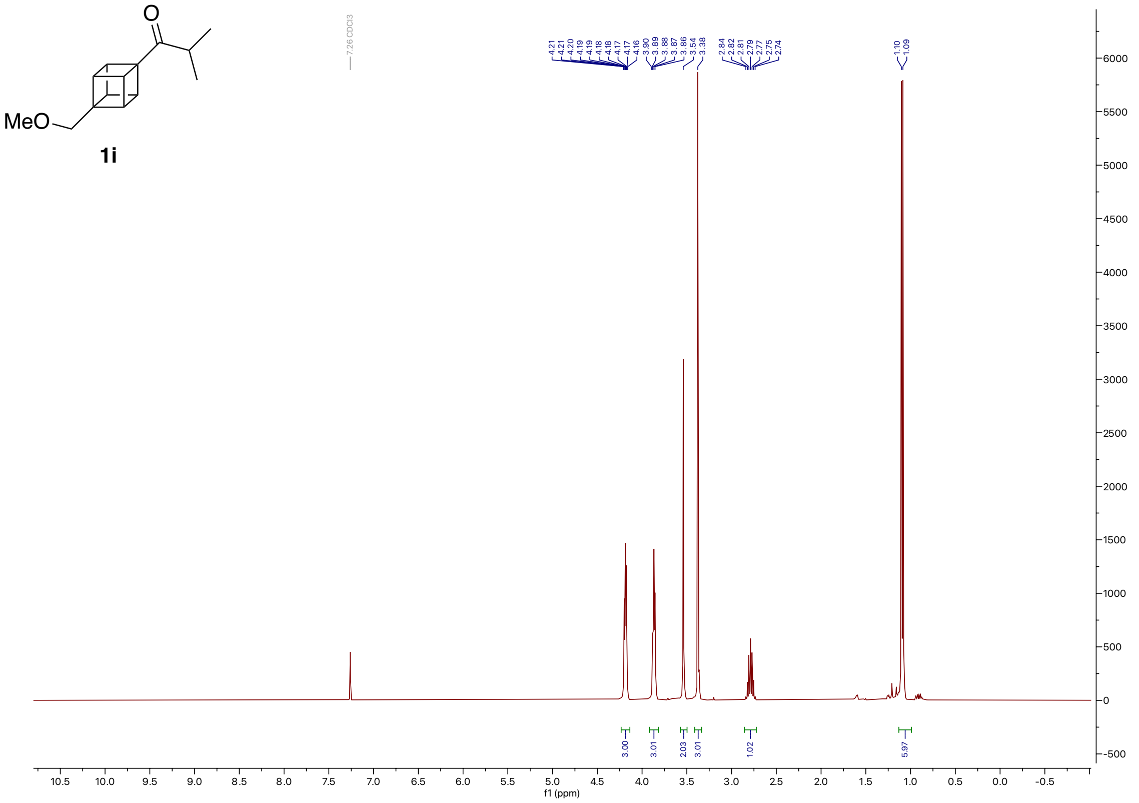

^{13}C NMR (101 MHz, $CDCl_3$) of 1i

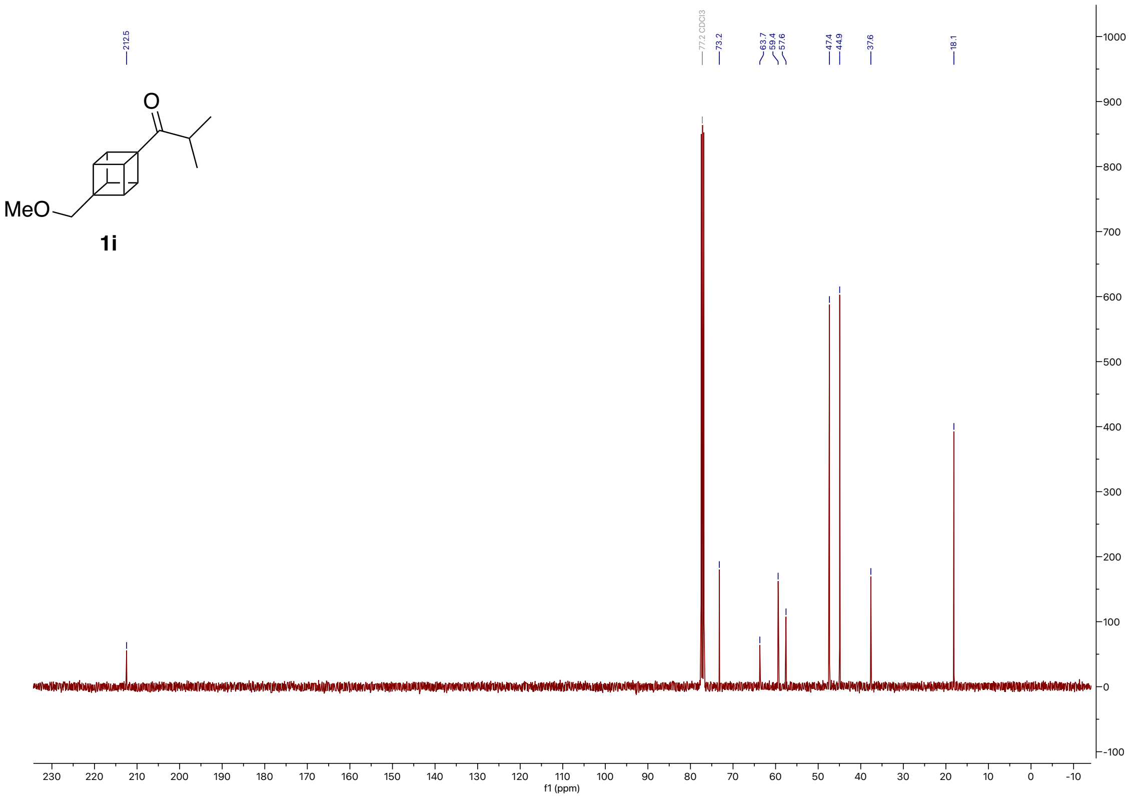

^{1}H NMR (500 MHz, CDCl$_3$) of S22

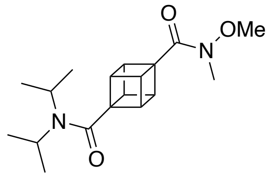

S22

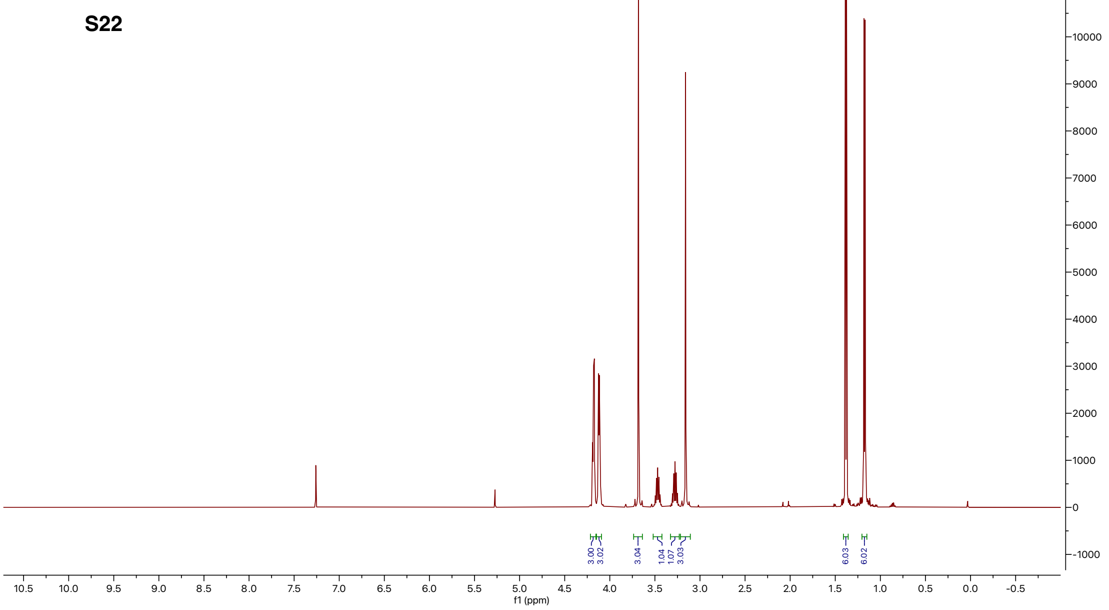

^{13}C NMR (126 MHz, CDCl$_3$) of S22

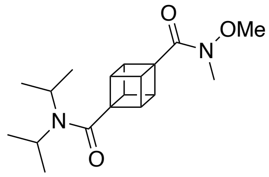

S22

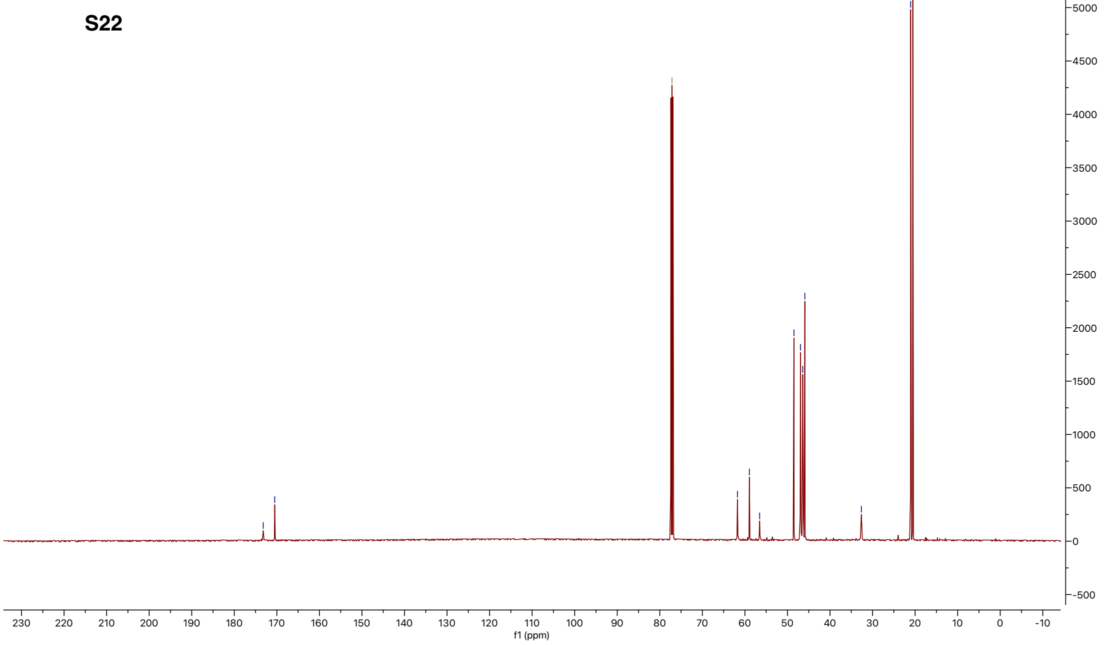

^{1}H NMR (400 MHz, $CDCl_3$) of 1j

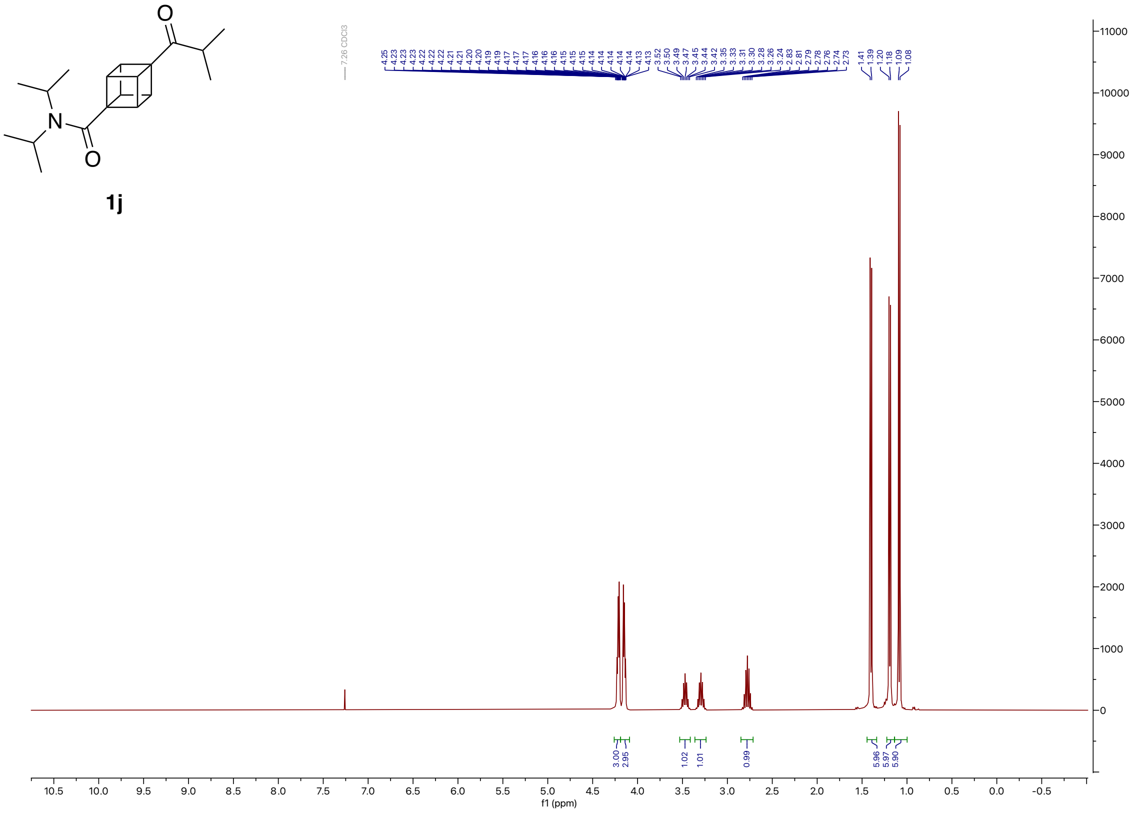

^{13}C NMR (101 MHz, $CDCl_3$) of 1j

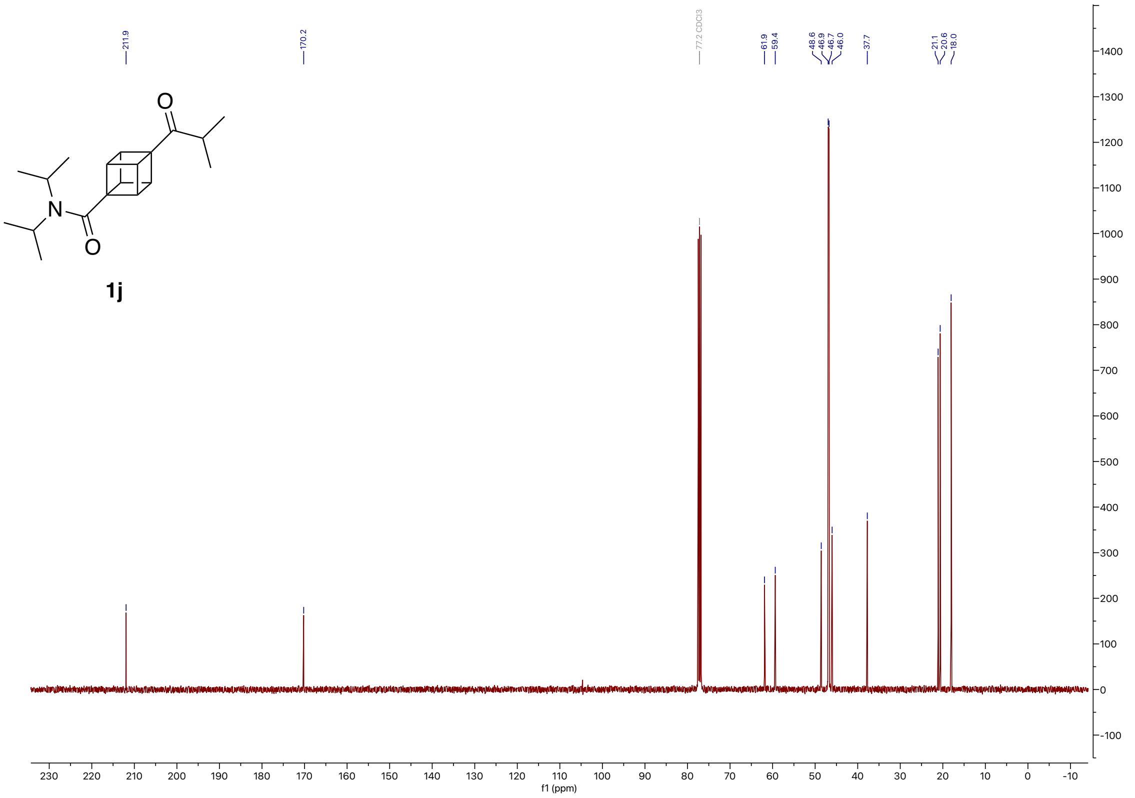

^{1}H NMR (400 MHz, $CDCl_3$) of S25

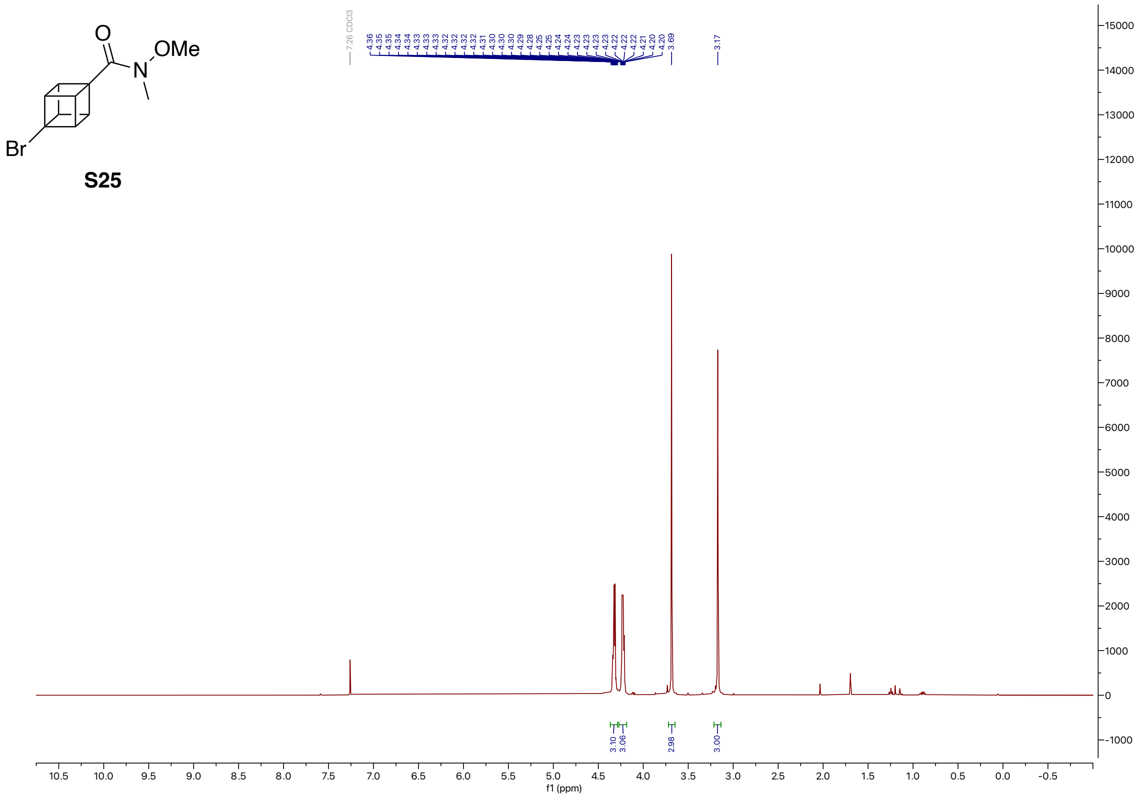

^{13}C NMR (101 MHz, $CDCl_3$) of S25

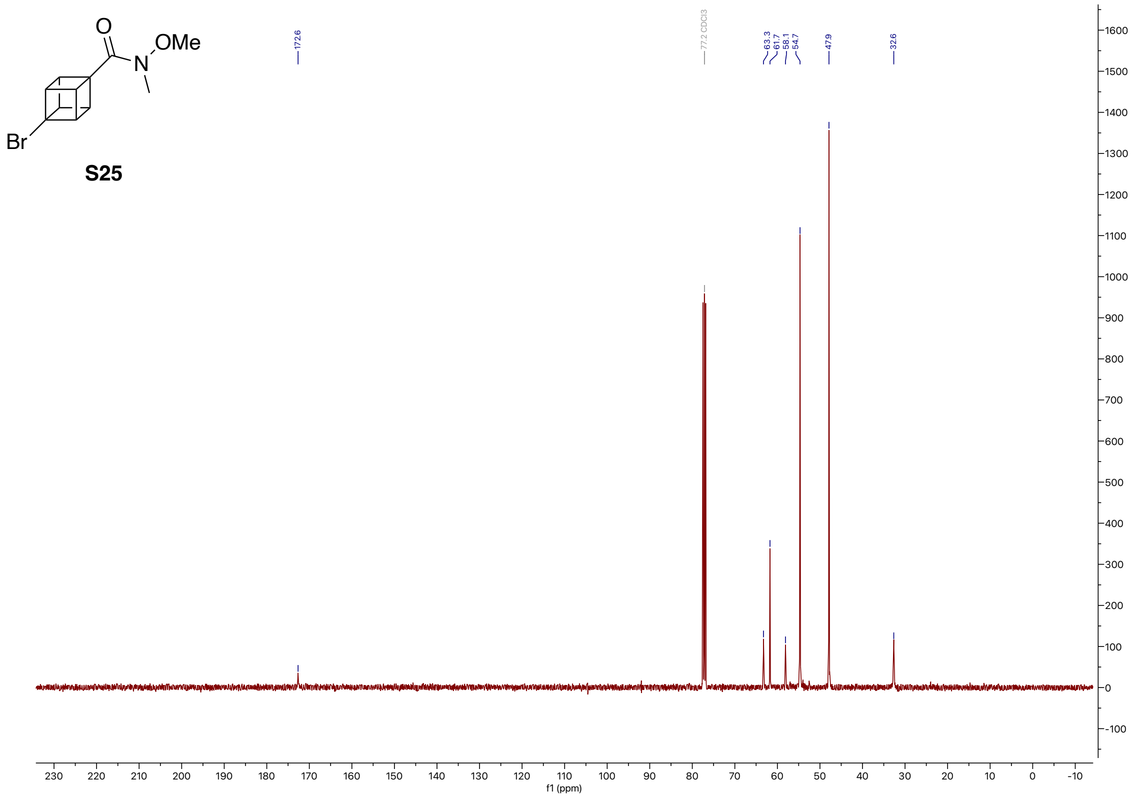

^{1}H NMR (500 MHz, $CDCl_3$) of 1k

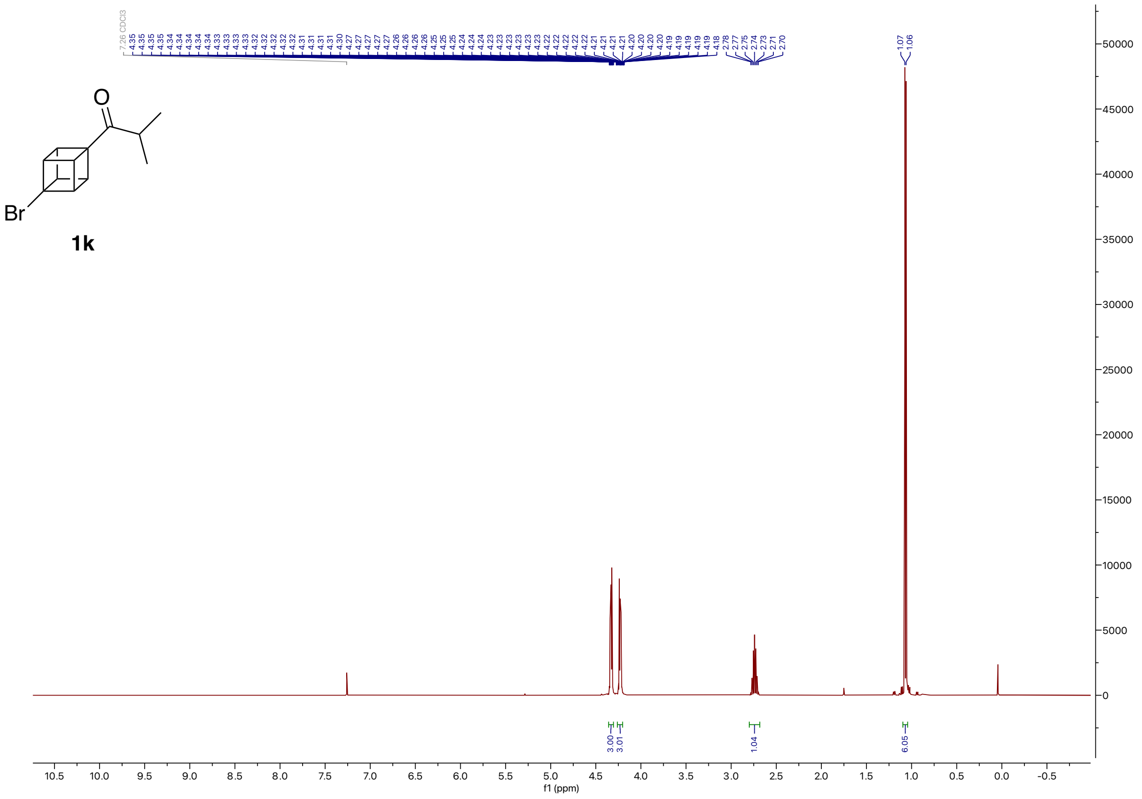

^{13}C NMR (126 MHz, $CDCl_3$) of 1k

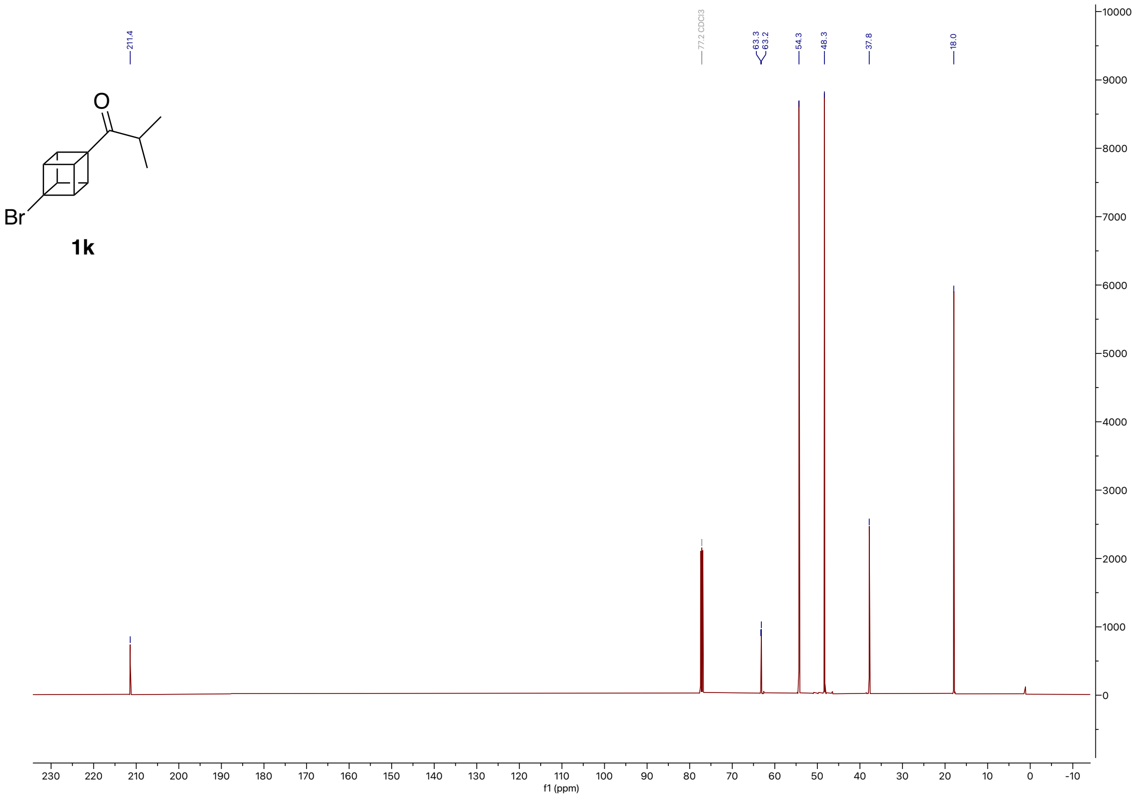

^{1}H NMR (500 MHz, $CDCl_3$) of S26

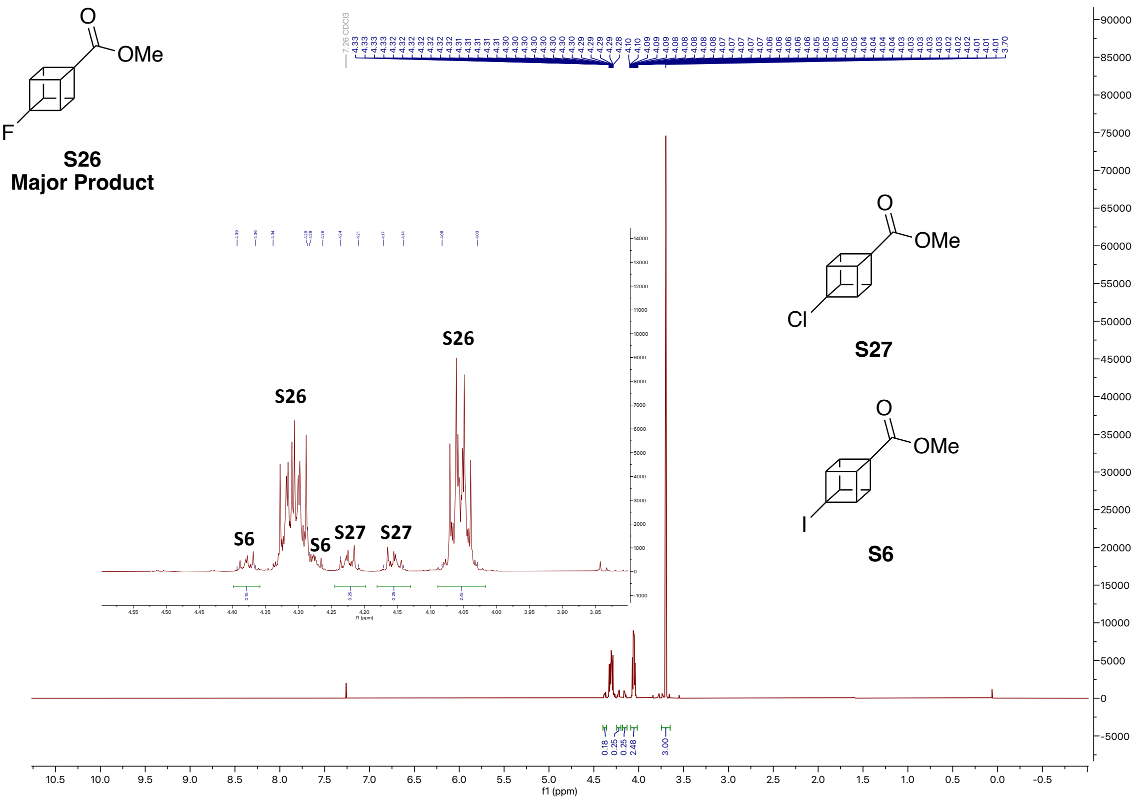

^{13}C NMR (126 MHz, $CDCl_3$) of S26

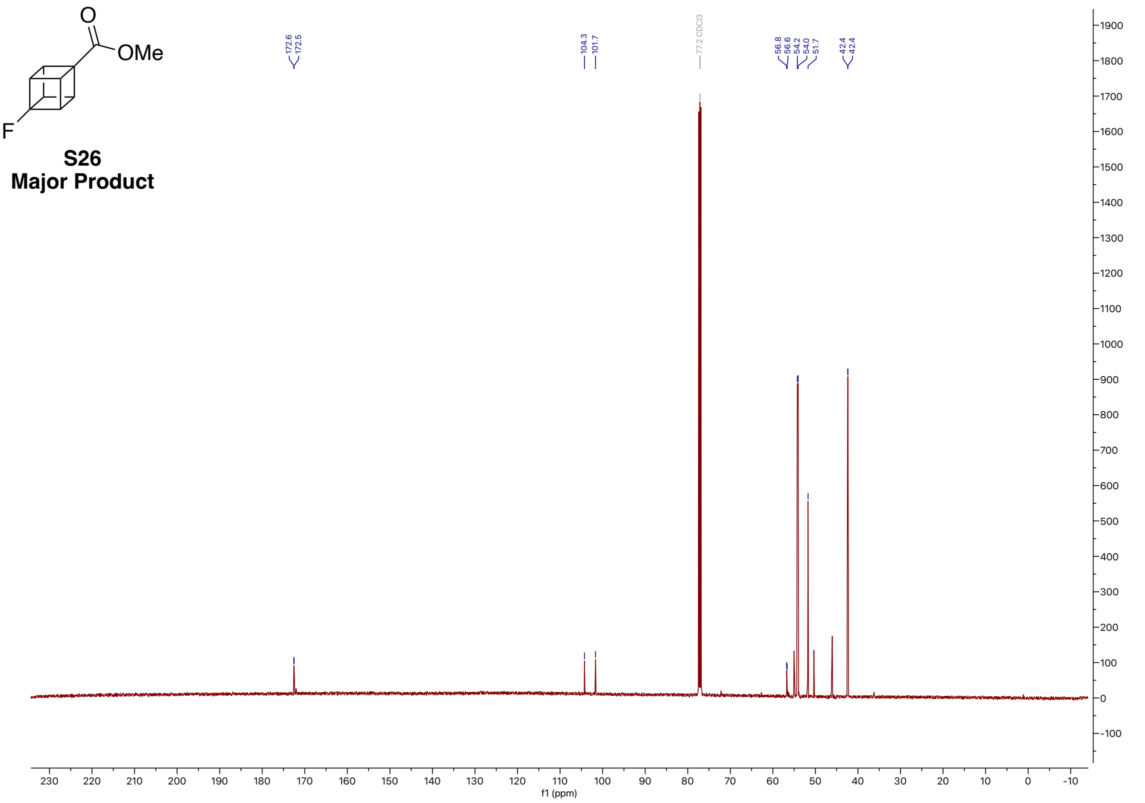

^{13}C NMR (126 MHz, $CDCl_3$) of S26&S27&S6

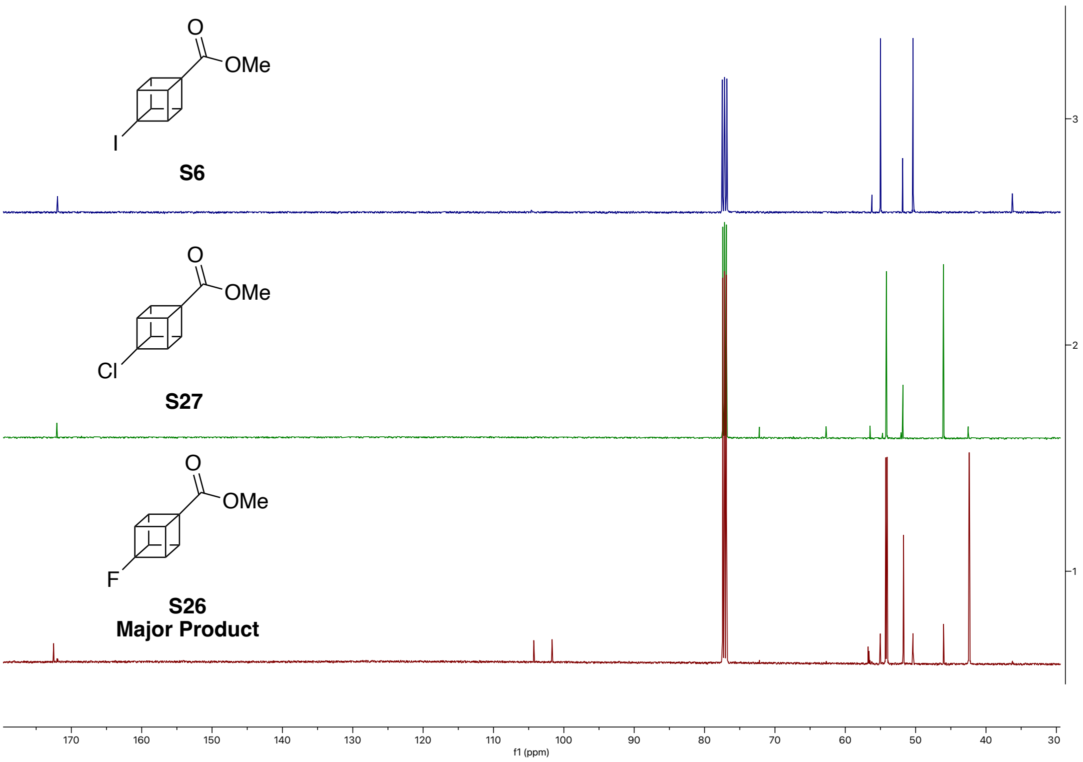

^{13}C NMR (126 MHz, $CDCl_3$) of S26&S27&S6 zoom

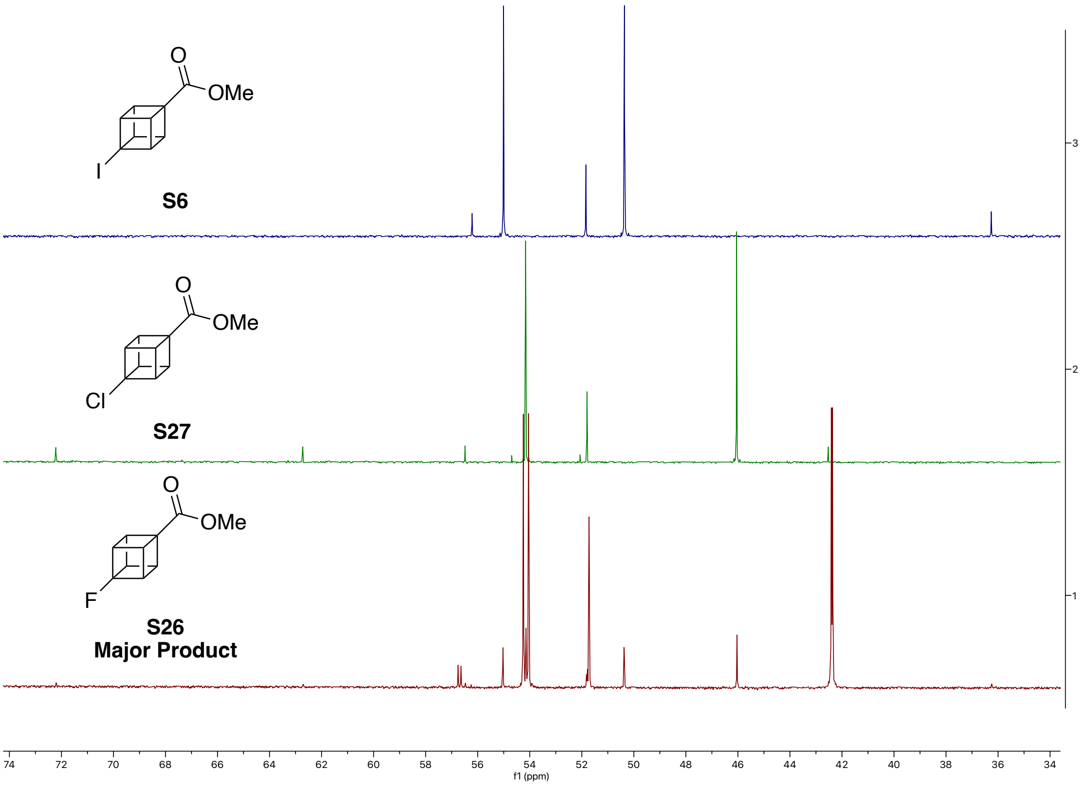

^{19}F{1H}NMR (471 MHz, $CDCl_3$) of S26

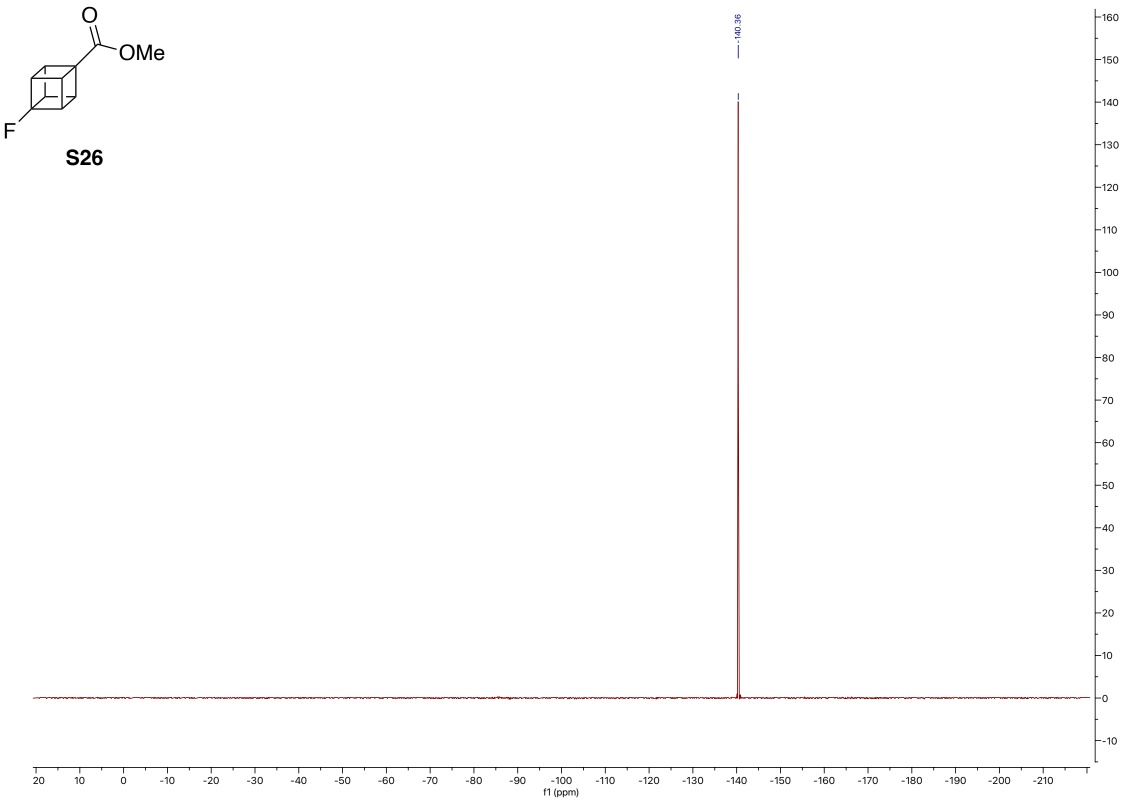

^{1}H NMR (500 MHz, $CDCl_3$) of S27

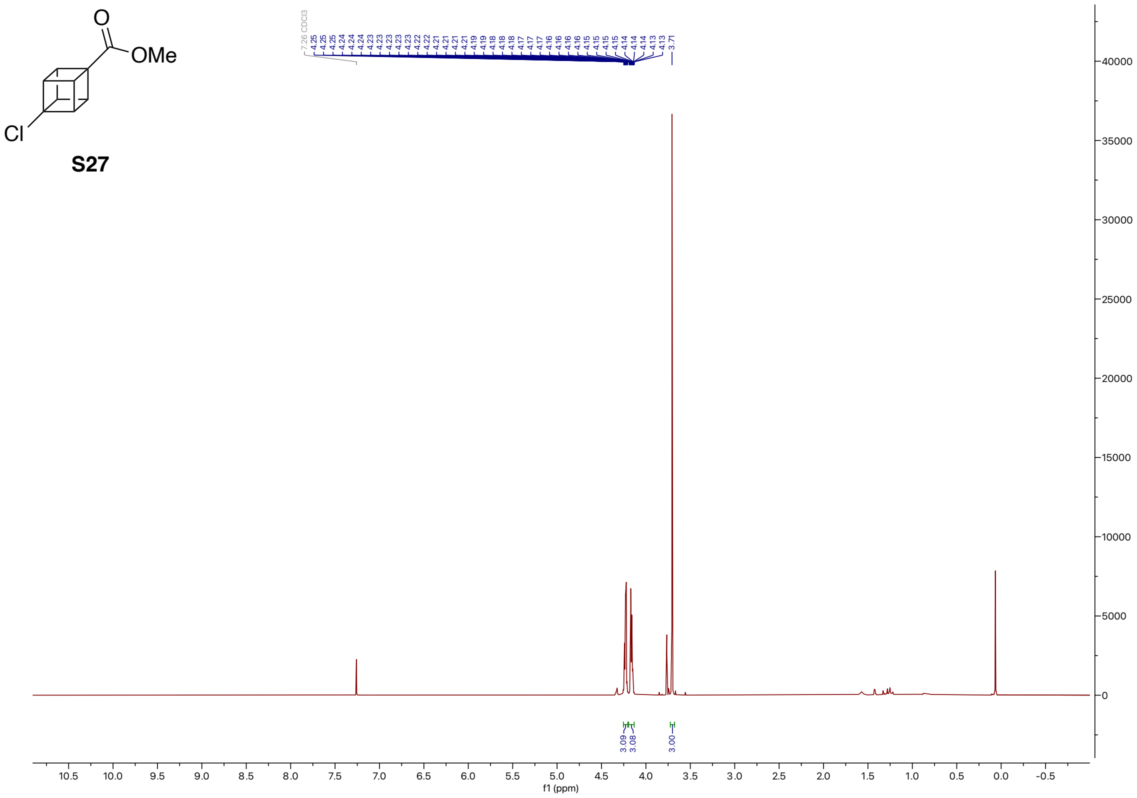

^{13}C NMR (126 MHz, $CDCl_3$) of S27

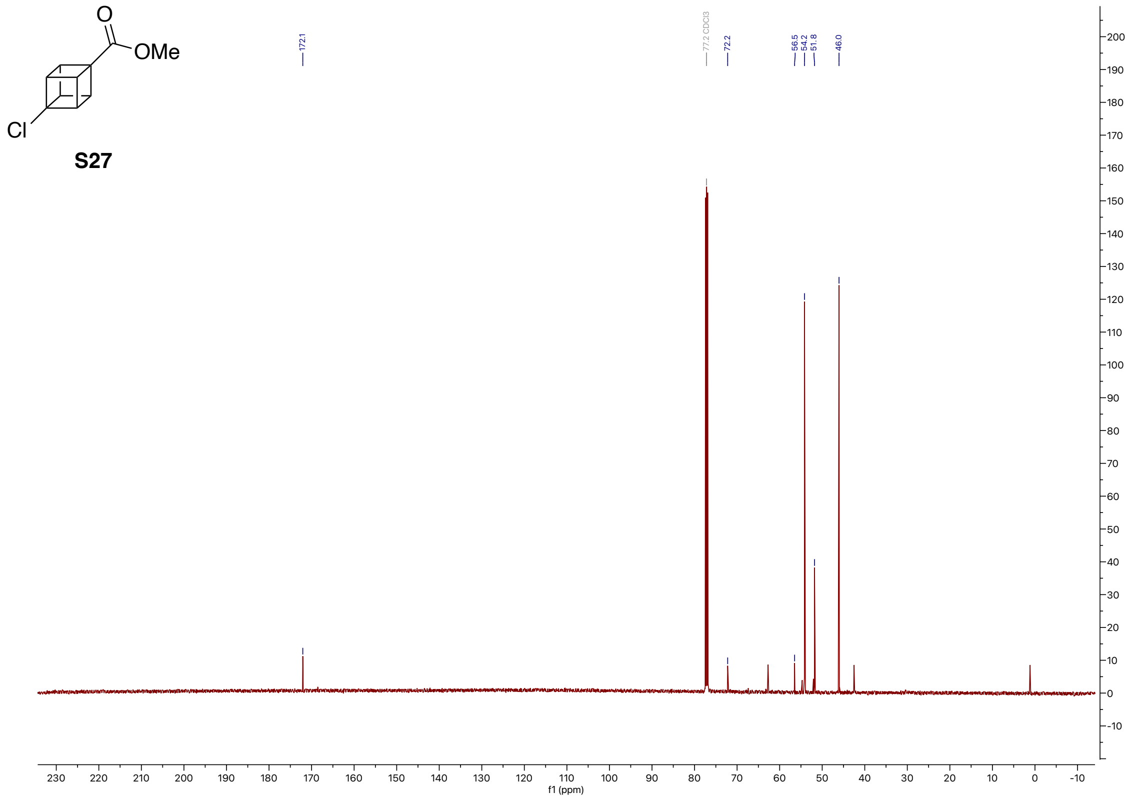

^{1}H NMR (400 MHz, $CDCl_3$) of S28

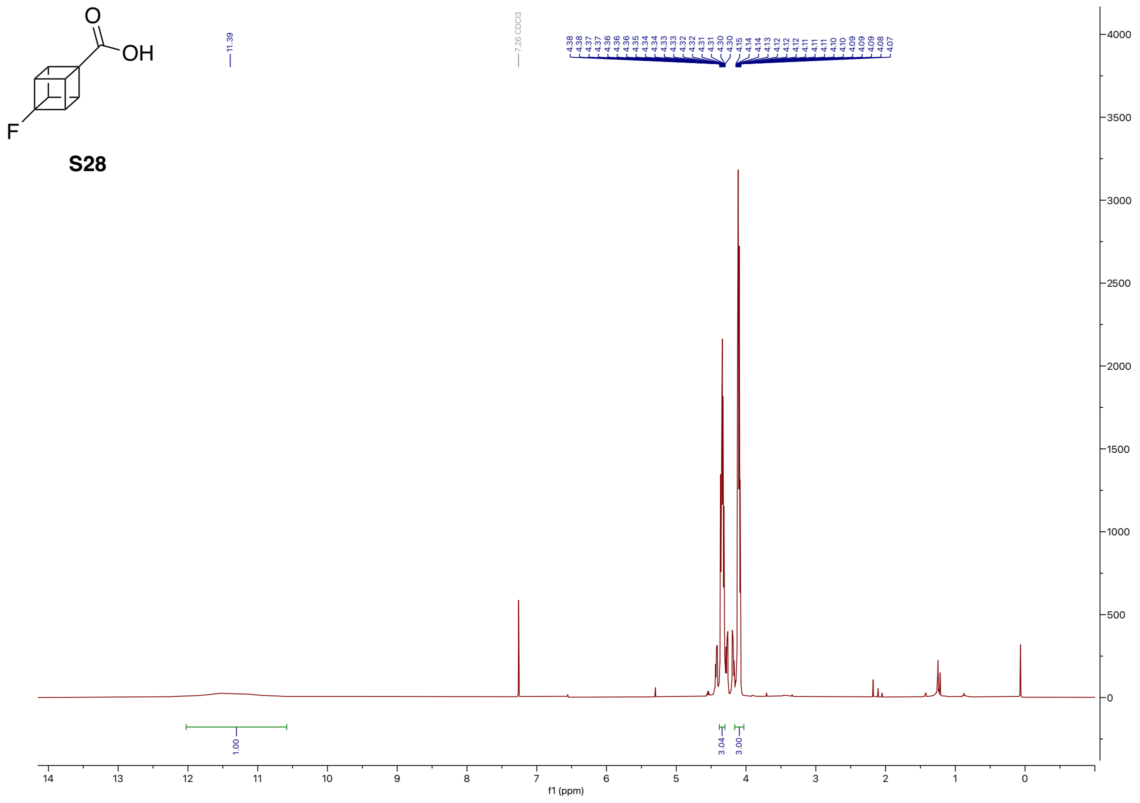

^{13}C NMR (101 MHz, $CDCl_3$) of S28

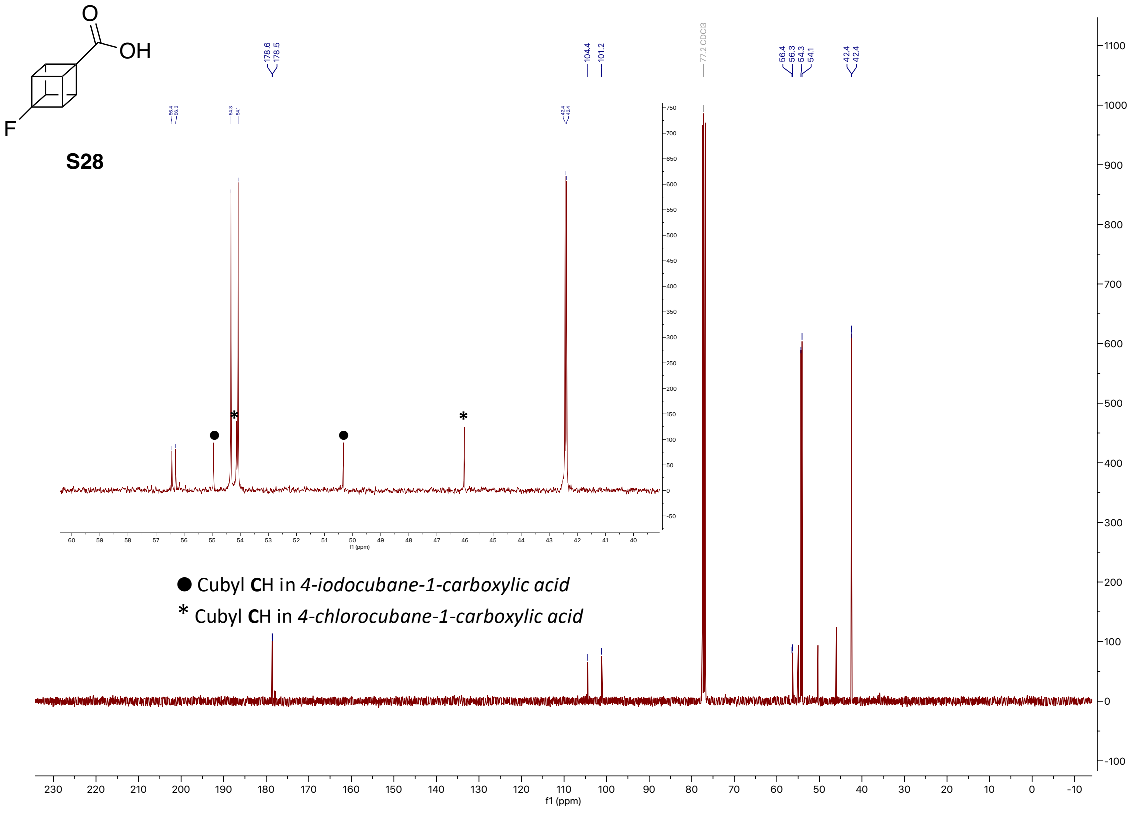

$^{19}F\{1H\}$NMR (376 MHz, $CDCl_3$) of S28

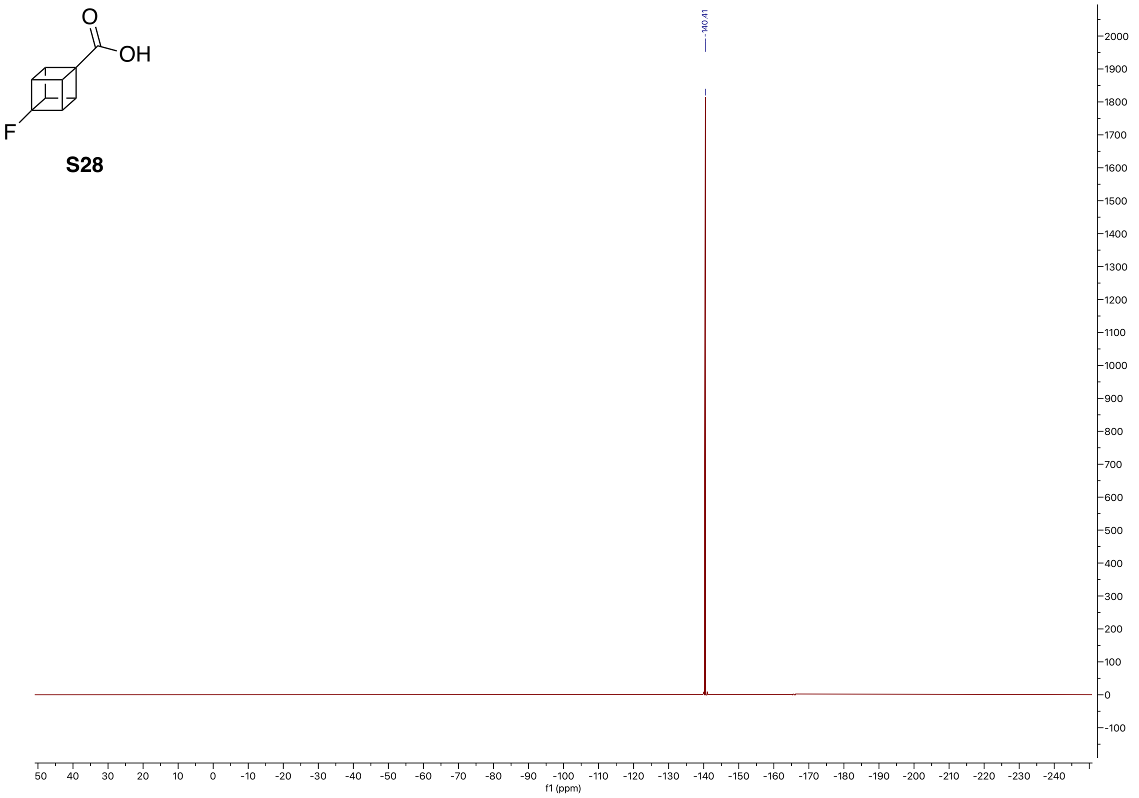

^1H NMR (500 MHz, $CDCl_3$) of S29

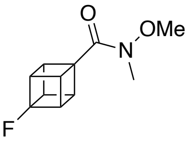

S29

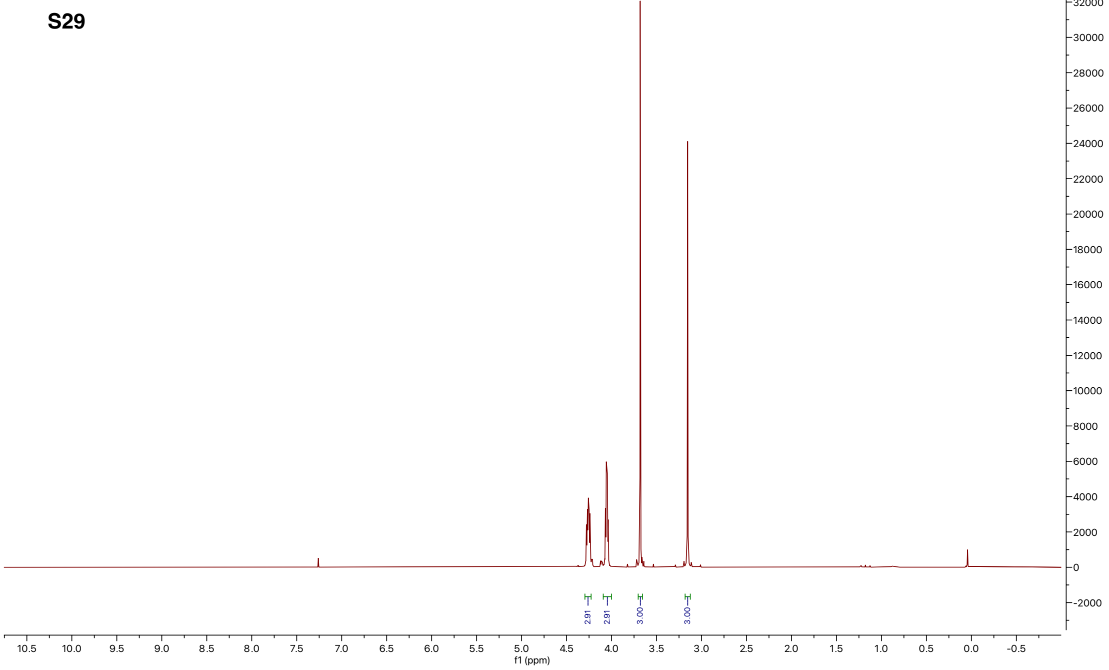

^{13}C NMR (126 MHz, $CDCl_3$) of S29

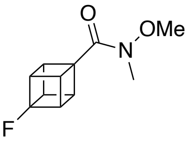

S29

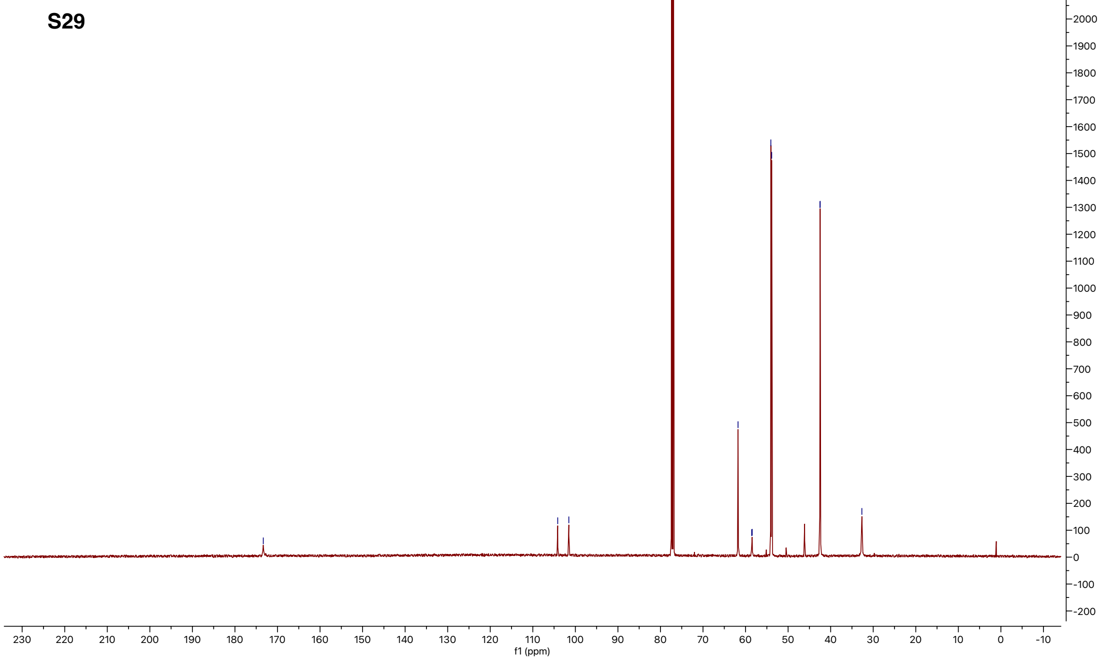

^{19}F{1H}NMR (471 MHz, CDCl$_3$) of S29

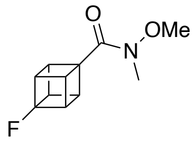

S29

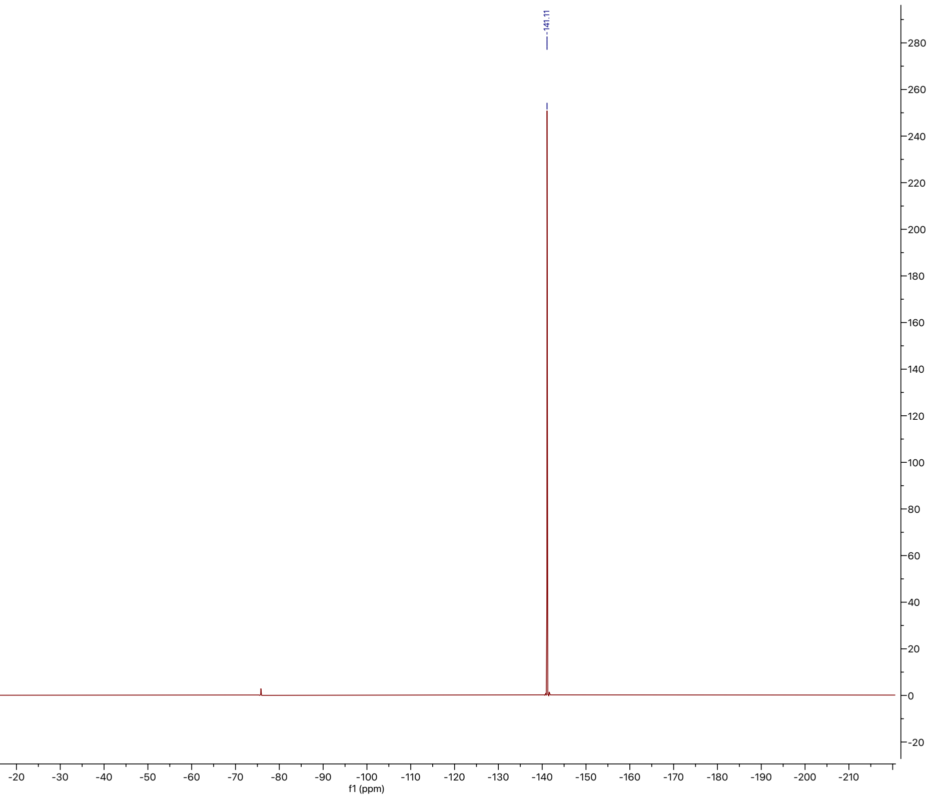

^{1}H NMR (400 MHz, CDCl$_3$) of 1l

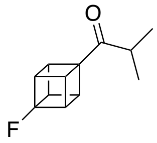

1l

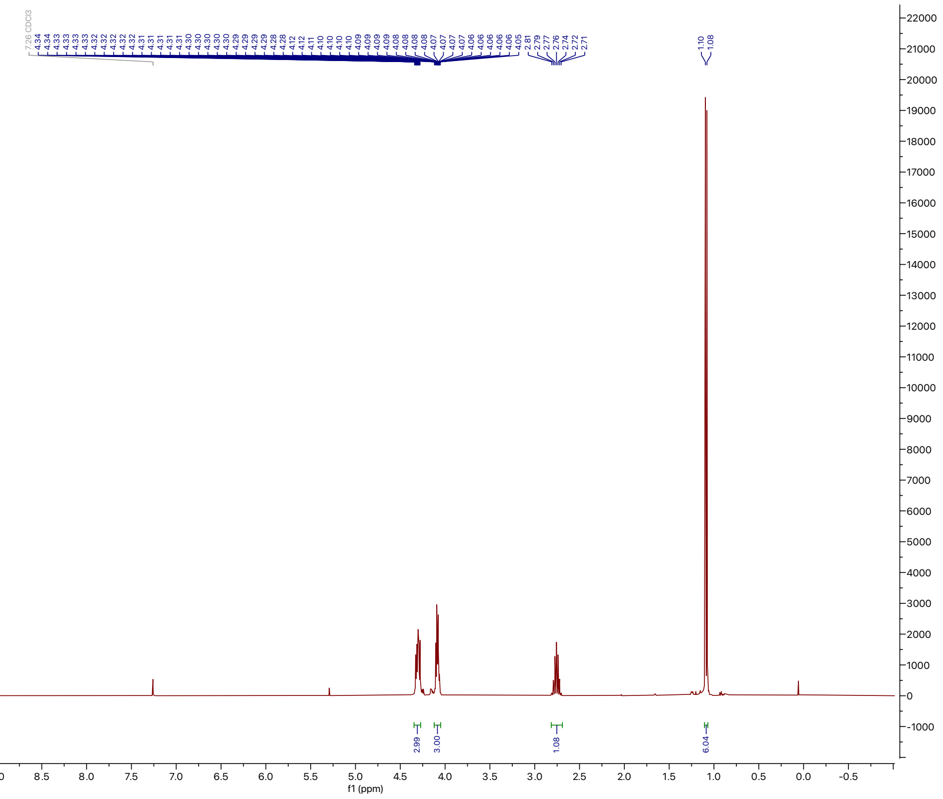

^{13}C NMR (101 MHz, $CDCl_3$) of 1l

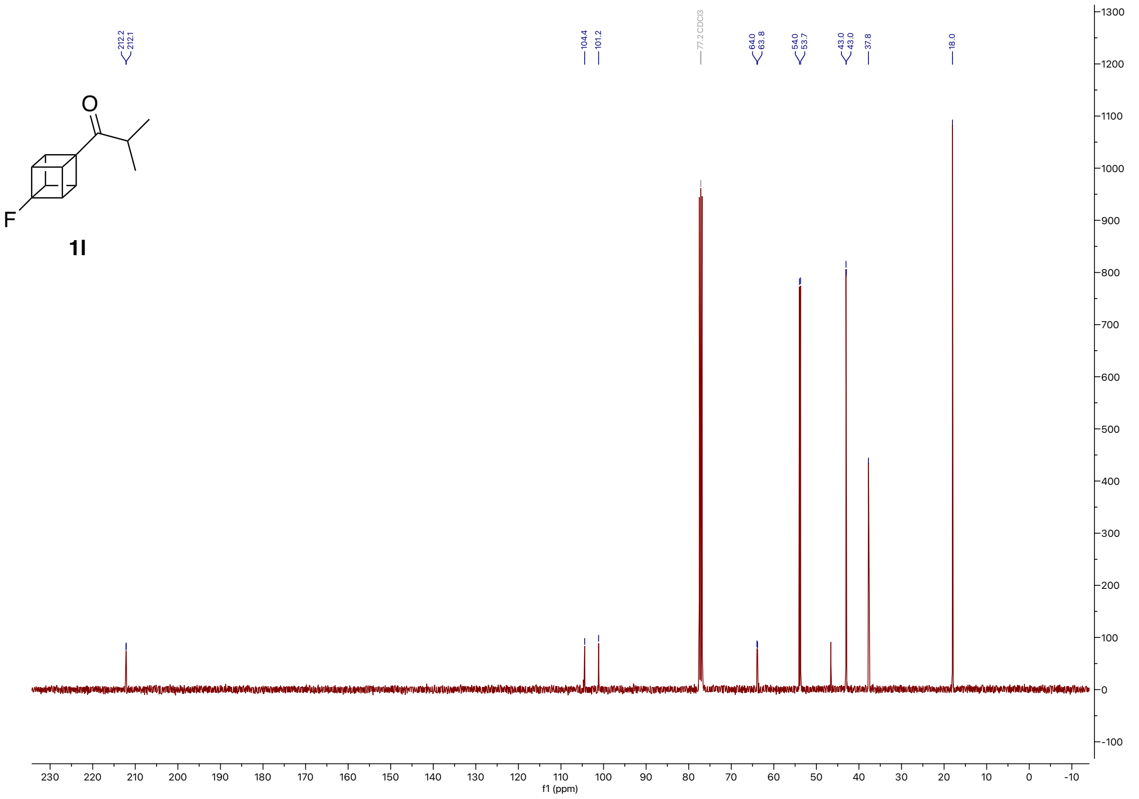

$^{19}F\{1H\}$NMR (376 MHz, $CDCl_3$) of 1l

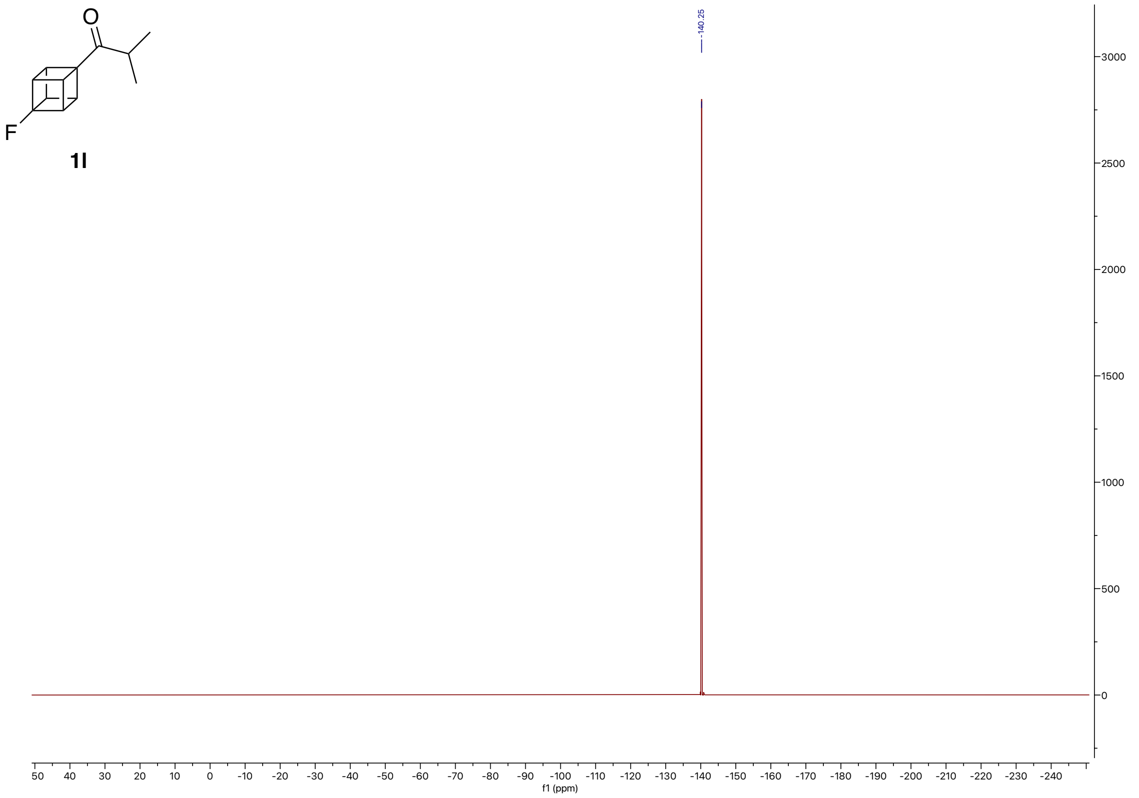

^{1}H NMR (500 MHz, $CDCl_3$) of 2a

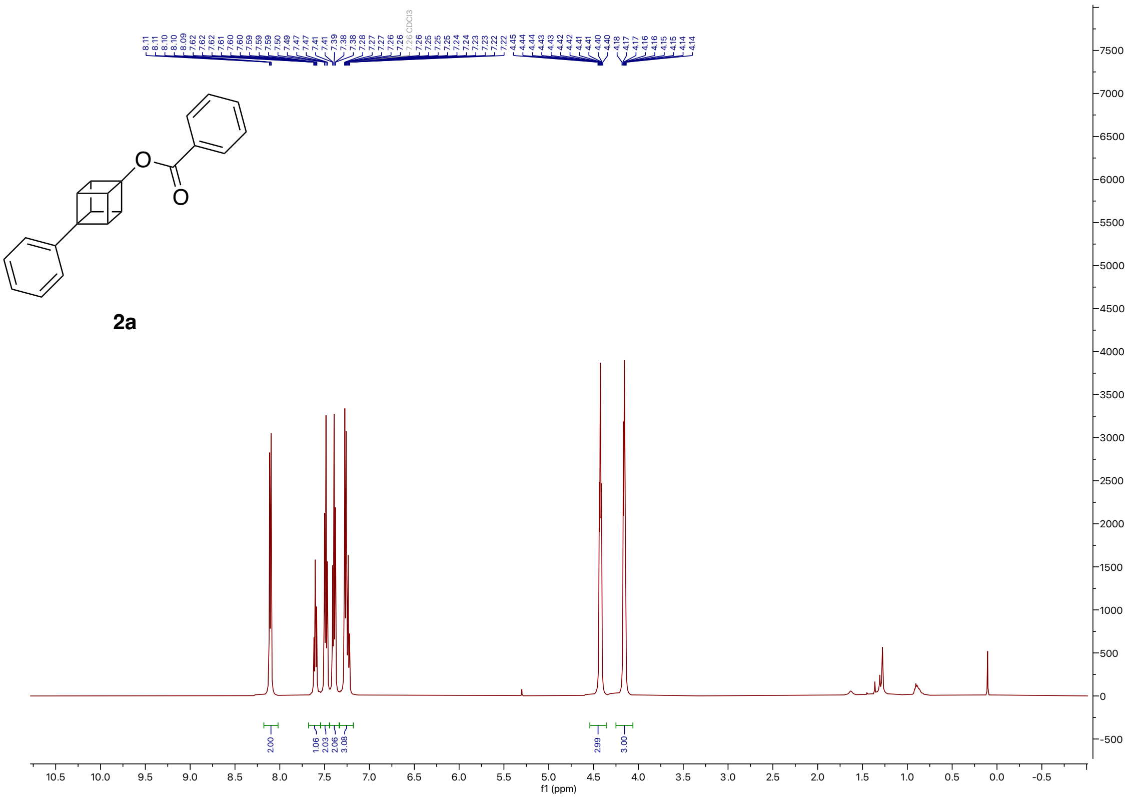

^{13}C NMR (126 MHz, $CDCl_3$) of 2a

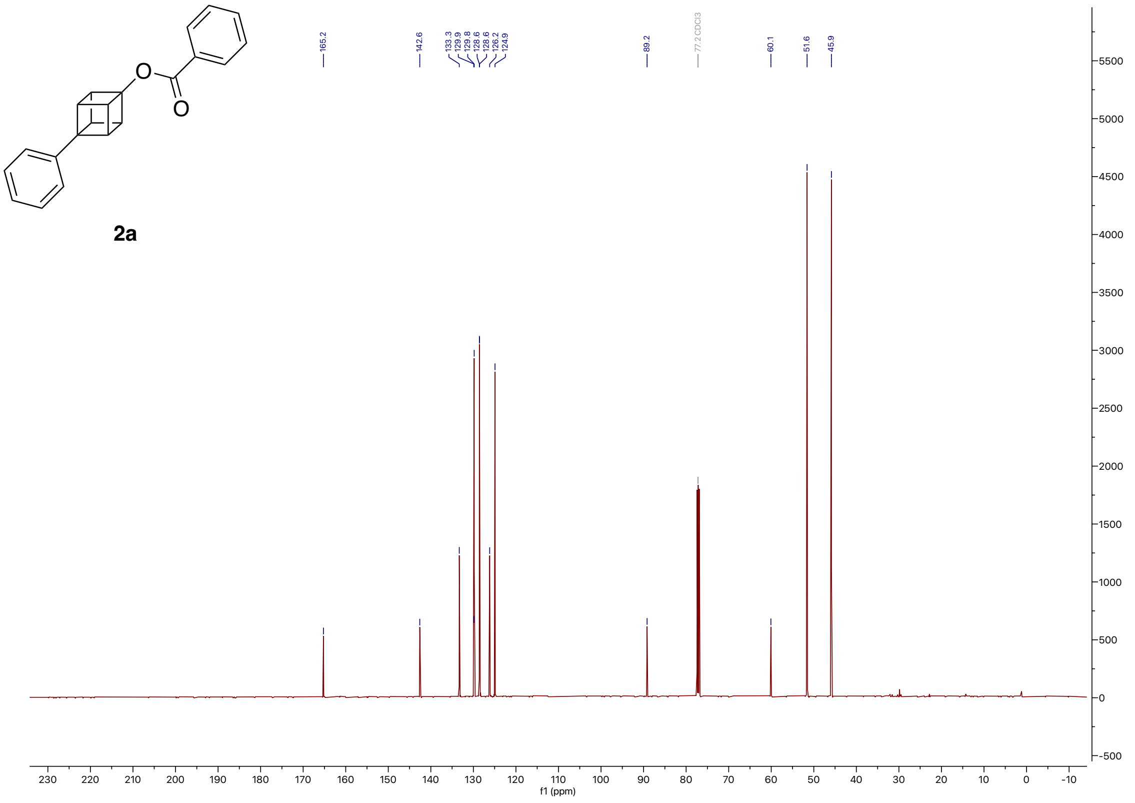

^{1}H NMR (400 MHz, $CDCl_3$) of 3a

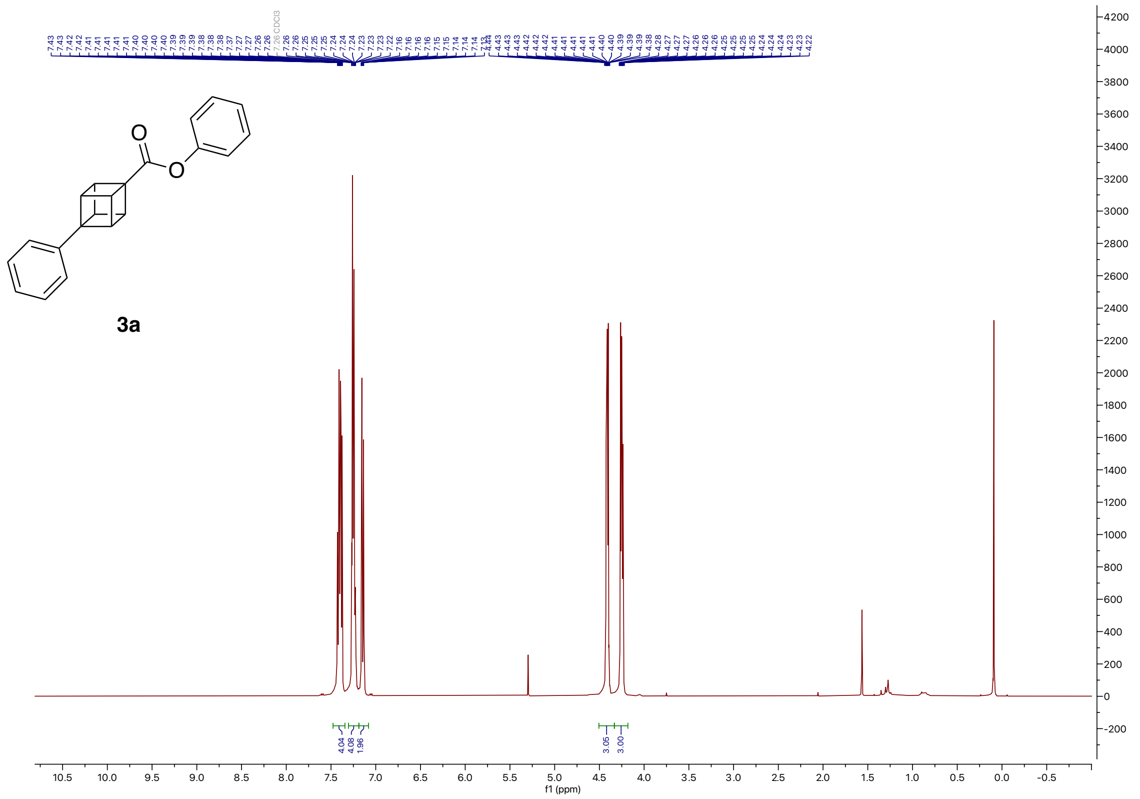

^{13}C NMR (101 MHz, $CDCl_3$) of 3a

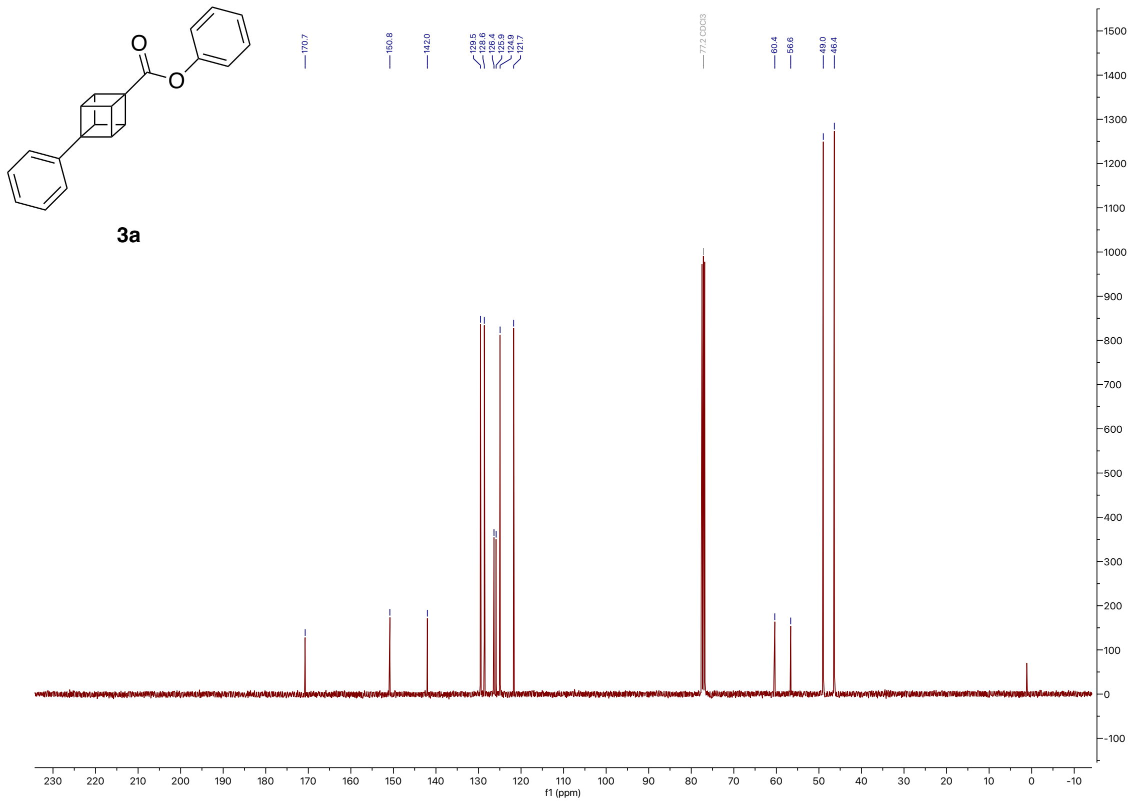

^{1}H NMR (400 MHz, $CDCl_3$) of 2b

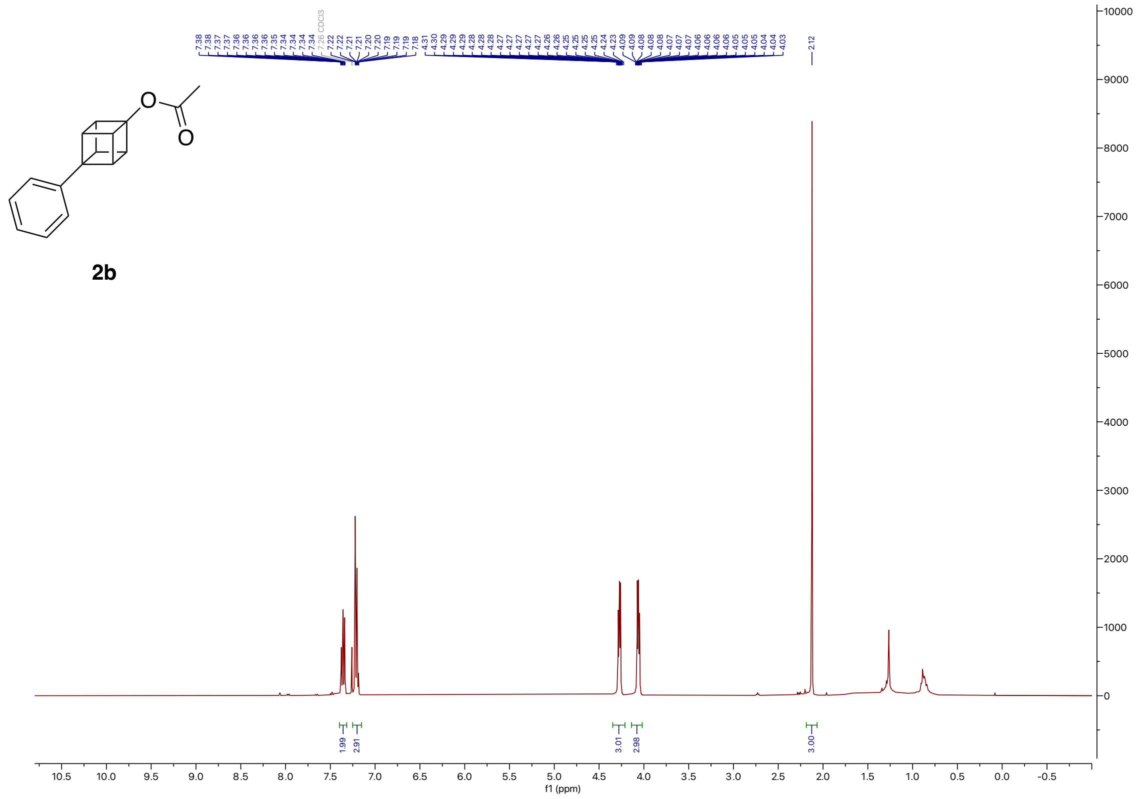

^{13}C NMR (101 MHz, $CDCl_3$) of 2b

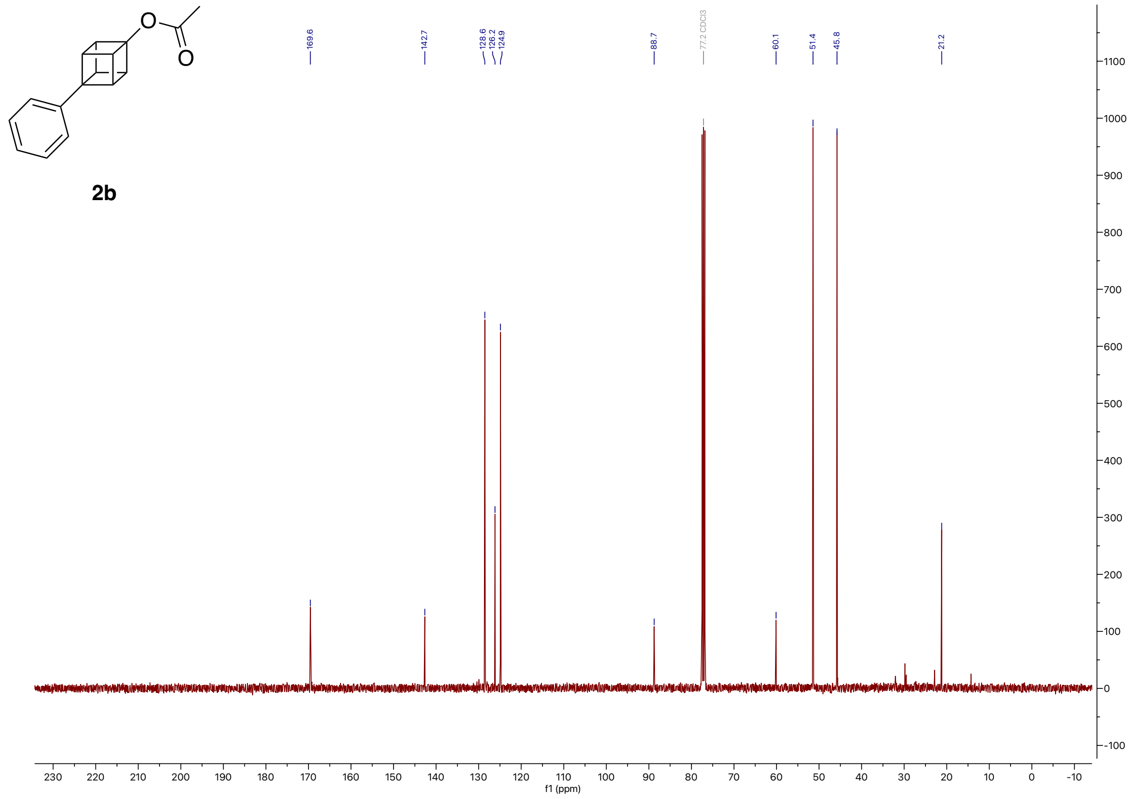

^{1}H NMR (400 MHz, $CDCl_3$) of 2c

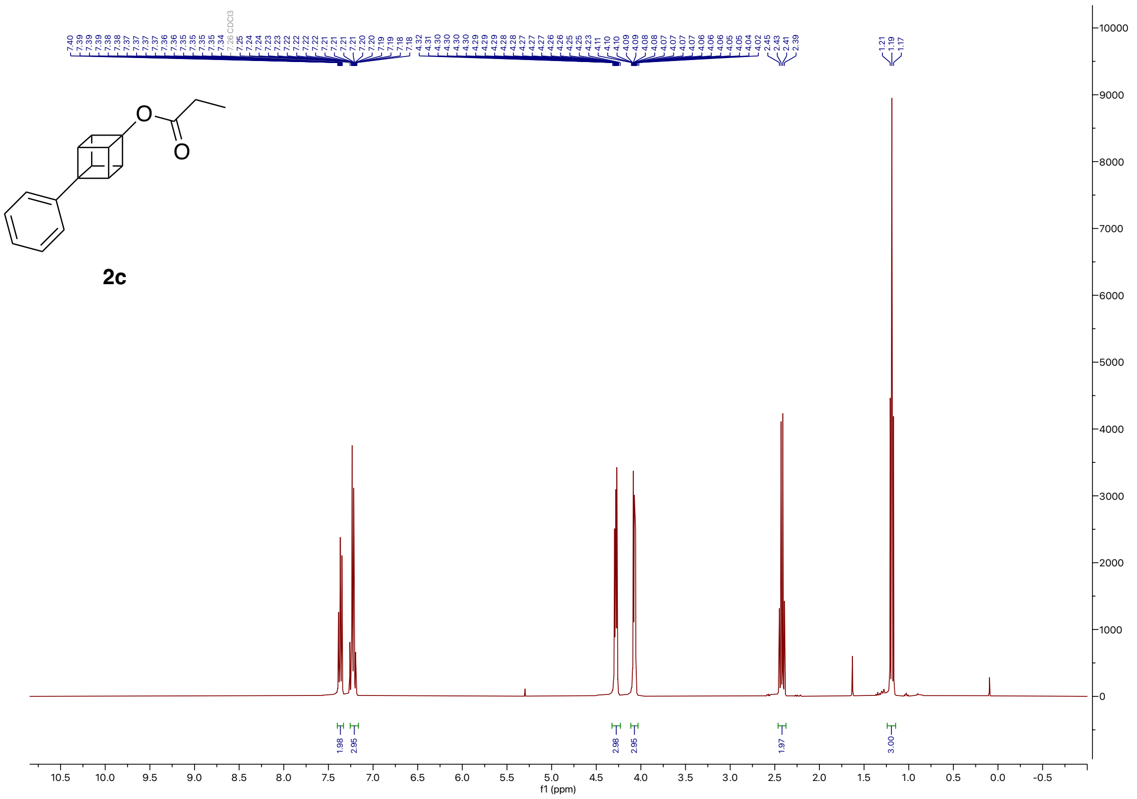

^{13}C NMR (101 MHz, $CDCl_3$) of 2c

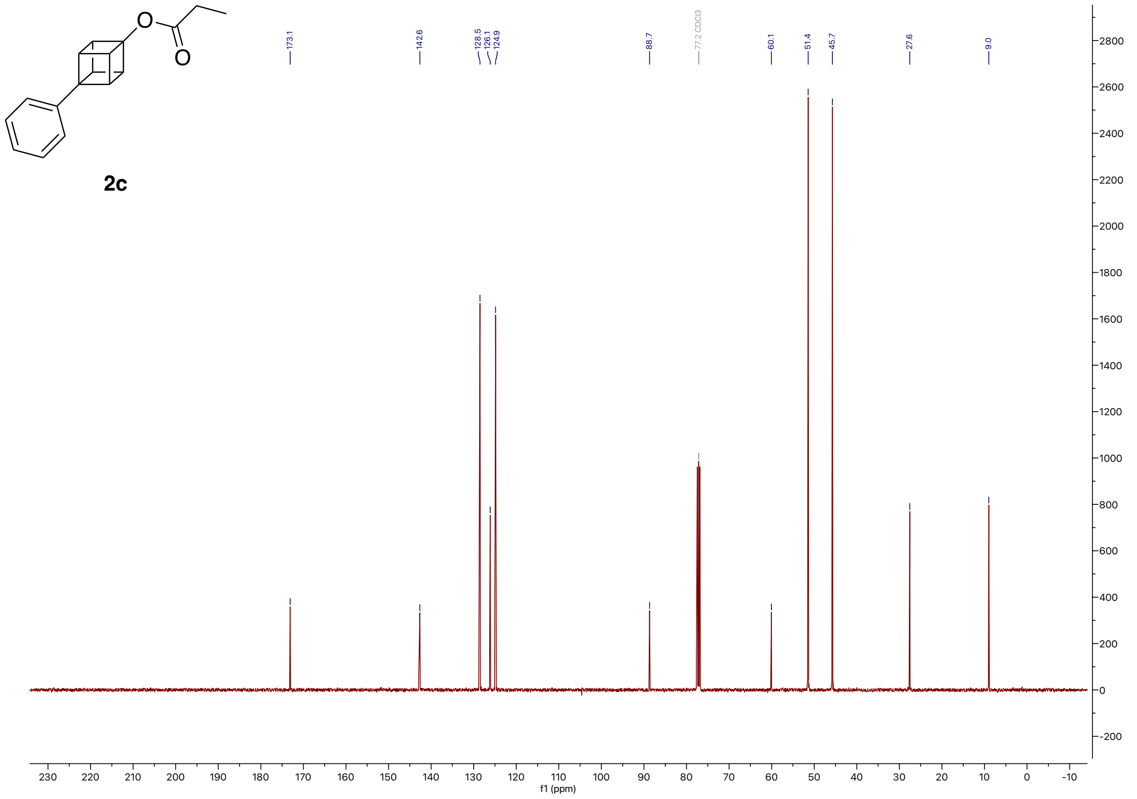

^{1}H NMR (400 MHz, $CDCl_3$) of 2d

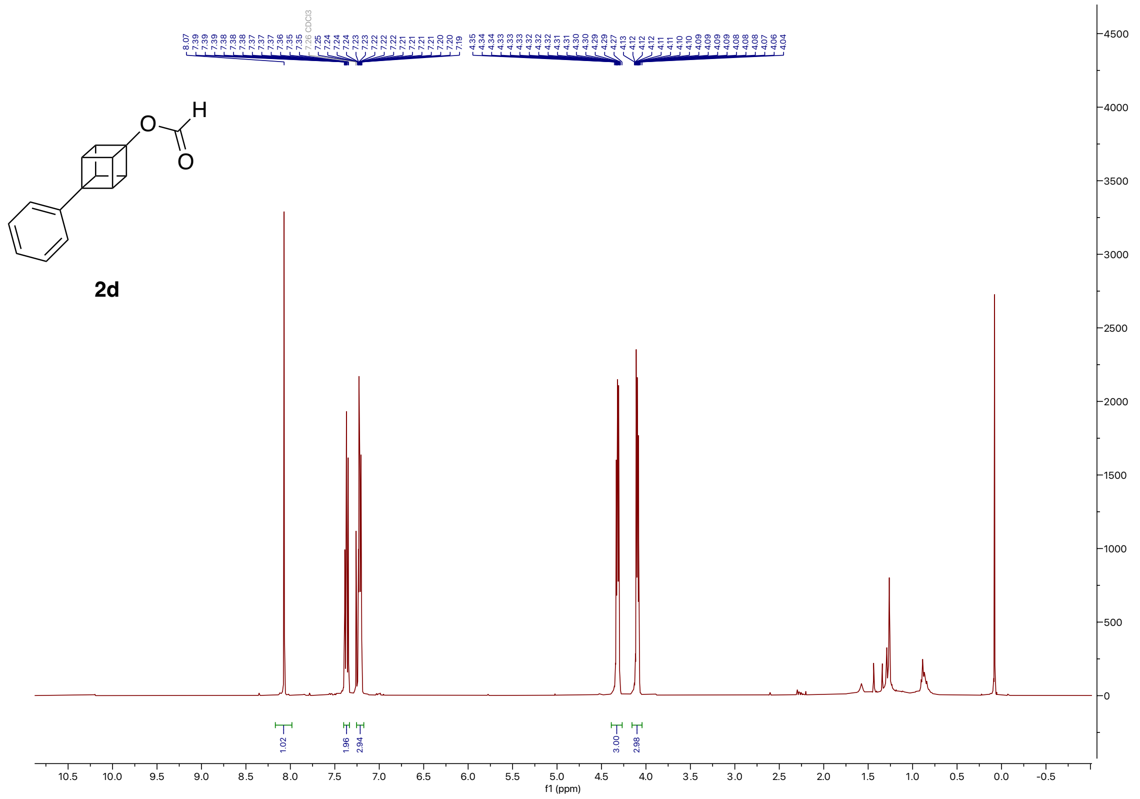

^{13}C NMR (101 MHz, $CDCl_3$) of 2d

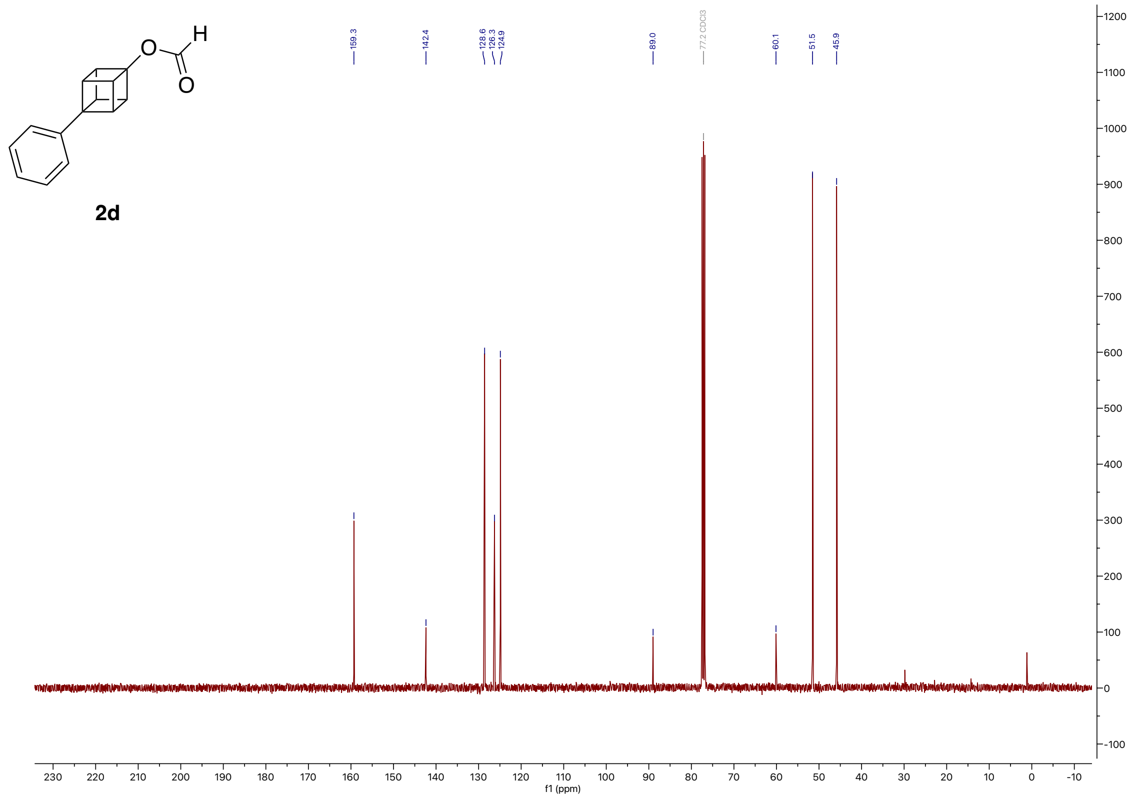

^{1}H NMR (400 MHz, $CDCl_3$) of 2e

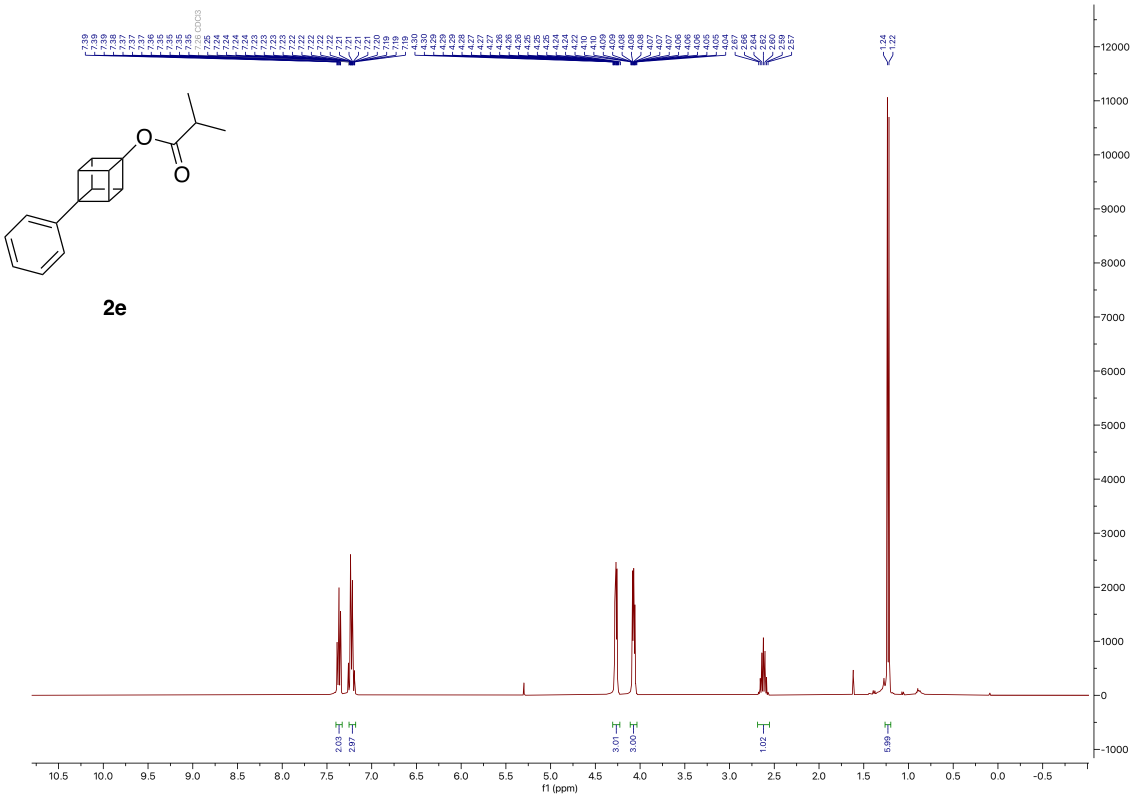

^{13}C NMR (101 MHz, $CDCl_3$) of 2e

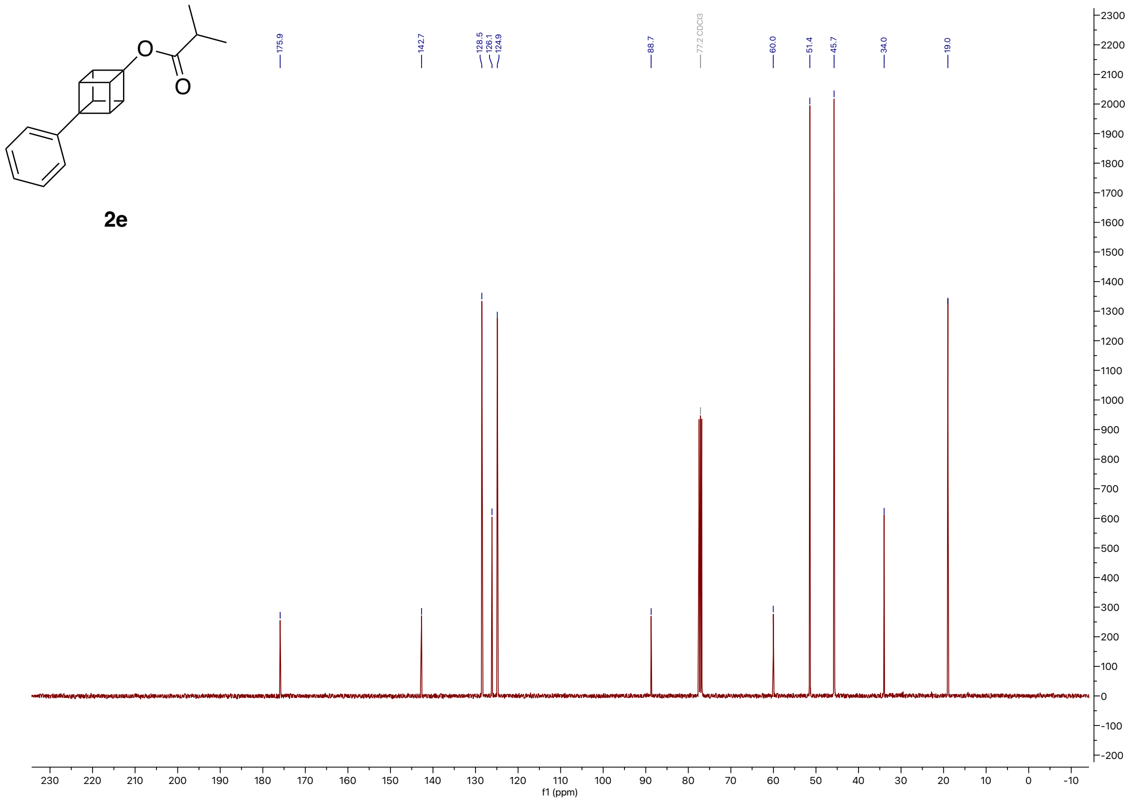

^{1}H NMR (400 MHz, $CDCl_3$) of 3e

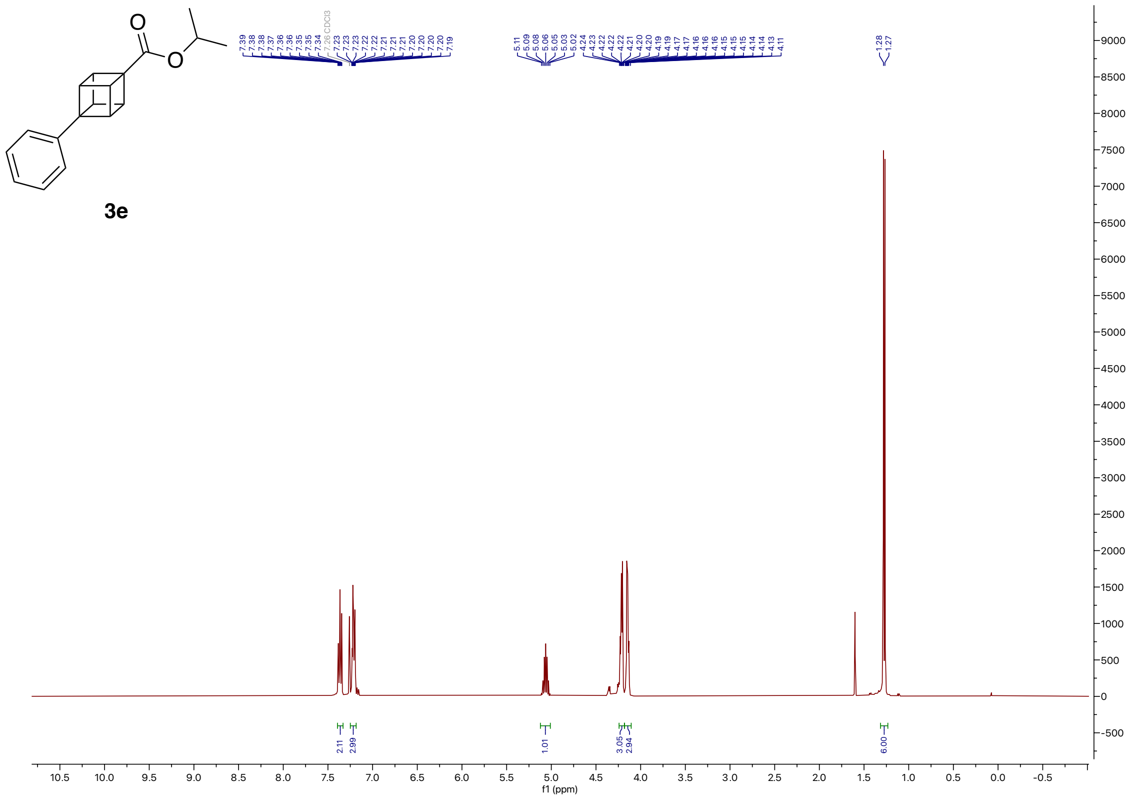

^{13}C NMR (101 MHz, $CDCl_3$) of 3e

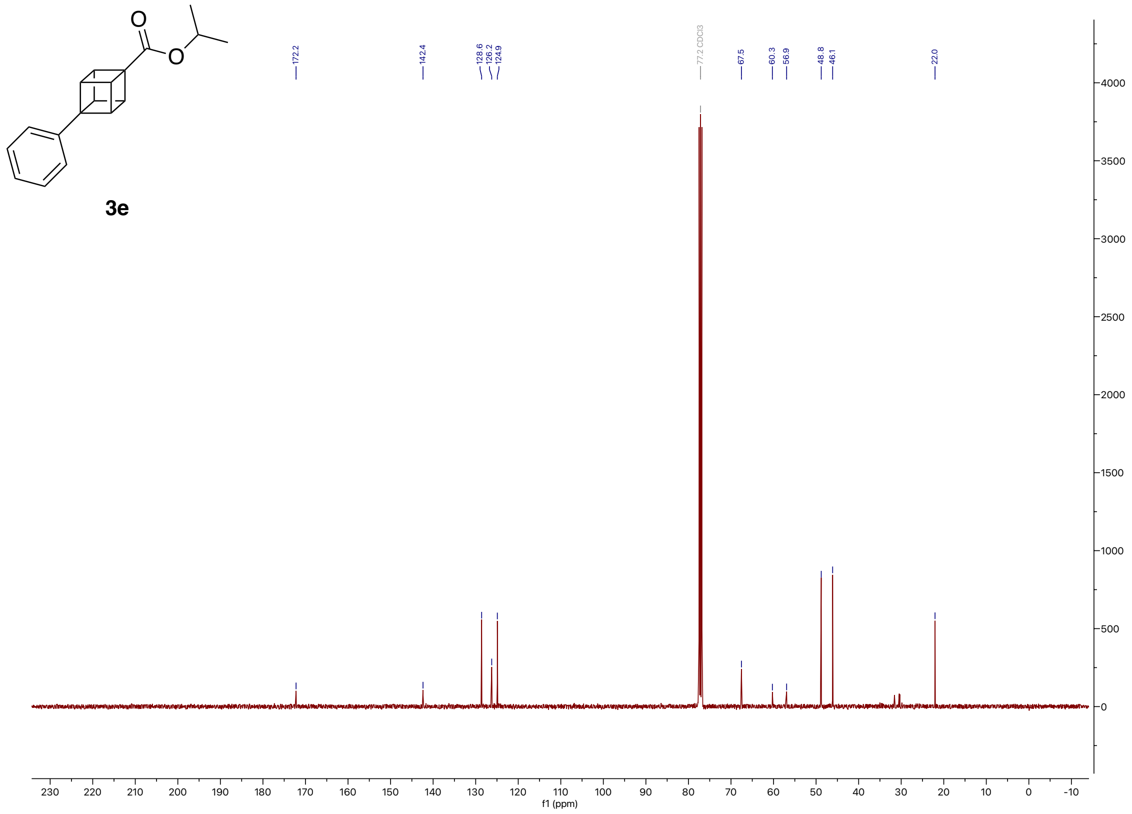

^{1}H NMR (400 MHz, $CDCl_3$) of 3f mixed with 1f in a ratio of 1:9

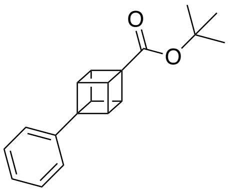

3f

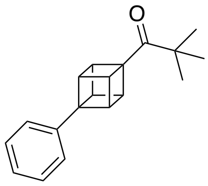

1f

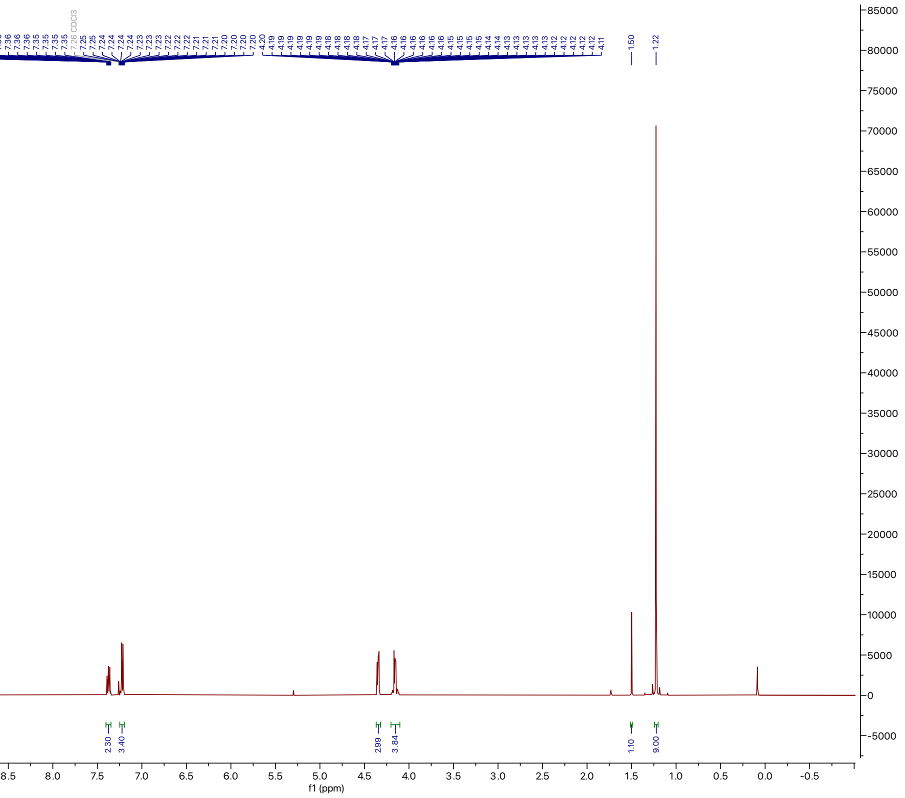

^{13}C NMR (101 MHz, $CDCl_3$) of 3f mixed with 1f in a ratio of 1:9

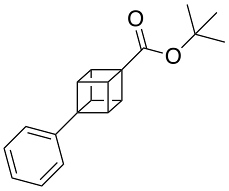

3f

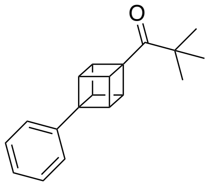

1f

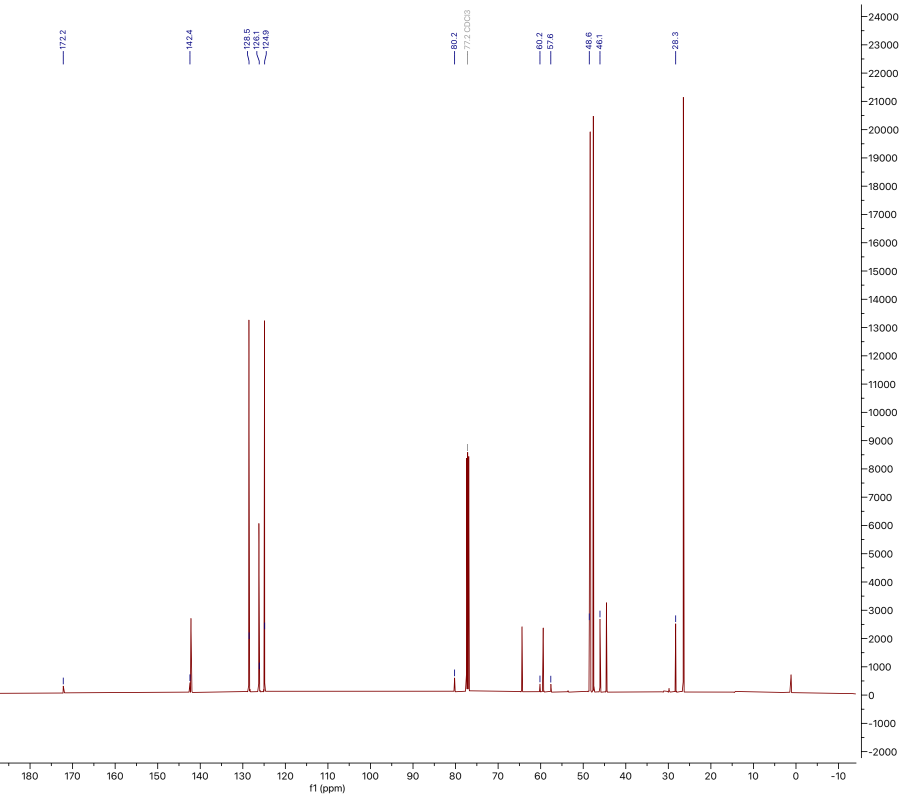

^{1}H NMR (400 MHz, $CDCl_3$) of 2g

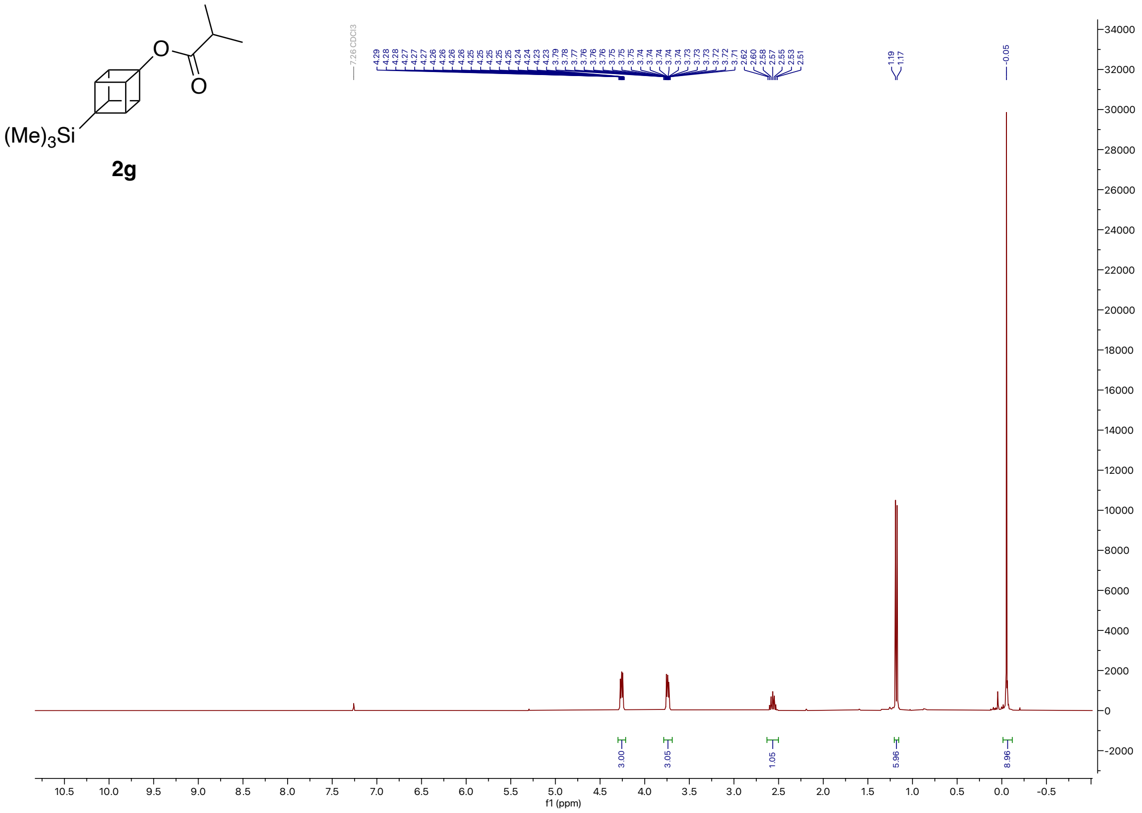

^{13}C NMR (101 MHz, $CDCl_3$) of 2g

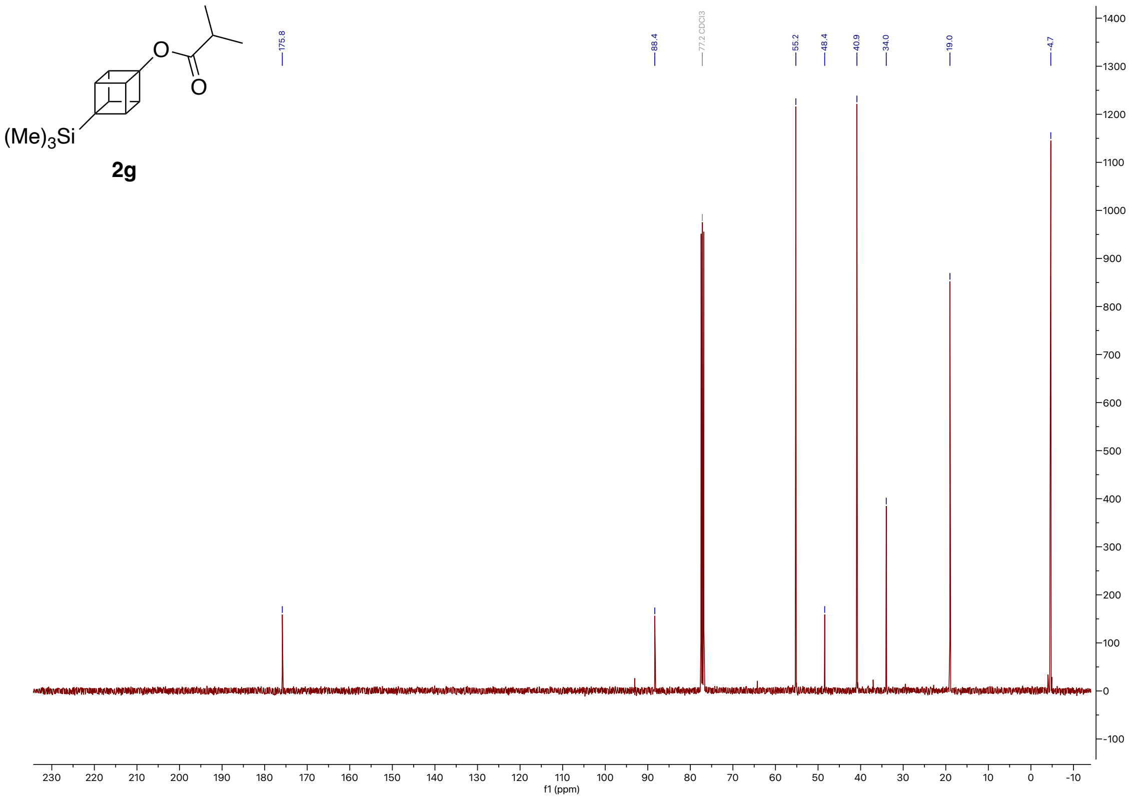

^{1}H NMR (400 MHz, $CDCl_3$) of 3g

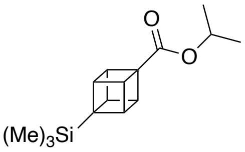

3g

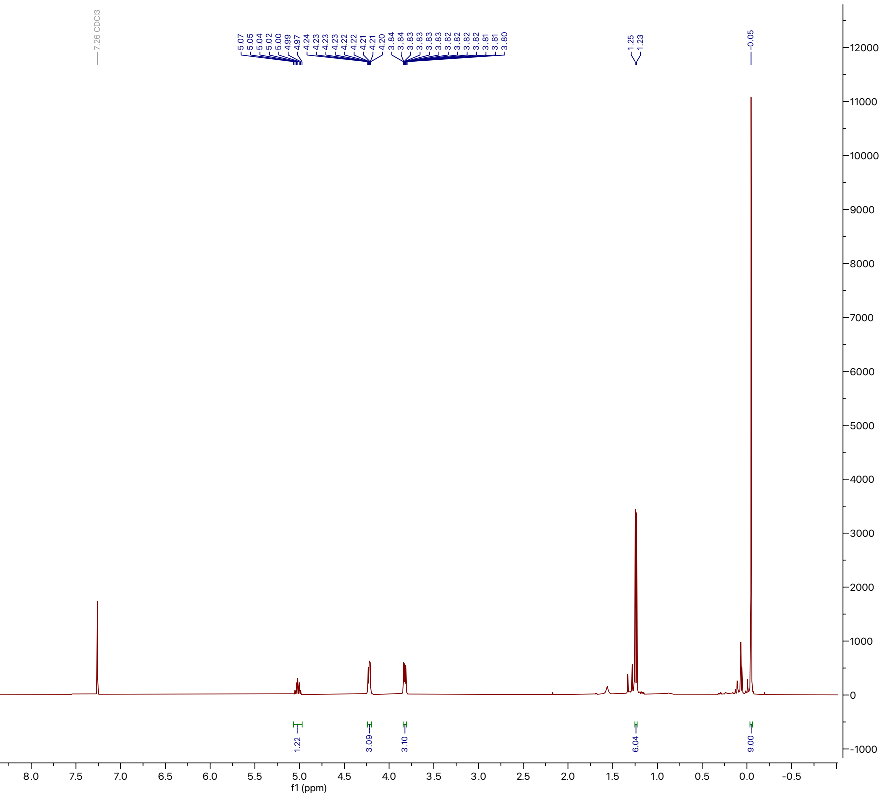

^{13}C NMR (126 MHz, $CDCl_3$) of 3g

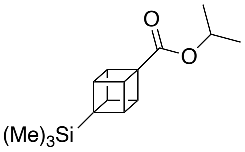

3g

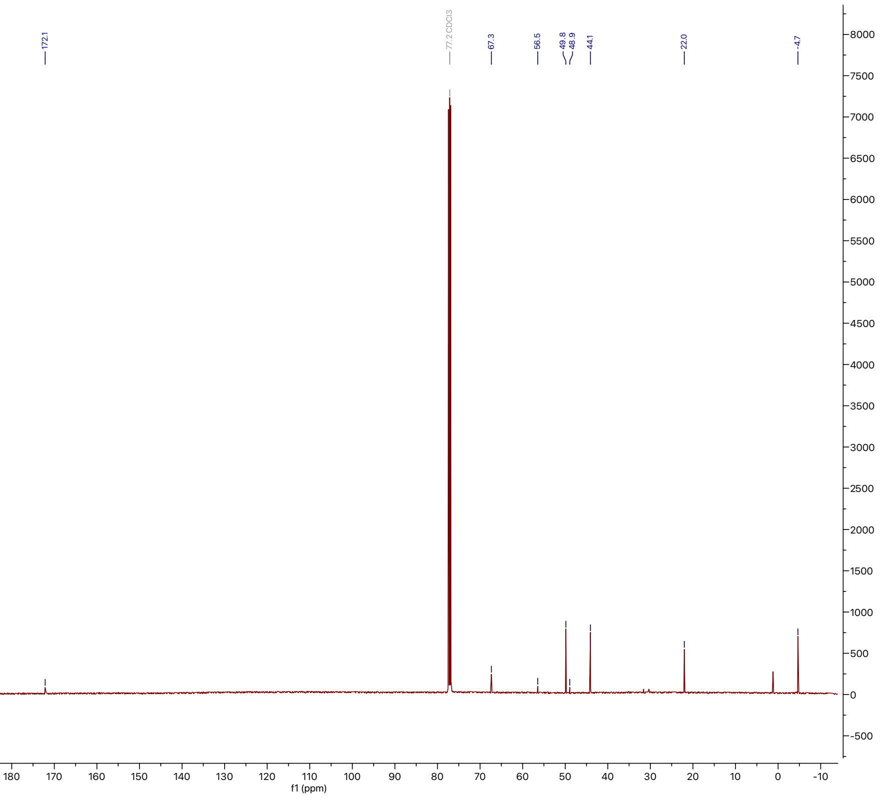

^{1}H NMR (400 MHz, $CDCl_3$) of 2h

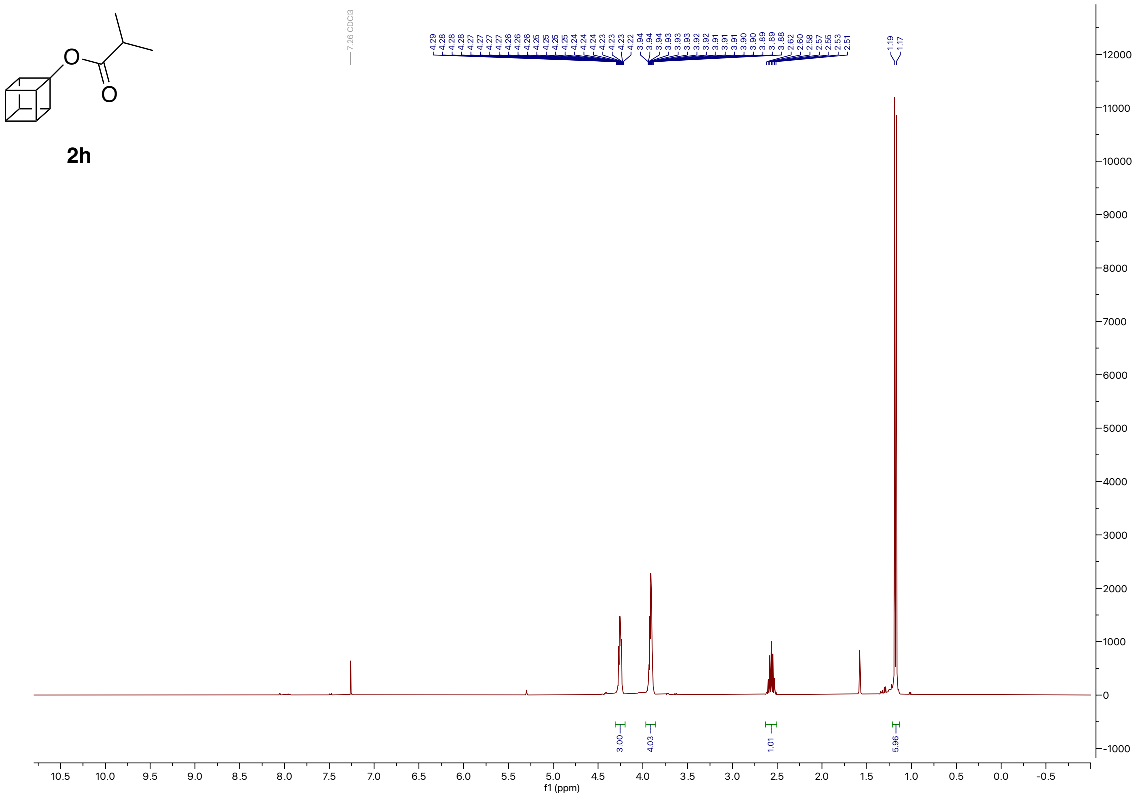

^{13}C NMR (101 MHz, $CDCl_3$) of 2h

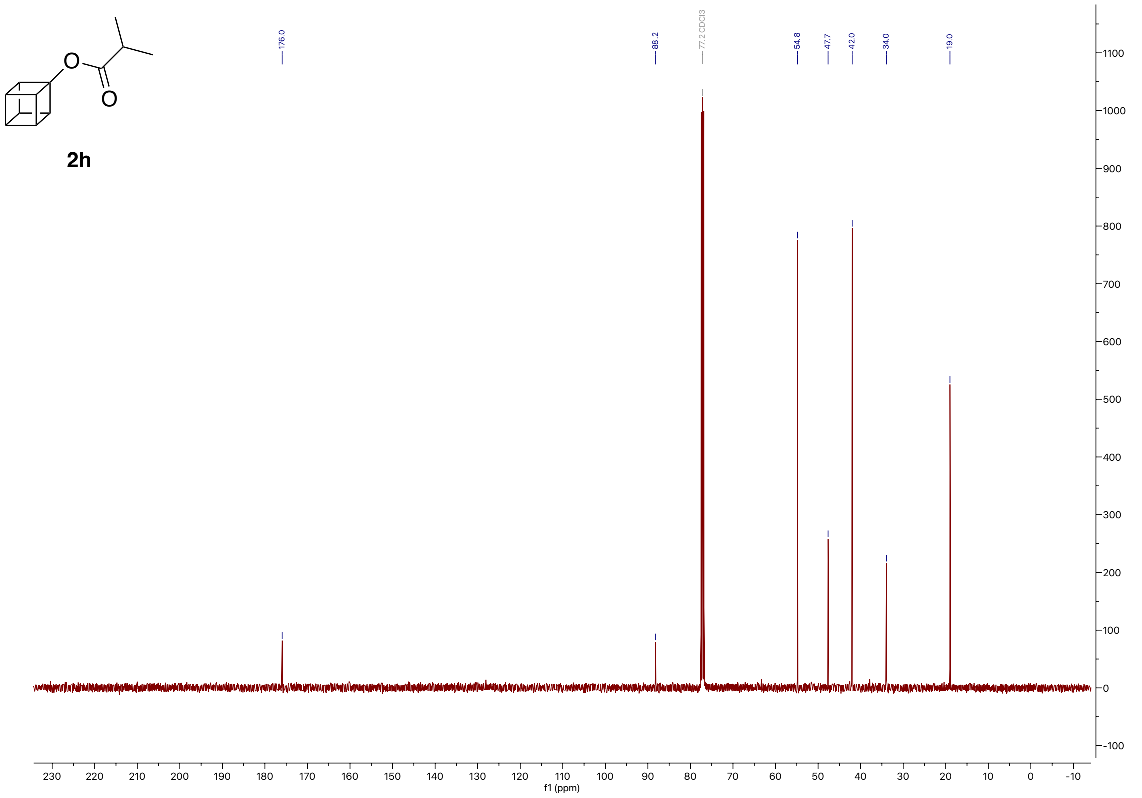

^{1}H NMR (500 MHz, $CDCl_3$) of 3h

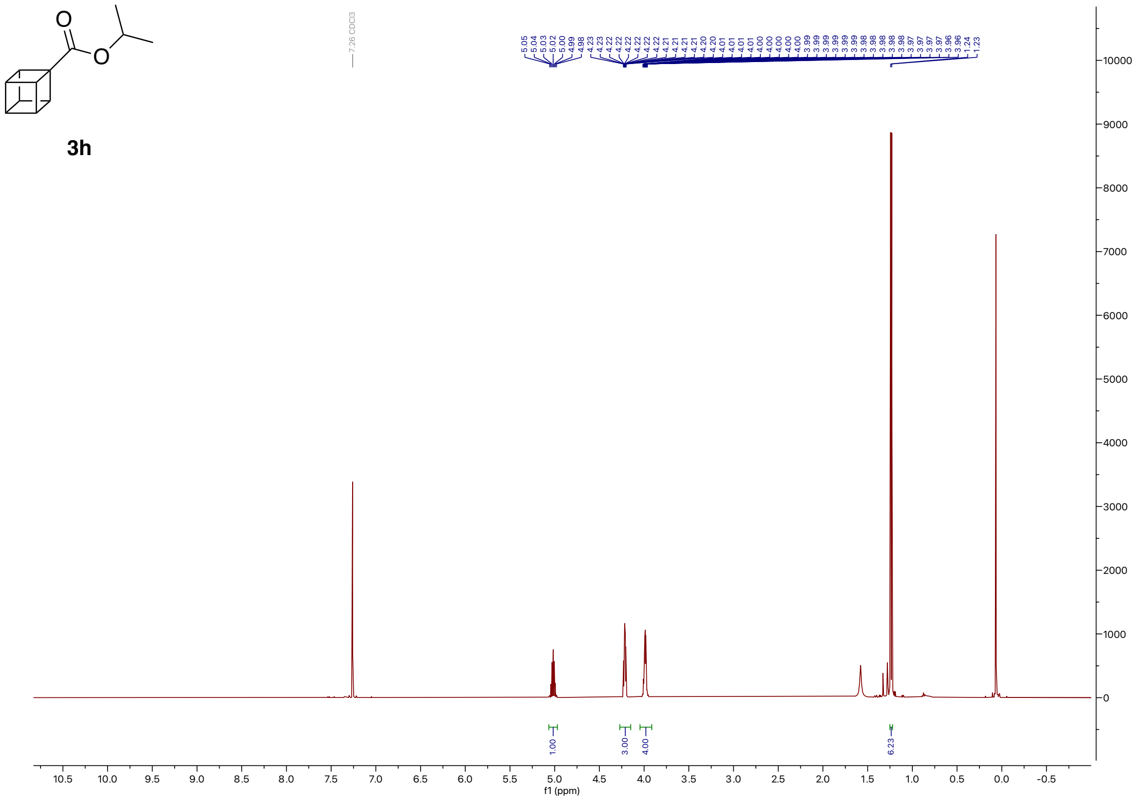

^{13}C NMR (126 MHz, $CDCl_3$) of 3h

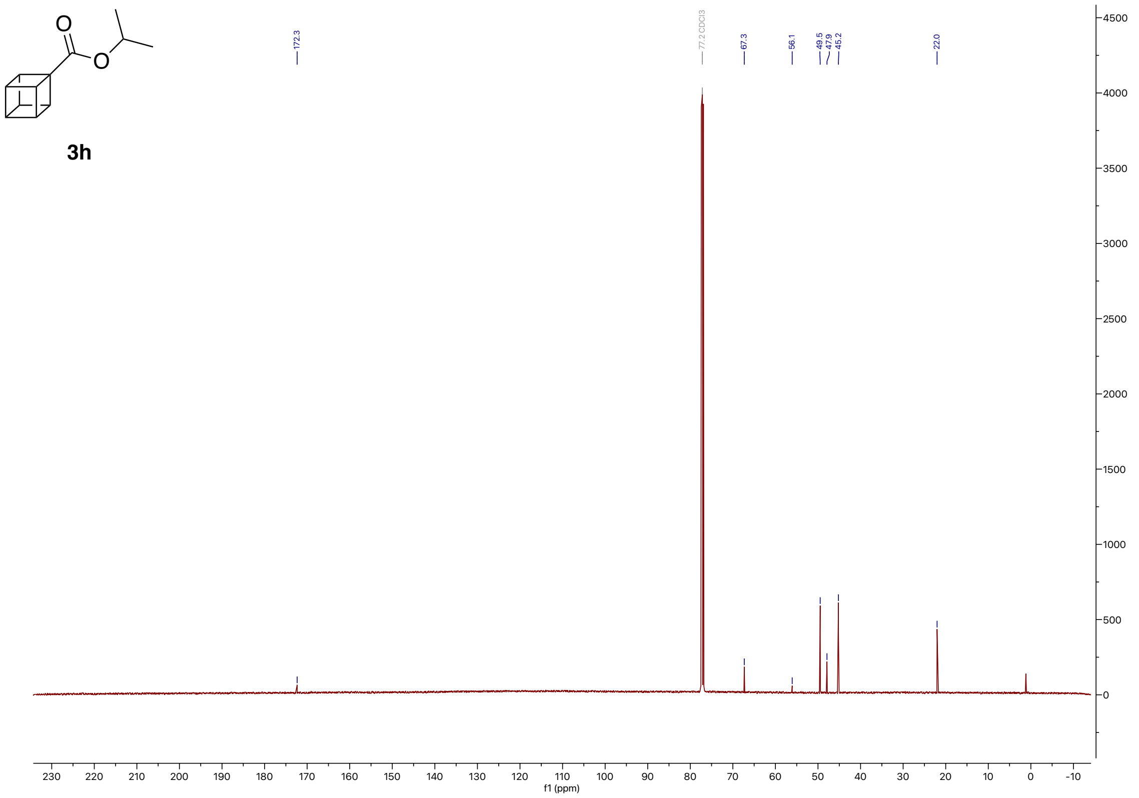

^{1}H NMR (500 MHz, $CDCl_3$) of 2i

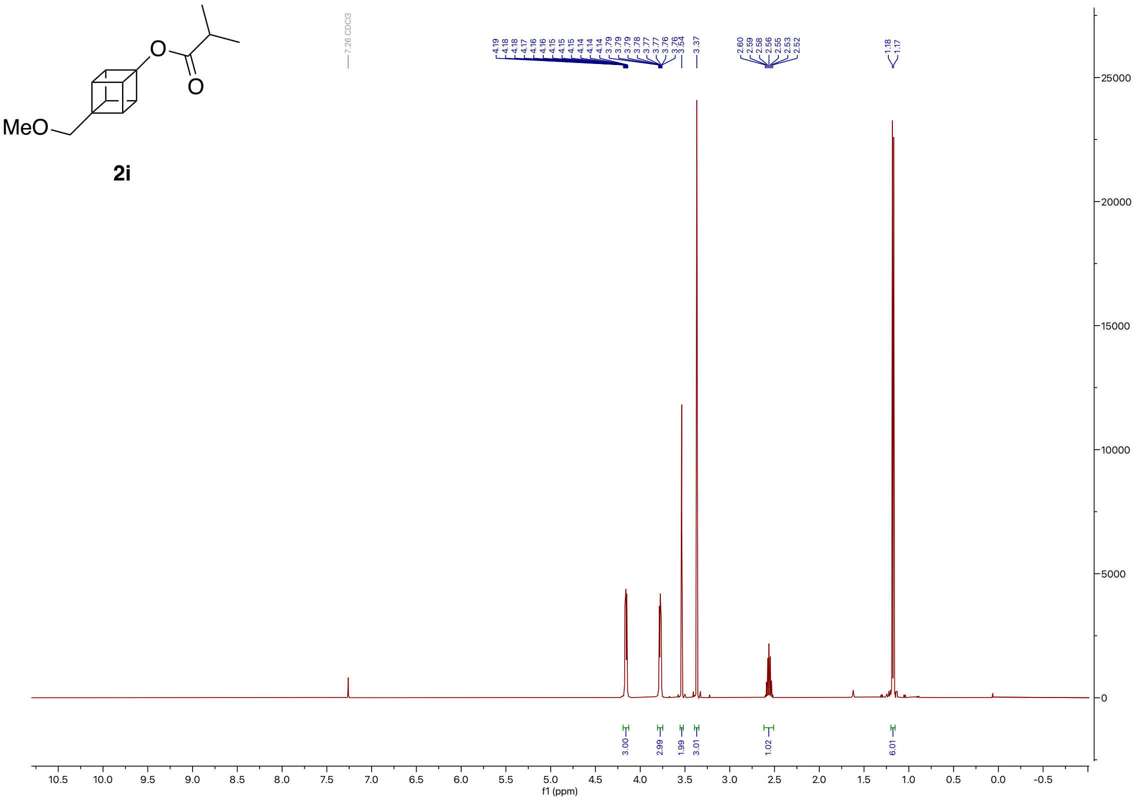

^{13}C NMR (126 MHz, $CDCl_3$) of 2i

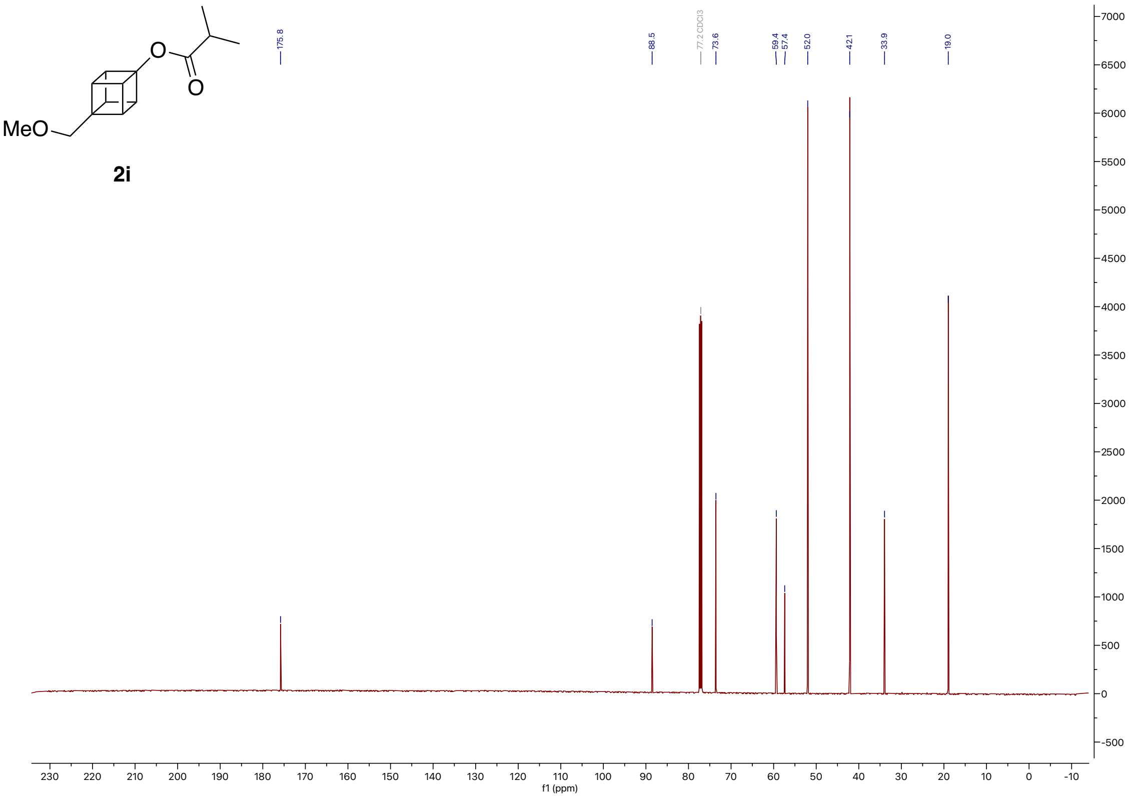

^{1}H NMR (500 MHz, $CDCl_3$) of 3i

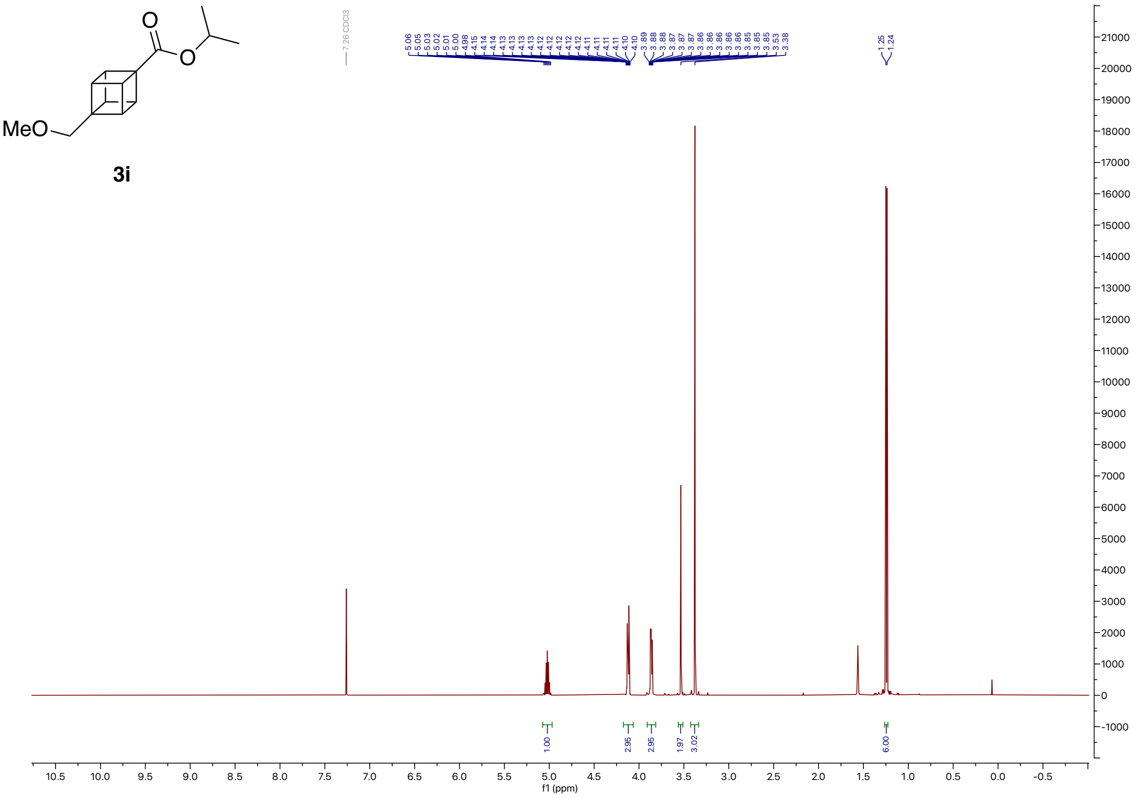

^{13}C NMR (126 MHz, $CDCl_3$) of 3i

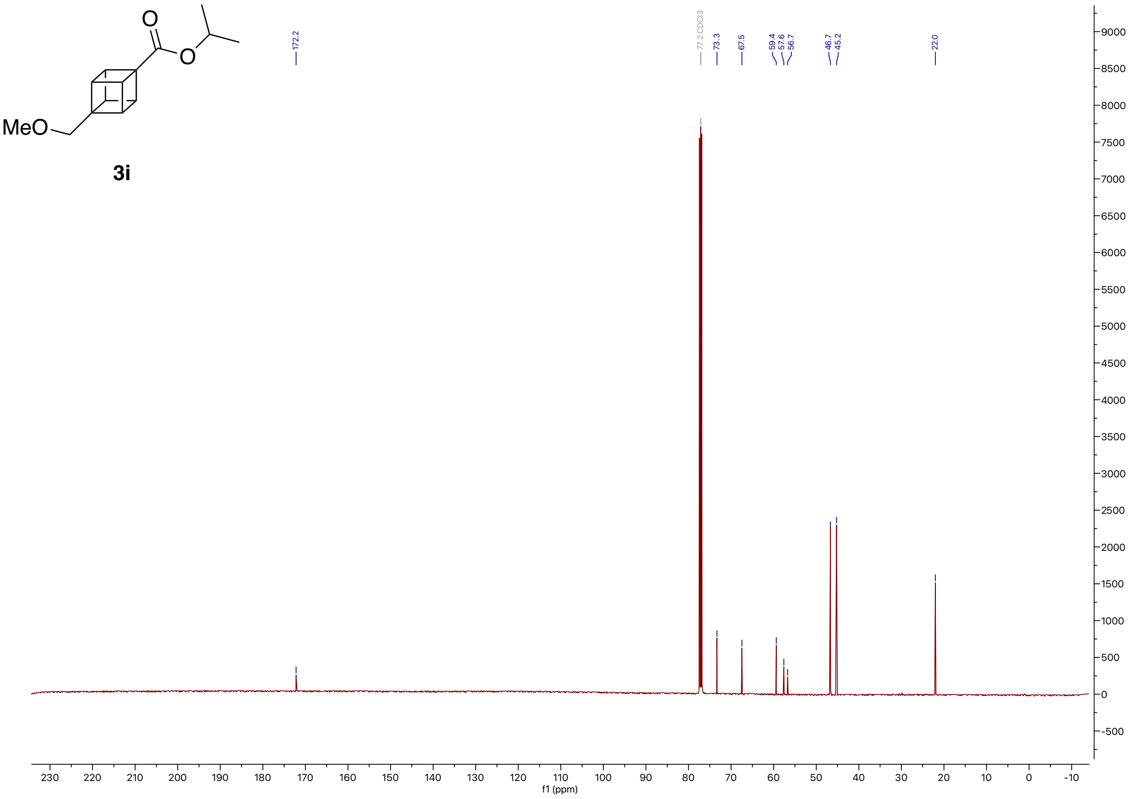

^{1}H NMR (500 MHz, $CDCl_3$) of 2j

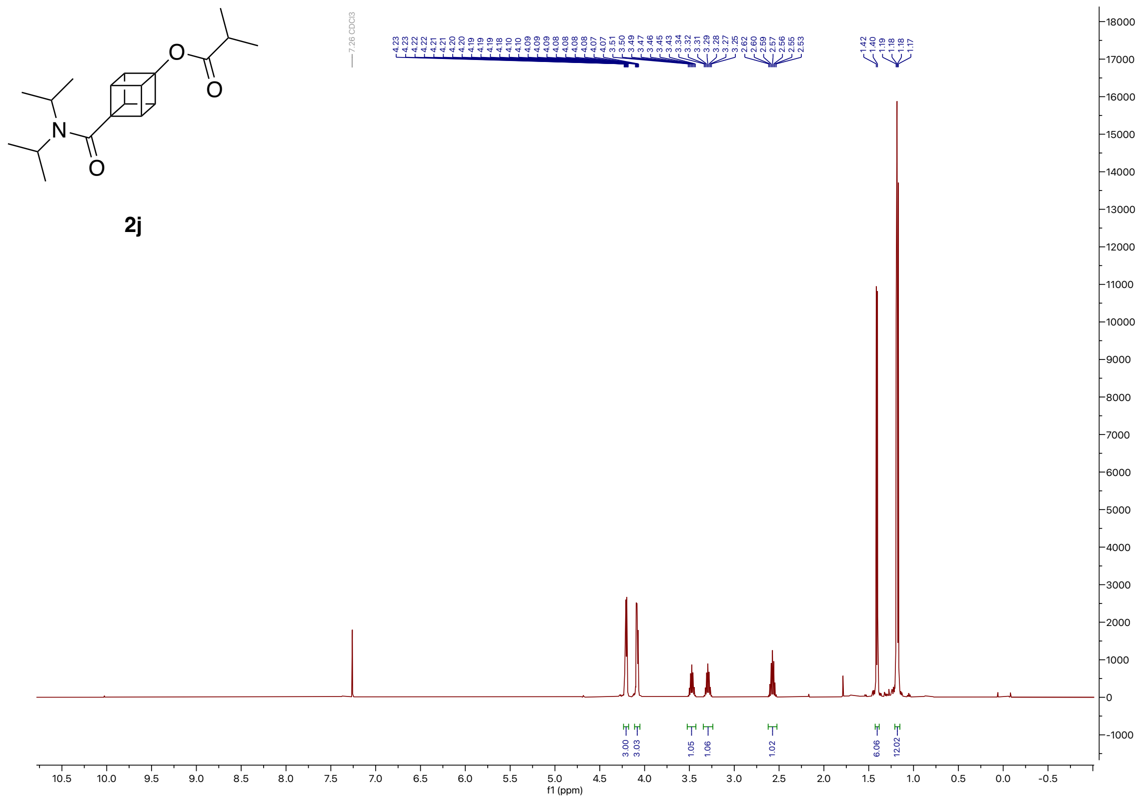

2j

^{13}C NMR (126 MHz, $CDCl_3$) of 2j

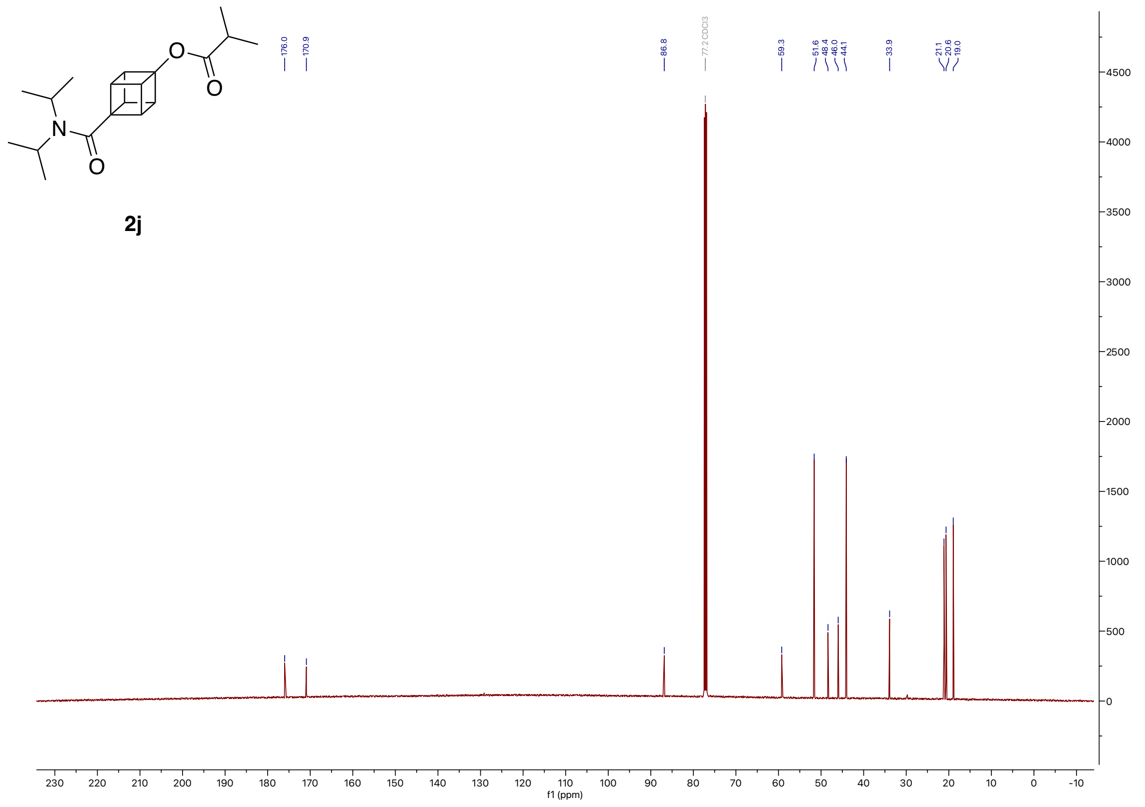

2j

^{1}H NMR (500 MHz, $CDCl_3$) of 3j

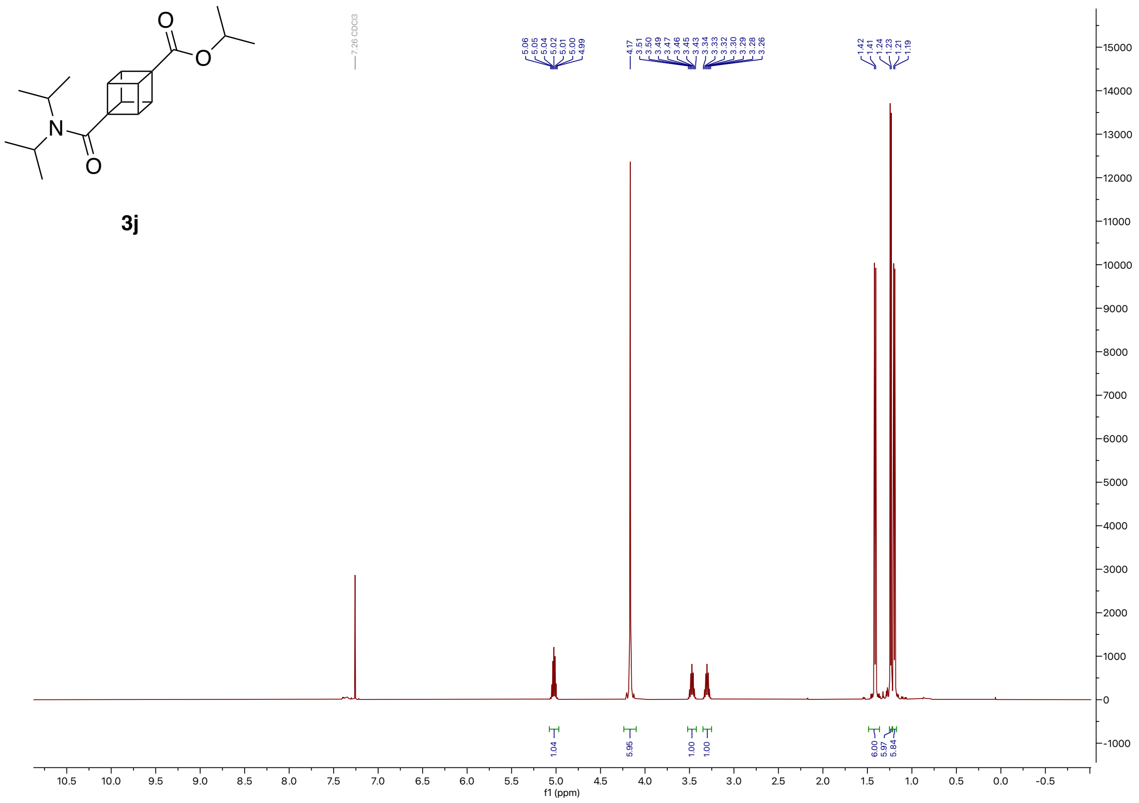

^{13}C NMR (126 MHz, $CDCl_3$) of 3j

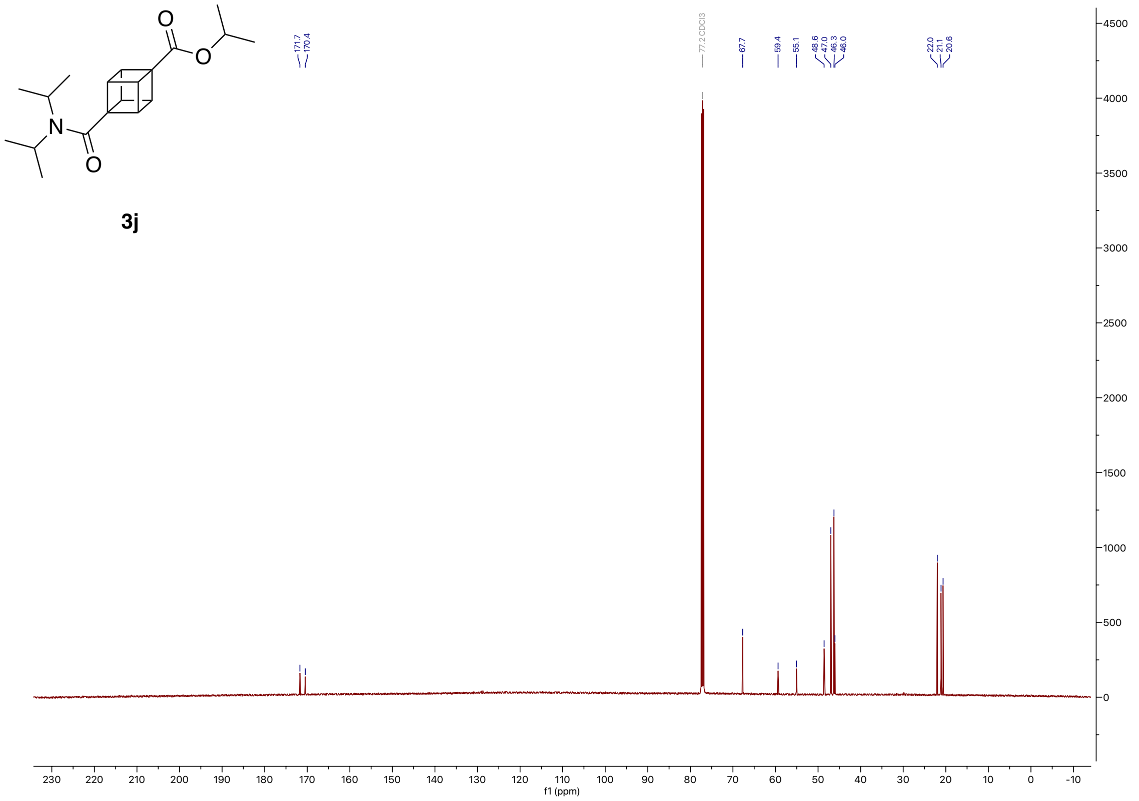

^{1}H NMR (500 MHz, $CDCl_3$) of 2k

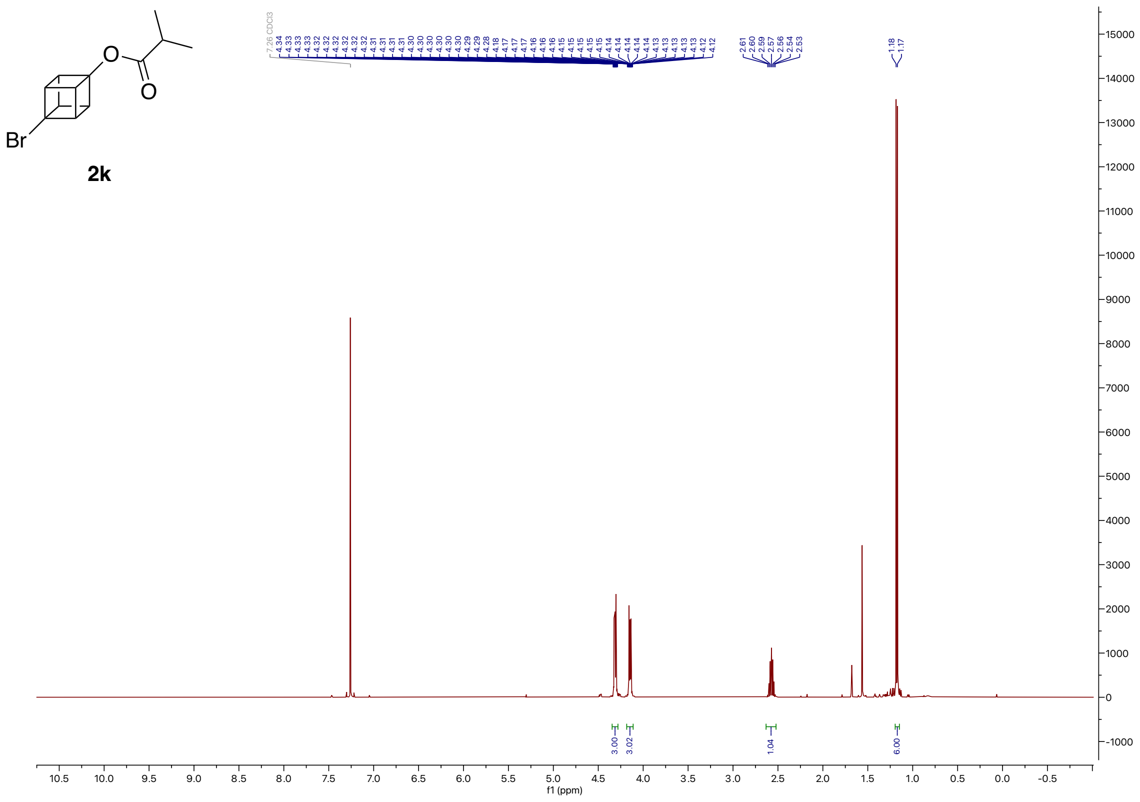

^{13}C NMR (126 MHz, $CDCl_3$) of 2k

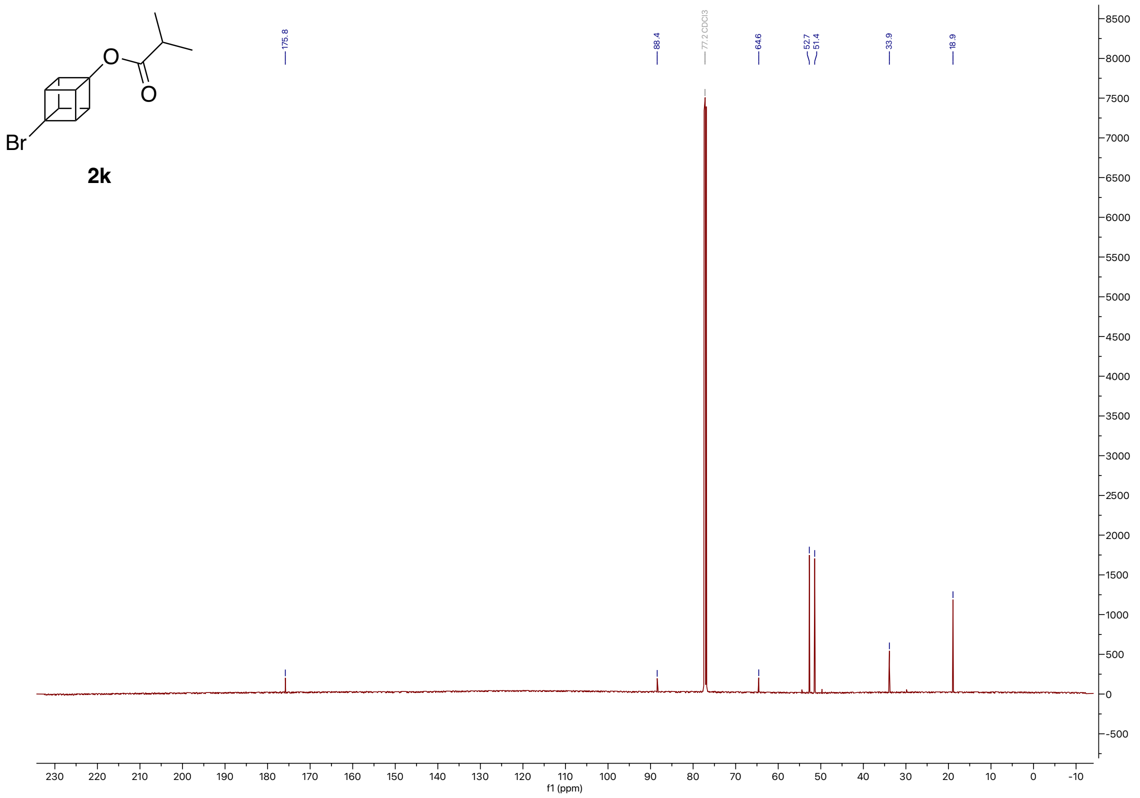

^{1}H NMR (400 MHz, $CDCl_3$) of 3k

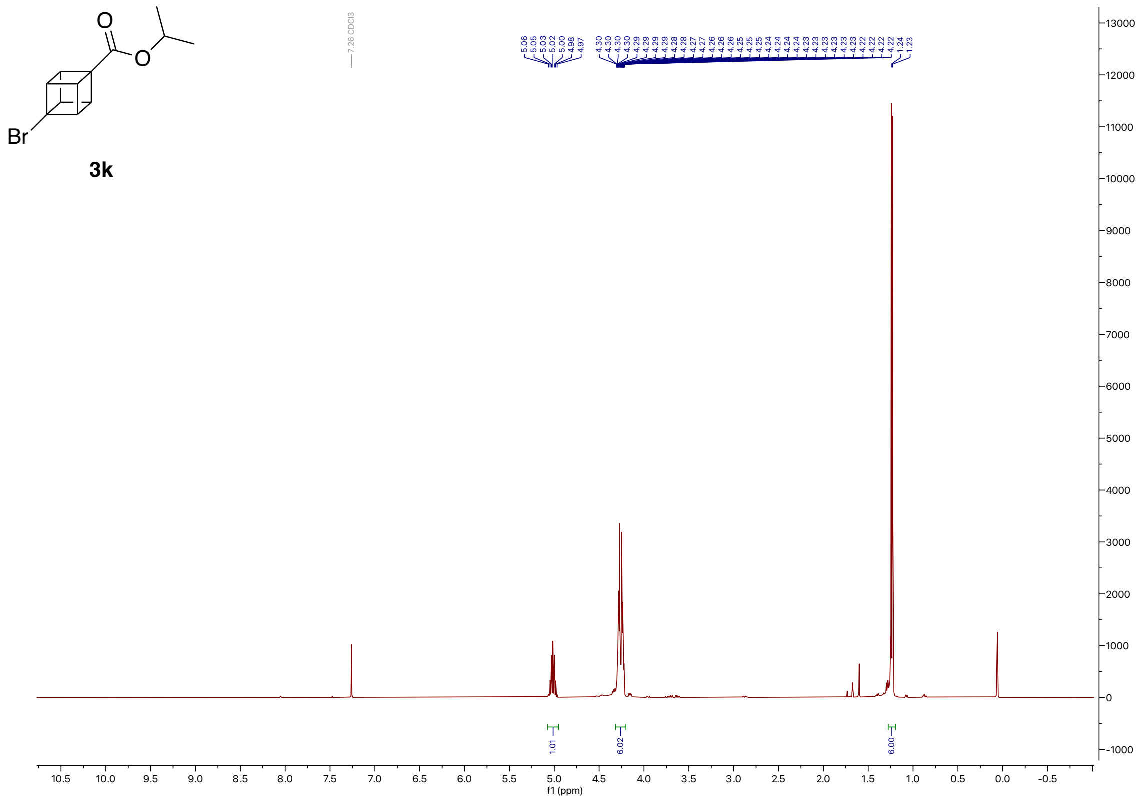

^{13}C NMR (101 MHz, $CDCl_3$) of 3k

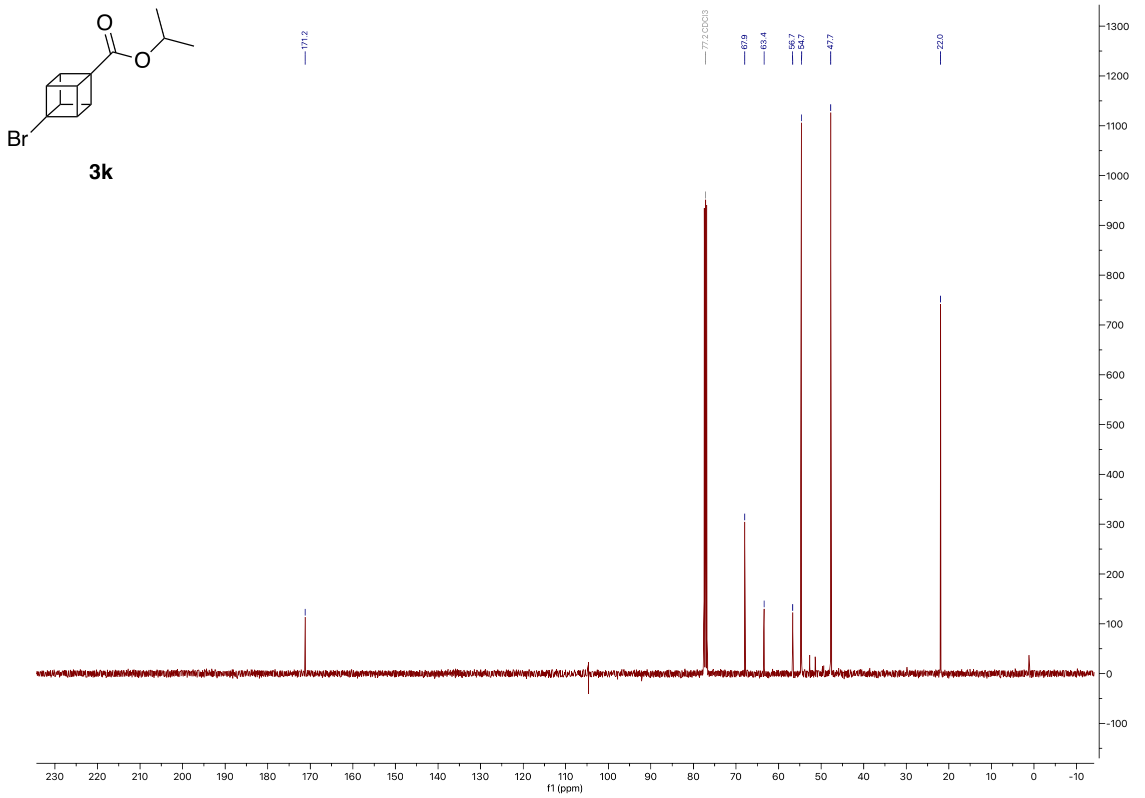

^1H NMR (500 MHz, $CDCl_3$) of 2l

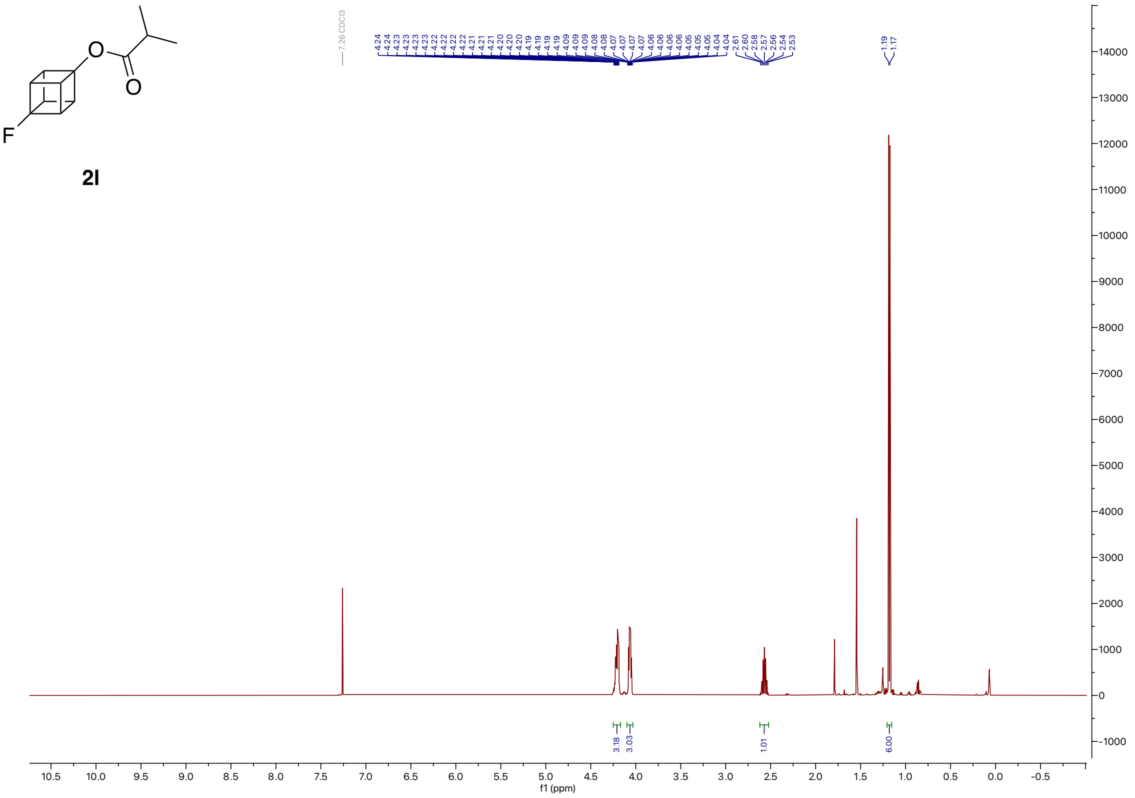

^{13}C NMR (126 MHz, $CDCl_3$) of 2l

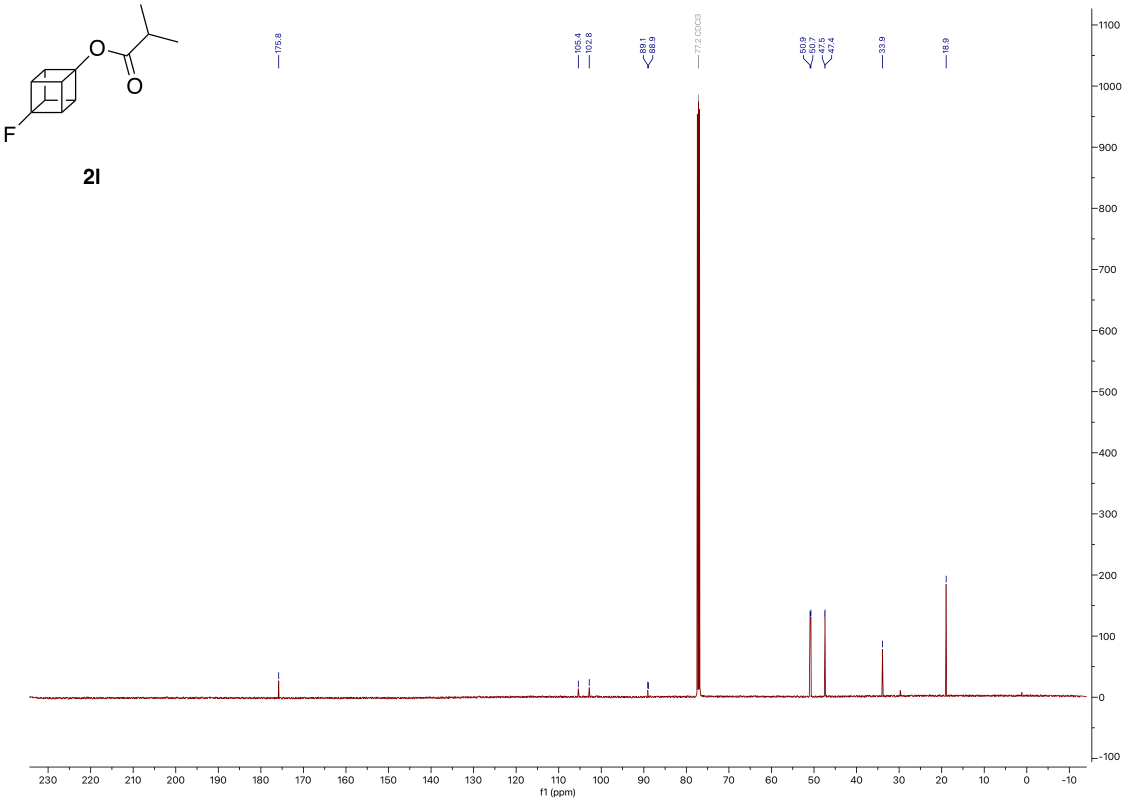

$^{19}F\{1H\}$NMR (471 MHz, $CDCl_3$) of 2l

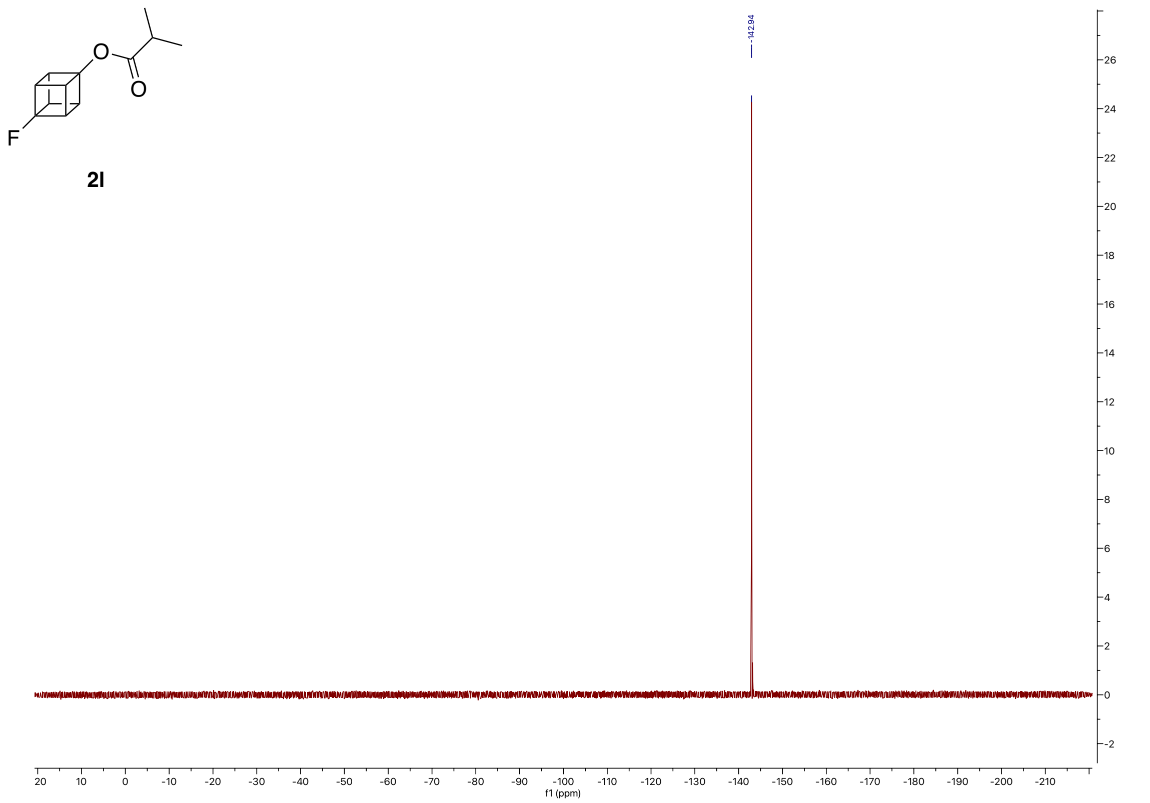

^{1}H NMR (400 MHz, $CDCl_3$) of 3l

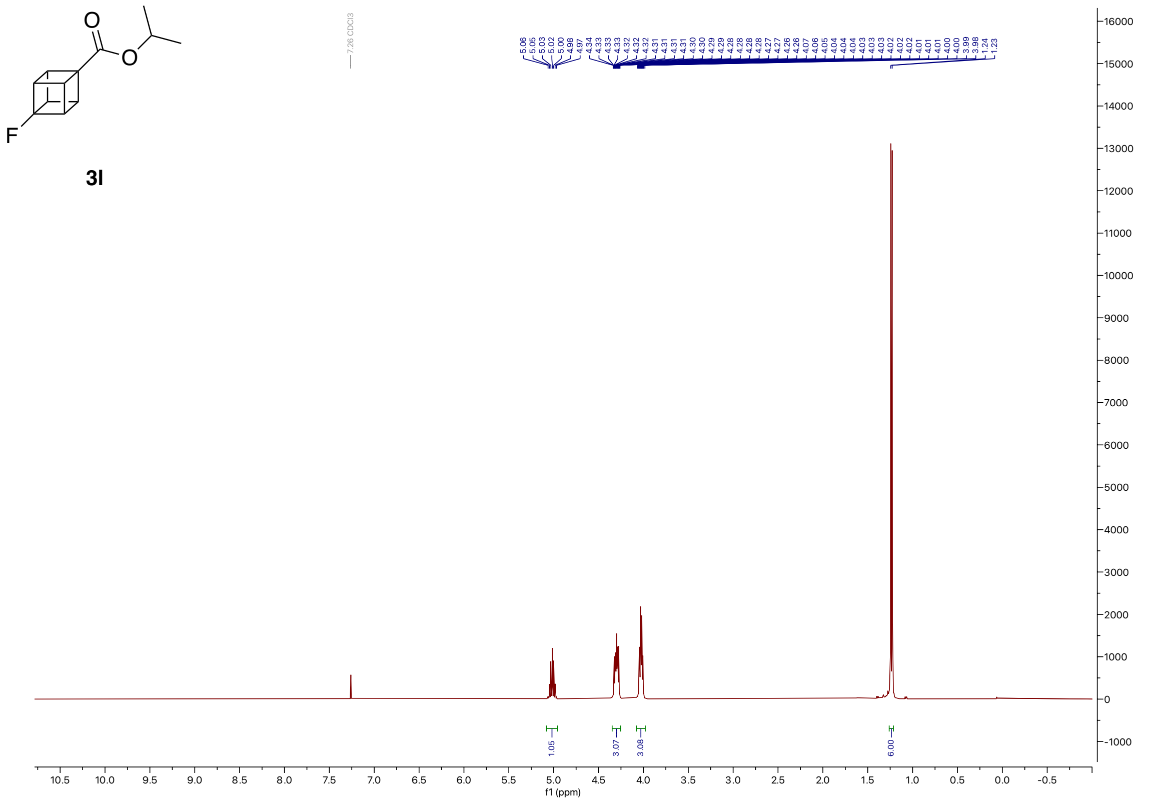

^{13}C NMR (101 MHz, $CDCl_3$) of 3l

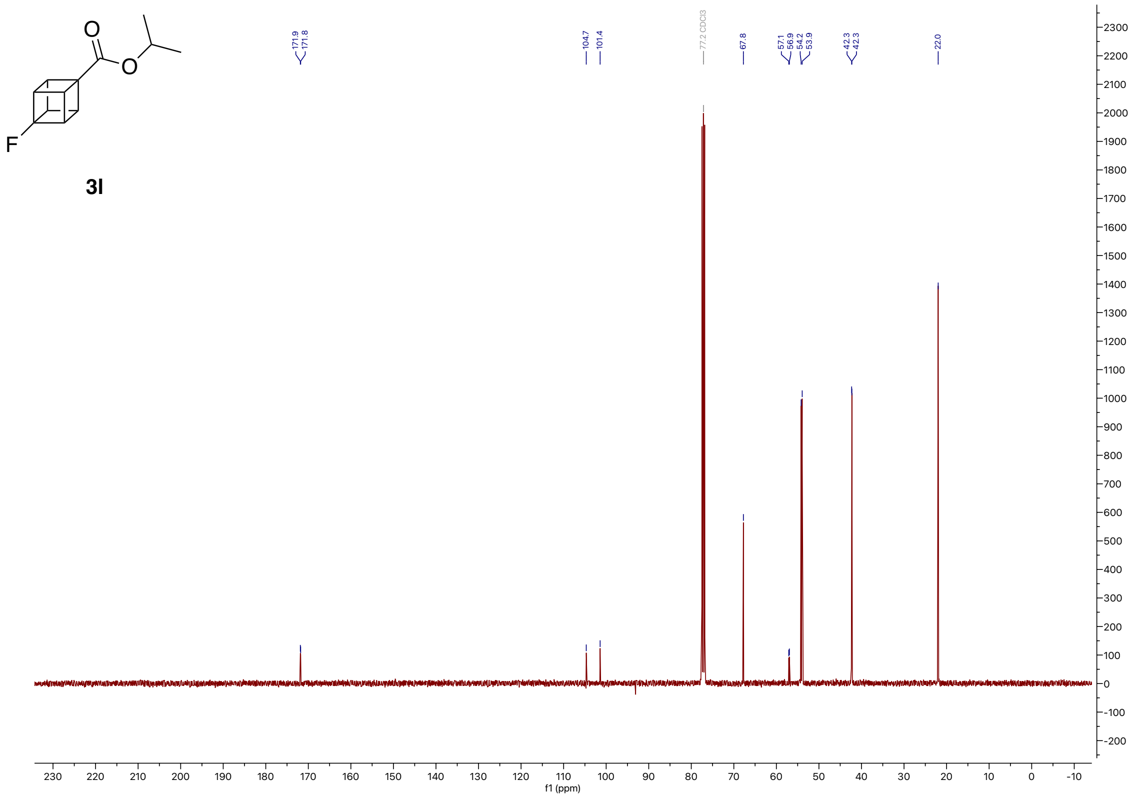

$^{19}F\{1H\}$NMR (376 MHz, $CDCl_3$) of 3l

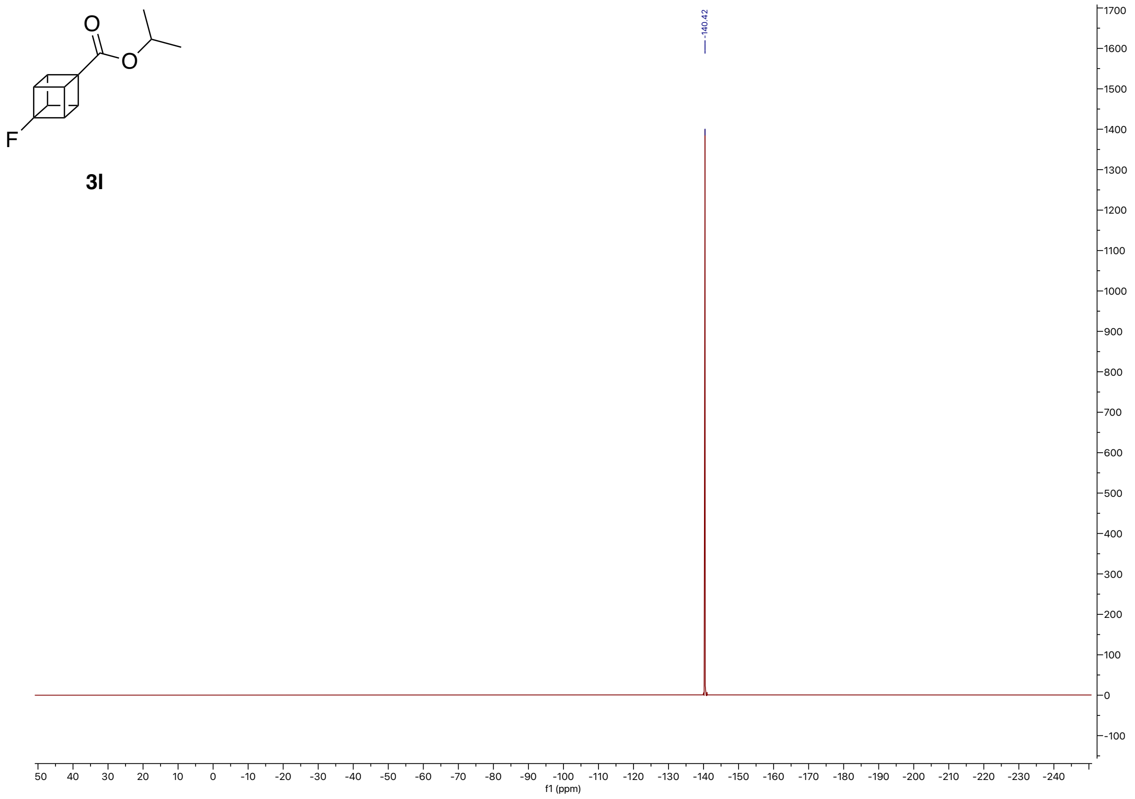

^{1}H NMR (300 MHz, $CDCl_3$) of 4b

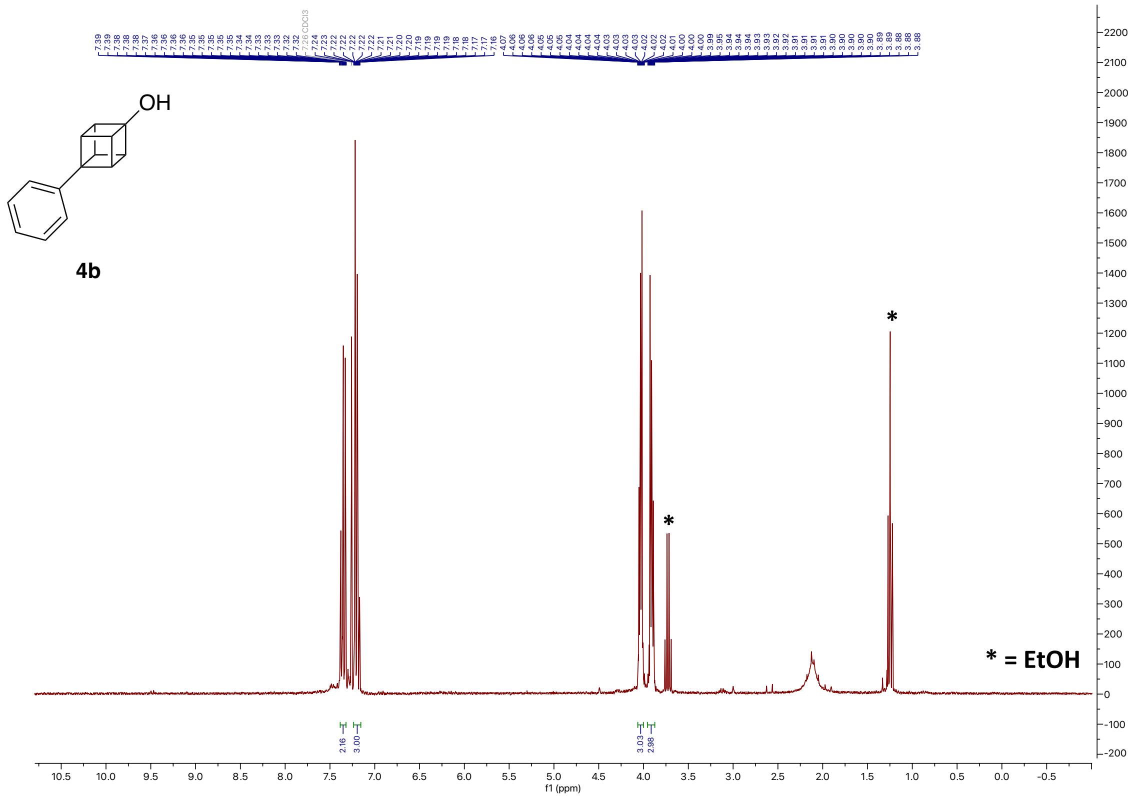

^{1}H NMR (300 MHz, $CDCl_3$) overlay of 2b and 4b

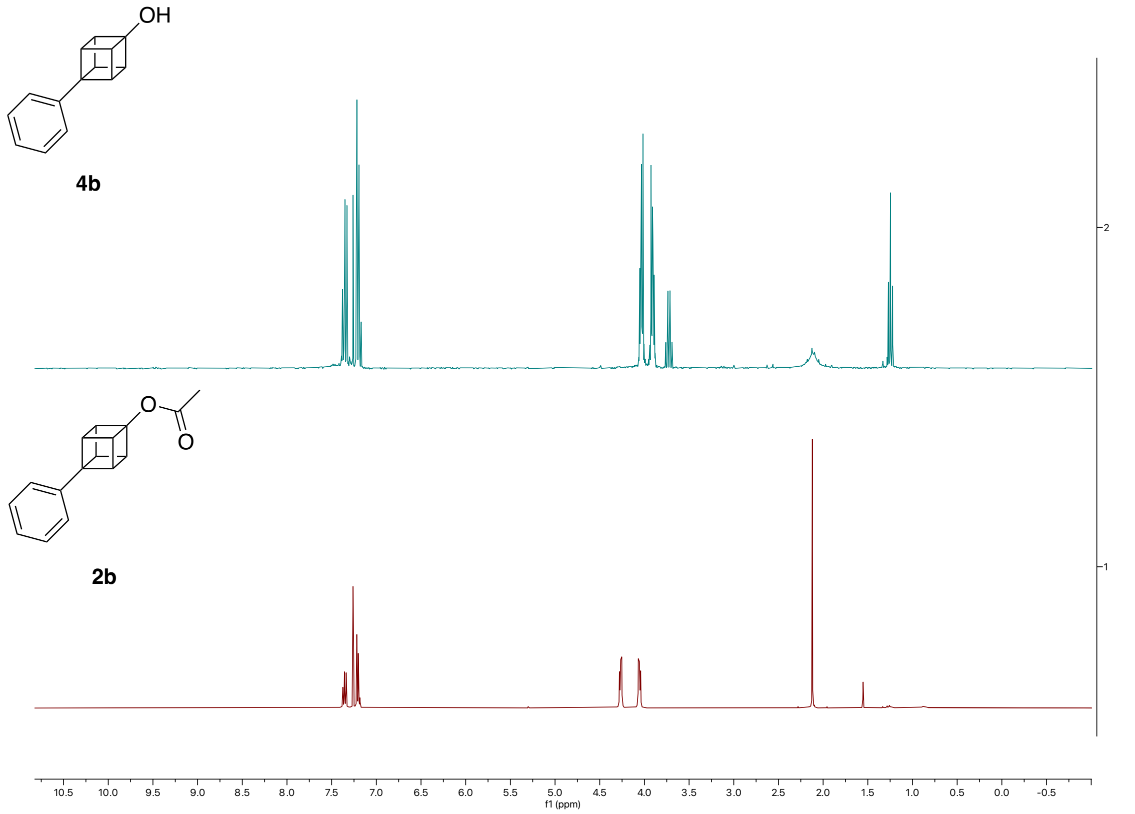

^{1}H NMR (300 MHz, $CDCl_3$) decomposition of 4b within 1 h in $CDCl_3$

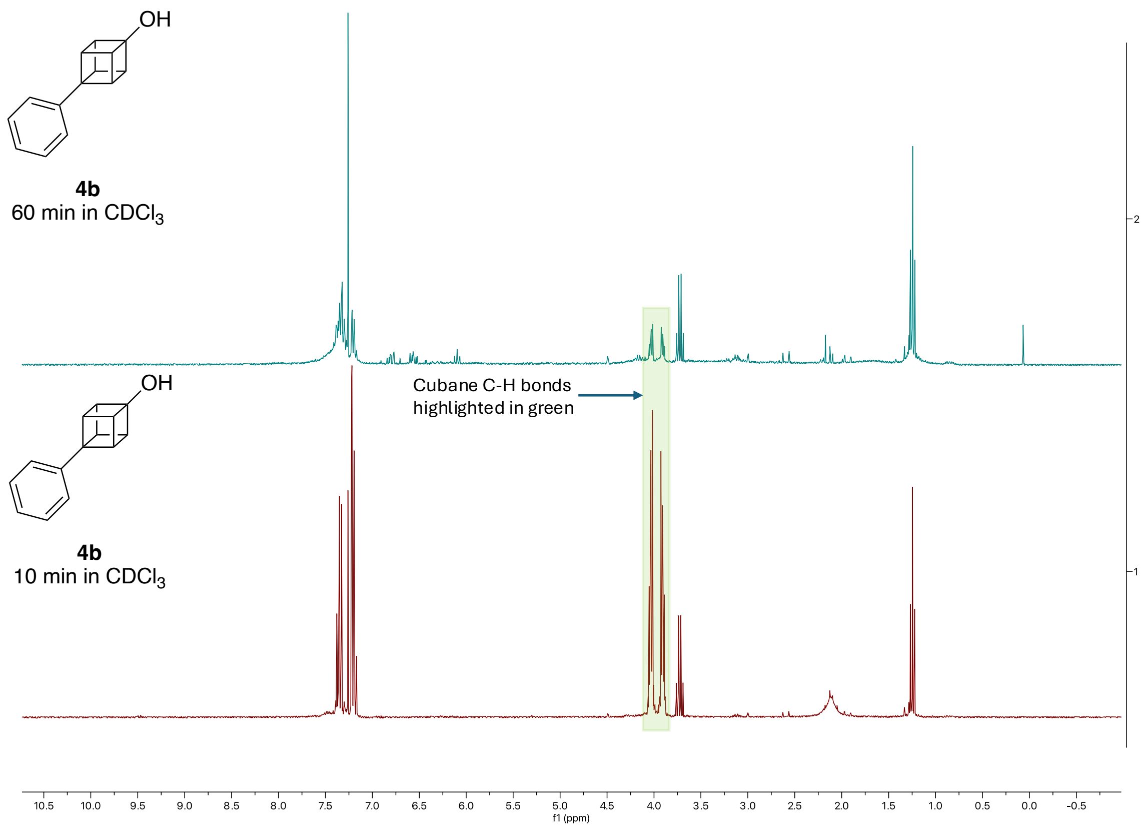

^{1}H NMR (400 MHz, Acetic Acid-d_3) 4b in Acetic Acid-d_3 for 24 h

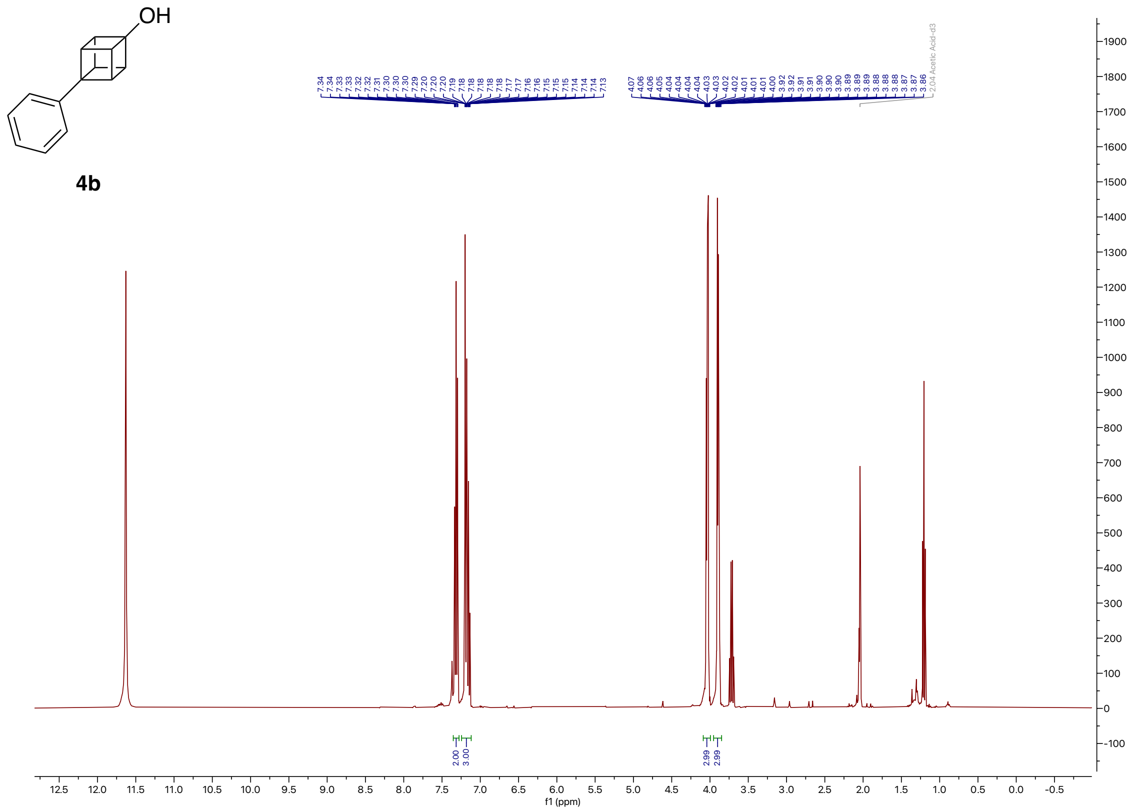

^{1}H NMR (400 MHz, $CDCl_3$) of S30

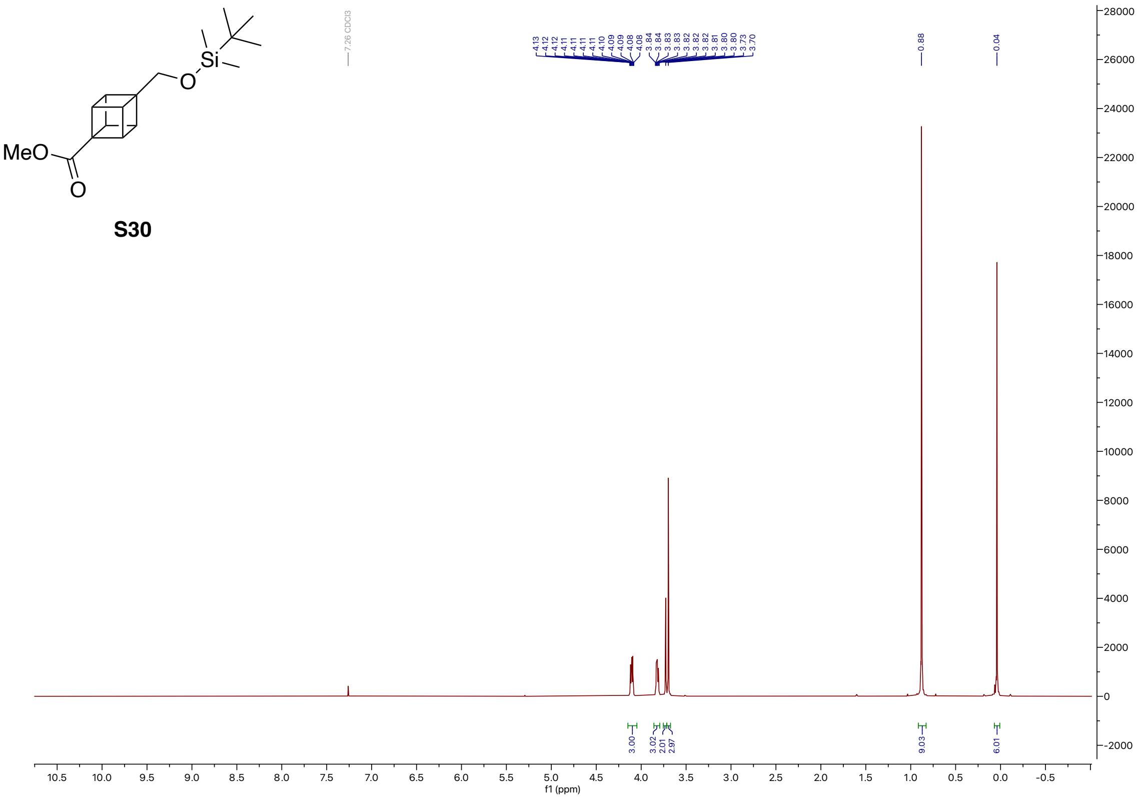

^{13}C NMR (101 MHz, $CDCl_3$) of S30

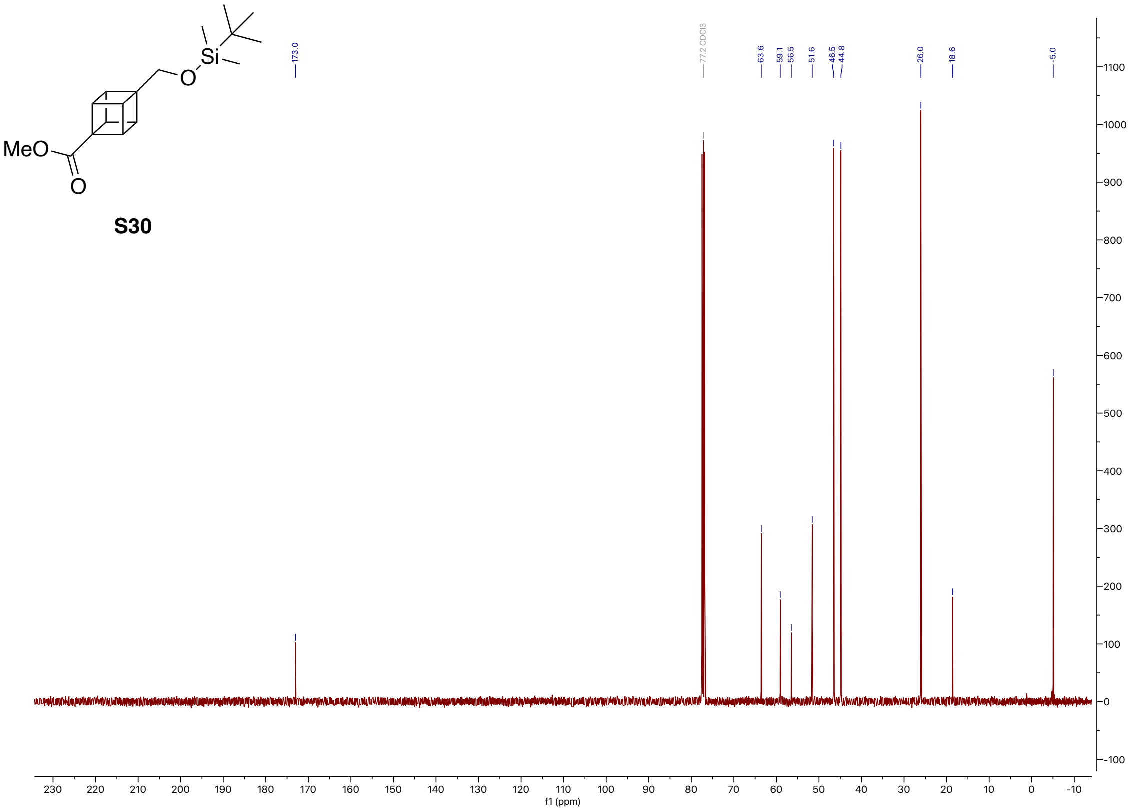

^{1}H NMR (400 MHz, $CDCl_3$) of S31

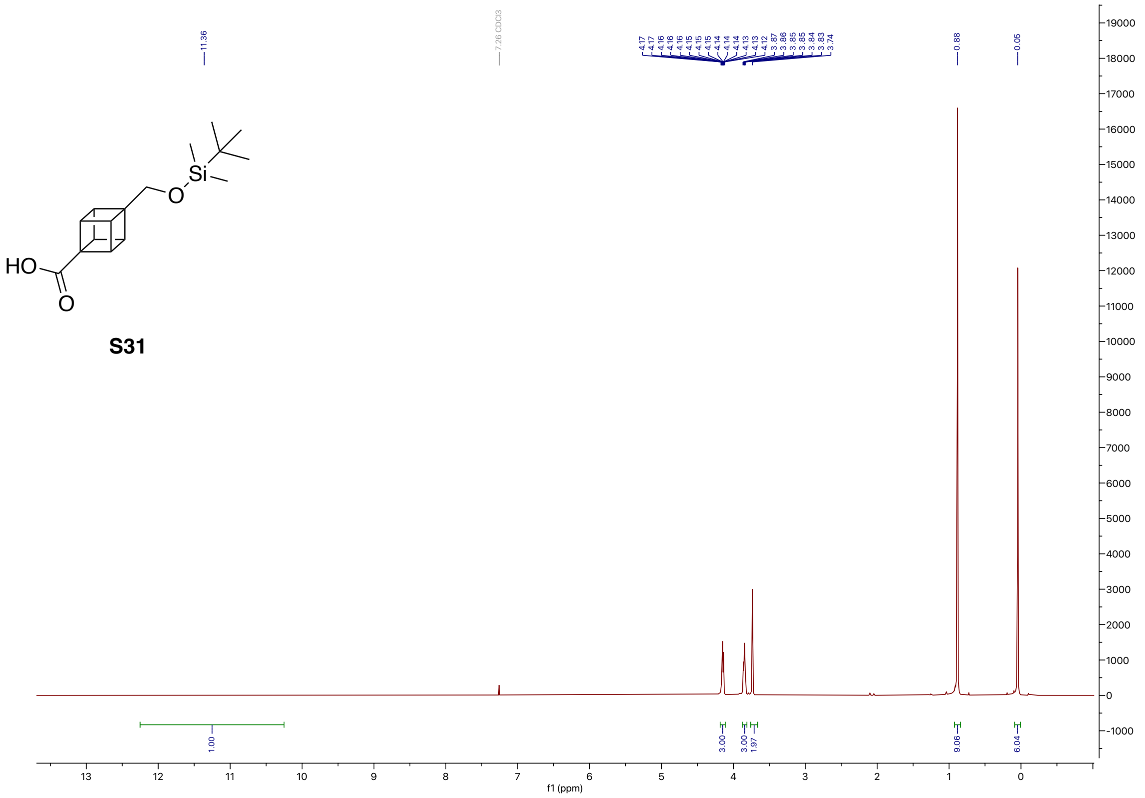

S31

^{13}C NMR (101 MHz, $CDCl_3$) of S31

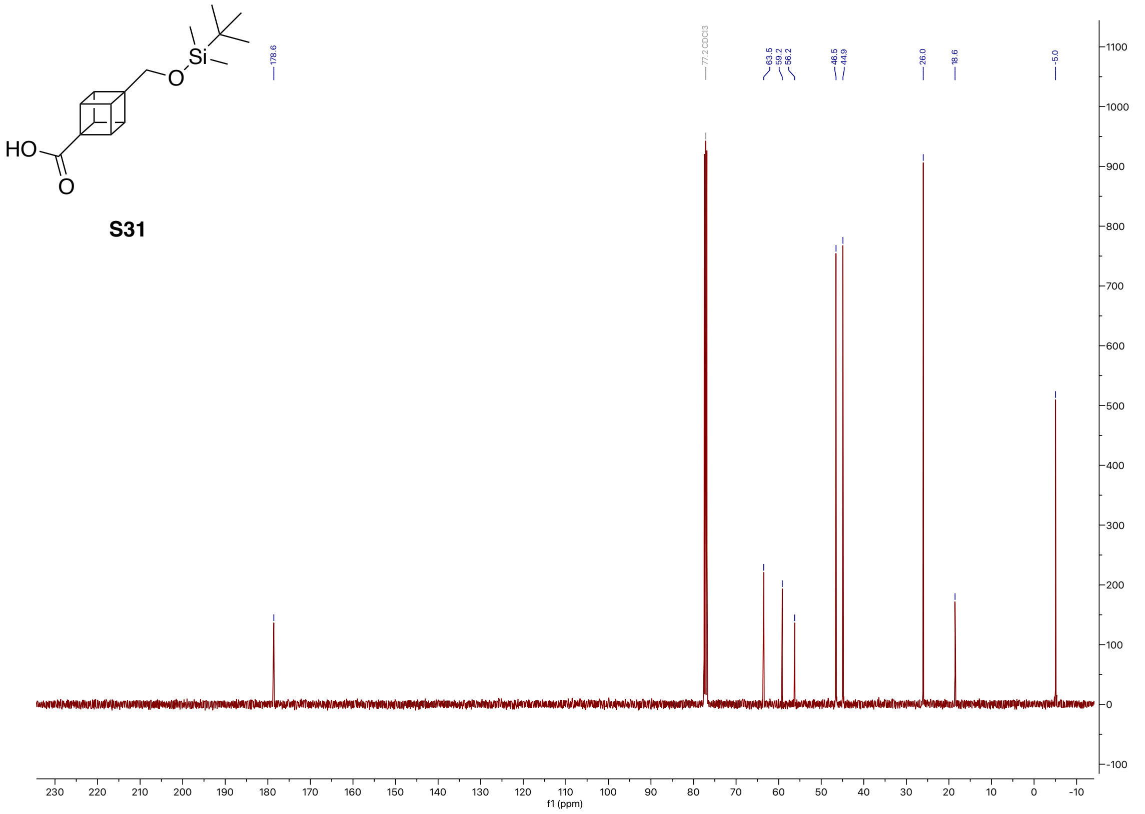

S31

^{1}H NMR (500 MHz, $CDCl_3$) of S32

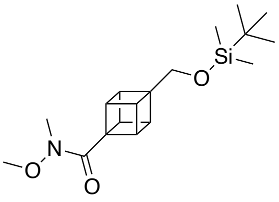

S32

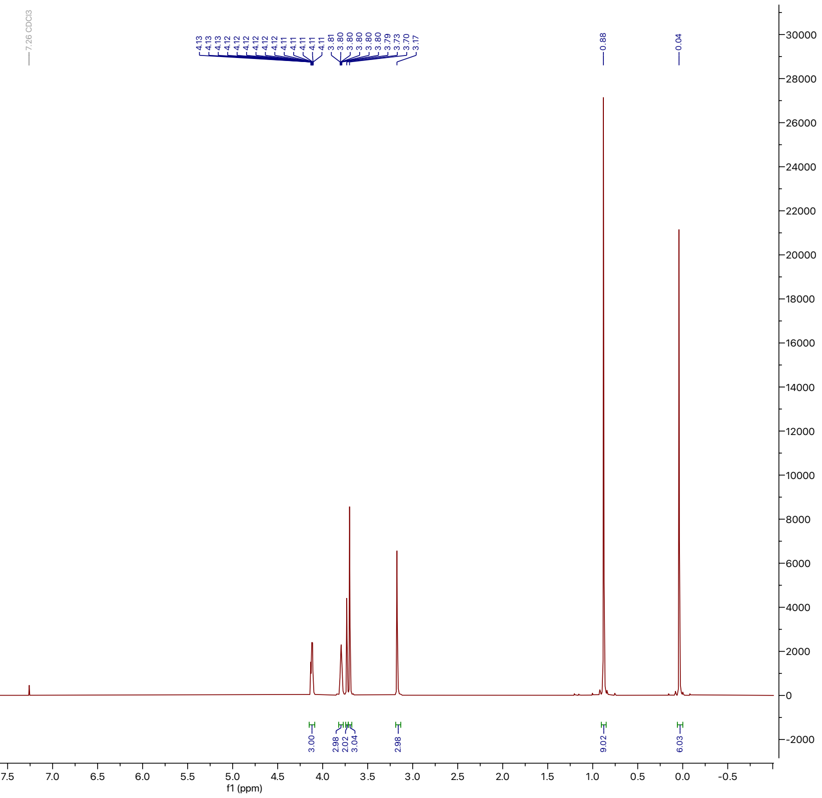

^{13}C NMR (126 MHz, $CDCl_3$) of S32

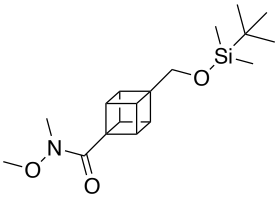

S32

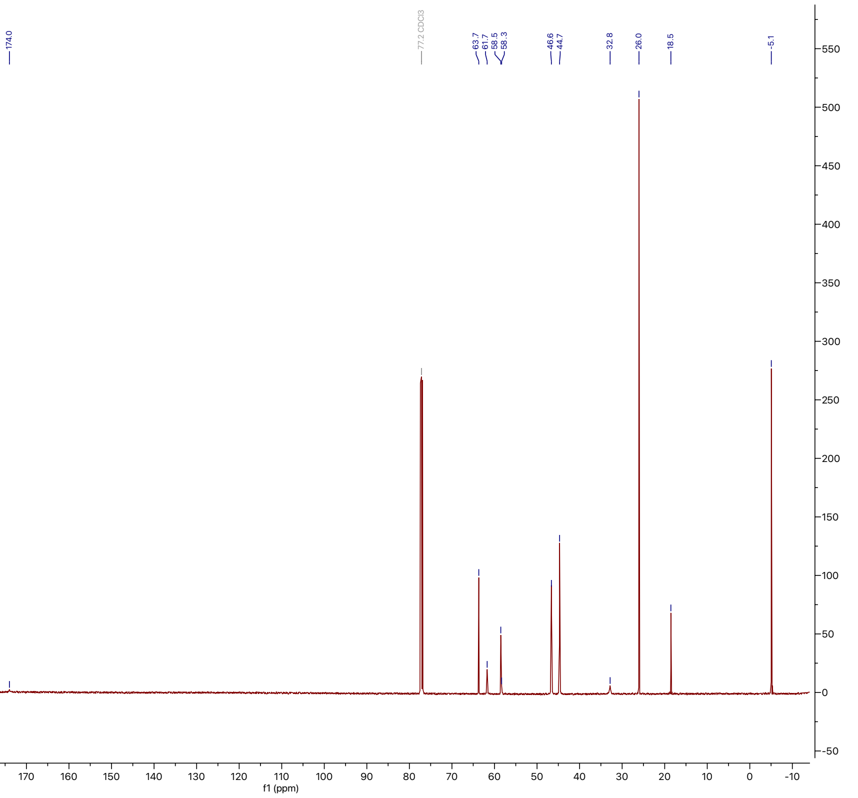

^{1}H NMR (400 MHz, $CDCl_3$) of 5

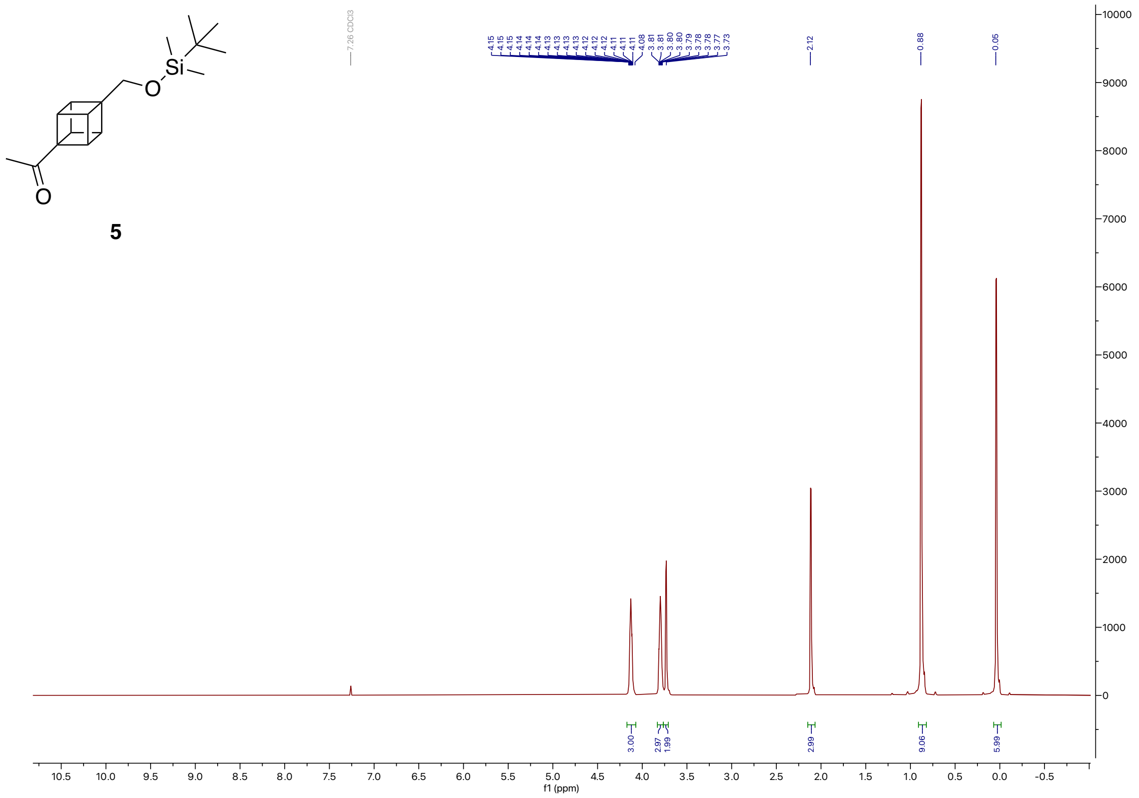

^{13}C NMR (101 MHz, $CDCl_3$) of 5

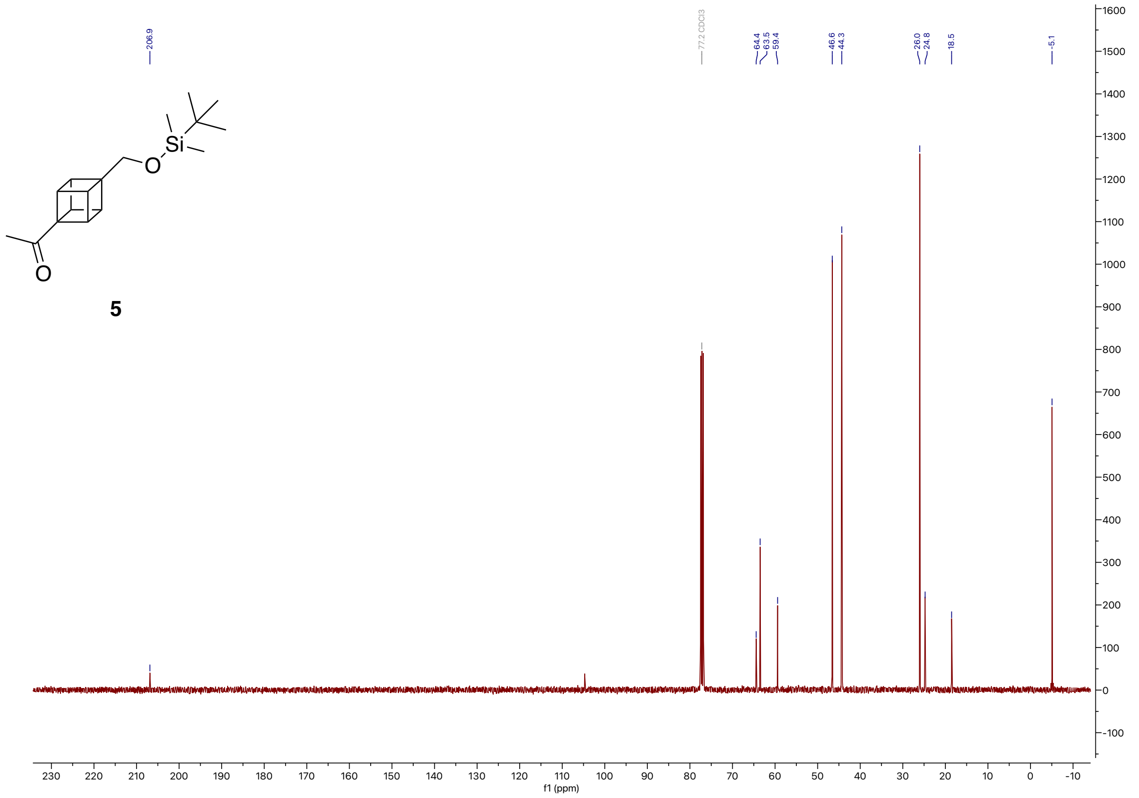

^{1}H NMR (500 MHz, CDCl$_3$) of 6

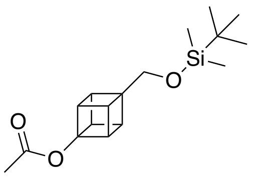

6

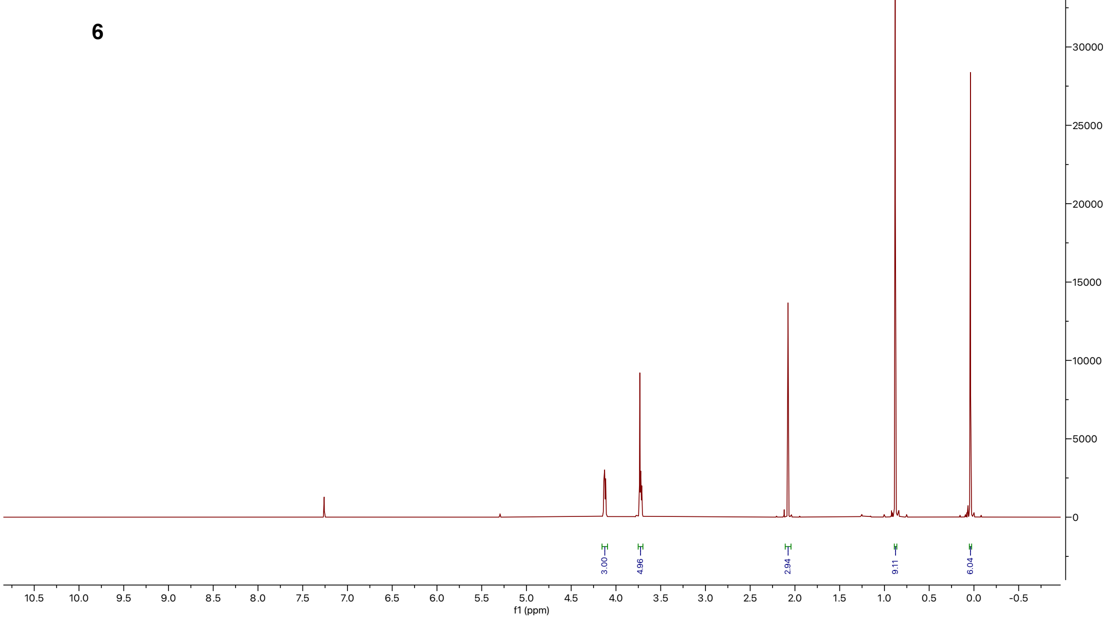

^{13}C NMR (126 MHz, CDCl$_3$) of 6

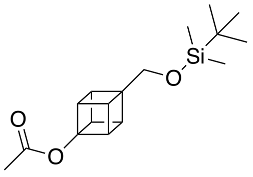

6

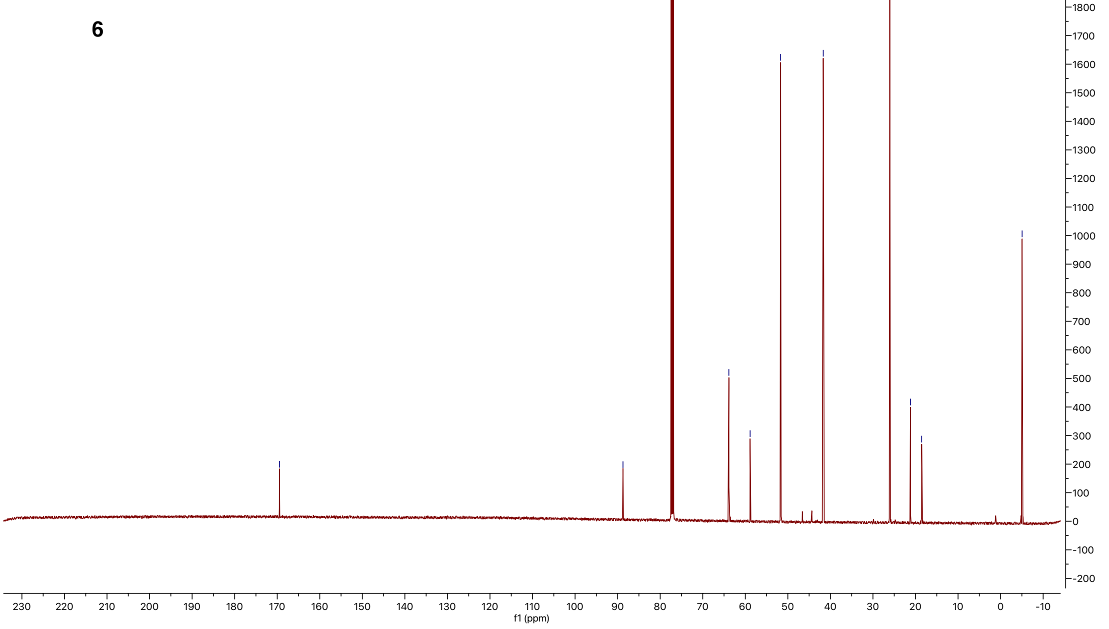

^{1}H NMR (400 MHz, $CDCl_3$) of S33

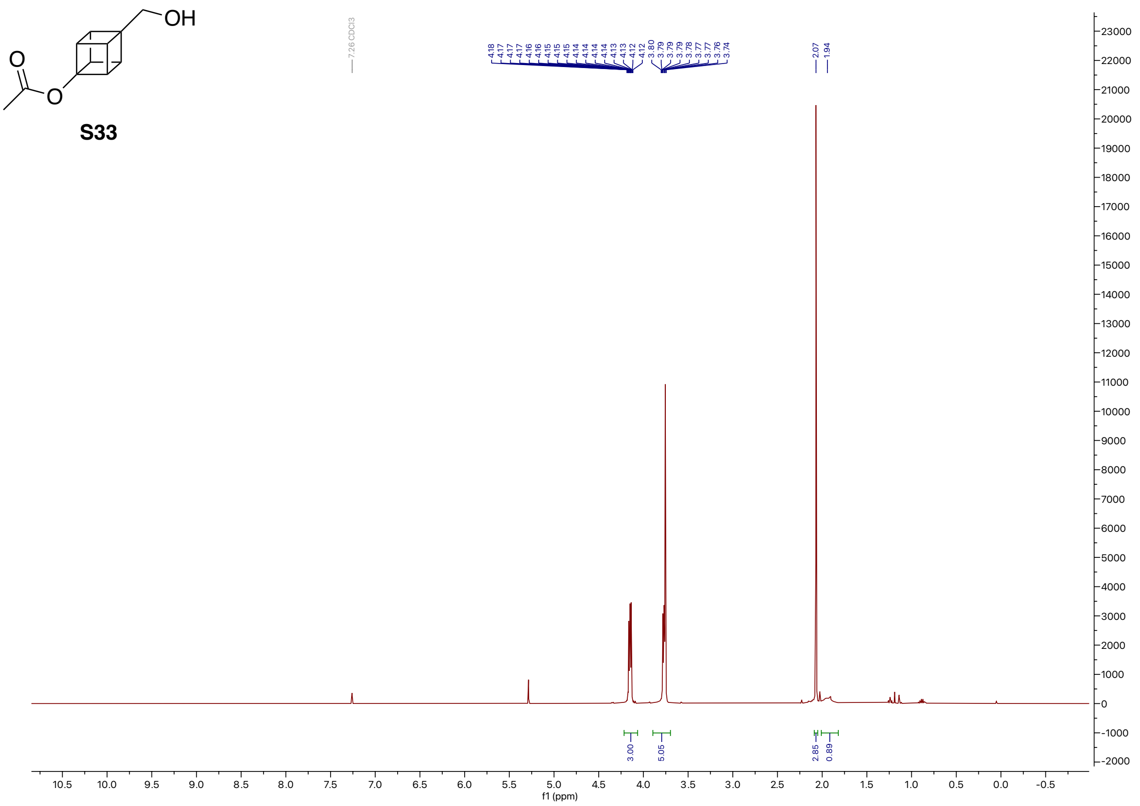

^{13}C NMR (101 MHz, $CDCl_3$) of S33

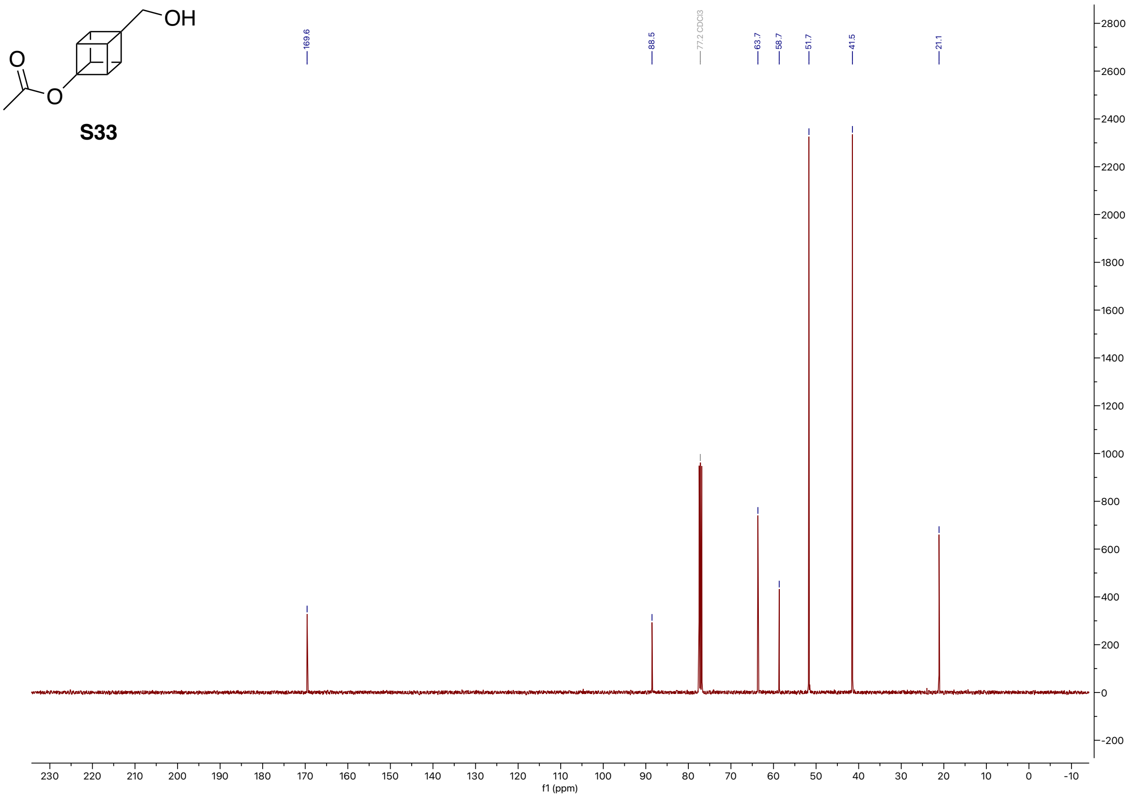

^{1}H NMR (400 MHz, CDCl$_3$) of 7

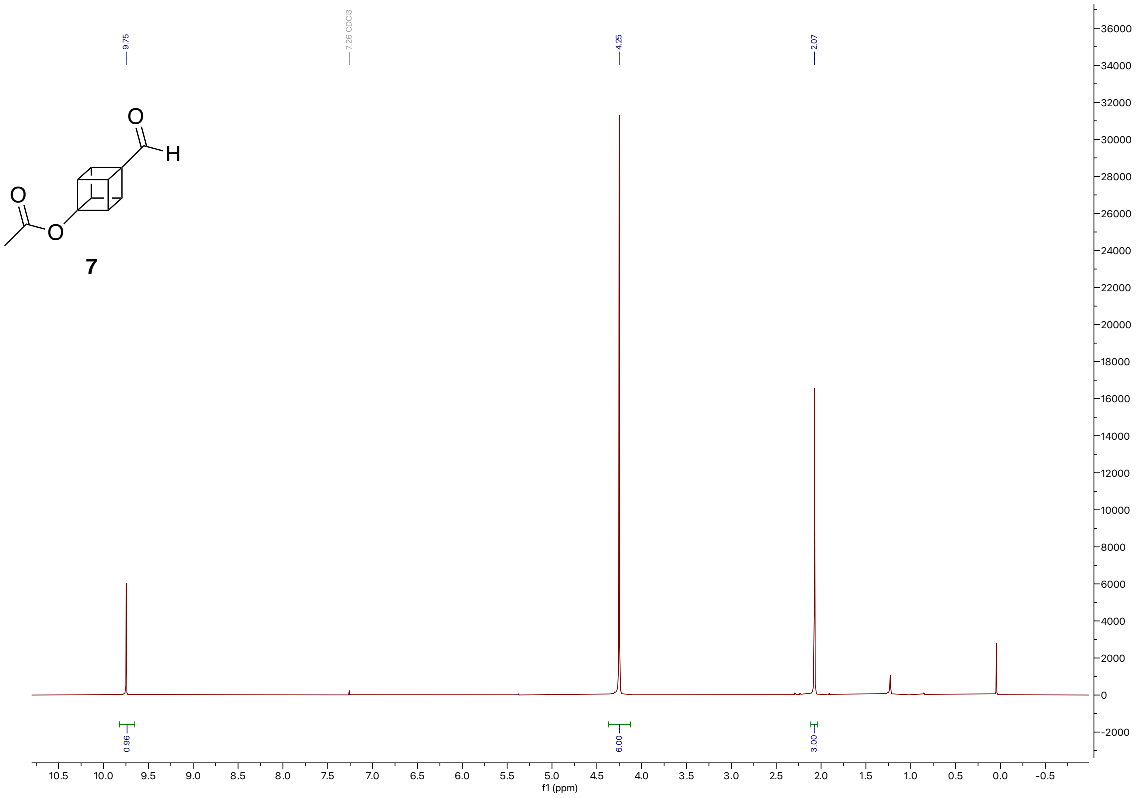

^{13}C NMR (101 MHz, CDCl$_3$) of 7

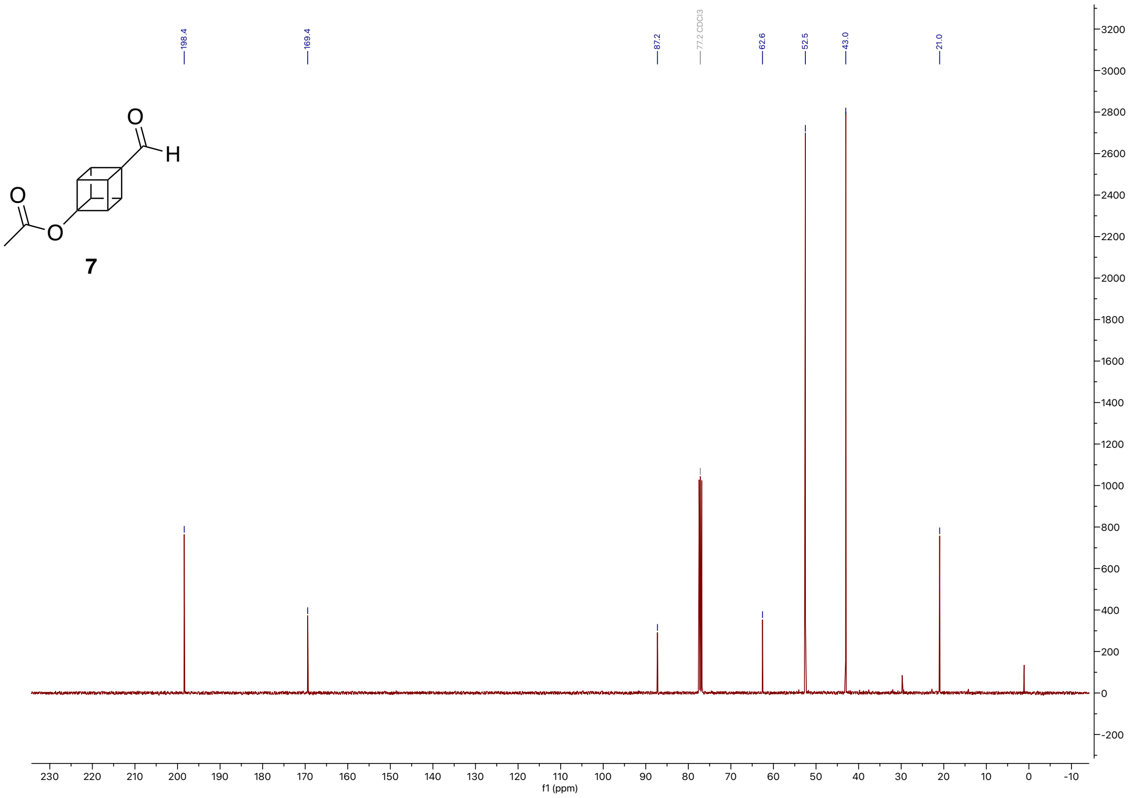

^{1}H NMR (400 MHz, Acetone-d$_6$) of S35

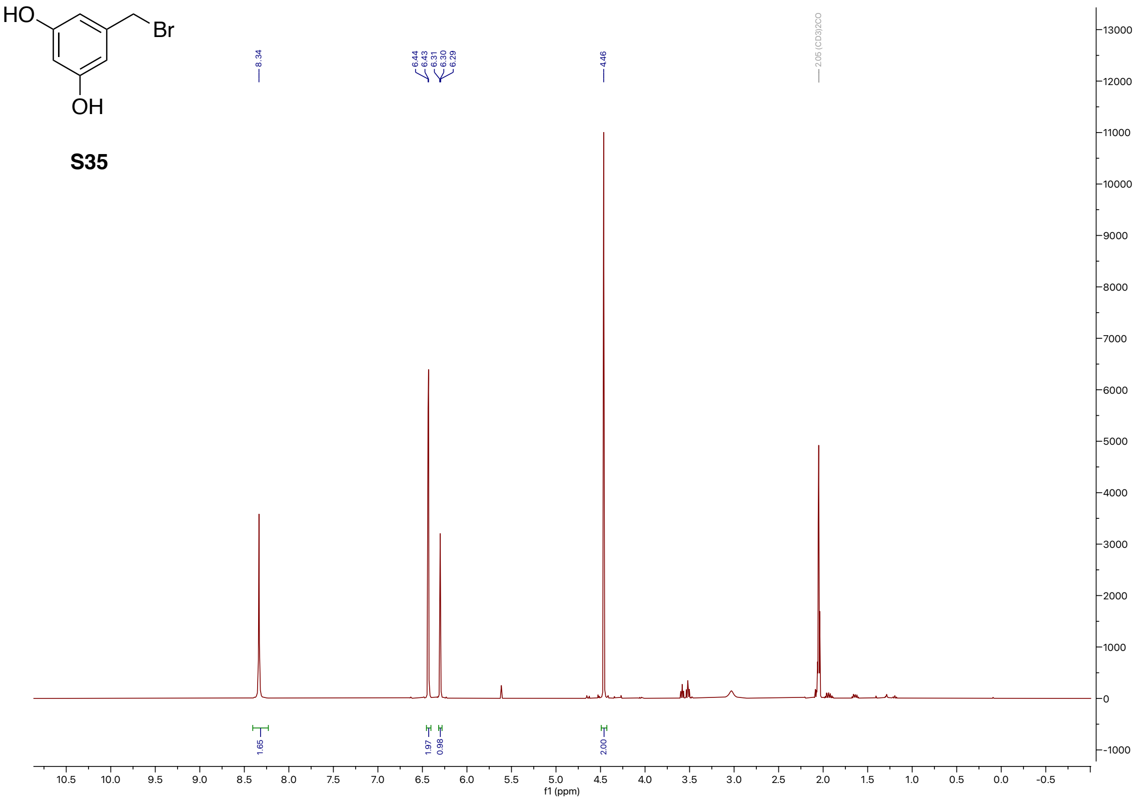

^{13}C NMR (101 MHz, Acetone-d$_6$) of S35

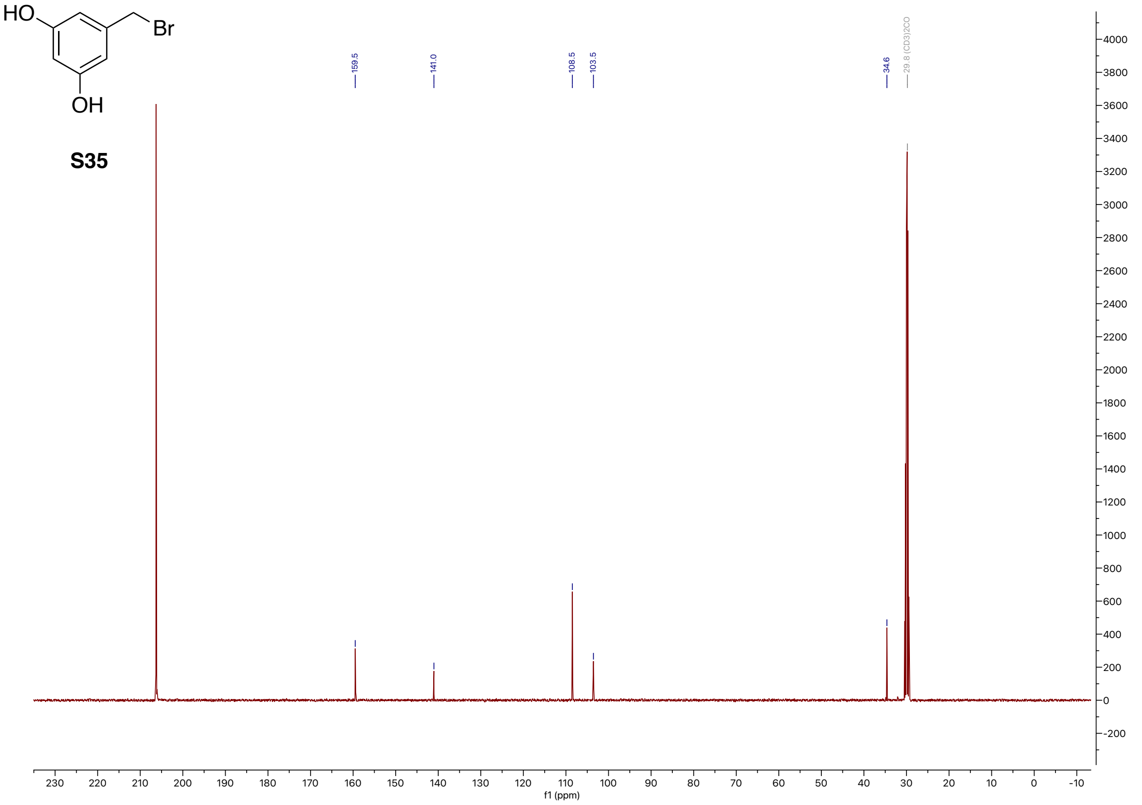

^{1}H NMR (500 MHz, DMSO-d6) of S36

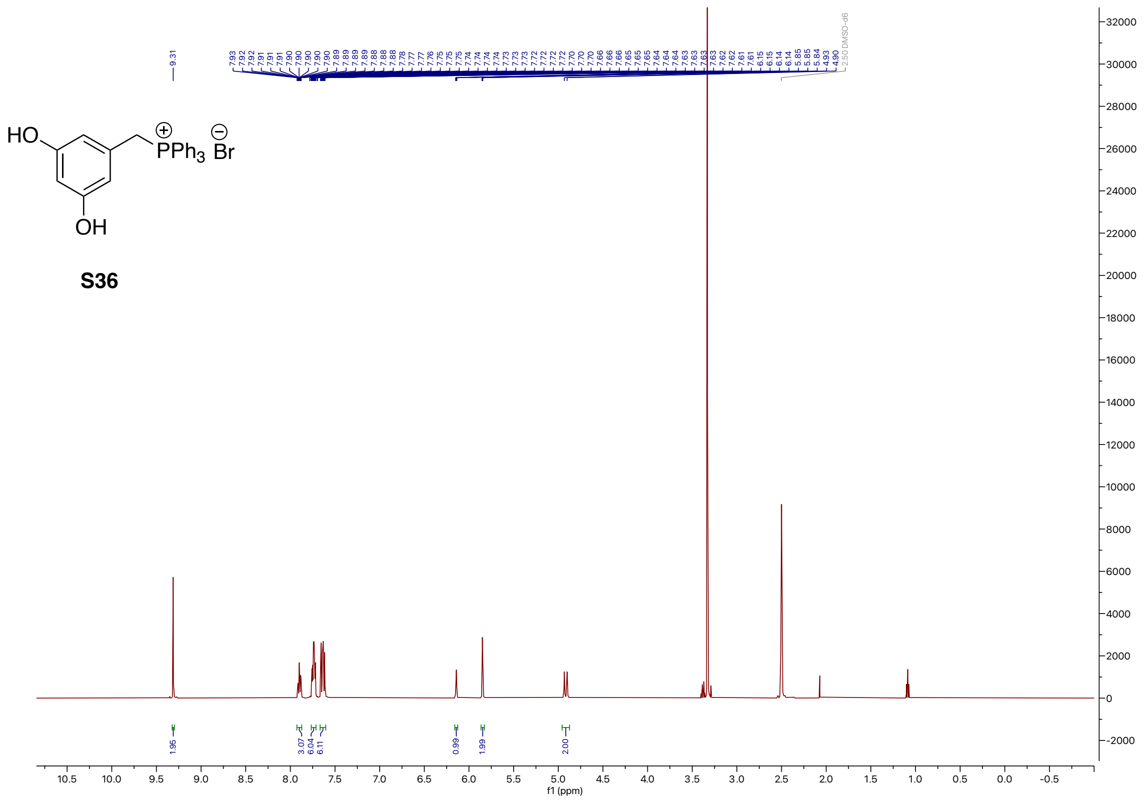

^{13}C NMR (126 MHz, DMSO-d6) of S36

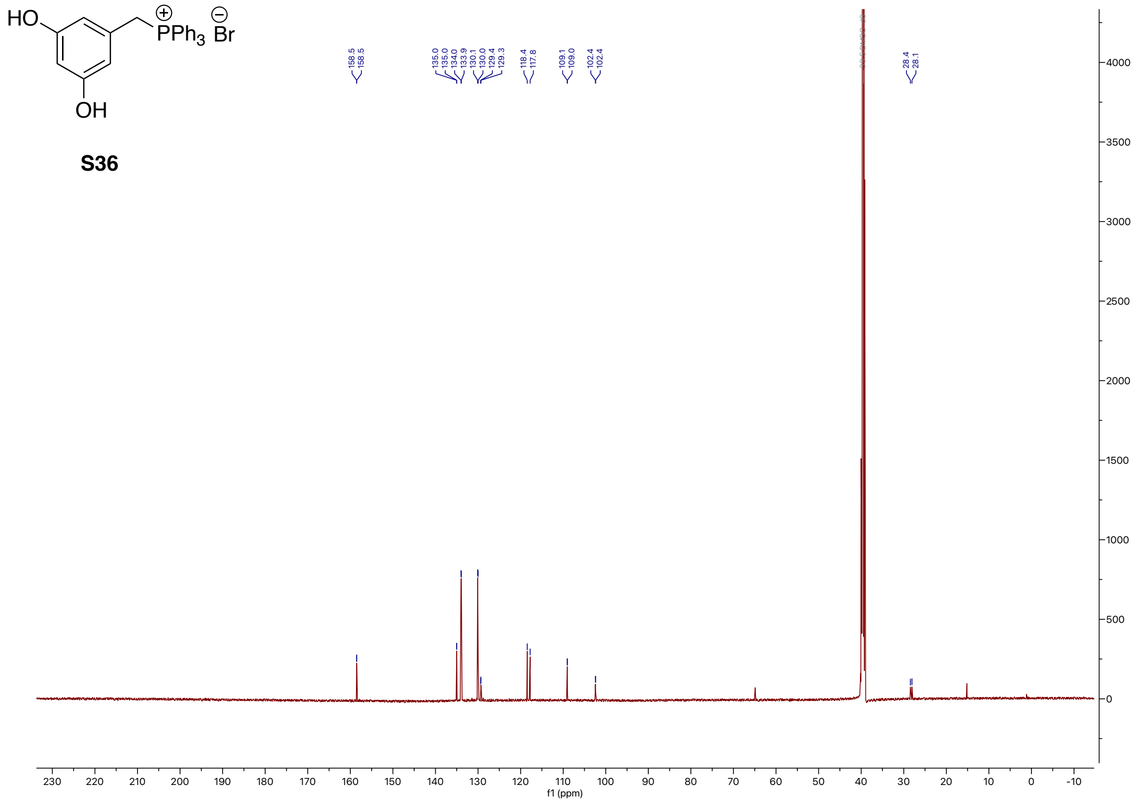

^{31}P NMR (162 MHz, DMSO-d6) of S36

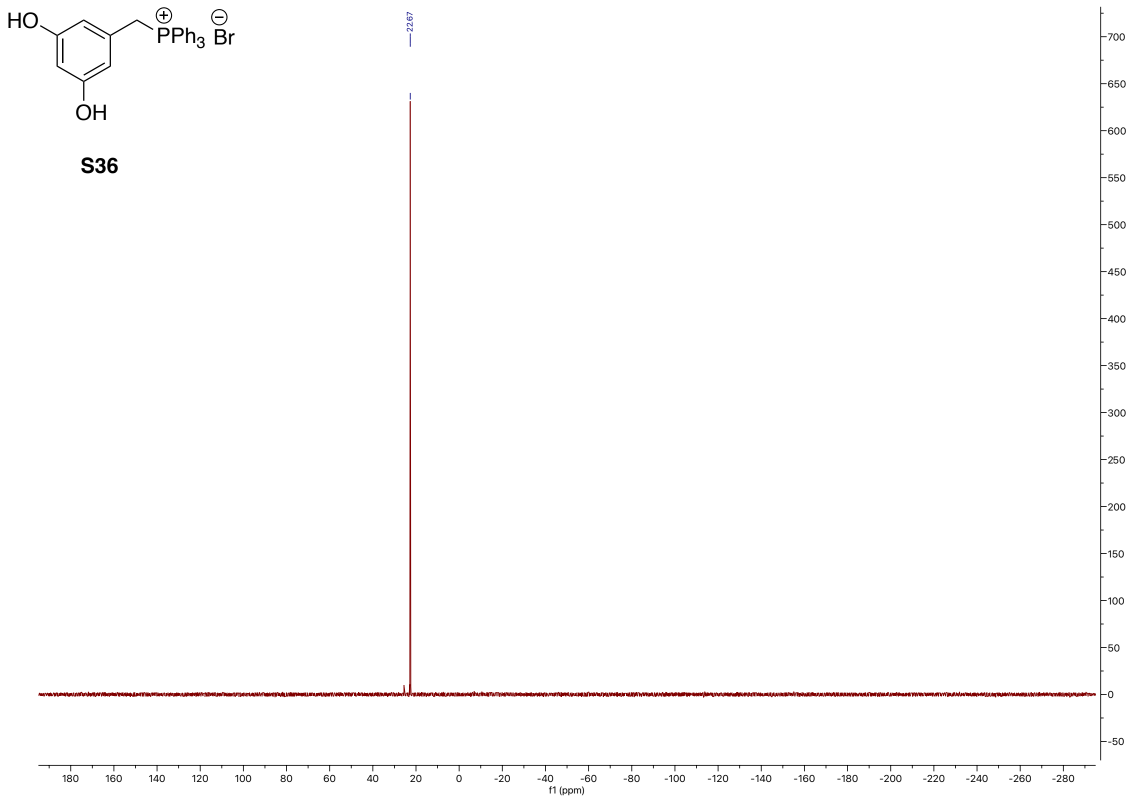

^{1}H NMR (500 MHz, DMSO-d6) of S37

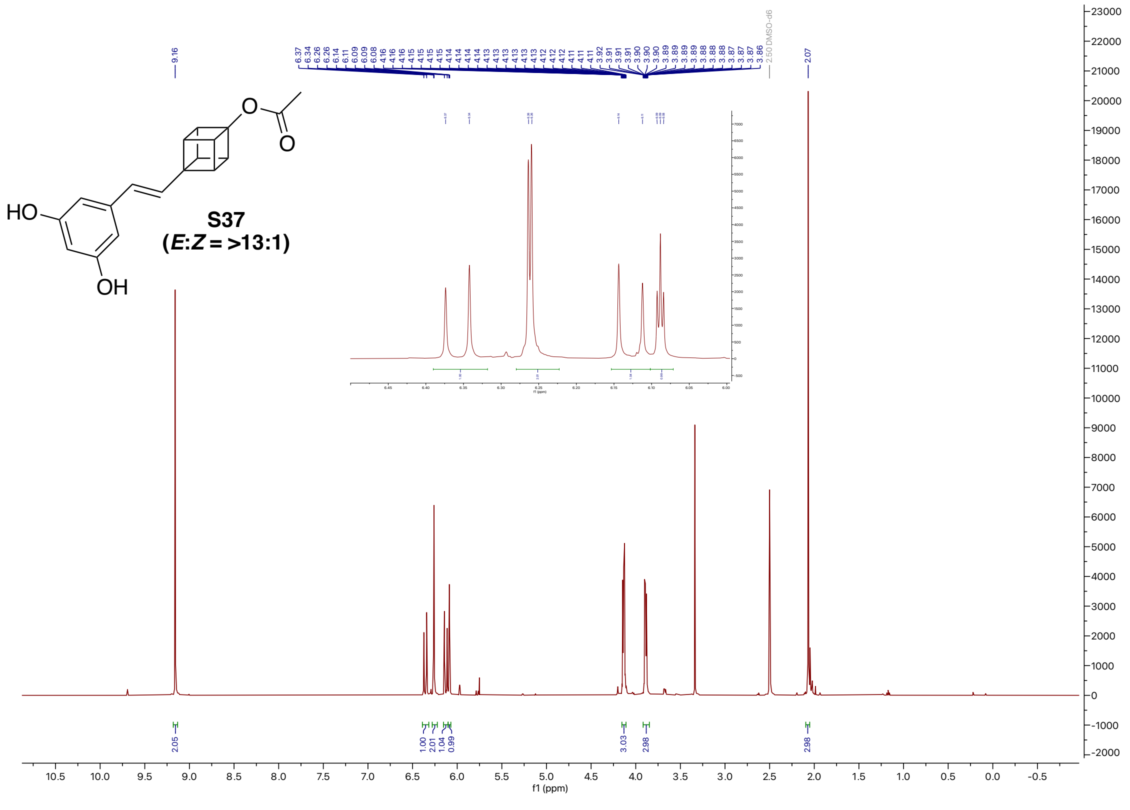

^{13}C NMR (126 MHz, DMSO-d6) of S37

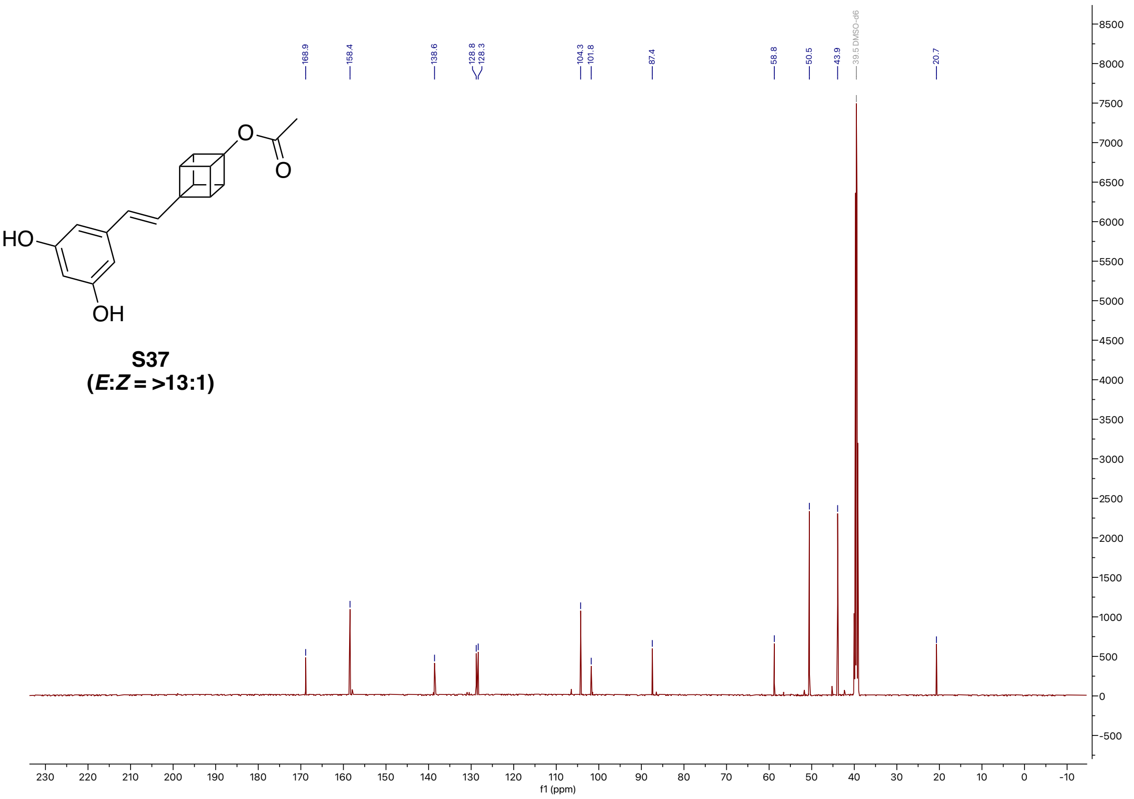

S37
(***E***:***Z*** = >13:1)

^{1}H NMR (500 MHz, DMSO-d6) of 8

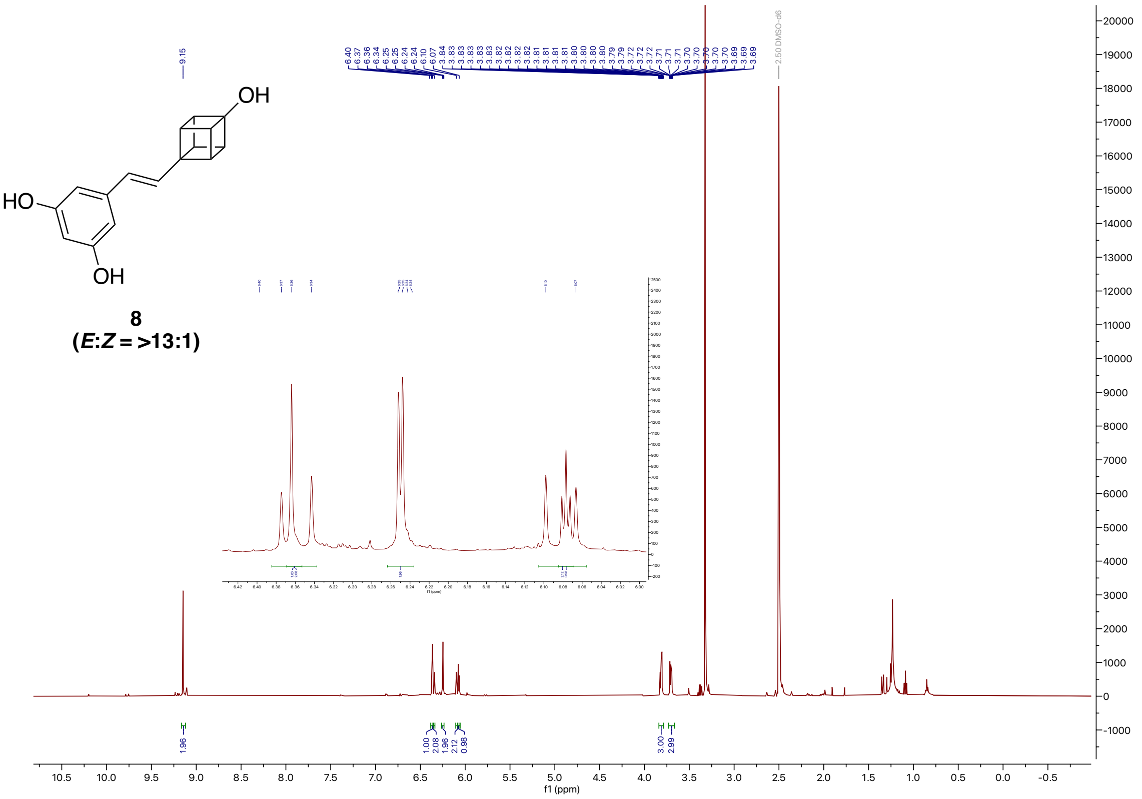

8
(***E***:***Z*** = >13:1)

^{13}C NMR (126 MHz, DMSO-d6) of 8

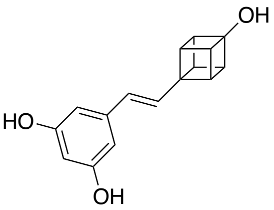

8
(*E*:*Z* = >13:1)

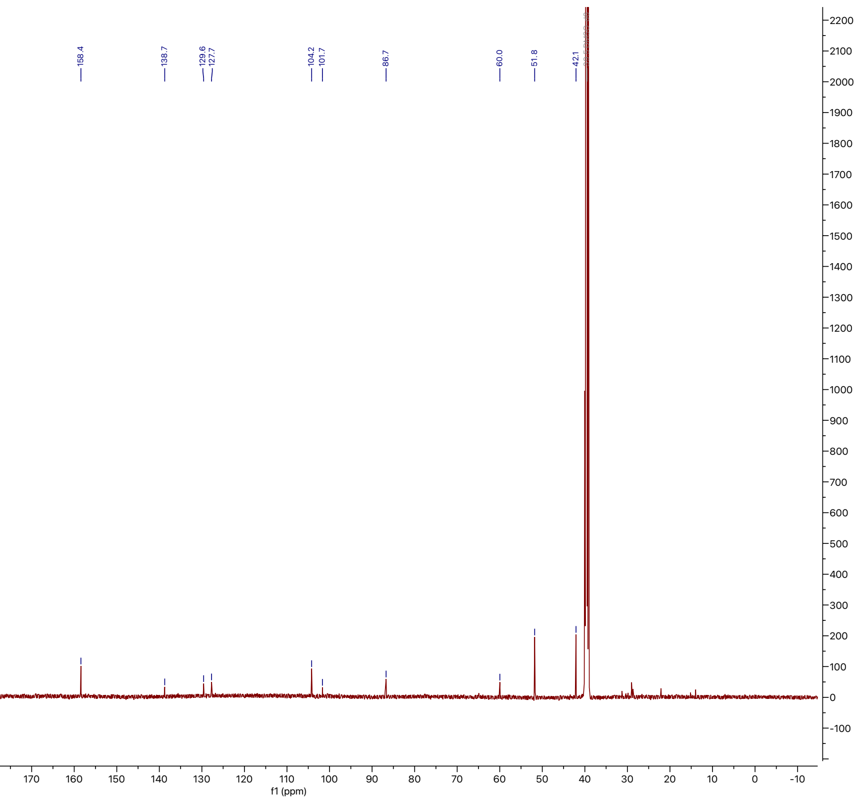

Supplement: Supplementary file 1 [file ol5c02760_si_001.pdf]
